# Ecological Consequences of Sediment on High-Energy Coral Reefs

**DOI:** 10.1371/journal.pone.0077737

**Published:** 2013-10-04

**Authors:** Christopher H. R. Goatley, David R. Bellwood

**Affiliations:** Australian Research Council Centre of Excellence for Coral Reef Studies, and School of Marine and Tropical Biology, James Cook University, Townsville, Queensland, Australia; University of Pennsylvania, United States of America

## Abstract

Sediments are widely accepted as a threat to coral reefs but our understanding of their ecological impacts is limited. Evidence has suggested that benthic sediments bound within the epilithic algal matrix (EAM) suppress reef fish herbivory, a key ecological process maintaining reef resilience. An experimental combination of caging and sediment addition treatments were used to investigate the effects of sediment pulses on herbivory and EAMs and to determine whether sediment addition could trigger a positive-feedback loop, leading to deep, sediment-rich turfs. A 1-week pulsed sediment addition resulted in rapid increases in algal turf length with effects comparable to those seen in herbivore exclusion cages. Contrary to the hypothesised positive-feedback mechanism, benthic sediment loads returned to natural levels within 3 weeks, however, the EAM turfs remained almost 60% longer for at least 3 months. While reduced herbivore density is widely understood to be a major threat to reefs, we show that acute disturbances to reef sediments elicit similar ecological responses in the EAM. With reefs increasingly threatened by both reductions in herbivore biomass and altered sediment fluxes, the development of longer turfs may become more common on coral reefs.

## Introduction

Coral reefs are among the most biodiverse ecosystems on the planet. Despite the ecological and physical complexity associated with this biodiversity, they are remarkably fragile. The increasing rate of disturbances associated with climate change and other anthropogenically induced stressors has repeatedly highlighted that acute stressors can have long lasting impacts [1,2]. For example, temperature anomalies lasting for very short periods can result in bleaching events, which have long-term effects at reef-wide scales [3]. While temperature anomalies might be the best understood, there are many other acute threats to coral reefs. These include storms, freshwater inundation and, potentially, sediments.

The source of sediment on coral reefs affects its physical and chemical properties. Fine marine (primarily carbonate) and terrigenous (primarily silicate) sediments can be transported long distances in suspension and/or driven by hydrodynamic activity [4]. While these sediments can be particularly deleterious to coral reefs, blocking light penetration and, upon settling, producing blankets of smothering silt [5], their affects tend to be best-documented on near-shore reefs [6]. The proximity to the sources of terrigenous sediments and shallow waters allow sediments to be effectively resuspended and transported to these reefs [4]. On reefs further from shore, however, most sediment is produced *in situ* as the reef matrix is eroded into sediments by physical, biological and, chemical processes [7].

While benthic sediments are highly dynamic on offshore coral reefs [8,9]. The continual production of sediments through erosion is often balanced by hydrodynamically driven losses to deep water or accretion into cays or beaches [8]. As such, although the sediment is constantly moving, the system remains in a state of dynamic stability [10,11], with all but the most severe perturbations being short lived [12].

On hard coral reef substrata some sediment exists in sand patches, but a large proportion of the benthic sediment is bound within algal turfs [13–16]. The turfs also trap detritus and provide habitat for infaunal organisms [17,18]. Together, these components form an epilithic algal matrix (EAM; [19]); a ubiquitous feature of coral reefs, often occupying more benthic space than corals [16,20]. On the Great Barrier Reef, EAMs cover between 18 and 59% of the reef surface [21]. While EAMs can play positive roles on coral reefs, with some components providing settlement cues for corals [22] and others directly adding to carbonate accretion [23], they also have several deleterious impacts. Algae within the EAMs can compete with corals for space [24,25], increase sediment deposition [26,27] and reduce the settlement success and survivorship of juvenile corals [28,29]. The algal turfs in EAMs can also represent an early stage in successional growth of macroalgae on reefs [30,31].

On ‘healthy’ coral reefs, EAMs are maintained as well-cropped productive turfs by herbivores, usually reef fishes [32]. The intensity of herbivory by fishes on these reefs can control the growth of algae [33–35]. While herbivory is clearly a vital function on coral reefs, recent evidence suggests that sediments may affect reef fish communities [36,37], and furthermore, sediments trapped in the EAM can deter herbivores from feeding [10,11]. Sediment reductions result in almost instantaneous increases in rates of reef fish herbivory and measurable declines in algal turf length in less than four hours [10]. The increased herbivory with reduced sediment loads indicates that trapped sediments actively deter herbivores, however, evidence for sediment suppression of herbivory remains almost entirely based on sediment removal experiments, which report increasing herbivory following sediment reductions [10,11]. Yet questions remain: a) will an increase in sediments produce similar changes in herbivory? And b) are these effects long-lasting or temporary? This is particularly interesting as increased sediments are believed to pose an ongoing threat to coral reefs [5,38].

The interactions between benthic sediment and herbivory suggest the possibility of a positive feedback loop. Any disturbance leading to increased EAM sediment load, such as exposure to a sediment plume from storms, flooding or dredging could deter herbivores. The reduced top down pressure on the EAM is likely to result in an increase in EAM length [10], allowing further sediment trapping [27,35]. The potential result is a self-sustaining, long, sediment-rich EAM, unpalatable to herbivores. Using a combination of herbivore exclusion cages and pulsed sediment additions we attempt to initiate this positive feedback and generate a deep, sediment-rich EAM. In doing so, we will provide information on the relative impacts of sediment pulses and herbivore removal on coral reef resilience.

## Materials and Methods

### Ethics statement

All procedures were conducted according to the ethics guidelines of James Cook University, Townsville (ethics approval A1522), and permitting requirements of the Great Barrier Reef Marine Parks Authority (permit number: G10/33755.1). Data are available in the supplementary material (Table S1).

### Experimental procedures

Experimental manipulations were conducted at two sites on the exposed reef crest southeast of Lizard Island in the northern section of the Great Barrier Reef, Australia. The experiment was conducted between December and March, in the southern hemisphere summer. The sites had similar topographies, were at a depth of 2-4 m, and separated by over 800 m. Sites were characterised by sparse coral colonies, separated by expanses of short well-cropped EAMs on a flat, low-complexity reef matrix. At each site, six fully caged, six open (uncaged) and six partially caged 1 m^2^ plots were haphazardly delineated on the flat EAM. Full cages were constructed of a steel bar frame, 25 cm high, enclosing the 1 m^2^ plot, attached to the reef using epoxy putty and enclosed with galvanised 4 cm wire mesh. Partial cages were constructed of vertical panels identical to those used in the fully caged treatments and were deployed to control for any effect of the cage treatments on the EAM or sediment, while allowing herbivores access to graze the EAM.

Half of the plots in each cage treatment were subjected to an experimental sediment addition. In these addition plots, a sediment load of 8.6 kg m^-2^ was maintained for one week. This sediment load was equivalent to that on the adjacent reef flat (approximately 40 m leeward of the crest), which was determined by weighing dried sediment samples collected using an electric vacuum sampler from 20 replicate 5.8 × 10^-3^ m^2^ rings (see [39]). The sediment for treatments was collected from a reef-margin sand apron and had a similar particle size profile to that found on the reef flat. When evenly spread, the 8.6 kg of sediment covered the 1 m^2^ plot to a depth of 15 mm. Sediment was added daily to each plot to maintain this depth for one week, simulating an acute sedimentation event. Then the plots were left undisturbed for the remainder of the three-month experimental period.

The sediment depth and EAM turf length in each plot were recorded using the depth probe of vernier calipers (n = 20 reps for each plot). Measurements were made as sediment was initially manipulated (*T*_0_), then at weekly intervals for 6 weeks post-manipulation (*T*_1-6_), and again after three months (*T*_7_).

EAM turf length data were standardised to percent increase over initial turf length (at *T*_0_) correcting for initial variation among plots. The data were then analysed using 2- and 3-way analyses of variances (ANOVAs) of the *T*_7_ data. Site was categorised as a random factor and was found to have no significant effect or interaction. It was therefore pooled to increase the power of the analysis [40,41]. Data normality was assessed using residuals plots and square-root transformations were applied where necessary. To allow data transformation for statistical analyses, negative percent EAM turf length values were removed by the addition of a constant to all data. Time series data were analysed using 2- and 3-way repeated measures ANOVAs (RM ANOVAs). Multivariate comparisons (Pillai’s Trace; RM MANOVAs) were used if assumptions of sphericity were violated.

## Results

**Figure 1:**
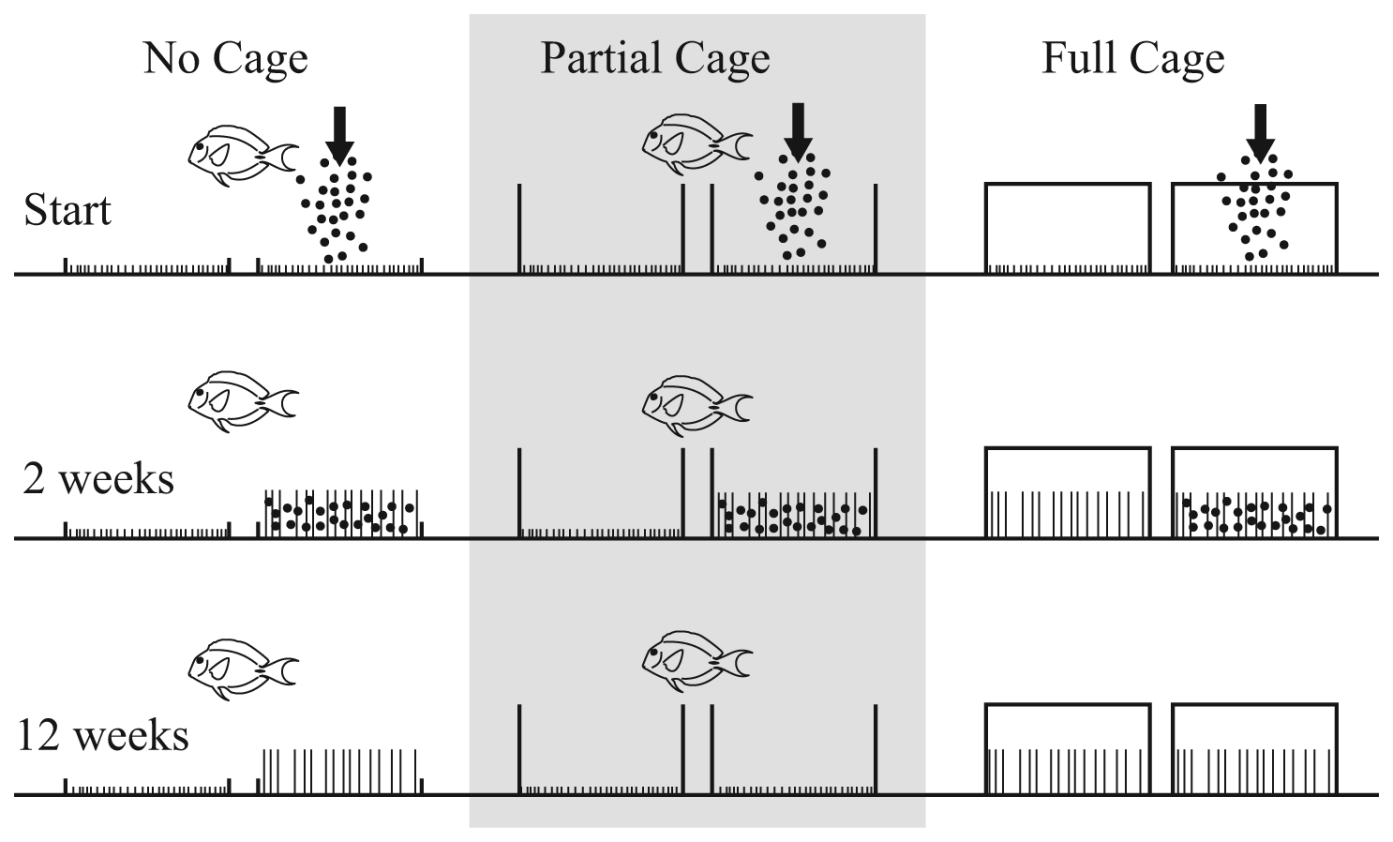
The effects of the experimental manipulations, at sediment addition, 2 and 12 weeks. Black circles represent added sediment and vertical lines the relative length of turfs in the EAM. Fishes indicate where herbivores had access to the plots.

**Figure 2:**
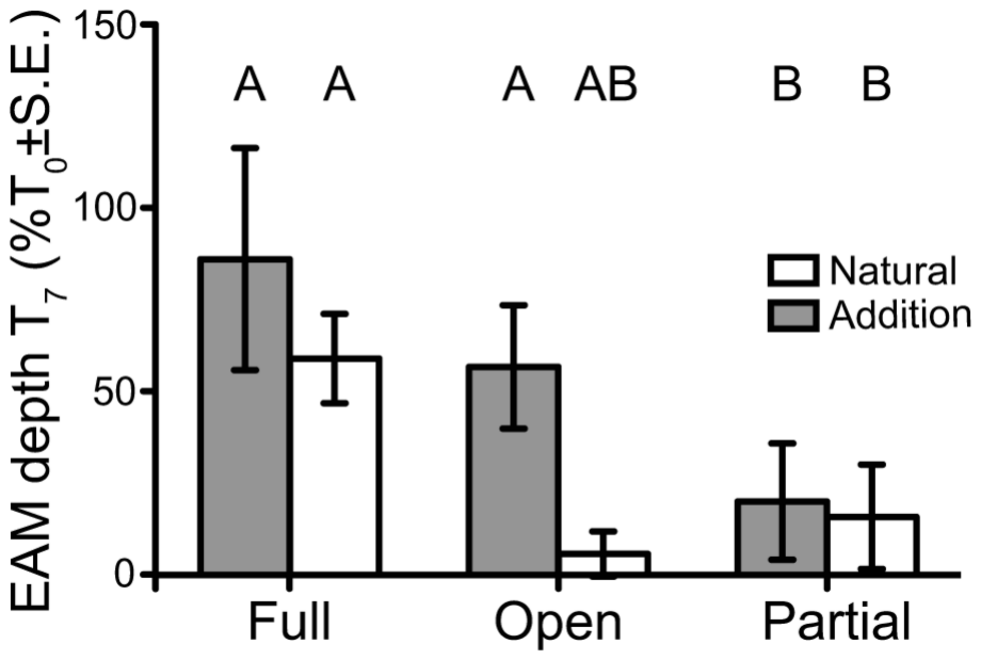
EAM turf lengths at T_7_ (3 months post sediment addition). Filled bars represent sediment addition treatments and open bars natural sediment treatments. A and B denote significant groupings (Fisher’s LSD). Data are shown as percentage of T_0_ ± standard error, sites are pooled, therefore n = 6 replicates for each.

After three months (*T*_7_) the greatest increase in the length of algae in the EAM was seen in the caged plots and a significant difference in turf length was observed between the caged and open plots (Figure 1; 3-way ANOVA on square root transformed data: *F*_2,24_ = 7.32, *P* = 0.003). While no other factors or interactions were significant (Table S2), a Fisher’s least significant difference (LSD) post-hoc test revealed two groupings in the caging × sediment treatment interaction (Figure 2). Without sediments, a clear cage effect was observed. Algal length increased by 58.9%; no response was seen in the open or partial cages.

Sediment additions to caged and partially caged plots resulted in no observable differences to the EAM when compared to the corresponding natural sediment plots. Sediment additions to the open plots, however, resulted in remarkable EAM turf algae growth, i.e., over 435% greater than observed on open, natural sediment treatment plots (Figure 2).

**Figure 3:**
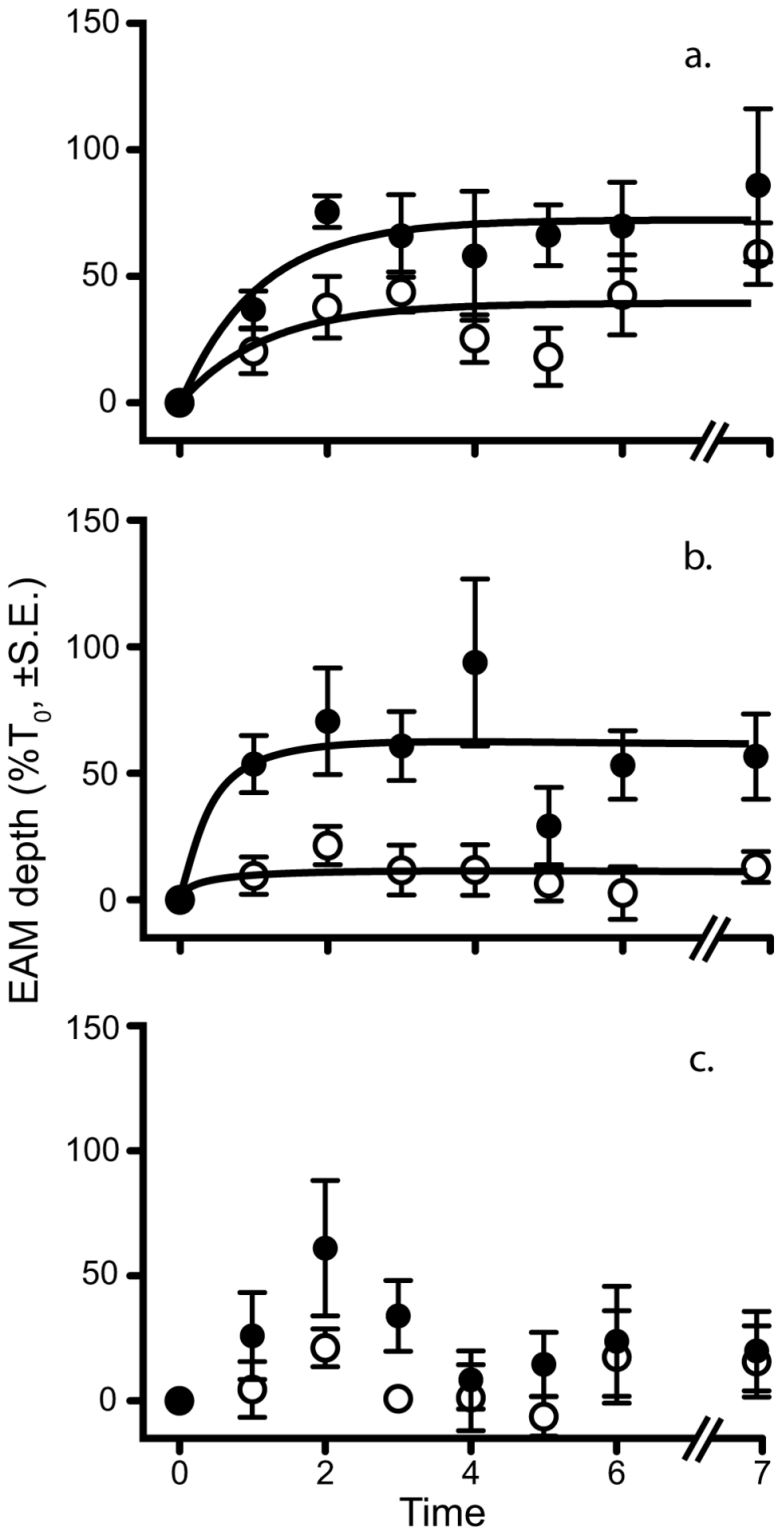
EAM turf length over time. Data are pooled between sites and standardized as percentage of T_0_. Filled circles represent sediment addition, open circles represent natural sediment loads. Time is measured in weeks except T_7_, which is three months after T_0_. a. shows the responses of the caged plots, b. the open plots, and c. the partially caged plots. Data are shown as percentage of T_0_ ± standard error, with sites pooled, n = 6 replicates for each point. The lines fitted follow a one-phase decay model where appropriate.

The time series data showed an initial rapid increase in turf length followed by an apparent asymptote after approximately three weeks (Figure 3). Data differed significantly over time (RM MANOVA; Pillai’s Trace = 0.647, *F*_6,25_ = 7.639, *P* <0.0001) and in the time × caging interaction (Pillai’s Trace = 0.636, *F*_12,52_ = 2.022, *P* = 0.041). No other factors showed significant interactions (Table S3).

**Figure 4:**
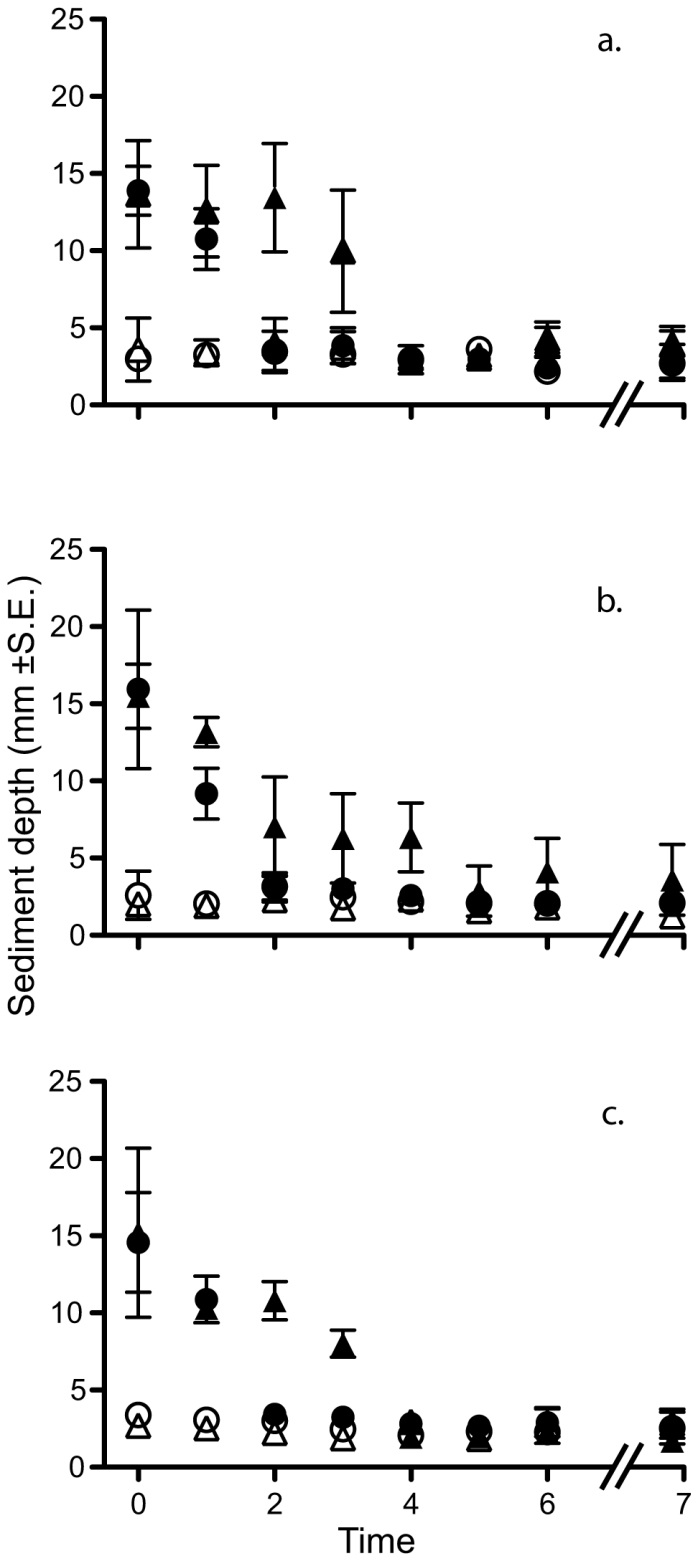
Sediment depth over time. Filled points represent sediment addition treatments, open points represent natural sediment treatments. Circles represent data from site 1, and triangles site 2. Time is measured in weeks except T_7_, which is three months after T_0_. a. shows data from the caged plots, b. the open plots, and c. the partially caged plots. Data are shown as mean depth in mm ± standard error, n = 3 replicates for each.

Direct measurements of the sediment loads in the EAMs were conducted alongside measurements of the EAM turf length. Unlike the turf length, sediment rapidly declined in all treatments to levels indistinguishable from natural sediment loads within three weeks (Figure 4; Table S4).

## Discussion

We found that a one-week pulsed increase in sediment loads resulted in significant and sustained (3-month) increases in the length of turfs in EAMs on a coral reef (Figure 1). However, our results provide only limited support for the existence of the proposed positive feedback loop. Although the length of turfs increased, high hydrodynamic activity on the crest (e.g. [42]) quickly removed sediment from the turfs and the predicted deep, sediment-rich turfs were not formed. Increased EAM turf length did not appear to require high sediment loads.

The EAMs on the exposed crest used in this study are heavily grazed by coral reef fishes, which maintain short, productive algal turfs [43]. Previous studies have demonstrated that when EAM with long turfs are transplanted from the reef flat (low herbivore pressure) to the crest (high herbivore pressure) they are rapidly consumed by fishes [13,35]. It was, therefore, expected that any sediment-induced increase in the length of EAM turfs would be rapidly reversed as sediments returned to normal levels, as a result of hydrodynamic activity, releasing the algae from any sediment-induced suppression of herbivory (e.g. [10,11]). This was not the case.

The EAMs in the present study grew in response to reduced herbivory (cages), increased sediments, or a combination of these factors. Growth in the cages was probably a result of the reduction in grazing [33,35]. While sediment addition may increase algal turf length as a result of the algae investing greater energy resources in linear extension to reach light [44] and/or increased availability of nutrients desorbed from the sediment [13,45]. In both cage and sediment treatments the algal growth was rapid and occurred soon after the initial manipulations were made, while any added sediment was still present. The growth of the EAMs slowed rapidly as sediment loads diminished. Notably, there was no growth of macroalgae, only an increase in turf length. Whether the time for macroalgal development was insufficient, if propagules or juveniles were absent or if the high hydrodynamic activity at this site limited development of macroalgae [10,42], the key result is that responses of algal turfs with artificially reduced herbivory (cages) were comparable to those with increased sediment loads.

Remarkably, after the initial growth of the algal filaments the new, longer, EAMs remained for at least three months in both caged and open plots. The three treatments followed different trajectories: (1) by reducing herbivory, cages maintained a long EAM regardless of sediments (as in previous studies, e.g. [31,34]). (2) In open plots, EAMs likewise remained long, but only if exposed to sediment. This was particularly striking given the rapid loss of sediment. It may be that the vertical distribution of the remaining sediments through the EAM plays an important role in deterring herbivores, or that changes in the microbial community [46,47] or detrital load of the turfs [48] made them less palatable to herbivores. The methods used in this study, however, could not detect any of these factors and, as such, further study is necessary to determine what is underpinning this stability. However, the lack of recovery is worrying as it highlights that reefs are slow to recover from sediment-induced disturbances, even short, ‘pulsed’ events. (3) The partially caged plots followed similar initial growth trajectories to their equivalent open plots, however, the longer EAMs appeared to ‘recover’ (i.e. reduce in length) as sediment was removed. The apparent ‘recovery’ of the EAM in the partial cages was unexpected. It was expected that, as herbivores had access to the benthos, the EAMs would follow the responses seen in the open plots. However, this difference appears to be a result of increased herbivory in the partial cages. The partial cages increased topographic complexity, and provided shelter for a range of reef herbivores allowing localised increases in grazing (cf. [49–52]).

We found that the growth of EAMs after a one-week sediment pulse was equivalent to, or greater than, that seen in simulated herbivore removals using cages. While increased sediment is often considered deleterious to reefs, most information pertaining to its effects are at a physiological scale, considering the impact on individual organisms [53]; little is known about how sediments affect the broader ecosystem functions on which coral reefs rely [54]. At physiological scales, sediments have deleterious impacts on almost all benthic reef taxa [53], however the role of sediments in mediating reef processes is currently limited to studies addressing the impact of sediment laden EAMs in driving settlement and survivorship of corals. While some algae within EAMs may induce settlement [22] increased sediments result in reduced settlement of reef corals as they create a mobile substrate, preventing access to the consolidated reef matrix [14,29]. Furthermore, benthic sediments reduce survival of post-settlement corals particularly during their early development presumably due to abrasion and shading [29,55]. In addition to affecting benthic coral reef organisms, our findings suggest that increased sediments might suppress herbivory resulting in the development and persistence of longer EAMs driving further implications for reef ecosystems. Reduced herbivory through increased fishing pressure is widely publicised as a major threat to coral reefs on a global scale [56], but it appears that short-term increases in sediment release or resuspension through anthropogenic activities (e.g. [5,38,57]) have the potential to have similar effects at localised scales.

The manipulation used in this study is comparable to that of other short-term (acute) perturbations caused by natural or anthropogenic disturbances. Storms and cyclones can move sediment from areas with high natural loads, such as lagoons or reef flats, which, as with this study site, may be only a few tens of meters away [58]. Storms can also increase runoff to inner and mid-shelf reefs [8,9,59]. Similarly, anthropogenic sediment perturbations caused by dredging and other shallow-water maritime activities can resuspend sediments [5,60,61]. These events may all generate long EAMs similar to those seen after the experimental sediment manipulation. An even greater impact may be observed if these events happen in succession, without sufficient recovery time. The longer EAMs generated in this study persisted for at least 3 months, with no obvious sign of recovery. If disturbances increasing sediment loads occurred more often than this, there is the risk that the turfs may grow longer still and persist for longer periods. An assessment of the recovery time is necessary to assess whether such a ratchet effect (*sensu* [62]) is likely to lead to the disruption of ecological processes on reefs.

It is essential to note that our observations are of the effects of ‘natural’ carbonate dominated sediments, which characterise offshore coral reefs. The disturbance induced by this study, therefore, represents the best-case scenario for the effects of sediments on reefs. Terrigenous sediments, with higher silicate and nutrient loads alongside a high likelihood of associated pollutants (e.g. from metals, chemical fertilisers and pesticides), are likely to have even greater impacts [38,63].

Although our study did not find evidence to support the existence of a positive feedback leading to sediment-laden turfs, a clear and lasting effect was observed following an acute disturbance, with the development of a long-turfed EAM. Longer EAMs on reefs have the potential to reduce coral settlement [29], provide inferior grazing surfaces for fishes [10,11], and present an ecologically and economically less-desirable state [2]. The development of these long EAMs appears to be driven by increases in both benthic sediments and reductions in herbivory. In a world where reefs are affected by increasingly unpredictable climatic conditions and chronic reductions in herbivore abundances, these longer EAMs are likely to become an increasingly common feature on coral reefs.

## Supporting Information

Table S1 **Raw data.** ‘Time’ column is measured in weeks. Plots 1-9 are site 1 and 10-18 site 2. ‘Turf/Sed’ column indicates what is being measured.
(PDF)

Table S2 **2-way ANOVA of T7 turf length data (square root x + 21.0633 transformed data, sites pooled).**
(PDF)

Table S3 **RM MANOVA results of EAM depth over time, across caging and sediment treatments, pooled between sites (Figure 3).** Assumptions of sphericity were violated by univariate tests, as such multivariate tests (Pillai’s Trace) were used.
(PDF)

Table S4 **RM MANOVA results of sediment depth over time, across caging and sediment treatments, pooled between sites (Figure 4).** Assumptions of sphericity were violated by univariate tests, as such multivariate tests (Pillai’s Trace) were used.
(PDF)
