# Supplementary material for: Ecological Consequences of Sediment on High-Energy Coral Reefs
Source: PLoS One. 2013 Oct 4;8(10):e77737. doi: 10.1371/journal.pone.0077737 (PMC3790735; doi:10.1371/journal.pone.0077737)
Supplement: Table S1 — Raw data. ‘Time’ column is measured in weeks. Plots 1-9 are site 1 and 10-18 site 2. ‘Turf/Sed’ column indicates what is being measured. (PDF) [file pone.0077737.s001.pdf]

Table S1: Raw data. ‘Time’ column is measured in weeks. Plots 1-9 are site 1 and 10-18 site 2. ‘Turf/Sed’ column indicates what is being measured.

Time	Plot	Cage	Sediment load	Turf/Sed	Substrate Depth (mm)
0	1	Full	Natural	Sed	2.40
0	1	Full	Natural	Sed	1.58
0	1	Full	Natural	Sed	2.38
0	1	Full	Natural	Sed	1.88
0	1	Full	Natural	Sed	3.04
0	1	Full	Natural	Sed	3.80
0	1	Full	Natural	Sed	2.84
0	1	Full	Natural	Sed	2.28
0	1	Full	Natural	Sed	0.00
0	1	Full	Natural	Sed	1.10
0	1	Full	Natural	Sed	6.24
0	1	Full	Natural	Sed	2.72
0	1	Full	Natural	Sed	3.90
0	1	Full	Natural	Sed	1.34
0	1	Full	Natural	Sed	2.02
0	1	Full	Natural	Sed	1.98
0	1	Full	Natural	Sed	1.54
0	1	Full	Natural	Sed	2.68
0	1	Full	Natural	Sed	0.00
0	1	Full	Natural	Sed	21.48
0	2	Full	Natural	Sed	3.69
0	2	Full	Natural	Sed	0.00
0	2	Full	Natural	Sed	0.61
0	2	Full	Natural	Sed	2.68
0	2	Full	Natural	Sed	4.76
0	2	Full	Natural	Sed	1.81
0	2	Full	Natural	Sed	4.20
0	2	Full	Natural	Sed	0.00
0	2	Full	Natural	Sed	1.60
0	2	Full	Natural	Sed	2.12
0	2	Full	Natural	Sed	7.74
0	2	Full	Natural	Sed	4.49
0	2	Full	Natural	Sed	1.03
0	2	Full	Natural	Sed	4.27
0	2	Full	Natural	Sed	3.37
0	2	Full	Natural	Sed	1.92
0	2	Full	Natural	Sed	5.17
0	2	Full	Natural	Sed	2.05
0	2	Full	Natural	Sed	2.30
0	2	Full	Natural	Sed	3.63
0	3	Full	Natural	Sed	2.86
0	3	Full	Natural	Sed	1.58
0	3	Full	Natural	Sed	3.62
0	3	Full	Natural	Sed	1.78
0	3	Full	Natural	Sed	2.24
0	3	Full	Natural	Sed	2.93
0	3	Full	Natural	Sed	2.70
0	3	Full	Natural	Sed	1.40
0	3	Full	Natural	Sed	5.66
0	3	Full	Natural	Sed	4.77
0	3	Full	Natural	Sed	4.12
0	3	Full	Natural	Sed	2.77

0	3	Full	Natural	Sed	2.28
0	3	Full	Natural	Sed	1.53
0	3	Full	Natural	Sed	4.87
0	3	Full	Natural	Sed	3.07
0	3	Full	Natural	Sed	1.74
0	3	Full	Natural	Sed	1.48
0	3	Full	Natural	Sed	2.58
0	3	Full	Natural	Sed	2.15
0	4	Full	Addition	Sed	19.65
0	4	Full	Addition	Sed	15.92
0	4	Full	Addition	Sed	3.10
0	4	Full	Addition	Sed	13.43
0	4	Full	Addition	Sed	6.99
0	4	Full	Addition	Sed	5.34
0	4	Full	Addition	Sed	6.59
0	4	Full	Addition	Sed	21.49
0	4	Full	Addition	Sed	14.71
0	4	Full	Addition	Sed	29.59
0	4	Full	Addition	Sed	10.00
0	4	Full	Addition	Sed	15.51
0	4	Full	Addition	Sed	12.93
0	4	Full	Addition	Sed	19.66
0	4	Full	Addition	Sed	15.22
0	4	Full	Addition	Sed	16.65
0	4	Full	Addition	Sed	14.24
0	4	Full	Addition	Sed	7.32
0	4	Full	Addition	Sed	5.76
0	4	Full	Addition	Sed	8.37
0	5	Full	Addition	Sed	11.36
0	5	Full	Addition	Sed	12.26
0	5	Full	Addition	Sed	25.17
0	5	Full	Addition	Sed	31.88
0	5	Full	Addition	Sed	19.94
0	5	Full	Addition	Sed	11.44
0	5	Full	Addition	Sed	5.60
0	5	Full	Addition	Sed	26.13
0	5	Full	Addition	Sed	29.73
0	5	Full	Addition	Sed	10.87
0	5	Full	Addition	Sed	15.37
0	5	Full	Addition	Sed	12.45
0	5	Full	Addition	Sed	24.39
0	5	Full	Addition	Sed	2.90
0	5	Full	Addition	Sed	15.64
0	5	Full	Addition	Sed	7.88
0	5	Full	Addition	Sed	14.27
0	5	Full	Addition	Sed	6.55
0	5	Full	Addition	Sed	13.49
0	5	Full	Addition	Sed	16.68
0	6	Full	Addition	Sed	11.22
0	6	Full	Addition	Sed	12.80
0	6	Full	Addition	Sed	17.69
0	6	Full	Addition	Sed	5.68
0	6	Full	Addition	Sed	5.08

0	6	Full	Addition	Sed	18.89
0	6	Full	Addition	Sed	36.15
0	6	Full	Addition	Sed	15.58
0	6	Full	Addition	Sed	1.40
0	6	Full	Addition	Sed	10.65
0	6	Full	Addition	Sed	7.48
0	6	Full	Addition	Sed	20.33
0	6	Full	Addition	Sed	3.95
0	6	Full	Addition	Sed	23.48
0	6	Full	Addition	Sed	8.56
0	6	Full	Addition	Sed	15.65
0	6	Full	Addition	Sed	5.54
0	6	Full	Addition	Sed	13.85
0	6	Full	Addition	Sed	7.64
0	6	Full	Addition	Sed	15.12
0	7	Half	Natural	Sed	6.64
0	7	Half	Natural	Sed	2.23
0	7	Half	Natural	Sed	2.58
0	7	Half	Natural	Sed	3.50
0	7	Half	Natural	Sed	1.95
0	7	Half	Natural	Sed	1.82
0	7	Half	Natural	Sed	3.32
0	7	Half	Natural	Sed	3.41
0	7	Half	Natural	Sed	2.97
0	7	Half	Natural	Sed	7.97
0	7	Half	Natural	Sed	6.13
0	7	Half	Natural	Sed	3.14
0	7	Half	Natural	Sed	1.76
0	7	Half	Natural	Sed	2.98
0	7	Half	Natural	Sed	1.86
0	7	Half	Natural	Sed	5.41
0	7	Half	Natural	Sed	6.62
0	7	Half	Natural	Sed	1.78
0	7	Half	Natural	Sed	1.27
0	7	Half	Natural	Sed	1.85
0	8	Half	Natural	Sed	1.24
0	8	Half	Natural	Sed	3.83
0	8	Half	Natural	Sed	5.19
0	8	Half	Natural	Sed	2.44
0	8	Half	Natural	Sed	1.98
0	8	Half	Natural	Sed	1.83
0	8	Half	Natural	Sed	8.68
0	8	Half	Natural	Sed	2.32
0	8	Half	Natural	Sed	1.78
0	8	Half	Natural	Sed	8.47
0	8	Half	Natural	Sed	2.59
0	8	Half	Natural	Sed	3.05
0	8	Half	Natural	Sed	5.04
0	8	Half	Natural	Sed	4.03
0	8	Half	Natural	Sed	2.02
0	8	Half	Natural	Sed	2.84
0	8	Half	Natural	Sed	3.17
0	8	Half	Natural	Sed	2.94

0	8	Half	Natural	Sed	2.22
0	8	Half	Natural	Sed	1.77
0	9	Half	Natural	Sed	1.57
0	9	Half	Natural	Sed	0.88
0	9	Half	Natural	Sed	6.28
0	9	Half	Natural	Sed	2.38
0	9	Half	Natural	Sed	5.98
0	9	Half	Natural	Sed	1.78
0	9	Half	Natural	Sed	5.30
0	9	Half	Natural	Sed	4.19
0	9	Half	Natural	Sed	1.61
0	9	Half	Natural	Sed	3.11
0	9	Half	Natural	Sed	3.49
0	9	Half	Natural	Sed	2.38
0	9	Half	Natural	Sed	4.00
0	9	Half	Natural	Sed	6.40
0	9	Half	Natural	Sed	3.47
0	9	Half	Natural	Sed	1.93
0	9	Half	Natural	Sed	3.61
0	9	Half	Natural	Sed	2.57
0	9	Half	Natural	Sed	3.26
0	9	Half	Natural	Sed	2.29
0	10	Half	Addition	Sed	7.46
0	10	Half	Addition	Sed	13.19
0	10	Half	Addition	Sed	12.26
0	10	Half	Addition	Sed	10.13
0	10	Half	Addition	Sed	7.41
0	10	Half	Addition	Sed	7.43
0	10	Half	Addition	Sed	8.50
0	10	Half	Addition	Sed	10.60
0	10	Half	Addition	Sed	41.08
0	10	Half	Addition	Sed	22.37
0	10	Half	Addition	Sed	17.93
0	10	Half	Addition	Sed	31.95
0	10	Half	Addition	Sed	15.54
0	10	Half	Addition	Sed	4.48
0	10	Half	Addition	Sed	8.59
0	10	Half	Addition	Sed	15.67
0	10	Half	Addition	Sed	4.47
0	10	Half	Addition	Sed	12.40
0	10	Half	Addition	Sed	17.81
0	10	Half	Addition	Sed	35.72
0	11	Half	Addition	Sed	12.67
0	11	Half	Addition	Sed	18.46
0	11	Half	Addition	Sed	3.02
0	11	Half	Addition	Sed	6.31
0	11	Half	Addition	Sed	7.88
0	11	Half	Addition	Sed	3.20
0	11	Half	Addition	Sed	16.29
0	11	Half	Addition	Sed	17.03
0	11	Half	Addition	Sed	7.65
0	11	Half	Addition	Sed	19.43
0	11	Half	Addition	Sed	3.28

0	11	Half	Addition	Sed	1.46
0	11	Half	Addition	Sed	7.49
0	11	Half	Addition	Sed	8.53
0	11	Half	Addition	Sed	11.99
0	11	Half	Addition	Sed	12.90
0	11	Half	Addition	Sed	2.96
0	11	Half	Addition	Sed	28.67
0	11	Half	Addition	Sed	8.14
0	11	Half	Addition	Sed	23.74
0	12	Half	Addition	Sed	7.62
0	12	Half	Addition	Sed	8.59
0	12	Half	Addition	Sed	24.02
0	12	Half	Addition	Sed	21.89
0	12	Half	Addition	Sed	15.46
0	12	Half	Addition	Sed	49.47
0	12	Half	Addition	Sed	19.70
0	12	Half	Addition	Sed	14.81
0	12	Half	Addition	Sed	16.31
0	12	Half	Addition	Sed	19.16
0	12	Half	Addition	Sed	16.02
0	12	Half	Addition	Sed	21.77
0	12	Half	Addition	Sed	11.38
0	12	Half	Addition	Sed	6.56
0	12	Half	Addition	Sed	21.30
0	12	Half	Addition	Sed	29.43
0	12	Half	Addition	Sed	15.72
0	12	Half	Addition	Sed	8.47
0	12	Half	Addition	Sed	9.30
0	12	Half	Addition	Sed	11.01
0	13	Open	Natural	Sed	2.60
0	13	Open	Natural	Sed	1.52
0	13	Open	Natural	Sed	2.03
0	13	Open	Natural	Sed	3.30
0	13	Open	Natural	Sed	1.76
0	13	Open	Natural	Sed	5.22
0	13	Open	Natural	Sed	3.06
0	13	Open	Natural	Sed	14.31
0	13	Open	Natural	Sed	6.18
0	13	Open	Natural	Sed	8.06
0	13	Open	Natural	Sed	1.51
0	13	Open	Natural	Sed	3.78
0	13	Open	Natural	Sed	8.61
0	13	Open	Natural	Sed	5.06
0	13	Open	Natural	Sed	4.20
0	13	Open	Natural	Sed	0.49
0	13	Open	Natural	Sed	4.45
0	13	Open	Natural	Sed	0.67
0	13	Open	Natural	Sed	3.01
0	13	Open	Natural	Sed	6.51
0	14	Open	Natural	Sed	0.00
0	14	Open	Natural	Sed	3.81
0	14	Open	Natural	Sed	1.10
0	14	Open	Natural	Sed	0.00

0	14 Open	Natural	Sed	3.15
0	14 Open	Natural	Sed	0.90
0	14 Open	Natural	Sed	4.47
0	14 Open	Natural	Sed	0.00
0	14 Open	Natural	Sed	1.79
0	14 Open	Natural	Sed	3.29
0	14 Open	Natural	Sed	1.58
0	14 Open	Natural	Sed	2.46
0	14 Open	Natural	Sed	2.48
0	14 Open	Natural	Sed	1.72
0	14 Open	Natural	Sed	2.54
0	14 Open	Natural	Sed	3.61
0	14 Open	Natural	Sed	6.19
0	14 Open	Natural	Sed	1.10
0	14 Open	Natural	Sed	2.51
0	14 Open	Natural	Sed	1.01
0	15 Open	Natural	Sed	1.51
0	15 Open	Natural	Sed	0.88
0	15 Open	Natural	Sed	0.73
0	15 Open	Natural	Sed	1.17
0	15 Open	Natural	Sed	1.73
0	15 Open	Natural	Sed	1.19
0	15 Open	Natural	Sed	1.04
0	15 Open	Natural	Sed	2.90
0	15 Open	Natural	Sed	1.53
0	15 Open	Natural	Sed	0.00
0	15 Open	Natural	Sed	0.00
0	15 Open	Natural	Sed	0.73
0	15 Open	Natural	Sed	3.05
0	15 Open	Natural	Sed	2.60
0	15 Open	Natural	Sed	1.10
0	15 Open	Natural	Sed	0.89
0	15 Open	Natural	Sed	1.63
0	15 Open	Natural	Sed	0.00
0	15 Open	Natural	Sed	1.18
0	15 Open	Natural	Sed	1.66
0	16 Open	Addition	Sed	29.93
0	16 Open	Addition	Sed	26.34
0	16 Open	Addition	Sed	25.01
0	16 Open	Addition	Sed	9.10
0	16 Open	Addition	Sed	12.11
0	16 Open	Addition	Sed	3.91
0	16 Open	Addition	Sed	2.12
0	16 Open	Addition	Sed	51.35
0	16 Open	Addition	Sed	50.88
0	16 Open	Addition	Sed	54.85
0	16 Open	Addition	Sed	24.09
0	16 Open	Addition	Sed	3.06
0	16 Open	Addition	Sed	2.90
0	16 Open	Addition	Sed	13.92
0	16 Open	Addition	Sed	18.08
0	16 Open	Addition	Sed	9.59
0	16 Open	Addition	Sed	11.56

0	16	Open	Addition	Sed	30.63
0	16	Open	Addition	Sed	31.92
0	16	Open	Addition	Sed	25.89
0	17	Open	Addition	Sed	13.34
0	17	Open	Addition	Sed	14.02
0	17	Open	Addition	Sed	4.31
0	17	Open	Addition	Sed	1.95
0	17	Open	Addition	Sed	4.92
0	17	Open	Addition	Sed	15.12
0	17	Open	Addition	Sed	7.43
0	17	Open	Addition	Sed	6.01
0	17	Open	Addition	Sed	8.90
0	17	Open	Addition	Sed	2.63
0	17	Open	Addition	Sed	4.99
0	17	Open	Addition	Sed	2.52
0	17	Open	Addition	Sed	30.81
0	17	Open	Addition	Sed	29.81
0	17	Open	Addition	Sed	17.19
0	17	Open	Addition	Sed	12.08
0	17	Open	Addition	Sed	62.12
0	17	Open	Addition	Sed	5.12
0	17	Open	Addition	Sed	5.55
0	17	Open	Addition	Sed	6.69
0	18	Open	Addition	Sed	29.46
0	18	Open	Addition	Sed	30.78
0	18	Open	Addition	Sed	26.58
0	18	Open	Addition	Sed	2.55
0	18	Open	Addition	Sed	8.96
0	18	Open	Addition	Sed	7.35
0	18	Open	Addition	Sed	3.17
0	18	Open	Addition	Sed	9.55
0	18	Open	Addition	Sed	8.57
0	18	Open	Addition	Sed	13.11
0	18	Open	Addition	Sed	11.22
0	18	Open	Addition	Sed	0.00
0	18	Open	Addition	Sed	3.30
0	18	Open	Addition	Sed	23.80
0	18	Open	Addition	Sed	14.51
0	18	Open	Addition	Sed	36.91
0	18	Open	Addition	Sed	4.52
0	18	Open	Addition	Sed	3.67
0	18	Open	Addition	Sed	12.11
0	18	Open	Addition	Sed	13.59
0	19	Full	Natural	Sed	4.38
0	19	Full	Natural	Sed	6.29
0	19	Full	Natural	Sed	6.79
0	19	Full	Natural	Sed	6.44
0	19	Full	Natural	Sed	3.30
0	19	Full	Natural	Sed	16.39
0	19	Full	Natural	Sed	4.86
0	19	Full	Natural	Sed	10.65
0	19	Full	Natural	Sed	9.09
0	19	Full	Natural	Sed	4.27

0	19 Full	Natural	Sed	5.22
0	19 Full	Natural	Sed	3.12
0	19 Full	Natural	Sed	8.71
0	19 Full	Natural	Sed	3.60
0	19 Full	Natural	Sed	5.51
0	19 Full	Natural	Sed	4.39
0	19 Full	Natural	Sed	3.06
0	19 Full	Natural	Sed	4.87
0	19 Full	Natural	Sed	6.39
0	19 Full	Natural	Sed	1.80
0	20 Full	Natural	Sed	2.54
0	20 Full	Natural	Sed	0.86
0	20 Full	Natural	Sed	2.35
0	20 Full	Natural	Sed	1.09
0	20 Full	Natural	Sed	6.73
0	20 Full	Natural	Sed	2.28
0	20 Full	Natural	Sed	4.09
0	20 Full	Natural	Sed	1.97
0	20 Full	Natural	Sed	1.33
0	20 Full	Natural	Sed	2.49
0	20 Full	Natural	Sed	4.08
0	20 Full	Natural	Sed	1.08
0	20 Full	Natural	Sed	0.00
0	20 Full	Natural	Sed	3.00
0	20 Full	Natural	Sed	0.00
0	20 Full	Natural	Sed	0.80
0	20 Full	Natural	Sed	2.42
0	20 Full	Natural	Sed	3.19
0	20 Full	Natural	Sed	4.88
0	20 Full	Natural	Sed	3.89
0	21 Full	Natural	Sed	5.01
0	21 Full	Natural	Sed	1.72
0	21 Full	Natural	Sed	1.90
0	21 Full	Natural	Sed	2.04
0	21 Full	Natural	Sed	1.78
0	21 Full	Natural	Sed	0.60
0	21 Full	Natural	Sed	1.75
0	21 Full	Natural	Sed	1.39
0	21 Full	Natural	Sed	1.44
0	21 Full	Natural	Sed	1.98
0	21 Full	Natural	Sed	2.12
0	21 Full	Natural	Sed	1.87
0	21 Full	Natural	Sed	1.96
0	21 Full	Natural	Sed	2.29
0	21 Full	Natural	Sed	2.40
0	21 Full	Natural	Sed	7.99
0	21 Full	Natural	Sed	2.20
0	21 Full	Natural	Sed	0.50
0	21 Full	Natural	Sed	3.06
0	21 Full	Natural	Sed	3.44
0	22 Full	Addition	Sed	7.94
0	22 Full	Addition	Sed	6.63
0	22 Full	Addition	Sed	19.03

0	22 Full	Addition	Sed	12.47
0	22 Full	Addition	Sed	12.26
0	22 Full	Addition	Sed	12.62
0	22 Full	Addition	Sed	7.54
0	22 Full	Addition	Sed	14.33
0	22 Full	Addition	Sed	8.13
0	22 Full	Addition	Sed	14.41
0	22 Full	Addition	Sed	17.24
0	22 Full	Addition	Sed	15.03
0	22 Full	Addition	Sed	8.32
0	22 Full	Addition	Sed	8.30
0	22 Full	Addition	Sed	5.51
0	22 Full	Addition	Sed	6.78
0	22 Full	Addition	Sed	7.16
0	22 Full	Addition	Sed	7.13
0	22 Full	Addition	Sed	3.71
0	22 Full	Addition	Sed	6.08
0	23 Full	Addition	Sed	4.53
0	23 Full	Addition	Sed	5.14
0	23 Full	Addition	Sed	5.52
0	23 Full	Addition	Sed	12.15
0	23 Full	Addition	Sed	6.57
0	23 Full	Addition	Sed	19.95
0	23 Full	Addition	Sed	5.62
0	23 Full	Addition	Sed	12.19
0	23 Full	Addition	Sed	6.29
0	23 Full	Addition	Sed	10.73
0	23 Full	Addition	Sed	41.71
0	23 Full	Addition	Sed	31.95
0	23 Full	Addition	Sed	19.76
0	23 Full	Addition	Sed	20.79
0	23 Full	Addition	Sed	23.86
0	23 Full	Addition	Sed	22.32
0	23 Full	Addition	Sed	11.12
0	23 Full	Addition	Sed	26.99
0	23 Full	Addition	Sed	31.17
0	23 Full	Addition	Sed	20.97
0	24 Full	Addition	Sed	6.52
0	24 Full	Addition	Sed	6.79
0	24 Full	Addition	Sed	15.20
0	24 Full	Addition	Sed	7.90
0	24 Full	Addition	Sed	3.84
0	24 Full	Addition	Sed	3.70
0	24 Full	Addition	Sed	6.73
0	24 Full	Addition	Sed	7.48
0	24 Full	Addition	Sed	8.06
0	24 Full	Addition	Sed	24.23
0	24 Full	Addition	Sed	20.50
0	24 Full	Addition	Sed	38.87
0	24 Full	Addition	Sed	20.37
0	24 Full	Addition	Sed	16.44
0	24 Full	Addition	Sed	20.97
0	24 Full	Addition	Sed	31.45

0	24	Full	Addition	Sed	19.53
0	24	Full	Addition	Sed	9.93
0	24	Full	Addition	Sed	4.60
0	24	Full	Addition	Sed	6.57
0	25	Half	Natural	Sed	3.48
0	25	Half	Natural	Sed	2.35
0	25	Half	Natural	Sed	7.75
0	25	Half	Natural	Sed	3.28
0	25	Half	Natural	Sed	1.13
0	25	Half	Natural	Sed	6.13
0	25	Half	Natural	Sed	4.70
0	25	Half	Natural	Sed	2.17
0	25	Half	Natural	Sed	8.01
0	25	Half	Natural	Sed	3.32
0	25	Half	Natural	Sed	4.35
0	25	Half	Natural	Sed	2.28
0	25	Half	Natural	Sed	1.97
0	25	Half	Natural	Sed	1.34
0	25	Half	Natural	Sed	2.45
0	25	Half	Natural	Sed	2.86
0	25	Half	Natural	Sed	4.32
0	25	Half	Natural	Sed	3.71
0	25	Half	Natural	Sed	2.31
0	25	Half	Natural	Sed	2.68
0	26	Half	Natural	Sed	1.03
0	26	Half	Natural	Sed	3.22
0	26	Half	Natural	Sed	2.11
0	26	Half	Natural	Sed	2.17
0	26	Half	Natural	Sed	3.58
0	26	Half	Natural	Sed	2.35
0	26	Half	Natural	Sed	2.47
0	26	Half	Natural	Sed	1.43
0	26	Half	Natural	Sed	1.92
0	26	Half	Natural	Sed	1.65
0	26	Half	Natural	Sed	3.08
0	26	Half	Natural	Sed	1.21
0	26	Half	Natural	Sed	1.21
0	26	Half	Natural	Sed	2.39
0	26	Half	Natural	Sed	1.69
0	26	Half	Natural	Sed	3.95
0	26	Half	Natural	Sed	1.45
0	26	Half	Natural	Sed	2.89
0	26	Half	Natural	Sed	3.62
0	26	Half	Natural	Sed	2.07
0	27	Half	Natural	Sed	3.53
0	27	Half	Natural	Sed	2.50
0	27	Half	Natural	Sed	1.19
0	27	Half	Natural	Sed	2.45
0	27	Half	Natural	Sed	4.28
0	27	Half	Natural	Sed	1.21
0	27	Half	Natural	Sed	2.31
0	27	Half	Natural	Sed	2.88
0	27	Half	Natural	Sed	1.80

0	27	Half	Natural	Sed	2.45
0	27	Half	Natural	Sed	1.95
0	27	Half	Natural	Sed	2.17
0	27	Half	Natural	Sed	4.96
0	27	Half	Natural	Sed	1.16
0	27	Half	Natural	Sed	2.28
0	27	Half	Natural	Sed	1.68
0	27	Half	Natural	Sed	1.80
0	27	Half	Natural	Sed	3.04
0	27	Half	Natural	Sed	0.00
0	27	Half	Natural	Sed	1.72
0	28	Half	Addition	Sed	21.32
0	28	Half	Addition	Sed	18.24
0	28	Half	Addition	Sed	4.86
0	28	Half	Addition	Sed	2.57
0	28	Half	Addition	Sed	12.56
0	28	Half	Addition	Sed	7.34
0	28	Half	Addition	Sed	12.01
0	28	Half	Addition	Sed	17.37
0	28	Half	Addition	Sed	2.72
0	28	Half	Addition	Sed	8.96
0	28	Half	Addition	Sed	9.10
0	28	Half	Addition	Sed	2.72
0	28	Half	Addition	Sed	6.07
0	28	Half	Addition	Sed	14.85
0	28	Half	Addition	Sed	6.68
0	28	Half	Addition	Sed	7.07
0	28	Half	Addition	Sed	14.14
0	28	Half	Addition	Sed	16.34
0	28	Half	Addition	Sed	4.59
0	28	Half	Addition	Sed	10.35
0	29	Half	Addition	Sed	14.54
0	29	Half	Addition	Sed	16.93
0	29	Half	Addition	Sed	24.24
0	29	Half	Addition	Sed	12.13
0	29	Half	Addition	Sed	6.67
0	29	Half	Addition	Sed	5.30
0	29	Half	Addition	Sed	5.12
0	29	Half	Addition	Sed	24.52
0	29	Half	Addition	Sed	25.43
0	29	Half	Addition	Sed	13.14
0	29	Half	Addition	Sed	10.81
0	29	Half	Addition	Sed	27.77
0	29	Half	Addition	Sed	15.47
0	29	Half	Addition	Sed	16.03
0	29	Half	Addition	Sed	12.97
0	29	Half	Addition	Sed	11.03
0	29	Half	Addition	Sed	13.31
0	29	Half	Addition	Sed	17.63
0	29	Half	Addition	Sed	10.17
0	29	Half	Addition	Sed	10.26
0	30	Half	Addition	Sed	14.17
0	30	Half	Addition	Sed	16.00

0	30	Half	Addition	Sed	3.70
0	30	Half	Addition	Sed	8.31
0	30	Half	Addition	Sed	3.14
0	30	Half	Addition	Sed	12.60
0	30	Half	Addition	Sed	24.02
0	30	Half	Addition	Sed	24.87
0	30	Half	Addition	Sed	20.55
0	30	Half	Addition	Sed	15.94
0	30	Half	Addition	Sed	12.88
0	30	Half	Addition	Sed	19.40
0	30	Half	Addition	Sed	17.61
0	30	Half	Addition	Sed	19.27
0	30	Half	Addition	Sed	30.80
0	30	Half	Addition	Sed	21.92
0	30	Half	Addition	Sed	28.44
0	30	Half	Addition	Sed	53.54
0	30	Half	Addition	Sed	44.98
0	30	Half	Addition	Sed	26.08
0	31	Open	Natural	Sed	1.62
0	31	Open	Natural	Sed	0.00
0	31	Open	Natural	Sed	2.38
0	31	Open	Natural	Sed	1.27
0	31	Open	Natural	Sed	1.65
0	31	Open	Natural	Sed	1.67
0	31	Open	Natural	Sed	1.97
0	31	Open	Natural	Sed	1.29
0	31	Open	Natural	Sed	2.09
0	31	Open	Natural	Sed	3.25
0	31	Open	Natural	Sed	1.33
0	31	Open	Natural	Sed	1.62
0	31	Open	Natural	Sed	1.31
0	31	Open	Natural	Sed	2.16
0	31	Open	Natural	Sed	1.72
0	31	Open	Natural	Sed	2.35
0	31	Open	Natural	Sed	2.14
0	31	Open	Natural	Sed	1.84
0	31	Open	Natural	Sed	2.93
0	31	Open	Natural	Sed	2.43
0	32	Open	Natural	Sed	1.78
0	32	Open	Natural	Sed	1.20
0	32	Open	Natural	Sed	3.51
0	32	Open	Natural	Sed	2.87
0	32	Open	Natural	Sed	1.84
0	32	Open	Natural	Sed	3.38
0	32	Open	Natural	Sed	2.25
0	32	Open	Natural	Sed	6.10
0	32	Open	Natural	Sed	2.21
0	32	Open	Natural	Sed	3.88
0	32	Open	Natural	Sed	3.52
0	32	Open	Natural	Sed	1.88
0	32	Open	Natural	Sed	2.00
0	32	Open	Natural	Sed	1.43
0	32	Open	Natural	Sed	0.00

0	32 Open	Natural	Sed	3.54
0	32 Open	Natural	Sed	2.43
0	32 Open	Natural	Sed	2.10
0	32 Open	Natural	Sed	1.67
0	32 Open	Natural	Sed	3.75
0	33 Open	Natural	Sed	1.56
0	33 Open	Natural	Sed	1.65
0	33 Open	Natural	Sed	0.00
0	33 Open	Natural	Sed	2.02
0	33 Open	Natural	Sed	1.02
0	33 Open	Natural	Sed	1.37
0	33 Open	Natural	Sed	1.26
0	33 Open	Natural	Sed	2.06
0	33 Open	Natural	Sed	0.70
0	33 Open	Natural	Sed	2.06
0	33 Open	Natural	Sed	2.66
0	33 Open	Natural	Sed	1.11
0	33 Open	Natural	Sed	2.56
0	33 Open	Natural	Sed	2.08
0	33 Open	Natural	Sed	1.18
0	33 Open	Natural	Sed	3.66
0	33 Open	Natural	Sed	1.77
0	33 Open	Natural	Sed	0.93
0	33 Open	Natural	Sed	2.65
0	33 Open	Natural	Sed	0.95
0	34 Open	Addition	Sed	36.96
0	34 Open	Addition	Sed	21.95
0	34 Open	Addition	Sed	7.94
0	34 Open	Addition	Sed	13.51
0	34 Open	Addition	Sed	18.38
0	34 Open	Addition	Sed	16.51
0	34 Open	Addition	Sed	16.73
0	34 Open	Addition	Sed	15.98
0	34 Open	Addition	Sed	4.74
0	34 Open	Addition	Sed	4.20
0	34 Open	Addition	Sed	12.52
0	34 Open	Addition	Sed	8.26
0	34 Open	Addition	Sed	6.76
0	34 Open	Addition	Sed	12.27
0	34 Open	Addition	Sed	13.53
0	34 Open	Addition	Sed	8.15
0	34 Open	Addition	Sed	9.50
0	34 Open	Addition	Sed	9.75
0	34 Open	Addition	Sed	12.02
0	34 Open	Addition	Sed	11.96
0	35 Open	Addition	Sed	11.56
0	35 Open	Addition	Sed	14.81
0	35 Open	Addition	Sed	16.52
0	35 Open	Addition	Sed	10.42
0	35 Open	Addition	Sed	13.41
0	35 Open	Addition	Sed	18.15
0	35 Open	Addition	Sed	35.77
0	35 Open	Addition	Sed	25.60

0	35	Open	Addition	Sed	8.73
0	35	Open	Addition	Sed	8.07
0	35	Open	Addition	Sed	21.58
0	35	Open	Addition	Sed	23.50
0	35	Open	Addition	Sed	15.84
0	35	Open	Addition	Sed	11.51
0	35	Open	Addition	Sed	10.10
0	35	Open	Addition	Sed	17.69
0	35	Open	Addition	Sed	15.41
0	35	Open	Addition	Sed	11.65
0	35	Open	Addition	Sed	18.48
0	35	Open	Addition	Sed	22.83
0	36	Open	Addition	Sed	19.01
0	36	Open	Addition	Sed	25.40
0	36	Open	Addition	Sed	11.30
0	36	Open	Addition	Sed	18.51
0	36	Open	Addition	Sed	24.80
0	36	Open	Addition	Sed	9.59
0	36	Open	Addition	Sed	13.88
0	36	Open	Addition	Sed	17.59
0	36	Open	Addition	Sed	17.78
0	36	Open	Addition	Sed	11.83
0	36	Open	Addition	Sed	6.57
0	36	Open	Addition	Sed	20.01
0	36	Open	Addition	Sed	20.16
0	36	Open	Addition	Sed	32.14
0	36	Open	Addition	Sed	9.19
0	36	Open	Addition	Sed	8.72
0	36	Open	Addition	Sed	19.68
0	36	Open	Addition	Sed	20.80
0	36	Open	Addition	Sed	13.11
0	36	Open	Addition	Sed	16.07
1	1	Full	Natural	Sed	3.00
1	1	Full	Natural	Sed	3.99
1	1	Full	Natural	Sed	2.81
1	1	Full	Natural	Sed	2.53
1	1	Full	Natural	Sed	4.86
1	1	Full	Natural	Sed	1.27
1	1	Full	Natural	Sed	2.62
1	1	Full	Natural	Sed	2.12
1	1	Full	Natural	Sed	3.36
1	1	Full	Natural	Sed	3.46
1	1	Full	Natural	Sed	2.30
1	1	Full	Natural	Sed	1.38
1	1	Full	Natural	Sed	10.92
1	1	Full	Natural	Sed	3.64
1	1	Full	Natural	Sed	9.16
1	1	Full	Natural	Sed	1.78
1	1	Full	Natural	Sed	2.58
1	1	Full	Natural	Sed	2.65
1	1	Full	Natural	Sed	3.16
1	1	Full	Natural	Sed	1.91
1	2	Full	Natural	Sed	2.83

1	2	Full	Natural	Sed	2.84
1	2	Full	Natural	Sed	2.13
1	2	Full	Natural	Sed	3.78
1	2	Full	Natural	Sed	3.04
1	2	Full	Natural	Sed	6.88
1	2	Full	Natural	Sed	4.31
1	2	Full	Natural	Sed	5.44
1	2	Full	Natural	Sed	1.88
1	2	Full	Natural	Sed	3.34
1	2	Full	Natural	Sed	3.77
1	2	Full	Natural	Sed	3.80
1	2	Full	Natural	Sed	2.95
1	2	Full	Natural	Sed	2.40
1	2	Full	Natural	Sed	3.18
1	2	Full	Natural	Sed	2.10
1	2	Full	Natural	Sed	3.68
1	2	Full	Natural	Sed	1.00
1	2	Full	Natural	Sed	3.25
1	2	Full	Natural	Sed	2.09
1	3	Full	Natural	Sed	3.19
1	3	Full	Natural	Sed	1.65
1	3	Full	Natural	Sed	4.50
1	3	Full	Natural	Sed	6.54
1	3	Full	Natural	Sed	1.91
1	3	Full	Natural	Sed	3.03
1	3	Full	Natural	Sed	4.28
1	3	Full	Natural	Sed	2.09
1	3	Full	Natural	Sed	1.34
1	3	Full	Natural	Sed	4.50
1	3	Full	Natural	Sed	3.30
1	3	Full	Natural	Sed	1.93
1	3	Full	Natural	Sed	2.88
1	3	Full	Natural	Sed	6.39
1	3	Full	Natural	Sed	1.68
1	3	Full	Natural	Sed	0.00
1	3	Full	Natural	Sed	3.70
1	3	Full	Natural	Sed	3.12
1	3	Full	Natural	Sed	1.73
1	3	Full	Natural	Sed	2.28
1	4	Full	Addition	Sed	13.30
1	4	Full	Addition	Sed	16.63
1	4	Full	Addition	Sed	6.47
1	4	Full	Addition	Sed	7.90
1	4	Full	Addition	Sed	4.20
1	4	Full	Addition	Sed	5.77
1	4	Full	Addition	Sed	6.49
1	4	Full	Addition	Sed	18.20
1	4	Full	Addition	Sed	5.77
1	4	Full	Addition	Sed	14.25
1	4	Full	Addition	Sed	4.20
1	4	Full	Addition	Sed	14.90
1	4	Full	Addition	Sed	11.17
1	4	Full	Addition	Sed	8.69

1	4	Full	Addition	Sed	9.96
1	4	Full	Addition	Sed	10.25
1	4	Full	Addition	Sed	11.28
1	4	Full	Addition	Sed	9.38
1	4	Full	Addition	Sed	27.74
1	4	Full	Addition	Sed	29.06
1	5	Full	Addition	Sed	8.05
1	5	Full	Addition	Sed	8.02
1	5	Full	Addition	Sed	13.25
1	5	Full	Addition	Sed	12.89
1	5	Full	Addition	Sed	8.44
1	5	Full	Addition	Sed	8.05
1	5	Full	Addition	Sed	29.89
1	5	Full	Addition	Sed	26.56
1	5	Full	Addition	Sed	15.51
1	5	Full	Addition	Sed	3.99
1	5	Full	Addition	Sed	6.95
1	5	Full	Addition	Sed	6.04
1	5	Full	Addition	Sed	7.67
1	5	Full	Addition	Sed	8.71
1	5	Full	Addition	Sed	8.48
1	5	Full	Addition	Sed	13.16
1	5	Full	Addition	Sed	9.42
1	5	Full	Addition	Sed	9.66
1	5	Full	Addition	Sed	30.90
1	5	Full	Addition	Sed	4.27
1	6	Full	Addition	Sed	9.24
1	6	Full	Addition	Sed	9.25
1	6	Full	Addition	Sed	16.34
1	6	Full	Addition	Sed	24.92
1	6	Full	Addition	Sed	5.26
1	6	Full	Addition	Sed	4.70
1	6	Full	Addition	Sed	10.39
1	6	Full	Addition	Sed	8.77
1	6	Full	Addition	Sed	6.63
1	6	Full	Addition	Sed	7.66
1	6	Full	Addition	Sed	8.61
1	6	Full	Addition	Sed	4.76
1	6	Full	Addition	Sed	8.20
1	6	Full	Addition	Sed	3.79
1	6	Full	Addition	Sed	5.02
1	6	Full	Addition	Sed	7.58
1	6	Full	Addition	Sed	0.00
1	6	Full	Addition	Sed	1.73
1	6	Full	Addition	Sed	18.10
1	6	Full	Addition	Sed	8.65
1	7	Half	Natural	Sed	1.87
1	7	Half	Natural	Sed	5.20
1	7	Half	Natural	Sed	5.07
1	7	Half	Natural	Sed	6.75
1	7	Half	Natural	Sed	0.98
1	7	Half	Natural	Sed	2.06
1	7	Half	Natural	Sed	2.45

1	7	Half	Natural	Sed	4.14
1	7	Half	Natural	Sed	3.67
1	7	Half	Natural	Sed	2.35
1	7	Half	Natural	Sed	3.44
1	7	Half	Natural	Sed	2.18
1	7	Half	Natural	Sed	3.87
1	7	Half	Natural	Sed	2.62
1	7	Half	Natural	Sed	0.51
1	7	Half	Natural	Sed	3.19
1	7	Half	Natural	Sed	4.50
1	7	Half	Natural	Sed	1.89
1	7	Half	Natural	Sed	4.64
1	7	Half	Natural	Sed	3.62
1	8	Half	Natural	Sed	2.37
1	8	Half	Natural	Sed	2.18
1	8	Half	Natural	Sed	0.00
1	8	Half	Natural	Sed	2.14
1	8	Half	Natural	Sed	4.51
1	8	Half	Natural	Sed	6.26
1	8	Half	Natural	Sed	12.56
1	8	Half	Natural	Sed	2.89
1	8	Half	Natural	Sed	2.38
1	8	Half	Natural	Sed	2.12
1	8	Half	Natural	Sed	3.13
1	8	Half	Natural	Sed	0.85
1	8	Half	Natural	Sed	4.11
1	8	Half	Natural	Sed	2.71
1	8	Half	Natural	Sed	1.92
1	8	Half	Natural	Sed	3.21
1	8	Half	Natural	Sed	1.70
1	8	Half	Natural	Sed	1.76
1	8	Half	Natural	Sed	1.74
1	8	Half	Natural	Sed	3.54
1	9	Half	Natural	Sed	6.67
1	9	Half	Natural	Sed	2.55
1	9	Half	Natural	Sed	0.95
1	9	Half	Natural	Sed	1.71
1	9	Half	Natural	Sed	5.98
1	9	Half	Natural	Sed	2.04
1	9	Half	Natural	Sed	10.09
1	9	Half	Natural	Sed	2.77
1	9	Half	Natural	Sed	3.09
1	9	Half	Natural	Sed	1.31
1	9	Half	Natural	Sed	2.61
1	9	Half	Natural	Sed	2.37
1	9	Half	Natural	Sed	0.00
1	9	Half	Natural	Sed	1.19
1	9	Half	Natural	Sed	2.11
1	9	Half	Natural	Sed	2.45
1	9	Half	Natural	Sed	1.92
1	9	Half	Natural	Sed	2.11
1	9	Half	Natural	Sed	2.33
1	9	Half	Natural	Sed	3.06

1	10	Half	Addition	Sed	4.72
1	10	Half	Addition	Sed	3.55
1	10	Half	Addition	Sed	16.71
1	10	Half	Addition	Sed	14.95
1	10	Half	Addition	Sed	7.42
1	10	Half	Addition	Sed	6.00
1	10	Half	Addition	Sed	6.37
1	10	Half	Addition	Sed	13.83
1	10	Half	Addition	Sed	6.48
1	10	Half	Addition	Sed	7.99
1	10	Half	Addition	Sed	4.98
1	10	Half	Addition	Sed	6.66
1	10	Half	Addition	Sed	9.17
1	10	Half	Addition	Sed	4.65
1	10	Half	Addition	Sed	7.43
1	10	Half	Addition	Sed	7.55
1	10	Half	Addition	Sed	11.78
1	10	Half	Addition	Sed	7.02
1	10	Half	Addition	Sed	6.71
1	10	Half	Addition	Sed	29.52
1	11	Half	Addition	Sed	10.16
1	11	Half	Addition	Sed	16.51
1	11	Half	Addition	Sed	7.89
1	11	Half	Addition	Sed	12.70
1	11	Half	Addition	Sed	3.98
1	11	Half	Addition	Sed	18.08
1	11	Half	Addition	Sed	5.91
1	11	Half	Addition	Sed	18.31
1	11	Half	Addition	Sed	10.21
1	11	Half	Addition	Sed	37.53
1	11	Half	Addition	Sed	18.72
1	11	Half	Addition	Sed	10.64
1	11	Half	Addition	Sed	16.34
1	11	Half	Addition	Sed	4.21
1	11	Half	Addition	Sed	5.18
1	11	Half	Addition	Sed	9.71
1	11	Half	Addition	Sed	10.25
1	11	Half	Addition	Sed	5.03
1	11	Half	Addition	Sed	9.06
1	11	Half	Addition	Sed	11.00
1	12	Half	Addition	Sed	8.42
1	12	Half	Addition	Sed	5.09
1	12	Half	Addition	Sed	7.52
1	12	Half	Addition	Sed	15.63
1	12	Half	Addition	Sed	5.09
1	12	Half	Addition	Sed	10.91
1	12	Half	Addition	Sed	1.93
1	12	Half	Addition	Sed	28.96
1	12	Half	Addition	Sed	14.04
1	12	Half	Addition	Sed	15.28
1	12	Half	Addition	Sed	10.38
1	12	Half	Addition	Sed	13.75
1	12	Half	Addition	Sed	15.12

1	12	Half	Addition	Sed	12.45
1	12	Half	Addition	Sed	9.52
1	12	Half	Addition	Sed	26.56
1	12	Half	Addition	Sed	2.91
1	12	Half	Addition	Sed	5.04
1	12	Half	Addition	Sed	12.53
1	12	Half	Addition	Sed	6.08
1	13	Open	Natural	Sed	2.78
1	13	Open	Natural	Sed	1.39
1	13	Open	Natural	Sed	2.45
1	13	Open	Natural	Sed	1.43
1	13	Open	Natural	Sed	1.76
1	13	Open	Natural	Sed	1.42
1	13	Open	Natural	Sed	1.99
1	13	Open	Natural	Sed	1.48
1	13	Open	Natural	Sed	1.86
1	13	Open	Natural	Sed	6.68
1	13	Open	Natural	Sed	9.57
1	13	Open	Natural	Sed	1.70
1	13	Open	Natural	Sed	1.68
1	13	Open	Natural	Sed	2.02
1	13	Open	Natural	Sed	2.74
1	13	Open	Natural	Sed	1.53
1	13	Open	Natural	Sed	3.30
1	13	Open	Natural	Sed	1.39
1	13	Open	Natural	Sed	3.18
1	13	Open	Natural	Sed	0.91
1	14	Open	Natural	Sed	2.02
1	14	Open	Natural	Sed	0.51
1	14	Open	Natural	Sed	2.53
1	14	Open	Natural	Sed	2.18
1	14	Open	Natural	Sed	0.78
1	14	Open	Natural	Sed	2.58
1	14	Open	Natural	Sed	1.46
1	14	Open	Natural	Sed	2.91
1	14	Open	Natural	Sed	2.19
1	14	Open	Natural	Sed	0.89
1	14	Open	Natural	Sed	2.05
1	14	Open	Natural	Sed	0.50
1	14	Open	Natural	Sed	4.11
1	14	Open	Natural	Sed	3.84
1	14	Open	Natural	Sed	1.70
1	14	Open	Natural	Sed	4.36
1	14	Open	Natural	Sed	3.16
1	14	Open	Natural	Sed	2.60
1	14	Open	Natural	Sed	1.93
1	14	Open	Natural	Sed	0.00
1	15	Open	Natural	Sed	1.30
1	15	Open	Natural	Sed	0.83
1	15	Open	Natural	Sed	2.12
1	15	Open	Natural	Sed	1.52
1	15	Open	Natural	Sed	1.54
1	15	Open	Natural	Sed	1.20

1	15 Open	Natural	Sed	1.28
1	15 Open	Natural	Sed	1.41
1	15 Open	Natural	Sed	1.50
1	15 Open	Natural	Sed	3.52
1	15 Open	Natural	Sed	1.35
1	15 Open	Natural	Sed	1.26
1	15 Open	Natural	Sed	0.93
1	15 Open	Natural	Sed	2.08
1	15 Open	Natural	Sed	1.12
1	15 Open	Natural	Sed	0.00
1	15 Open	Natural	Sed	1.98
1	15 Open	Natural	Sed	1.73
1	15 Open	Natural	Sed	1.81
1	15 Open	Natural	Sed	1.19
1	16 Open	Addition	Sed	20.22
1	16 Open	Addition	Sed	5.03
1	16 Open	Addition	Sed	4.93
1	16 Open	Addition	Sed	5.47
1	16 Open	Addition	Sed	3.31
1	16 Open	Addition	Sed	2.80
1	16 Open	Addition	Sed	4.06
1	16 Open	Addition	Sed	9.92
1	16 Open	Addition	Sed	9.67
1	16 Open	Addition	Sed	12.09
1	16 Open	Addition	Sed	10.43
1	16 Open	Addition	Sed	16.24
1	16 Open	Addition	Sed	4.03
1	16 Open	Addition	Sed	10.60
1	16 Open	Addition	Sed	8.02
1	16 Open	Addition	Sed	5.34
1	16 Open	Addition	Sed	6.34
1	16 Open	Addition	Sed	3.20
1	16 Open	Addition	Sed	4.34
1	16 Open	Addition	Sed	5.61
1	17 Open	Addition	Sed	7.57
1	17 Open	Addition	Sed	9.81
1	17 Open	Addition	Sed	6.58
1	17 Open	Addition	Sed	4.23
1	17 Open	Addition	Sed	9.34
1	17 Open	Addition	Sed	5.72
1	17 Open	Addition	Sed	8.18
1	17 Open	Addition	Sed	5.47
1	17 Open	Addition	Sed	4.88
1	17 Open	Addition	Sed	20.81
1	17 Open	Addition	Sed	2.17
1	17 Open	Addition	Sed	5.86
1	17 Open	Addition	Sed	8.06
1	17 Open	Addition	Sed	6.88
1	17 Open	Addition	Sed	9.87
1	17 Open	Addition	Sed	4.68
1	17 Open	Addition	Sed	4.35
1	17 Open	Addition	Sed	10.55
1	17 Open	Addition	Sed	21.68

1	17	Open	Addition	Sed	24.65
1	18	Open	Addition	Sed	12.40
1	18	Open	Addition	Sed	4.16
1	18	Open	Addition	Sed	2.68
1	18	Open	Addition	Sed	16.94
1	18	Open	Addition	Sed	15.41
1	18	Open	Addition	Sed	4.28
1	18	Open	Addition	Sed	7.21
1	18	Open	Addition	Sed	11.92
1	18	Open	Addition	Sed	18.36
1	18	Open	Addition	Sed	6.46
1	18	Open	Addition	Sed	11.28
1	18	Open	Addition	Sed	7.13
1	18	Open	Addition	Sed	4.77
1	18	Open	Addition	Sed	8.99
1	18	Open	Addition	Sed	13.58
1	18	Open	Addition	Sed	4.04
1	18	Open	Addition	Sed	11.40
1	18	Open	Addition	Sed	6.68
1	18	Open	Addition	Sed	25.80
1	18	Open	Addition	Sed	24.21
1	19	Cage	Natural	Sed	3.68
1	19	Cage	Natural	Sed	1.71
1	19	Cage	Natural	Sed	1.82
1	19	Cage	Natural	Sed	1.91
1	19	Cage	Natural	Sed	2.26
1	19	Cage	Natural	Sed	10.23
1	19	Cage	Natural	Sed	4.36
1	19	Cage	Natural	Sed	2.96
1	19	Cage	Natural	Sed	2.72
1	19	Cage	Natural	Sed	2.54
1	19	Cage	Natural	Sed	12.96
1	19	Cage	Natural	Sed	6.25
1	19	Cage	Natural	Sed	3.67
1	19	Cage	Natural	Sed	2.41
1	19	Cage	Natural	Sed	6.37
1	19	Cage	Natural	Sed	0.25
1	19	Cage	Natural	Sed	1.95
1	19	Cage	Natural	Sed	4.43
1	19	Cage	Natural	Sed	3.27
1	19	Cage	Natural	Sed	3.46
1	20	Cage	Natural	Sed	4.29
1	20	Cage	Natural	Sed	5.48
1	20	Cage	Natural	Sed	2.26
1	20	Cage	Natural	Sed	1.86
1	20	Cage	Natural	Sed	0.95
1	20	Cage	Natural	Sed	3.04
1	20	Cage	Natural	Sed	2.29
1	20	Cage	Natural	Sed	1.95
1	20	Cage	Natural	Sed	2.93
1	20	Cage	Natural	Sed	0.00
1	20	Cage	Natural	Sed	2.82
1	20	Cage	Natural	Sed	2.39

1	20	Cage	Natural	Sed	1.74
1	20	Cage	Natural	Sed	0.98
1	20	Cage	Natural	Sed	1.60
1	20	Cage	Natural	Sed	4.47
1	20	Cage	Natural	Sed	2.12
1	20	Cage	Natural	Sed	2.74
1	20	Cage	Natural	Sed	2.27
1	20	Cage	Natural	Sed	2.58
1	21	Cage	Natural	Sed	6.04
1	21	Cage	Natural	Sed	4.22
1	21	Cage	Natural	Sed	2.26
1	21	Cage	Natural	Sed	3.61
1	21	Cage	Natural	Sed	2.29
1	21	Cage	Natural	Sed	3.58
1	21	Cage	Natural	Sed	6.03
1	21	Cage	Natural	Sed	11.56
1	21	Cage	Natural	Sed	1.83
1	21	Cage	Natural	Sed	2.39
1	21	Cage	Natural	Sed	12.56
1	21	Cage	Natural	Sed	1.80
1	21	Cage	Natural	Sed	3.01
1	21	Cage	Natural	Sed	1.18
1	21	Cage	Natural	Sed	2.38
1	21	Cage	Natural	Sed	1.46
1	21	Cage	Natural	Sed	2.14
1	21	Cage	Natural	Sed	1.79
1	21	Cage	Natural	Sed	3.07
1	21	Cage	Natural	Sed	2.53
1	22	Cage	Addition	Sed	5.28
1	22	Cage	Addition	Sed	4.28
1	22	Cage	Addition	Sed	21.47
1	22	Cage	Addition	Sed	18.05
1	22	Cage	Addition	Sed	20.25
1	22	Cage	Addition	Sed	10.97
1	22	Cage	Addition	Sed	2.63
1	22	Cage	Addition	Sed	8.10
1	22	Cage	Addition	Sed	6.98
1	22	Cage	Addition	Sed	17.54
1	22	Cage	Addition	Sed	7.78
1	22	Cage	Addition	Sed	11.37
1	22	Cage	Addition	Sed	12.79
1	22	Cage	Addition	Sed	7.91
1	22	Cage	Addition	Sed	27.38
1	22	Cage	Addition	Sed	9.03
1	22	Cage	Addition	Sed	3.65
1	22	Cage	Addition	Sed	20.10
1	22	Cage	Addition	Sed	30.74
1	22	Cage	Addition	Sed	26.35
1	23	Cage	Addition	Sed	14.10
1	23	Cage	Addition	Sed	8.13
1	23	Cage	Addition	Sed	8.45
1	23	Cage	Addition	Sed	6.84
1	23	Cage	Addition	Sed	8.43

1	23	Cage	Addition	Sed	9.12
1	23	Cage	Addition	Sed	9.79
1	23	Cage	Addition	Sed	7.87
1	23	Cage	Addition	Sed	2.86
1	23	Cage	Addition	Sed	19.35
1	23	Cage	Addition	Sed	16.23
1	23	Cage	Addition	Sed	15.64
1	23	Cage	Addition	Sed	37.03
1	23	Cage	Addition	Sed	43.83
1	23	Cage	Addition	Sed	29.57
1	23	Cage	Addition	Sed	32.23
1	23	Cage	Addition	Sed	6.09
1	23	Cage	Addition	Sed	8.74
1	23	Cage	Addition	Sed	3.83
1	23	Cage	Addition	Sed	8.58
1	24	Cage	Addition	Sed	7.48
1	24	Cage	Addition	Sed	3.09
1	24	Cage	Addition	Sed	13.92
1	24	Cage	Addition	Sed	9.60
1	24	Cage	Addition	Sed	7.93
1	24	Cage	Addition	Sed	5.74
1	24	Cage	Addition	Sed	12.69
1	24	Cage	Addition	Sed	11.42
1	24	Cage	Addition	Sed	2.28
1	24	Cage	Addition	Sed	6.37
1	24	Cage	Addition	Sed	7.51
1	24	Cage	Addition	Sed	17.47
1	24	Cage	Addition	Sed	11.73
1	24	Cage	Addition	Sed	6.15
1	24	Cage	Addition	Sed	19.84
1	24	Cage	Addition	Sed	7.28
1	24	Cage	Addition	Sed	20.07
1	24	Cage	Addition	Sed	4.28
1	24	Cage	Addition	Sed	3.23
1	24	Cage	Addition	Sed	6.11
1	25	Half	Natural	Sed	7.41
1	25	Half	Natural	Sed	1.83
1	25	Half	Natural	Sed	2.01
1	25	Half	Natural	Sed	2.43
1	25	Half	Natural	Sed	2.98
1	25	Half	Natural	Sed	3.95
1	25	Half	Natural	Sed	3.67
1	25	Half	Natural	Sed	3.84
1	25	Half	Natural	Sed	0.00
1	25	Half	Natural	Sed	1.40
1	25	Half	Natural	Sed	5.85
1	25	Half	Natural	Sed	2.06
1	25	Half	Natural	Sed	2.57
1	25	Half	Natural	Sed	1.76
1	25	Half	Natural	Sed	5.42
1	25	Half	Natural	Sed	5.62
1	25	Half	Natural	Sed	1.87
1	25	Half	Natural	Sed	0.64

1	25	Half	Natural	Sed	1.61
1	25	Half	Natural	Sed	5.72
1	26	Half	Natural	Sed	1.22
1	26	Half	Natural	Sed	1.46
1	26	Half	Natural	Sed	1.47
1	26	Half	Natural	Sed	1.30
1	26	Half	Natural	Sed	1.82
1	26	Half	Natural	Sed	1.41
1	26	Half	Natural	Sed	6.08
1	26	Half	Natural	Sed	1.82
1	26	Half	Natural	Sed	1.92
1	26	Half	Natural	Sed	0.00
1	26	Half	Natural	Sed	1.60
1	26	Half	Natural	Sed	2.04
1	26	Half	Natural	Sed	1.76
1	26	Half	Natural	Sed	3.18
1	26	Half	Natural	Sed	3.53
1	26	Half	Natural	Sed	3.54
1	26	Half	Natural	Sed	1.73
1	26	Half	Natural	Sed	1.91
1	26	Half	Natural	Sed	1.30
1	26	Half	Natural	Sed	1.65
1	27	Half	Natural	Sed	2.54
1	27	Half	Natural	Sed	3.22
1	27	Half	Natural	Sed	2.21
1	27	Half	Natural	Sed	2.52
1	27	Half	Natural	Sed	1.99
1	27	Half	Natural	Sed	3.70
1	27	Half	Natural	Sed	2.26
1	27	Half	Natural	Sed	2.23
1	27	Half	Natural	Sed	2.20
1	27	Half	Natural	Sed	2.92
1	27	Half	Natural	Sed	2.20
1	27	Half	Natural	Sed	2.19
1	27	Half	Natural	Sed	2.09
1	27	Half	Natural	Sed	2.03
1	27	Half	Natural	Sed	2.44
1	27	Half	Natural	Sed	2.76
1	27	Half	Natural	Sed	3.92
1	27	Half	Natural	Sed	1.59
1	27	Half	Natural	Sed	2.00
1	27	Half	Natural	Sed	1.73
1	28	Half	Addition	Sed	19.44
1	28	Half	Addition	Sed	7.00
1	28	Half	Addition	Sed	10.62
1	28	Half	Addition	Sed	5.23
1	28	Half	Addition	Sed	4.43
1	28	Half	Addition	Sed	18.30
1	28	Half	Addition	Sed	10.38
1	28	Half	Addition	Sed	7.98
1	28	Half	Addition	Sed	3.19
1	28	Half	Addition	Sed	5.42
1	28	Half	Addition	Sed	7.40

1	28	Half	Addition	Sed	10.27
1	28	Half	Addition	Sed	10.18
1	28	Half	Addition	Sed	7.86
1	28	Half	Addition	Sed	14.26
1	28	Half	Addition	Sed	7.45
1	28	Half	Addition	Sed	3.93
1	28	Half	Addition	Sed	6.16
1	28	Half	Addition	Sed	13.67
1	28	Half	Addition	Sed	20.11
1	29	Half	Addition	Sed	8.83
1	29	Half	Addition	Sed	14.93
1	29	Half	Addition	Sed	21.86
1	29	Half	Addition	Sed	3.09
1	29	Half	Addition	Sed	20.71
1	29	Half	Addition	Sed	20.00
1	29	Half	Addition	Sed	11.45
1	29	Half	Addition	Sed	9.35
1	29	Half	Addition	Sed	3.84
1	29	Half	Addition	Sed	20.27
1	29	Half	Addition	Sed	6.69
1	29	Half	Addition	Sed	6.48
1	29	Half	Addition	Sed	8.66
1	29	Half	Addition	Sed	3.37
1	29	Half	Addition	Sed	0.00
1	29	Half	Addition	Sed	10.58
1	29	Half	Addition	Sed	5.61
1	29	Half	Addition	Sed	9.19
1	29	Half	Addition	Sed	10.36
1	29	Half	Addition	Sed	24.87
1	30	Half	Addition	Sed	6.89
1	30	Half	Addition	Sed	6.38
1	30	Half	Addition	Sed	15.40
1	30	Half	Addition	Sed	11.50
1	30	Half	Addition	Sed	5.75
1	30	Half	Addition	Sed	19.39
1	30	Half	Addition	Sed	14.36
1	30	Half	Addition	Sed	14.56
1	30	Half	Addition	Sed	2.81
1	30	Half	Addition	Sed	8.03
1	30	Half	Addition	Sed	9.97
1	30	Half	Addition	Sed	13.66
1	30	Half	Addition	Sed	18.90
1	30	Half	Addition	Sed	9.38
1	30	Half	Addition	Sed	3.31
1	30	Half	Addition	Sed	6.55
1	30	Half	Addition	Sed	7.64
1	30	Half	Addition	Sed	18.13
1	30	Half	Addition	Sed	4.91
1	30	Half	Addition	Sed	7.73
1	31	Open	Natural	Sed	0.95
1	31	Open	Natural	Sed	1.62
1	31	Open	Natural	Sed	1.86
1	31	Open	Natural	Sed	1.31

1	31	Open	Natural	Sed	4.03
1	31	Open	Natural	Sed	0.00
1	31	Open	Natural	Sed	1.79
1	31	Open	Natural	Sed	1.91
1	31	Open	Natural	Sed	2.94
1	31	Open	Natural	Sed	3.03
1	31	Open	Natural	Sed	1.47
1	31	Open	Natural	Sed	3.75
1	31	Open	Natural	Sed	1.44
1	31	Open	Natural	Sed	3.01
1	31	Open	Natural	Sed	2.00
1	31	Open	Natural	Sed	0.00
1	31	Open	Natural	Sed	1.33
1	31	Open	Natural	Sed	2.22
1	31	Open	Natural	Sed	2.51
1	31	Open	Natural	Sed	0.94
1	32	Open	Natural	Sed	1.88
1	32	Open	Natural	Sed	2.48
1	32	Open	Natural	Sed	1.27
1	32	Open	Natural	Sed	2.63
1	32	Open	Natural	Sed	2.78
1	32	Open	Natural	Sed	0.86
1	32	Open	Natural	Sed	0.00
1	32	Open	Natural	Sed	3.06
1	32	Open	Natural	Sed	1.16
1	32	Open	Natural	Sed	1.73
1	32	Open	Natural	Sed	2.25
1	32	Open	Natural	Sed	3.94
1	32	Open	Natural	Sed	2.23
1	32	Open	Natural	Sed	1.86
1	32	Open	Natural	Sed	2.59
1	32	Open	Natural	Sed	2.10
1	32	Open	Natural	Sed	2.46
1	32	Open	Natural	Sed	2.59
1	32	Open	Natural	Sed	2.11
1	32	Open	Natural	Sed	2.29
1	33	Open	Natural	Sed	2.88
1	33	Open	Natural	Sed	1.65
1	33	Open	Natural	Sed	0.00
1	33	Open	Natural	Sed	1.42
1	33	Open	Natural	Sed	1.54
1	33	Open	Natural	Sed	1.27
1	33	Open	Natural	Sed	1.39
1	33	Open	Natural	Sed	1.29
1	33	Open	Natural	Sed	1.67
1	33	Open	Natural	Sed	2.36
1	33	Open	Natural	Sed	1.78
1	33	Open	Natural	Sed	1.85
1	33	Open	Natural	Sed	2.06
1	33	Open	Natural	Sed	1.50
1	33	Open	Natural	Sed	1.90
1	33	Open	Natural	Sed	1.60
1	33	Open	Natural	Sed	2.23

1	33	Open	Natural	Sed	2.51
1	33	Open	Natural	Sed	1.56
1	33	Open	Natural	Sed	2.16
1	34	Open	Addition	Sed	5.44
1	34	Open	Addition	Sed	10.80
1	34	Open	Addition	Sed	9.23
1	34	Open	Addition	Sed	16.24
1	34	Open	Addition	Sed	7.03
1	34	Open	Addition	Sed	11.88
1	34	Open	Addition	Sed	8.14
1	34	Open	Addition	Sed	13.59
1	34	Open	Addition	Sed	4.98
1	34	Open	Addition	Sed	10.89
1	34	Open	Addition	Sed	8.74
1	34	Open	Addition	Sed	11.40
1	34	Open	Addition	Sed	13.52
1	34	Open	Addition	Sed	18.46
1	34	Open	Addition	Sed	13.43
1	34	Open	Addition	Sed	8.87
1	34	Open	Addition	Sed	5.72
1	34	Open	Addition	Sed	13.95
1	34	Open	Addition	Sed	22.64
1	34	Open	Addition	Sed	27.65
1	35	Open	Addition	Sed	9.27
1	35	Open	Addition	Sed	16.25
1	35	Open	Addition	Sed	11.91
1	35	Open	Addition	Sed	9.74
1	35	Open	Addition	Sed	14.33
1	35	Open	Addition	Sed	4.37
1	35	Open	Addition	Sed	14.92
1	35	Open	Addition	Sed	17.61
1	35	Open	Addition	Sed	6.28
1	35	Open	Addition	Sed	13.44
1	35	Open	Addition	Sed	9.33
1	35	Open	Addition	Sed	13.89
1	35	Open	Addition	Sed	24.74
1	35	Open	Addition	Sed	11.31
1	35	Open	Addition	Sed	12.58
1	35	Open	Addition	Sed	15.54
1	35	Open	Addition	Sed	11.23
1	35	Open	Addition	Sed	14.55
1	35	Open	Addition	Sed	27.73
1	35	Open	Addition	Sed	20.96
1	36	Open	Addition	Sed	10.09
1	36	Open	Addition	Sed	10.96
1	36	Open	Addition	Sed	10.03
1	36	Open	Addition	Sed	15.67
1	36	Open	Addition	Sed	9.99
1	36	Open	Addition	Sed	10.69
1	36	Open	Addition	Sed	12.74
1	36	Open	Addition	Sed	3.66
1	36	Open	Addition	Sed	6.24
1	36	Open	Addition	Sed	8.05

1	36	Open	Addition	Sed	14.23
1	36	Open	Addition	Sed	8.08
1	36	Open	Addition	Sed	10.08
1	36	Open	Addition	Sed	12.91
1	36	Open	Addition	Sed	14.87
1	36	Open	Addition	Sed	20.51
1	36	Open	Addition	Sed	15.32
1	36	Open	Addition	Sed	25.86
1	36	Open	Addition	Sed	24.33
1	36	Open	Addition	Sed	22.84
2	1	Full	Natural	Sed	1.86
2	1	Full	Natural	Sed	3.06
2	1	Full	Natural	Sed	1.77
2	1	Full	Natural	Sed	13.68
2	1	Full	Natural	Sed	2.22
2	1	Full	Natural	Sed	3.16
2	1	Full	Natural	Sed	2.45
2	1	Full	Natural	Sed	3.08
2	1	Full	Natural	Sed	8.86
2	1	Full	Natural	Sed	3.08
2	1	Full	Natural	Sed	3.87
2	1	Full	Natural	Sed	3.38
2	1	Full	Natural	Sed	3.18
2	1	Full	Natural	Sed	3.46
2	1	Full	Natural	Sed	3.12
2	1	Full	Natural	Sed	3.16
2	1	Full	Natural	Sed	3.93
2	1	Full	Natural	Sed	2.43
2	1	Full	Natural	Sed	2.67
2	1	Full	Natural	Sed	3.01
2	2	Full	Natural	Sed	1.96
2	2	Full	Natural	Sed	4.64
2	2	Full	Natural	Sed	2.99
2	2	Full	Natural	Sed	5.17
2	2	Full	Natural	Sed	4.13
2	2	Full	Natural	Sed	5.26
2	2	Full	Natural	Sed	1.81
2	2	Full	Natural	Sed	5.17
2	2	Full	Natural	Sed	5.42
2	2	Full	Natural	Sed	1.10
2	2	Full	Natural	Sed	2.30
2	2	Full	Natural	Sed	4.62
2	2	Full	Natural	Sed	3.28
2	2	Full	Natural	Sed	2.55
2	2	Full	Natural	Sed	2.02
2	2	Full	Natural	Sed	1.79
2	2	Full	Natural	Sed	3.48
2	2	Full	Natural	Sed	4.41
2	2	Full	Natural	Sed	6.17
2	2	Full	Natural	Sed	3.49
2	3	Full	Natural	Sed	3.09
2	3	Full	Natural	Sed	3.03
2	3	Full	Natural	Sed	2.62

2	3 Full	Natural	Sed	5.79
2	3 Full	Natural	Sed	3.67
2	3 Full	Natural	Sed	4.41
2	3 Full	Natural	Sed	2.52
2	3 Full	Natural	Sed	2.42
2	3 Full	Natural	Sed	3.90
2	3 Full	Natural	Sed	0.00
2	3 Full	Natural	Sed	2.09
2	3 Full	Natural	Sed	3.59
2	3 Full	Natural	Sed	3.07
2	3 Full	Natural	Sed	2.24
2	3 Full	Natural	Sed	2.79
2	3 Full	Natural	Sed	2.44
2	3 Full	Natural	Sed	1.84
2	3 Full	Natural	Sed	2.07
2	3 Full	Natural	Sed	3.27
2	3 Full	Natural	Sed	6.18
2	4 Full	Addition	Sed	4.42
2	4 Full	Addition	Sed	12.71
2	4 Full	Addition	Sed	2.21
2	4 Full	Addition	Sed	2.35
2	4 Full	Addition	Sed	5.23
2	4 Full	Addition	Sed	10.30
2	4 Full	Addition	Sed	3.78
2	4 Full	Addition	Sed	5.35
2	4 Full	Addition	Sed	6.32
2	4 Full	Addition	Sed	5.74
2	4 Full	Addition	Sed	10.05
2	4 Full	Addition	Sed	3.01
2	4 Full	Addition	Sed	4.53
2	4 Full	Addition	Sed	1.83
2	4 Full	Addition	Sed	3.27
2	4 Full	Addition	Sed	2.12
2	4 Full	Addition	Sed	3.06
2	4 Full	Addition	Sed	3.71
2	4 Full	Addition	Sed	5.22
2	4 Full	Addition	Sed	3.79
2	5 Full	Addition	Sed	4.48
2	5 Full	Addition	Sed	2.40
2	5 Full	Addition	Sed	9.34
2	5 Full	Addition	Sed	1.88
2	5 Full	Addition	Sed	2.19
2	5 Full	Addition	Sed	1.37
2	5 Full	Addition	Sed	1.67
2	5 Full	Addition	Sed	1.52
2	5 Full	Addition	Sed	2.05
2	5 Full	Addition	Sed	2.75
2	5 Full	Addition	Sed	2.06
2	5 Full	Addition	Sed	1.87
2	5 Full	Addition	Sed	1.80
2	5 Full	Addition	Sed	1.73
2	5 Full	Addition	Sed	1.42
2	5 Full	Addition	Sed	2.68

2	5	Full	Addition	Sed	2.01
2	5	Full	Addition	Sed	1.12
2	5	Full	Addition	Sed	1.90
2	5	Full	Addition	Sed	1.24
2	6	Full	Addition	Sed	4.28
2	6	Full	Addition	Sed	5.86
2	6	Full	Addition	Sed	0.00
2	6	Full	Addition	Sed	6.44
2	6	Full	Addition	Sed	2.61
2	6	Full	Addition	Sed	7.18
2	6	Full	Addition	Sed	5.02
2	6	Full	Addition	Sed	2.96
2	6	Full	Addition	Sed	1.53
2	6	Full	Addition	Sed	2.71
2	6	Full	Addition	Sed	2.38
2	6	Full	Addition	Sed	3.22
2	6	Full	Addition	Sed	1.80
2	6	Full	Addition	Sed	3.38
2	6	Full	Addition	Sed	1.99
2	6	Full	Addition	Sed	1.37
2	6	Full	Addition	Sed	2.09
2	6	Full	Addition	Sed	1.58
2	6	Full	Addition	Sed	1.49
2	6	Full	Addition	Sed	1.92
2	7	Half	Natural	Sed	3.38
2	7	Half	Natural	Sed	3.66
2	7	Half	Natural	Sed	3.69
2	7	Half	Natural	Sed	6.50
2	7	Half	Natural	Sed	2.47
2	7	Half	Natural	Sed	1.93
2	7	Half	Natural	Sed	2.70
2	7	Half	Natural	Sed	2.91
2	7	Half	Natural	Sed	1.09
2	7	Half	Natural	Sed	2.40
2	7	Half	Natural	Sed	3.52
2	7	Half	Natural	Sed	1.83
2	7	Half	Natural	Sed	8.72
2	7	Half	Natural	Sed	6.79
2	7	Half	Natural	Sed	2.42
2	7	Half	Natural	Sed	1.76
2	7	Half	Natural	Sed	2.34
2	7	Half	Natural	Sed	2.97
2	7	Half	Natural	Sed	1.45
2	7	Half	Natural	Sed	2.18
2	8	Half	Natural	Sed	2.81
2	8	Half	Natural	Sed	0.00
2	8	Half	Natural	Sed	1.72
2	8	Half	Natural	Sed	2.65
2	8	Half	Natural	Sed	1.92
2	8	Half	Natural	Sed	5.53
2	8	Half	Natural	Sed	1.67
2	8	Half	Natural	Sed	3.09
2	8	Half	Natural	Sed	1.21

2	8	Half	Natural	Sed	2.64
2	8	Half	Natural	Sed	2.35
2	8	Half	Natural	Sed	1.23
2	8	Half	Natural	Sed	2.41
2	8	Half	Natural	Sed	1.82
2	8	Half	Natural	Sed	1.98
2	8	Half	Natural	Sed	2.37
2	8	Half	Natural	Sed	3.14
2	8	Half	Natural	Sed	2.22
2	8	Half	Natural	Sed	2.44
2	8	Half	Natural	Sed	2.04
2	9	Half	Natural	Sed	9.67
2	9	Half	Natural	Sed	4.28
2	9	Half	Natural	Sed	1.99
2	9	Half	Natural	Sed	2.46
2	9	Half	Natural	Sed	2.45
2	9	Half	Natural	Sed	3.76
2	9	Half	Natural	Sed	4.15
2	9	Half	Natural	Sed	2.79
2	9	Half	Natural	Sed	3.32
2	9	Half	Natural	Sed	2.81
2	9	Half	Natural	Sed	4.90
2	9	Half	Natural	Sed	2.15
2	9	Half	Natural	Sed	3.18
2	9	Half	Natural	Sed	1.40
2	9	Half	Natural	Sed	6.99
2	9	Half	Natural	Sed	2.14
2	9	Half	Natural	Sed	2.34
2	9	Half	Natural	Sed	2.07
2	9	Half	Natural	Sed	4.08
2	9	Half	Natural	Sed	3.42
2	10	Half	Addition	Sed	1.48
2	10	Half	Addition	Sed	1.53
2	10	Half	Addition	Sed	2.60
2	10	Half	Addition	Sed	3.02
2	10	Half	Addition	Sed	7.17
2	10	Half	Addition	Sed	5.48
2	10	Half	Addition	Sed	3.29
2	10	Half	Addition	Sed	1.63
2	10	Half	Addition	Sed	2.84
2	10	Half	Addition	Sed	3.27
2	10	Half	Addition	Sed	1.62
2	10	Half	Addition	Sed	6.03
2	10	Half	Addition	Sed	1.37
2	10	Half	Addition	Sed	2.52
2	10	Half	Addition	Sed	4.17
2	10	Half	Addition	Sed	1.90
2	10	Half	Addition	Sed	3.20
2	10	Half	Addition	Sed	2.22
2	10	Half	Addition	Sed	2.30
2	10	Half	Addition	Sed	2.15
2	11	Half	Addition	Sed	2.76
2	11	Half	Addition	Sed	5.72

2	11	Half	Addition	Sed	2.94
2	11	Half	Addition	Sed	2.57
2	11	Half	Addition	Sed	3.07
2	11	Half	Addition	Sed	4.44
2	11	Half	Addition	Sed	4.05
2	11	Half	Addition	Sed	1.41
2	11	Half	Addition	Sed	3.07
2	11	Half	Addition	Sed	9.66
2	11	Half	Addition	Sed	4.48
2	11	Half	Addition	Sed	2.46
2	11	Half	Addition	Sed	7.18
2	11	Half	Addition	Sed	3.38
2	11	Half	Addition	Sed	3.75
2	11	Half	Addition	Sed	1.79
2	11	Half	Addition	Sed	3.17
2	11	Half	Addition	Sed	2.63
2	11	Half	Addition	Sed	2.52
2	11	Half	Addition	Sed	3.84
2	12	Half	Addition	Sed	1.45
2	12	Half	Addition	Sed	1.61
2	12	Half	Addition	Sed	2.08
2	12	Half	Addition	Sed	4.51
2	12	Half	Addition	Sed	1.44
2	12	Half	Addition	Sed	1.90
2	12	Half	Addition	Sed	11.27
2	12	Half	Addition	Sed	3.46
2	12	Half	Addition	Sed	2.03
2	12	Half	Addition	Sed	4.58
2	12	Half	Addition	Sed	2.21
2	12	Half	Addition	Sed	3.47
2	12	Half	Addition	Sed	1.26
2	12	Half	Addition	Sed	0.99
2	12	Half	Addition	Sed	3.03
2	12	Half	Addition	Sed	3.29
2	12	Half	Addition	Sed	1.28
2	12	Half	Addition	Sed	4.99
2	12	Half	Addition	Sed	2.66
2	12	Half	Addition	Sed	14.00
2	13	Open	Natural	Sed	2.67
2	13	Open	Natural	Sed	4.53
2	13	Open	Natural	Sed	6.71
2	13	Open	Natural	Sed	2.13
2	13	Open	Natural	Sed	3.30
2	13	Open	Natural	Sed	2.81
2	13	Open	Natural	Sed	2.28
2	13	Open	Natural	Sed	4.04
2	13	Open	Natural	Sed	3.65
2	13	Open	Natural	Sed	4.46
2	13	Open	Natural	Sed	11.88
2	13	Open	Natural	Sed	10.59
2	13	Open	Natural	Sed	4.36
2	13	Open	Natural	Sed	2.36
2	13	Open	Natural	Sed	2.94

2	13	Open	Natural	Sed	2.54
2	13	Open	Natural	Sed	2.00
2	13	Open	Natural	Sed	1.80
2	13	Open	Natural	Sed	3.28
2	13	Open	Natural	Sed	3.57
2	14	Open	Natural	Sed	11.65
2	14	Open	Natural	Sed	0.00
2	14	Open	Natural	Sed	2.61
2	14	Open	Natural	Sed	3.20
2	14	Open	Natural	Sed	8.06
2	14	Open	Natural	Sed	1.96
2	14	Open	Natural	Sed	2.08
2	14	Open	Natural	Sed	3.91
2	14	Open	Natural	Sed	2.05
2	14	Open	Natural	Sed	3.50
2	14	Open	Natural	Sed	1.91
2	14	Open	Natural	Sed	2.75
2	14	Open	Natural	Sed	2.41
2	14	Open	Natural	Sed	1.41
2	14	Open	Natural	Sed	2.11
2	14	Open	Natural	Sed	2.33
2	14	Open	Natural	Sed	2.78
2	14	Open	Natural	Sed	1.44
2	14	Open	Natural	Sed	3.64
2	14	Open	Natural	Sed	1.30
2	15	Open	Natural	Sed	1.69
2	15	Open	Natural	Sed	2.54
2	15	Open	Natural	Sed	2.40
2	15	Open	Natural	Sed	2.50
2	15	Open	Natural	Sed	2.34
2	15	Open	Natural	Sed	1.02
2	15	Open	Natural	Sed	0.98
2	15	Open	Natural	Sed	0.00
2	15	Open	Natural	Sed	2.86
2	15	Open	Natural	Sed	3.20
2	15	Open	Natural	Sed	3.11
2	15	Open	Natural	Sed	2.07
2	15	Open	Natural	Sed	3.29
2	15	Open	Natural	Sed	4.19
2	15	Open	Natural	Sed	2.31
2	15	Open	Natural	Sed	2.04
2	15	Open	Natural	Sed	2.77
2	15	Open	Natural	Sed	3.02
2	15	Open	Natural	Sed	2.00
2	15	Open	Natural	Sed	2.32
2	16	Open	Addition	Sed	4.06
2	16	Open	Addition	Sed	1.65
2	16	Open	Addition	Sed	3.94
2	16	Open	Addition	Sed	2.52
2	16	Open	Addition	Sed	2.07
2	16	Open	Addition	Sed	1.19
2	16	Open	Addition	Sed	2.03
2	16	Open	Addition	Sed	3.51

2	16	Open	Addition	Sed	2.32
2	16	Open	Addition	Sed	1.89
2	16	Open	Addition	Sed	2.45
2	16	Open	Addition	Sed	1.45
2	16	Open	Addition	Sed	1.60
2	16	Open	Addition	Sed	3.60
2	16	Open	Addition	Sed	3.66
2	16	Open	Addition	Sed	1.41
2	16	Open	Addition	Sed	2.12
2	16	Open	Addition	Sed	1.98
2	16	Open	Addition	Sed	2.18
2	16	Open	Addition	Sed	1.69
2	17	Open	Addition	Sed	8.78
2	17	Open	Addition	Sed	3.56
2	17	Open	Addition	Sed	2.42
2	17	Open	Addition	Sed	6.49
2	17	Open	Addition	Sed	1.80
2	17	Open	Addition	Sed	7.45
2	17	Open	Addition	Sed	3.10
2	17	Open	Addition	Sed	2.95
2	17	Open	Addition	Sed	5.29
2	17	Open	Addition	Sed	3.15
2	17	Open	Addition	Sed	1.73
2	17	Open	Addition	Sed	6.93
2	17	Open	Addition	Sed	5.15
2	17	Open	Addition	Sed	3.56
2	17	Open	Addition	Sed	3.87
2	17	Open	Addition	Sed	2.79
2	17	Open	Addition	Sed	2.37
2	17	Open	Addition	Sed	2.29
2	17	Open	Addition	Sed	3.56
2	17	Open	Addition	Sed	2.52
2	18	Open	Addition	Sed	1.75
2	18	Open	Addition	Sed	2.52
2	18	Open	Addition	Sed	3.98
2	18	Open	Addition	Sed	2.76
2	18	Open	Addition	Sed	3.07
2	18	Open	Addition	Sed	2.57
2	18	Open	Addition	Sed	1.72
2	18	Open	Addition	Sed	2.42
2	18	Open	Addition	Sed	3.42
2	18	Open	Addition	Sed	2.04
2	18	Open	Addition	Sed	2.18
2	18	Open	Addition	Sed	2.02
2	18	Open	Addition	Sed	1.72
2	18	Open	Addition	Sed	2.58
2	18	Open	Addition	Sed	1.21
2	18	Open	Addition	Sed	2.87
2	18	Open	Addition	Sed	2.05
2	18	Open	Addition	Sed	3.05
2	18	Open	Addition	Sed	3.64
2	18	Open	Addition	Sed	6.58
2	19	Cage	Natural	Sed	5.95

2	19	Cage	Natural	Sed	3.02
2	19	Cage	Natural	Sed	4.93
2	19	Cage	Natural	Sed	12.63
2	19	Cage	Natural	Sed	5.40
2	19	Cage	Natural	Sed	13.18
2	19	Cage	Natural	Sed	3.94
2	19	Cage	Natural	Sed	2.51
2	19	Cage	Natural	Sed	6.80
2	19	Cage	Natural	Sed	13.73
2	19	Cage	Natural	Sed	3.45
2	19	Cage	Natural	Sed	6.06
2	19	Cage	Natural	Sed	6.50
2	19	Cage	Natural	Sed	3.01
2	19	Cage	Natural	Sed	4.29
2	19	Cage	Natural	Sed	2.78
2	19	Cage	Natural	Sed	1.97
2	19	Cage	Natural	Sed	2.39
2	19	Cage	Natural	Sed	1.33
2	19	Cage	Natural	Sed	13.68
2	20	Cage	Natural	Sed	2.14
2	20	Cage	Natural	Sed	2.64
2	20	Cage	Natural	Sed	1.94
2	20	Cage	Natural	Sed	3.35
2	20	Cage	Natural	Sed	1.97
2	20	Cage	Natural	Sed	8.88
2	20	Cage	Natural	Sed	2.06
2	20	Cage	Natural	Sed	3.30
2	20	Cage	Natural	Sed	3.63
2	20	Cage	Natural	Sed	2.54
2	20	Cage	Natural	Sed	2.03
2	20	Cage	Natural	Sed	4.45
2	20	Cage	Natural	Sed	2.00
2	20	Cage	Natural	Sed	4.66
2	20	Cage	Natural	Sed	1.78
2	20	Cage	Natural	Sed	2.86
2	20	Cage	Natural	Sed	1.41
2	20	Cage	Natural	Sed	0.00
2	20	Cage	Natural	Sed	3.38
2	20	Cage	Natural	Sed	1.70
2	21	Cage	Natural	Sed	4.40
2	21	Cage	Natural	Sed	2.22
2	21	Cage	Natural	Sed	2.55
2	21	Cage	Natural	Sed	2.86
2	21	Cage	Natural	Sed	3.98
2	21	Cage	Natural	Sed	2.34
2	21	Cage	Natural	Sed	4.92
2	21	Cage	Natural	Sed	3.31
2	21	Cage	Natural	Sed	4.28
2	21	Cage	Natural	Sed	1.78
2	21	Cage	Natural	Sed	2.85
2	21	Cage	Natural	Sed	2.13
2	21	Cage	Natural	Sed	1.69
2	21	Cage	Natural	Sed	2.50

2	21	Cage	Natural	Sed	2.45
2	21	Cage	Natural	Sed	1.97
2	21	Cage	Natural	Sed	5.94
2	21	Cage	Natural	Sed	2.74
2	21	Cage	Natural	Sed	3.12
2	21	Cage	Natural	Sed	3.48
2	22	Cage	Addition	Sed	3.29
2	22	Cage	Addition	Sed	17.99
2	22	Cage	Addition	Sed	5.58
2	22	Cage	Addition	Sed	6.16
2	22	Cage	Addition	Sed	15.47
2	22	Cage	Addition	Sed	13.28
2	22	Cage	Addition	Sed	3.26
2	22	Cage	Addition	Sed	9.61
2	22	Cage	Addition	Sed	8.53
2	22	Cage	Addition	Sed	21.03
2	22	Cage	Addition	Sed	6.60
2	22	Cage	Addition	Sed	7.05
2	22	Cage	Addition	Sed	4.34
2	22	Cage	Addition	Sed	5.56
2	22	Cage	Addition	Sed	7.46
2	22	Cage	Addition	Sed	4.99
2	22	Cage	Addition	Sed	29.09
2	22	Cage	Addition	Sed	33.86
2	22	Cage	Addition	Sed	37.01
2	22	Cage	Addition	Sed	6.21
2	23	Cage	Addition	Sed	19.79
2	23	Cage	Addition	Sed	7.29
2	23	Cage	Addition	Sed	8.62
2	23	Cage	Addition	Sed	5.58
2	23	Cage	Addition	Sed	19.00
2	23	Cage	Addition	Sed	13.95
2	23	Cage	Addition	Sed	3.74
2	23	Cage	Addition	Sed	16.95
2	23	Cage	Addition	Sed	22.46
2	23	Cage	Addition	Sed	8.23
2	23	Cage	Addition	Sed	2.86
2	23	Cage	Addition	Sed	13.45
2	23	Cage	Addition	Sed	3.60
2	23	Cage	Addition	Sed	16.49
2	23	Cage	Addition	Sed	4.05
2	23	Cage	Addition	Sed	6.23
2	23	Cage	Addition	Sed	9.43
2	23	Cage	Addition	Sed	70.15
2	23	Cage	Addition	Sed	69.58
2	23	Cage	Addition	Sed	25.98
2	24	Cage	Addition	Sed	4.62
2	24	Cage	Addition	Sed	5.52
2	24	Cage	Addition	Sed	4.90
2	24	Cage	Addition	Sed	8.65
2	24	Cage	Addition	Sed	4.98
2	24	Cage	Addition	Sed	8.70
2	24	Cage	Addition	Sed	14.94

2	24	Cage	Addition	Sed	10.48
2	24	Cage	Addition	Sed	7.48
2	24	Cage	Addition	Sed	16.36
2	24	Cage	Addition	Sed	6.01
2	24	Cage	Addition	Sed	6.31
2	24	Cage	Addition	Sed	4.27
2	24	Cage	Addition	Sed	4.34
2	24	Cage	Addition	Sed	2.48
2	24	Cage	Addition	Sed	4.72
2	24	Cage	Addition	Sed	5.90
2	24	Cage	Addition	Sed	29.72
2	24	Cage	Addition	Sed	34.30
2	24	Cage	Addition	Sed	27.78
2	25	Half	Natural	Sed	1.52
2	25	Half	Natural	Sed	3.87
2	25	Half	Natural	Sed	1.53
2	25	Half	Natural	Sed	1.63
2	25	Half	Natural	Sed	1.61
2	25	Half	Natural	Sed	1.68
2	25	Half	Natural	Sed	3.50
2	25	Half	Natural	Sed	5.64
2	25	Half	Natural	Sed	2.50
2	25	Half	Natural	Sed	1.13
2	25	Half	Natural	Sed	1.62
2	25	Half	Natural	Sed	3.63
2	25	Half	Natural	Sed	2.75
2	25	Half	Natural	Sed	8.26
2	25	Half	Natural	Sed	1.23
2	25	Half	Natural	Sed	3.00
2	25	Half	Natural	Sed	3.94
2	25	Half	Natural	Sed	3.43
2	25	Half	Natural	Sed	4.02
2	25	Half	Natural	Sed	1.53
2	26	Half	Natural	Sed	1.44
2	26	Half	Natural	Sed	0.90
2	26	Half	Natural	Sed	1.71
2	26	Half	Natural	Sed	1.80
2	26	Half	Natural	Sed	1.33
2	26	Half	Natural	Sed	0.00
2	26	Half	Natural	Sed	2.76
2	26	Half	Natural	Sed	1.14
2	26	Half	Natural	Sed	1.69
2	26	Half	Natural	Sed	1.80
2	26	Half	Natural	Sed	1.73
2	26	Half	Natural	Sed	1.75
2	26	Half	Natural	Sed	1.51
2	26	Half	Natural	Sed	2.60
2	26	Half	Natural	Sed	2.05
2	26	Half	Natural	Sed	2.39
2	26	Half	Natural	Sed	4.00
2	26	Half	Natural	Sed	4.66
2	26	Half	Natural	Sed	1.92
2	26	Half	Natural	Sed	0.96

2	27	Half	Natural	Sed	0.89
2	27	Half	Natural	Sed	1.60
2	27	Half	Natural	Sed	2.97
2	27	Half	Natural	Sed	1.75
2	27	Half	Natural	Sed	2.14
2	27	Half	Natural	Sed	2.14
2	27	Half	Natural	Sed	1.55
2	27	Half	Natural	Sed	1.61
2	27	Half	Natural	Sed	1.72
2	27	Half	Natural	Sed	3.01
2	27	Half	Natural	Sed	2.29
2	27	Half	Natural	Sed	1.35
2	27	Half	Natural	Sed	1.51
2	27	Half	Natural	Sed	1.33
2	27	Half	Natural	Sed	1.82
2	27	Half	Natural	Sed	2.92
2	27	Half	Natural	Sed	2.22
2	27	Half	Natural	Sed	1.81
2	27	Half	Natural	Sed	2.28
2	27	Half	Natural	Sed	1.56
2	28	Half	Addition	Sed	11.92
2	28	Half	Addition	Sed	4.08
2	28	Half	Addition	Sed	9.78
2	28	Half	Addition	Sed	8.41
2	28	Half	Addition	Sed	9.68
2	28	Half	Addition	Sed	4.06
2	28	Half	Addition	Sed	14.34
2	28	Half	Addition	Sed	9.66
2	28	Half	Addition	Sed	2.48
2	28	Half	Addition	Sed	6.72
2	28	Half	Addition	Sed	19.89
2	28	Half	Addition	Sed	8.25
2	28	Half	Addition	Sed	4.69
2	28	Half	Addition	Sed	11.70
2	28	Half	Addition	Sed	8.49
2	28	Half	Addition	Sed	6.91
2	28	Half	Addition	Sed	7.45
2	28	Half	Addition	Sed	18.37
2	28	Half	Addition	Sed	7.07
2	28	Half	Addition	Sed	22.67
2	29	Half	Addition	Sed	21.09
2	29	Half	Addition	Sed	4.25
2	29	Half	Addition	Sed	11.22
2	29	Half	Addition	Sed	17.33
2	29	Half	Addition	Sed	6.70
2	29	Half	Addition	Sed	17.31
2	29	Half	Addition	Sed	6.40
2	29	Half	Addition	Sed	13.55
2	29	Half	Addition	Sed	19.36
2	29	Half	Addition	Sed	4.27
2	29	Half	Addition	Sed	7.50
2	29	Half	Addition	Sed	8.65
2	29	Half	Addition	Sed	11.61

2	29	Half	Addition	Sed	5.40
2	29	Half	Addition	Sed	4.18
2	29	Half	Addition	Sed	11.42
2	29	Half	Addition	Sed	8.26
2	29	Half	Addition	Sed	6.97
2	29	Half	Addition	Sed	3.05
2	29	Half	Addition	Sed	55.36
2	30	Half	Addition	Sed	8.37
2	30	Half	Addition	Sed	5.59
2	30	Half	Addition	Sed	2.86
2	30	Half	Addition	Sed	9.31
2	30	Half	Addition	Sed	4.69
2	30	Half	Addition	Sed	7.79
2	30	Half	Addition	Sed	10.16
2	30	Half	Addition	Sed	7.72
2	30	Half	Addition	Sed	1.57
2	30	Half	Addition	Sed	6.40
2	30	Half	Addition	Sed	3.92
2	30	Half	Addition	Sed	2.24
2	30	Half	Addition	Sed	6.47
2	30	Half	Addition	Sed	21.69
2	30	Half	Addition	Sed	22.09
2	30	Half	Addition	Sed	17.51
2	30	Half	Addition	Sed	23.25
2	30	Half	Addition	Sed	11.50
2	30	Half	Addition	Sed	14.60
2	30	Half	Addition	Sed	19.49
2	31	Open	Natural	Sed	2.78
2	31	Open	Natural	Sed	2.78
2	31	Open	Natural	Sed	1.12
2	31	Open	Natural	Sed	2.23
2	31	Open	Natural	Sed	1.72
2	31	Open	Natural	Sed	1.85
2	31	Open	Natural	Sed	2.20
2	31	Open	Natural	Sed	1.80
2	31	Open	Natural	Sed	3.09
2	31	Open	Natural	Sed	3.12
2	31	Open	Natural	Sed	2.67
2	31	Open	Natural	Sed	1.17
2	31	Open	Natural	Sed	2.78
2	31	Open	Natural	Sed	2.68
2	31	Open	Natural	Sed	1.81
2	31	Open	Natural	Sed	3.65
2	31	Open	Natural	Sed	5.02
2	31	Open	Natural	Sed	2.73
2	31	Open	Natural	Sed	3.15
2	31	Open	Natural	Sed	4.65
2	32	Open	Natural	Sed	0.00
2	32	Open	Natural	Sed	2.28
2	32	Open	Natural	Sed	1.82
2	32	Open	Natural	Sed	3.27
2	32	Open	Natural	Sed	2.13
2	32	Open	Natural	Sed	1.12

2	32	Open	Natural	Sed	0.00
2	32	Open	Natural	Sed	3.71
2	32	Open	Natural	Sed	1.27
2	32	Open	Natural	Sed	5.18
2	32	Open	Natural	Sed	2.60
2	32	Open	Natural	Sed	1.64
2	32	Open	Natural	Sed	0.89
2	32	Open	Natural	Sed	1.77
2	32	Open	Natural	Sed	2.36
2	32	Open	Natural	Sed	2.37
2	32	Open	Natural	Sed	1.97
2	32	Open	Natural	Sed	2.40
2	32	Open	Natural	Sed	2.19
2	32	Open	Natural	Sed	4.45
2	33	Open	Natural	Sed	1.06
2	33	Open	Natural	Sed	1.74
2	33	Open	Natural	Sed	2.78
2	33	Open	Natural	Sed	2.86
2	33	Open	Natural	Sed	2.41
2	33	Open	Natural	Sed	1.77
2	33	Open	Natural	Sed	2.81
2	33	Open	Natural	Sed	2.39
2	33	Open	Natural	Sed	1.27
2	33	Open	Natural	Sed	0.87
2	33	Open	Natural	Sed	3.64
2	33	Open	Natural	Sed	2.04
2	33	Open	Natural	Sed	1.54
2	33	Open	Natural	Sed	0.00
2	33	Open	Natural	Sed	1.20
2	33	Open	Natural	Sed	1.13
2	33	Open	Natural	Sed	1.86
2	33	Open	Natural	Sed	3.12
2	33	Open	Natural	Sed	2.12
2	33	Open	Natural	Sed	3.95
2	34	Open	Addition	Sed	1.88
2	34	Open	Addition	Sed	8.81
2	34	Open	Addition	Sed	2.76
2	34	Open	Addition	Sed	7.62
2	34	Open	Addition	Sed	9.04
2	34	Open	Addition	Sed	34.14
2	34	Open	Addition	Sed	16.13
2	34	Open	Addition	Sed	32.17
2	34	Open	Addition	Sed	5.25
2	34	Open	Addition	Sed	11.27
2	34	Open	Addition	Sed	19.50
2	34	Open	Addition	Sed	13.50
2	34	Open	Addition	Sed	4.74
2	34	Open	Addition	Sed	3.58
2	34	Open	Addition	Sed	4.73
2	34	Open	Addition	Sed	7.39
2	34	Open	Addition	Sed	8.09
2	34	Open	Addition	Sed	2.71
2	34	Open	Addition	Sed	2.95

2	34	Open	Addition	Sed	3.06
2	35	Open	Addition	Sed	3.56
2	35	Open	Addition	Sed	10.32
2	35	Open	Addition	Sed	4.63
2	35	Open	Addition	Sed	4.73
2	35	Open	Addition	Sed	5.18
2	35	Open	Addition	Sed	6.20
2	35	Open	Addition	Sed	12.24
2	35	Open	Addition	Sed	3.69
2	35	Open	Addition	Sed	7.68
2	35	Open	Addition	Sed	3.75
2	35	Open	Addition	Sed	9.97
2	35	Open	Addition	Sed	8.74
2	35	Open	Addition	Sed	8.46
2	35	Open	Addition	Sed	5.55
2	35	Open	Addition	Sed	5.00
2	35	Open	Addition	Sed	3.65
2	35	Open	Addition	Sed	4.88
2	35	Open	Addition	Sed	6.38
2	35	Open	Addition	Sed	0.89
2	35	Open	Addition	Sed	35.80
2	36	Open	Addition	Sed	1.81
2	36	Open	Addition	Sed	1.28
2	36	Open	Addition	Sed	1.62
2	36	Open	Addition	Sed	2.39
2	36	Open	Addition	Sed	10.34
2	36	Open	Addition	Sed	3.15
2	36	Open	Addition	Sed	4.27
2	36	Open	Addition	Sed	5.62
2	36	Open	Addition	Sed	1.57
2	36	Open	Addition	Sed	2.71
2	36	Open	Addition	Sed	8.34
2	36	Open	Addition	Sed	5.19
2	36	Open	Addition	Sed	3.02
2	36	Open	Addition	Sed	2.07
2	36	Open	Addition	Sed	1.60
2	36	Open	Addition	Sed	2.84
2	36	Open	Addition	Sed	5.77
2	36	Open	Addition	Sed	3.79
2	36	Open	Addition	Sed	2.08
2	36	Open	Addition	Sed	2.37
3	1	Full	Natural	Sed	1.80
3	1	Full	Natural	Sed	1.89
3	1	Full	Natural	Sed	2.39
3	1	Full	Natural	Sed	1.93
3	1	Full	Natural	Sed	2.46
3	1	Full	Natural	Sed	2.04
3	1	Full	Natural	Sed	3.62
3	1	Full	Natural	Sed	6.06
3	1	Full	Natural	Sed	8.18
3	1	Full	Natural	Sed	1.70
3	1	Full	Natural	Sed	4.23
3	1	Full	Natural	Sed	1.78

3	1	Full	Natural	Sed	1.84
3	1	Full	Natural	Sed	3.65
3	1	Full	Natural	Sed	2.49
3	1	Full	Natural	Sed	2.89
3	1	Full	Natural	Sed	2.88
3	1	Full	Natural	Sed	2.10
3	1	Full	Natural	Sed	4.01
3	1	Full	Natural	Sed	2.19
3	2	Full	Natural	Sed	3.13
3	2	Full	Natural	Sed	2.20
3	2	Full	Natural	Sed	3.23
3	2	Full	Natural	Sed	1.90
3	2	Full	Natural	Sed	3.47
3	2	Full	Natural	Sed	1.90
3	2	Full	Natural	Sed	3.21
3	2	Full	Natural	Sed	3.11
3	2	Full	Natural	Sed	2.35
3	2	Full	Natural	Sed	3.04
3	2	Full	Natural	Sed	7.55
3	2	Full	Natural	Sed	1.63
3	2	Full	Natural	Sed	4.36
3	2	Full	Natural	Sed	3.41
3	2	Full	Natural	Sed	4.34
3	2	Full	Natural	Sed	3.71
3	2	Full	Natural	Sed	3.26
3	2	Full	Natural	Sed	2.89
3	2	Full	Natural	Sed	3.39
3	2	Full	Natural	Sed	3.45
3	3	Full	Natural	Sed	8.18
3	3	Full	Natural	Sed	4.05
3	3	Full	Natural	Sed	1.94
3	3	Full	Natural	Sed	3.56
3	3	Full	Natural	Sed	2.16
3	3	Full	Natural	Sed	3.61
3	3	Full	Natural	Sed	2.47
3	3	Full	Natural	Sed	1.76
3	3	Full	Natural	Sed	3.71
3	3	Full	Natural	Sed	2.90
3	3	Full	Natural	Sed	3.15
3	3	Full	Natural	Sed	1.65
3	3	Full	Natural	Sed	2.18
3	3	Full	Natural	Sed	3.75
3	3	Full	Natural	Sed	2.65
3	3	Full	Natural	Sed	2.73
3	3	Full	Natural	Sed	2.11
3	3	Full	Natural	Sed	4.74
3	3	Full	Natural	Sed	4.05
3	3	Full	Natural	Sed	9.74
3	4	Full	Addition	Sed	5.58
3	4	Full	Addition	Sed	10.79
3	4	Full	Addition	Sed	3.19
3	4	Full	Addition	Sed	4.00
3	4	Full	Addition	Sed	1.64

3	4 Full	Addition	Sed	2.32
3	4 Full	Addition	Sed	4.33
3	4 Full	Addition	Sed	5.27
3	4 Full	Addition	Sed	3.68
3	4 Full	Addition	Sed	3.67
3	4 Full	Addition	Sed	3.08
3	4 Full	Addition	Sed	3.78
3	4 Full	Addition	Sed	3.50
3	4 Full	Addition	Sed	2.85
3	4 Full	Addition	Sed	2.32
3	4 Full	Addition	Sed	3.86
3	4 Full	Addition	Sed	2.72
3	4 Full	Addition	Sed	8.58
3	4 Full	Addition	Sed	24.64
3	4 Full	Addition	Sed	3.50
3	5 Full	Addition	Sed	1.72
3	5 Full	Addition	Sed	1.79
3	5 Full	Addition	Sed	3.84
3	5 Full	Addition	Sed	2.40
3	5 Full	Addition	Sed	3.53
3	5 Full	Addition	Sed	3.03
3	5 Full	Addition	Sed	2.96
3	5 Full	Addition	Sed	3.42
3	5 Full	Addition	Sed	3.49
3	5 Full	Addition	Sed	3.26
3	5 Full	Addition	Sed	2.87
3	5 Full	Addition	Sed	3.01
3	5 Full	Addition	Sed	3.02
3	5 Full	Addition	Sed	3.78
3	5 Full	Addition	Sed	4.29
3	5 Full	Addition	Sed	1.62
3	5 Full	Addition	Sed	2.73
3	5 Full	Addition	Sed	3.10
3	5 Full	Addition	Sed	3.58
3	5 Full	Addition	Sed	2.25
3	6 Full	Addition	Sed	1.78
3	6 Full	Addition	Sed	3.77
3	6 Full	Addition	Sed	2.31
3	6 Full	Addition	Sed	2.84
3	6 Full	Addition	Sed	2.24
3	6 Full	Addition	Sed	2.02
3	6 Full	Addition	Sed	4.08
3	6 Full	Addition	Sed	2.44
3	6 Full	Addition	Sed	3.55
3	6 Full	Addition	Sed	4.33
3	6 Full	Addition	Sed	5.88
3	6 Full	Addition	Sed	3.85
3	6 Full	Addition	Sed	3.30
3	6 Full	Addition	Sed	2.02
3	6 Full	Addition	Sed	3.39
3	6 Full	Addition	Sed	3.68
3	6 Full	Addition	Sed	3.76
3	6 Full	Addition	Sed	4.77

3	6	Full	Addition	Sed	4.14
3	6	Full	Addition	Sed	3.41
3	7	Half	Natural	Sed	0.94
3	7	Half	Natural	Sed	1.91
3	7	Half	Natural	Sed	0.77
3	7	Half	Natural	Sed	8.55
3	7	Half	Natural	Sed	1.15
3	7	Half	Natural	Sed	6.45
3	7	Half	Natural	Sed	2.59
3	7	Half	Natural	Sed	2.11
3	7	Half	Natural	Sed	2.32
3	7	Half	Natural	Sed	2.17
3	7	Half	Natural	Sed	1.96
3	7	Half	Natural	Sed	1.33
3	7	Half	Natural	Sed	1.46
3	7	Half	Natural	Sed	1.61
3	7	Half	Natural	Sed	3.98
3	7	Half	Natural	Sed	1.15
3	7	Half	Natural	Sed	1.12
3	7	Half	Natural	Sed	2.37
3	7	Half	Natural	Sed	2.19
3	7	Half	Natural	Sed	1.78
3	8	Half	Natural	Sed	2.18
3	8	Half	Natural	Sed	1.33
3	8	Half	Natural	Sed	1.89
3	8	Half	Natural	Sed	3.02
3	8	Half	Natural	Sed	2.06
3	8	Half	Natural	Sed	2.00
3	8	Half	Natural	Sed	1.55
3	8	Half	Natural	Sed	2.37
3	8	Half	Natural	Sed	2.44
3	8	Half	Natural	Sed	2.43
3	8	Half	Natural	Sed	2.52
3	8	Half	Natural	Sed	2.10
3	8	Half	Natural	Sed	2.04
3	8	Half	Natural	Sed	1.44
3	8	Half	Natural	Sed	2.13
3	8	Half	Natural	Sed	1.79
3	8	Half	Natural	Sed	2.28
3	8	Half	Natural	Sed	2.09
3	8	Half	Natural	Sed	3.71
3	8	Half	Natural	Sed	1.48
3	9	Half	Natural	Sed	1.80
3	9	Half	Natural	Sed	2.94
3	9	Half	Natural	Sed	3.87
3	9	Half	Natural	Sed	4.12
3	9	Half	Natural	Sed	1.98
3	9	Half	Natural	Sed	1.52
3	9	Half	Natural	Sed	2.45
3	9	Half	Natural	Sed	1.43
3	9	Half	Natural	Sed	1.36
3	9	Half	Natural	Sed	2.47
3	9	Half	Natural	Sed	3.16

3	9	Half	Natural	Sed	2.21
3	9	Half	Natural	Sed	2.79
3	9	Half	Natural	Sed	3.40
3	9	Half	Natural	Sed	4.16
3	9	Half	Natural	Sed	3.42
3	9	Half	Natural	Sed	2.52
3	9	Half	Natural	Sed	6.09
3	9	Half	Natural	Sed	3.30
3	9	Half	Natural	Sed	2.47
3	10	Half	Addition	Sed	1.75
3	10	Half	Addition	Sed	3.51
3	10	Half	Addition	Sed	3.22
3	10	Half	Addition	Sed	2.26
3	10	Half	Addition	Sed	2.84
3	10	Half	Addition	Sed	5.43
3	10	Half	Addition	Sed	3.02
3	10	Half	Addition	Sed	2.19
3	10	Half	Addition	Sed	1.50
3	10	Half	Addition	Sed	3.00
3	10	Half	Addition	Sed	2.84
3	10	Half	Addition	Sed	5.82
3	10	Half	Addition	Sed	2.66
3	10	Half	Addition	Sed	1.80
3	10	Half	Addition	Sed	2.43
3	10	Half	Addition	Sed	1.63
3	10	Half	Addition	Sed	3.01
3	10	Half	Addition	Sed	2.41
3	10	Half	Addition	Sed	4.45
3	10	Half	Addition	Sed	1.60
3	11	Half	Addition	Sed	3.33
3	11	Half	Addition	Sed	5.97
3	11	Half	Addition	Sed	6.70
3	11	Half	Addition	Sed	3.66
3	11	Half	Addition	Sed	1.57
3	11	Half	Addition	Sed	6.95
3	11	Half	Addition	Sed	3.65
3	11	Half	Addition	Sed	2.05
3	11	Half	Addition	Sed	3.20
3	11	Half	Addition	Sed	3.12
3	11	Half	Addition	Sed	2.00
3	11	Half	Addition	Sed	1.88
3	11	Half	Addition	Sed	2.79
3	11	Half	Addition	Sed	0.00
3	11	Half	Addition	Sed	1.55
3	11	Half	Addition	Sed	2.68
3	11	Half	Addition	Sed	3.04
3	11	Half	Addition	Sed	2.91
3	11	Half	Addition	Sed	3.97
3	11	Half	Addition	Sed	4.17
3	12	Half	Addition	Sed	2.06
3	12	Half	Addition	Sed	2.72
3	12	Half	Addition	Sed	2.65
3	12	Half	Addition	Sed	3.39

3	12	Half	Addition	Sed	2.92
3	12	Half	Addition	Sed	5.32
3	12	Half	Addition	Sed	6.60
3	12	Half	Addition	Sed	2.83
3	12	Half	Addition	Sed	4.21
3	12	Half	Addition	Sed	0.00
3	12	Half	Addition	Sed	2.78
3	12	Half	Addition	Sed	2.49
3	12	Half	Addition	Sed	2.46
3	12	Half	Addition	Sed	3.70
3	12	Half	Addition	Sed	5.36
3	12	Half	Addition	Sed	2.16
3	12	Half	Addition	Sed	2.49
3	12	Half	Addition	Sed	12.22
3	12	Half	Addition	Sed	1.78
3	12	Half	Addition	Sed	3.31
3	13	Open	Natural	Sed	0.69
3	13	Open	Natural	Sed	1.54
3	13	Open	Natural	Sed	5.48
3	13	Open	Natural	Sed	1.37
3	13	Open	Natural	Sed	1.90
3	13	Open	Natural	Sed	1.49
3	13	Open	Natural	Sed	4.61
3	13	Open	Natural	Sed	3.90
3	13	Open	Natural	Sed	3.35
3	13	Open	Natural	Sed	2.25
3	13	Open	Natural	Sed	1.15
3	13	Open	Natural	Sed	3.22
3	13	Open	Natural	Sed	4.07
3	13	Open	Natural	Sed	1.88
3	13	Open	Natural	Sed	0.94
3	13	Open	Natural	Sed	1.69
3	13	Open	Natural	Sed	4.54
3	13	Open	Natural	Sed	4.88
3	13	Open	Natural	Sed	1.62
3	13	Open	Natural	Sed	1.76
3	14	Open	Natural	Sed	1.60
3	14	Open	Natural	Sed	8.31
3	14	Open	Natural	Sed	4.52
3	14	Open	Natural	Sed	4.34
3	14	Open	Natural	Sed	1.73
3	14	Open	Natural	Sed	3.91
3	14	Open	Natural	Sed	6.92
3	14	Open	Natural	Sed	4.13
3	14	Open	Natural	Sed	2.44
3	14	Open	Natural	Sed	2.02
3	14	Open	Natural	Sed	5.34
3	14	Open	Natural	Sed	2.45
3	14	Open	Natural	Sed	1.09
3	14	Open	Natural	Sed	0.96
3	14	Open	Natural	Sed	1.91
3	14	Open	Natural	Sed	2.26
3	14	Open	Natural	Sed	0.66

3	14	Open	Natural	Sed	2.75
3	14	Open	Natural	Sed	3.02
3	14	Open	Natural	Sed	1.75
3	15	Open	Natural	Sed	0.78
3	15	Open	Natural	Sed	0.00
3	15	Open	Natural	Sed	0.77
3	15	Open	Natural	Sed	2.72
3	15	Open	Natural	Sed	1.22
3	15	Open	Natural	Sed	0.00
3	15	Open	Natural	Sed	2.76
3	15	Open	Natural	Sed	1.43
3	15	Open	Natural	Sed	2.62
3	15	Open	Natural	Sed	1.23
3	15	Open	Natural	Sed	2.32
3	15	Open	Natural	Sed	2.23
3	15	Open	Natural	Sed	2.74
3	15	Open	Natural	Sed	5.32
3	15	Open	Natural	Sed	0.99
3	15	Open	Natural	Sed	2.28
3	15	Open	Natural	Sed	2.36
3	15	Open	Natural	Sed	1.59
3	15	Open	Natural	Sed	0.00
3	15	Open	Natural	Sed	0.00
3	16	Open	Addition	Sed	1.90
3	16	Open	Addition	Sed	1.50
3	16	Open	Addition	Sed	1.08
3	16	Open	Addition	Sed	6.51
3	16	Open	Addition	Sed	5.81
3	16	Open	Addition	Sed	2.03
3	16	Open	Addition	Sed	7.17
3	16	Open	Addition	Sed	2.23
3	16	Open	Addition	Sed	1.94
3	16	Open	Addition	Sed	2.65
3	16	Open	Addition	Sed	1.17
3	16	Open	Addition	Sed	1.71
3	16	Open	Addition	Sed	3.06
3	16	Open	Addition	Sed	2.86
3	16	Open	Addition	Sed	2.03
3	16	Open	Addition	Sed	1.38
3	16	Open	Addition	Sed	2.48
3	16	Open	Addition	Sed	1.73
3	16	Open	Addition	Sed	2.37
3	16	Open	Addition	Sed	2.73
3	17	Open	Addition	Sed	1.32
3	17	Open	Addition	Sed	5.74
3	17	Open	Addition	Sed	8.73
3	17	Open	Addition	Sed	2.07
3	17	Open	Addition	Sed	12.81
3	17	Open	Addition	Sed	3.78
3	17	Open	Addition	Sed	6.66
3	17	Open	Addition	Sed	2.66
3	17	Open	Addition	Sed	1.00
3	17	Open	Addition	Sed	1.87

3	17	Open	Addition	Sed	0.67
3	17	Open	Addition	Sed	1.94
3	17	Open	Addition	Sed	1.82
3	17	Open	Addition	Sed	0.80
3	17	Open	Addition	Sed	1.93
3	17	Open	Addition	Sed	2.03
3	17	Open	Addition	Sed	2.55
3	17	Open	Addition	Sed	5.35
3	17	Open	Addition	Sed	5.13
3	17	Open	Addition	Sed	1.91
3	18	Open	Addition	Sed	4.35
3	18	Open	Addition	Sed	3.22
3	18	Open	Addition	Sed	3.69
3	18	Open	Addition	Sed	4.03
3	18	Open	Addition	Sed	2.93
3	18	Open	Addition	Sed	4.52
3	18	Open	Addition	Sed	4.05
3	18	Open	Addition	Sed	4.92
3	18	Open	Addition	Sed	2.54
3	18	Open	Addition	Sed	3.02
3	18	Open	Addition	Sed	2.22
3	18	Open	Addition	Sed	1.69
3	18	Open	Addition	Sed	3.40
3	18	Open	Addition	Sed	1.46
3	18	Open	Addition	Sed	1.38
3	18	Open	Addition	Sed	1.79
3	18	Open	Addition	Sed	1.80
3	18	Open	Addition	Sed	0.00
3	18	Open	Addition	Sed	3.19
3	18	Open	Addition	Sed	1.30
3	19	Cage	Natural	Sed	2.67
3	19	Cage	Natural	Sed	1.41
3	19	Cage	Natural	Sed	1.90
3	19	Cage	Natural	Sed	12.75
3	19	Cage	Natural	Sed	8.19
3	19	Cage	Natural	Sed	9.69
3	19	Cage	Natural	Sed	4.54
3	19	Cage	Natural	Sed	5.72
3	19	Cage	Natural	Sed	2.57
3	19	Cage	Natural	Sed	3.00
3	19	Cage	Natural	Sed	9.18
3	19	Cage	Natural	Sed	12.70
3	19	Cage	Natural	Sed	2.11
3	19	Cage	Natural	Sed	1.88
3	19	Cage	Natural	Sed	6.60
3	19	Cage	Natural	Sed	3.76
3	19	Cage	Natural	Sed	2.39
3	19	Cage	Natural	Sed	4.60
3	19	Cage	Natural	Sed	1.74
3	19	Cage	Natural	Sed	0.96
3	20	Cage	Natural	Sed	5.21
3	20	Cage	Natural	Sed	3.10
3	20	Cage	Natural	Sed	2.57

3	20	Cage	Natural	Sed	1.11
3	20	Cage	Natural	Sed	3.90
3	20	Cage	Natural	Sed	2.03
3	20	Cage	Natural	Sed	1.30
3	20	Cage	Natural	Sed	1.85
3	20	Cage	Natural	Sed	1.39
3	20	Cage	Natural	Sed	11.50
3	20	Cage	Natural	Sed	2.54
3	20	Cage	Natural	Sed	2.23
3	20	Cage	Natural	Sed	2.38
3	20	Cage	Natural	Sed	3.81
3	20	Cage	Natural	Sed	2.58
3	20	Cage	Natural	Sed	3.53
3	20	Cage	Natural	Sed	6.18
3	20	Cage	Natural	Sed	0.74
3	20	Cage	Natural	Sed	2.36
3	20	Cage	Natural	Sed	4.03
3	21	Cage	Natural	Sed	2.16
3	21	Cage	Natural	Sed	2.35
3	21	Cage	Natural	Sed	2.47
3	21	Cage	Natural	Sed	1.92
3	21	Cage	Natural	Sed	5.03
3	21	Cage	Natural	Sed	2.90
3	21	Cage	Natural	Sed	8.22
3	21	Cage	Natural	Sed	1.76
3	21	Cage	Natural	Sed	0.77
3	21	Cage	Natural	Sed	1.19
3	21	Cage	Natural	Sed	2.43
3	21	Cage	Natural	Sed	4.32
3	21	Cage	Natural	Sed	2.49
3	21	Cage	Natural	Sed	1.67
3	21	Cage	Natural	Sed	5.31
3	21	Cage	Natural	Sed	3.70
3	21	Cage	Natural	Sed	3.00
3	21	Cage	Natural	Sed	4.30
3	21	Cage	Natural	Sed	2.48
3	21	Cage	Natural	Sed	2.45
3	22	Cage	Addition	Sed	3.12
3	22	Cage	Addition	Sed	4.98
3	22	Cage	Addition	Sed	1.03
3	22	Cage	Addition	Sed	4.05
3	22	Cage	Addition	Sed	10.87
3	22	Cage	Addition	Sed	12.01
3	22	Cage	Addition	Sed	5.68
3	22	Cage	Addition	Sed	19.38
3	22	Cage	Addition	Sed	1.12
3	22	Cage	Addition	Sed	5.12
3	22	Cage	Addition	Sed	1.94
3	22	Cage	Addition	Sed	3.40
3	22	Cage	Addition	Sed	4.49
3	22	Cage	Addition	Sed	2.35
3	22	Cage	Addition	Sed	28.25
3	22	Cage	Addition	Sed	44.76

3	22	Cage	Addition	Sed	15.70
3	22	Cage	Addition	Sed	14.75
3	22	Cage	Addition	Sed	51.37
3	22	Cage	Addition	Sed	37.13
3	23	Cage	Addition	Sed	4.90
3	23	Cage	Addition	Sed	2.07
3	23	Cage	Addition	Sed	4.41
3	23	Cage	Addition	Sed	9.58
3	23	Cage	Addition	Sed	22.34
3	23	Cage	Addition	Sed	10.03
3	23	Cage	Addition	Sed	10.49
3	23	Cage	Addition	Sed	12.04
3	23	Cage	Addition	Sed	7.04
3	23	Cage	Addition	Sed	3.90
3	23	Cage	Addition	Sed	3.68
3	23	Cage	Addition	Sed	3.66
3	23	Cage	Addition	Sed	7.79
3	23	Cage	Addition	Sed	4.72
3	23	Cage	Addition	Sed	3.53
3	23	Cage	Addition	Sed	15.47
3	23	Cage	Addition	Sed	17.65
3	23	Cage	Addition	Sed	11.60
3	23	Cage	Addition	Sed	13.20
3	23	Cage	Addition	Sed	44.28
3	24	Cage	Addition	Sed	1.85
3	24	Cage	Addition	Sed	4.85
3	24	Cage	Addition	Sed	8.03
3	24	Cage	Addition	Sed	3.57
3	24	Cage	Addition	Sed	1.29
3	24	Cage	Addition	Sed	10.29
3	24	Cage	Addition	Sed	6.69
3	24	Cage	Addition	Sed	15.64
3	24	Cage	Addition	Sed	1.67
3	24	Cage	Addition	Sed	4.92
3	24	Cage	Addition	Sed	16.23
3	24	Cage	Addition	Sed	6.72
3	24	Cage	Addition	Sed	3.37
3	24	Cage	Addition	Sed	2.80
3	24	Cage	Addition	Sed	1.60
3	24	Cage	Addition	Sed	6.26
3	24	Cage	Addition	Sed	2.52
3	24	Cage	Addition	Sed	6.44
3	24	Cage	Addition	Sed	5.16
3	24	Cage	Addition	Sed	4.65
3	25	Half	Natural	Sed	1.50
3	25	Half	Natural	Sed	2.24
3	25	Half	Natural	Sed	0.95
3	25	Half	Natural	Sed	1.53
3	25	Half	Natural	Sed	3.03
3	25	Half	Natural	Sed	0.76
3	25	Half	Natural	Sed	2.19
3	25	Half	Natural	Sed	1.56
3	25	Half	Natural	Sed	1.23

3	25	Half	Natural	Sed	2.25
3	25	Half	Natural	Sed	1.22
3	25	Half	Natural	Sed	1.38
3	25	Half	Natural	Sed	5.75
3	25	Half	Natural	Sed	2.38
3	25	Half	Natural	Sed	7.65
3	25	Half	Natural	Sed	0.73
3	25	Half	Natural	Sed	1.61
3	25	Half	Natural	Sed	1.73
3	25	Half	Natural	Sed	1.72
3	25	Half	Natural	Sed	1.19
3	26	Half	Natural	Sed	0.78
3	26	Half	Natural	Sed	3.11
3	26	Half	Natural	Sed	2.30
3	26	Half	Natural	Sed	1.34
3	26	Half	Natural	Sed	1.39
3	26	Half	Natural	Sed	5.00
3	26	Half	Natural	Sed	0.91
3	26	Half	Natural	Sed	2.02
3	26	Half	Natural	Sed	1.18
3	26	Half	Natural	Sed	0.96
3	26	Half	Natural	Sed	1.80
3	26	Half	Natural	Sed	1.07
3	26	Half	Natural	Sed	1.89
3	26	Half	Natural	Sed	0.52
3	26	Half	Natural	Sed	2.10
3	26	Half	Natural	Sed	2.12
3	26	Half	Natural	Sed	1.12
3	26	Half	Natural	Sed	2.14
3	26	Half	Natural	Sed	1.81
3	26	Half	Natural	Sed	3.00
3	27	Half	Natural	Sed	2.30
3	27	Half	Natural	Sed	1.49
3	27	Half	Natural	Sed	1.10
3	27	Half	Natural	Sed	0.87
3	27	Half	Natural	Sed	1.74
3	27	Half	Natural	Sed	2.06
3	27	Half	Natural	Sed	2.18
3	27	Half	Natural	Sed	1.31
3	27	Half	Natural	Sed	1.76
3	27	Half	Natural	Sed	0.71
3	27	Half	Natural	Sed	1.39
3	27	Half	Natural	Sed	1.63
3	27	Half	Natural	Sed	2.44
3	27	Half	Natural	Sed	1.69
3	27	Half	Natural	Sed	1.00
3	27	Half	Natural	Sed	1.79
3	27	Half	Natural	Sed	1.45
3	27	Half	Natural	Sed	1.99
3	27	Half	Natural	Sed	2.43
3	27	Half	Natural	Sed	2.35
3	28	Half	Addition	Sed	3.97
3	28	Half	Addition	Sed	8.08

3	28	Half	Addition	Sed	4.24
3	28	Half	Addition	Sed	9.50
3	28	Half	Addition	Sed	2.82
3	28	Half	Addition	Sed	9.79
3	28	Half	Addition	Sed	5.40
3	28	Half	Addition	Sed	4.93
3	28	Half	Addition	Sed	3.33
3	28	Half	Addition	Sed	11.71
3	28	Half	Addition	Sed	4.43
3	28	Half	Addition	Sed	20.73
3	28	Half	Addition	Sed	29.38
3	28	Half	Addition	Sed	6.56
3	28	Half	Addition	Sed	3.02
3	28	Half	Addition	Sed	3.29
3	28	Half	Addition	Sed	7.33
3	28	Half	Addition	Sed	2.34
3	28	Half	Addition	Sed	2.33
3	28	Half	Addition	Sed	5.24
3	29	Half	Addition	Sed	4.81
3	29	Half	Addition	Sed	9.37
3	29	Half	Addition	Sed	1.48
3	29	Half	Addition	Sed	3.77
3	29	Half	Addition	Sed	3.54
3	29	Half	Addition	Sed	18.98
3	29	Half	Addition	Sed	2.27
3	29	Half	Addition	Sed	3.84
3	29	Half	Addition	Sed	2.90
3	29	Half	Addition	Sed	9.42
3	29	Half	Addition	Sed	14.96
3	29	Half	Addition	Sed	13.48
3	29	Half	Addition	Sed	8.30
3	29	Half	Addition	Sed	6.67
3	29	Half	Addition	Sed	8.27
3	29	Half	Addition	Sed	5.80
3	29	Half	Addition	Sed	3.31
3	29	Half	Addition	Sed	2.18
3	29	Half	Addition	Sed	31.51
3	29	Half	Addition	Sed	25.59
3	30	Half	Addition	Sed	3.01
3	30	Half	Addition	Sed	11.01
3	30	Half	Addition	Sed	6.07
3	30	Half	Addition	Sed	3.15
3	30	Half	Addition	Sed	6.52
3	30	Half	Addition	Sed	8.51
3	30	Half	Addition	Sed	3.13
3	30	Half	Addition	Sed	9.41
3	30	Half	Addition	Sed	4.06
3	30	Half	Addition	Sed	4.95
3	30	Half	Addition	Sed	10.17
3	30	Half	Addition	Sed	12.55
3	30	Half	Addition	Sed	10.12
3	30	Half	Addition	Sed	3.06
3	30	Half	Addition	Sed	3.27

3	30	Half	Addition	Sed	4.20
3	30	Half	Addition	Sed	2.81
3	30	Half	Addition	Sed	2.04
3	30	Half	Addition	Sed	3.33
3	30	Half	Addition	Sed	40.84
3	31	Open	Natural	Sed	1.65
3	31	Open	Natural	Sed	2.92
3	31	Open	Natural	Sed	2.42
3	31	Open	Natural	Sed	5.15
3	31	Open	Natural	Sed	1.20
3	31	Open	Natural	Sed	2.27
3	31	Open	Natural	Sed	2.42
3	31	Open	Natural	Sed	1.13
3	31	Open	Natural	Sed	3.25
3	31	Open	Natural	Sed	2.95
3	31	Open	Natural	Sed	1.14
3	31	Open	Natural	Sed	1.84
3	31	Open	Natural	Sed	1.04
3	31	Open	Natural	Sed	0.92
3	31	Open	Natural	Sed	1.27
3	31	Open	Natural	Sed	3.00
3	31	Open	Natural	Sed	1.93
3	31	Open	Natural	Sed	1.09
3	31	Open	Natural	Sed	1.19
3	31	Open	Natural	Sed	1.92
3	32	Open	Natural	Sed	1.27
3	32	Open	Natural	Sed	1.13
3	32	Open	Natural	Sed	3.25
3	32	Open	Natural	Sed	0.00
3	32	Open	Natural	Sed	1.27
3	32	Open	Natural	Sed	2.75
3	32	Open	Natural	Sed	1.94
3	32	Open	Natural	Sed	2.06
3	32	Open	Natural	Sed	0.97
3	32	Open	Natural	Sed	0.92
3	32	Open	Natural	Sed	2.51
3	32	Open	Natural	Sed	0.80
3	32	Open	Natural	Sed	1.51
3	32	Open	Natural	Sed	1.47
3	32	Open	Natural	Sed	2.37
3	32	Open	Natural	Sed	1.15
3	32	Open	Natural	Sed	0.41
3	32	Open	Natural	Sed	0.67
3	32	Open	Natural	Sed	2.14
3	32	Open	Natural	Sed	1.93
3	33	Open	Natural	Sed	1.20
3	33	Open	Natural	Sed	0.00
3	33	Open	Natural	Sed	5.07
3	33	Open	Natural	Sed	1.63
3	33	Open	Natural	Sed	4.21
3	33	Open	Natural	Sed	4.56
3	33	Open	Natural	Sed	1.26
3	33	Open	Natural	Sed	2.26

3	33	Open	Natural	Sed	1.12
3	33	Open	Natural	Sed	1.85
3	33	Open	Natural	Sed	1.09
3	33	Open	Natural	Sed	1.65
3	33	Open	Natural	Sed	1.59
3	33	Open	Natural	Sed	1.41
3	33	Open	Natural	Sed	0.65
3	33	Open	Natural	Sed	1.86
3	33	Open	Natural	Sed	0.89
3	33	Open	Natural	Sed	0.00
3	33	Open	Natural	Sed	1.08
3	33	Open	Natural	Sed	1.49
3	34	Open	Addition	Sed	3.75
3	34	Open	Addition	Sed	10.76
3	34	Open	Addition	Sed	11.08
3	34	Open	Addition	Sed	5.78
3	34	Open	Addition	Sed	7.13
3	34	Open	Addition	Sed	2.38
3	34	Open	Addition	Sed	7.59
3	34	Open	Addition	Sed	1.45
3	34	Open	Addition	Sed	5.25
3	34	Open	Addition	Sed	2.34
3	34	Open	Addition	Sed	6.15
3	34	Open	Addition	Sed	4.34
3	34	Open	Addition	Sed	7.97
3	34	Open	Addition	Sed	4.86
3	34	Open	Addition	Sed	2.00
3	34	Open	Addition	Sed	15.68
3	34	Open	Addition	Sed	16.95
3	34	Open	Addition	Sed	13.99
3	34	Open	Addition	Sed	19.09
3	34	Open	Addition	Sed	13.18
3	35	Open	Addition	Sed	11.99
3	35	Open	Addition	Sed	3.21
3	35	Open	Addition	Sed	11.83
3	35	Open	Addition	Sed	2.11
3	35	Open	Addition	Sed	3.20
3	35	Open	Addition	Sed	3.99
3	35	Open	Addition	Sed	4.84
3	35	Open	Addition	Sed	5.10
3	35	Open	Addition	Sed	12.14
3	35	Open	Addition	Sed	1.04
3	35	Open	Addition	Sed	22.87
3	35	Open	Addition	Sed	38.54
3	35	Open	Addition	Sed	3.56
3	35	Open	Addition	Sed	4.22
3	35	Open	Addition	Sed	8.91
3	35	Open	Addition	Sed	7.43
3	35	Open	Addition	Sed	4.03
3	35	Open	Addition	Sed	3.54
3	35	Open	Addition	Sed	1.00
3	35	Open	Addition	Sed	2.61
3	36	Open	Addition	Sed	3.62

3	36	Open	Addition	Sed	2.87
3	36	Open	Addition	Sed	7.77
3	36	Open	Addition	Sed	5.54
3	36	Open	Addition	Sed	1.40
3	36	Open	Addition	Sed	3.41
3	36	Open	Addition	Sed	2.43
3	36	Open	Addition	Sed	2.24
3	36	Open	Addition	Sed	2.08
3	36	Open	Addition	Sed	1.63
3	36	Open	Addition	Sed	4.49
3	36	Open	Addition	Sed	2.76
3	36	Open	Addition	Sed	1.77
3	36	Open	Addition	Sed	1.56
3	36	Open	Addition	Sed	1.32
3	36	Open	Addition	Sed	4.10
3	36	Open	Addition	Sed	0.00
3	36	Open	Addition	Sed	2.48
3	36	Open	Addition	Sed	4.97
3	36	Open	Addition	Sed	2.21
4	1	Full	Natural	Sed	0.81
4	1	Full	Natural	Sed	3.45
4	1	Full	Natural	Sed	1.39
4	1	Full	Natural	Sed	3.61
4	1	Full	Natural	Sed	1.48
4	1	Full	Natural	Sed	2.62
4	1	Full	Natural	Sed	1.44
4	1	Full	Natural	Sed	4.94
4	1	Full	Natural	Sed	2.34
4	1	Full	Natural	Sed	1.59
4	1	Full	Natural	Sed	3.14
4	1	Full	Natural	Sed	1.80
4	1	Full	Natural	Sed	3.84
4	1	Full	Natural	Sed	1.55
4	1	Full	Natural	Sed	1.58
4	1	Full	Natural	Sed	0.97
4	1	Full	Natural	Sed	4.05
4	1	Full	Natural	Sed	1.80
4	1	Full	Natural	Sed	2.28
4	1	Full	Natural	Sed	2.32
4	2	Full	Natural	Sed	6.94
4	2	Full	Natural	Sed	7.38
4	2	Full	Natural	Sed	1.89
4	2	Full	Natural	Sed	0.93
4	2	Full	Natural	Sed	2.08
4	2	Full	Natural	Sed	0.00
4	2	Full	Natural	Sed	1.75
4	2	Full	Natural	Sed	2.14
4	2	Full	Natural	Sed	0.91
4	2	Full	Natural	Sed	1.01
4	2	Full	Natural	Sed	1.04
4	2	Full	Natural	Sed	4.41
4	2	Full	Natural	Sed	2.34
4	2	Full	Natural	Sed	0.96

4	2	Full	Natural	Sed	1.96
4	2	Full	Natural	Sed	2.47
4	2	Full	Natural	Sed	6.66
4	2	Full	Natural	Sed	2.47
4	2	Full	Natural	Sed	1.61
4	2	Full	Natural	Sed	0.97
4	3	Full	Natural	Sed	2.16
4	3	Full	Natural	Sed	5.30
4	3	Full	Natural	Sed	2.82
4	3	Full	Natural	Sed	4.90
4	3	Full	Natural	Sed	8.29
4	3	Full	Natural	Sed	5.00
4	3	Full	Natural	Sed	7.13
4	3	Full	Natural	Sed	1.62
4	3	Full	Natural	Sed	2.54
4	3	Full	Natural	Sed	0.90
4	3	Full	Natural	Sed	2.12
4	3	Full	Natural	Sed	0.70
4	3	Full	Natural	Sed	2.97
4	3	Full	Natural	Sed	5.60
4	3	Full	Natural	Sed	4.93
4	3	Full	Natural	Sed	2.63
4	3	Full	Natural	Sed	4.00
4	3	Full	Natural	Sed	0.94
4	3	Full	Natural	Sed	2.30
4	3	Full	Natural	Sed	12.97
4	4	Full	Addition	Sed	0.58
4	4	Full	Addition	Sed	1.10
4	4	Full	Addition	Sed	2.22
4	4	Full	Addition	Sed	1.91
4	4	Full	Addition	Sed	2.78
4	4	Full	Addition	Sed	2.12
4	4	Full	Addition	Sed	2.73
4	4	Full	Addition	Sed	2.23
4	4	Full	Addition	Sed	4.96
4	4	Full	Addition	Sed	8.65
4	4	Full	Addition	Sed	9.29
4	4	Full	Addition	Sed	3.22
4	4	Full	Addition	Sed	3.45
4	4	Full	Addition	Sed	2.15
4	4	Full	Addition	Sed	2.12
4	4	Full	Addition	Sed	3.15
4	4	Full	Addition	Sed	2.08
4	4	Full	Addition	Sed	2.95
4	4	Full	Addition	Sed	2.91
4	4	Full	Addition	Sed	3.34
4	5	Full	Addition	Sed	1.56
4	5	Full	Addition	Sed	6.10
4	5	Full	Addition	Sed	2.62
4	5	Full	Addition	Sed	3.81
4	5	Full	Addition	Sed	3.12
4	5	Full	Addition	Sed	1.51
4	5	Full	Addition	Sed	3.36

4	5	Full	Addition	Sed	2.21
4	5	Full	Addition	Sed	4.21
4	5	Full	Addition	Sed	2.46
4	5	Full	Addition	Sed	1.44
4	5	Full	Addition	Sed	3.98
4	5	Full	Addition	Sed	0.90
4	5	Full	Addition	Sed	2.49
4	5	Full	Addition	Sed	2.92
4	5	Full	Addition	Sed	1.73
4	5	Full	Addition	Sed	2.38
4	5	Full	Addition	Sed	4.19
4	5	Full	Addition	Sed	1.04
4	5	Full	Addition	Sed	0.00
4	6	Full	Addition	Sed	7.60
4	6	Full	Addition	Sed	4.84
4	6	Full	Addition	Sed	2.11
4	6	Full	Addition	Sed	3.39
4	6	Full	Addition	Sed	5.69
4	6	Full	Addition	Sed	1.29
4	6	Full	Addition	Sed	4.72
4	6	Full	Addition	Sed	3.97
4	6	Full	Addition	Sed	3.74
4	6	Full	Addition	Sed	0.98
4	6	Full	Addition	Sed	1.22
4	6	Full	Addition	Sed	3.24
4	6	Full	Addition	Sed	2.00
4	6	Full	Addition	Sed	0.75
4	6	Full	Addition	Sed	4.19
4	6	Full	Addition	Sed	2.23
4	6	Full	Addition	Sed	1.52
4	6	Full	Addition	Sed	3.44
4	6	Full	Addition	Sed	1.46
4	6	Full	Addition	Sed	1.25
4	7	Half	Natural	Sed	3.51
4	7	Half	Natural	Sed	1.45
4	7	Half	Natural	Sed	3.15
4	7	Half	Natural	Sed	1.16
4	7	Half	Natural	Sed	4.69
4	7	Half	Natural	Sed	1.72
4	7	Half	Natural	Sed	0.82
4	7	Half	Natural	Sed	1.45
4	7	Half	Natural	Sed	2.00
4	7	Half	Natural	Sed	3.20
4	7	Half	Natural	Sed	1.52
4	7	Half	Natural	Sed	1.45
4	7	Half	Natural	Sed	2.25
4	7	Half	Natural	Sed	2.15
4	7	Half	Natural	Sed	1.52
4	7	Half	Natural	Sed	3.02
4	7	Half	Natural	Sed	1.23
4	7	Half	Natural	Sed	2.44
4	7	Half	Natural	Sed	0.00
4	7	Half	Natural	Sed	1.06

4	8	Half	Natural	Sed	7.56
4	8	Half	Natural	Sed	1.56
4	8	Half	Natural	Sed	1.74
4	8	Half	Natural	Sed	1.08
4	8	Half	Natural	Sed	2.22
4	8	Half	Natural	Sed	2.30
4	8	Half	Natural	Sed	0.49
4	8	Half	Natural	Sed	0.94
4	8	Half	Natural	Sed	1.85
4	8	Half	Natural	Sed	0.61
4	8	Half	Natural	Sed	1.77
4	8	Half	Natural	Sed	0.97
4	8	Half	Natural	Sed	0.84
4	8	Half	Natural	Sed	2.24
4	8	Half	Natural	Sed	1.93
4	8	Half	Natural	Sed	3.62
4	8	Half	Natural	Sed	2.45
4	8	Half	Natural	Sed	1.72
4	8	Half	Natural	Sed	2.36
4	8	Half	Natural	Sed	1.21
4	9	Half	Natural	Sed	2.47
4	9	Half	Natural	Sed	2.35
4	9	Half	Natural	Sed	2.15
4	9	Half	Natural	Sed	0.81
4	9	Half	Natural	Sed	0.94
4	9	Half	Natural	Sed	1.38
4	9	Half	Natural	Sed	1.41
4	9	Half	Natural	Sed	1.65
4	9	Half	Natural	Sed	1.51
4	9	Half	Natural	Sed	4.14
4	9	Half	Natural	Sed	9.23
4	9	Half	Natural	Sed	1.09
4	9	Half	Natural	Sed	5.36
4	9	Half	Natural	Sed	0.76
4	9	Half	Natural	Sed	2.18
4	9	Half	Natural	Sed	1.97
4	9	Half	Natural	Sed	0.44
4	9	Half	Natural	Sed	1.28
4	9	Half	Natural	Sed	1.47
4	9	Half	Natural	Sed	1.96
4	10	Half	Addition	Sed	3.83
4	10	Half	Addition	Sed	1.06
4	10	Half	Addition	Sed	4.75
4	10	Half	Addition	Sed	2.26
4	10	Half	Addition	Sed	2.67
4	10	Half	Addition	Sed	0.85
4	10	Half	Addition	Sed	1.18
4	10	Half	Addition	Sed	1.27
4	10	Half	Addition	Sed	12.30
4	10	Half	Addition	Sed	4.31
4	10	Half	Addition	Sed	1.44
4	10	Half	Addition	Sed	3.82
4	10	Half	Addition	Sed	0.89

4	10	Half	Addition	Sed	2.36
4	10	Half	Addition	Sed	1.68
4	10	Half	Addition	Sed	0.93
4	10	Half	Addition	Sed	2.22
4	10	Half	Addition	Sed	3.44
4	10	Half	Addition	Sed	1.63
4	10	Half	Addition	Sed	1.63
4	11	Half	Addition	Sed	0.64
4	11	Half	Addition	Sed	2.80
4	11	Half	Addition	Sed	4.56
4	11	Half	Addition	Sed	1.14
4	11	Half	Addition	Sed	0.78
4	11	Half	Addition	Sed	10.30
4	11	Half	Addition	Sed	0.99
4	11	Half	Addition	Sed	4.84
4	11	Half	Addition	Sed	1.52
4	11	Half	Addition	Sed	2.27
4	11	Half	Addition	Sed	12.45
4	11	Half	Addition	Sed	2.47
4	11	Half	Addition	Sed	4.16
4	11	Half	Addition	Sed	5.58
4	11	Half	Addition	Sed	4.30
4	11	Half	Addition	Sed	0.71
4	11	Half	Addition	Sed	1.47
4	11	Half	Addition	Sed	1.76
4	11	Half	Addition	Sed	1.45
4	11	Half	Addition	Sed	3.05
4	12	Half	Addition	Sed	1.00
4	12	Half	Addition	Sed	1.99
4	12	Half	Addition	Sed	1.91
4	12	Half	Addition	Sed	6.39
4	12	Half	Addition	Sed	2.82
4	12	Half	Addition	Sed	1.83
4	12	Half	Addition	Sed	2.04
4	12	Half	Addition	Sed	1.03
4	12	Half	Addition	Sed	1.52
4	12	Half	Addition	Sed	1.06
4	12	Half	Addition	Sed	2.01
4	12	Half	Addition	Sed	2.45
4	12	Half	Addition	Sed	1.77
4	12	Half	Addition	Sed	4.91
4	12	Half	Addition	Sed	1.78
4	12	Half	Addition	Sed	2.23
4	12	Half	Addition	Sed	2.10
4	12	Half	Addition	Sed	4.63
4	12	Half	Addition	Sed	2.47
4	12	Half	Addition	Sed	1.28
4	13	Open	Natural	Sed	0.88
4	13	Open	Natural	Sed	3.70
4	13	Open	Natural	Sed	2.21
4	13	Open	Natural	Sed	2.75
4	13	Open	Natural	Sed	3.42
4	13	Open	Natural	Sed	0.58

4	13	Open	Natural	Sed	1.94
4	13	Open	Natural	Sed	1.06
4	13	Open	Natural	Sed	1.95
4	13	Open	Natural	Sed	2.28
4	13	Open	Natural	Sed	2.34
4	13	Open	Natural	Sed	1.62
4	13	Open	Natural	Sed	2.72
4	13	Open	Natural	Sed	2.13
4	13	Open	Natural	Sed	1.72
4	13	Open	Natural	Sed	2.78
4	13	Open	Natural	Sed	1.18
4	13	Open	Natural	Sed	0.50
4	13	Open	Natural	Sed	4.28
4	13	Open	Natural	Sed	2.05
4	14	Open	Natural	Sed	1.17
4	14	Open	Natural	Sed	1.80
4	14	Open	Natural	Sed	1.48
4	14	Open	Natural	Sed	10.30
4	14	Open	Natural	Sed	3.12
4	14	Open	Natural	Sed	4.06
4	14	Open	Natural	Sed	2.43
4	14	Open	Natural	Sed	6.77
4	14	Open	Natural	Sed	1.07
4	14	Open	Natural	Sed	1.65
4	14	Open	Natural	Sed	4.02
4	14	Open	Natural	Sed	1.88
4	14	Open	Natural	Sed	0.00
4	14	Open	Natural	Sed	2.68
4	14	Open	Natural	Sed	4.16
4	14	Open	Natural	Sed	1.79
4	14	Open	Natural	Sed	1.81
4	14	Open	Natural	Sed	2.08
4	14	Open	Natural	Sed	2.50
4	14	Open	Natural	Sed	1.50
4	15	Open	Natural	Sed	2.20
4	15	Open	Natural	Sed	1.09
4	15	Open	Natural	Sed	1.46
4	15	Open	Natural	Sed	0.59
4	15	Open	Natural	Sed	1.17
4	15	Open	Natural	Sed	0.86
4	15	Open	Natural	Sed	0.94
4	15	Open	Natural	Sed	2.62
4	15	Open	Natural	Sed	1.11
4	15	Open	Natural	Sed	0.56
4	15	Open	Natural	Sed	2.48
4	15	Open	Natural	Sed	1.97
4	15	Open	Natural	Sed	2.43
4	15	Open	Natural	Sed	1.21
4	15	Open	Natural	Sed	1.03
4	15	Open	Natural	Sed	1.71
4	15	Open	Natural	Sed	2.43
4	15	Open	Natural	Sed	1.54
4	15	Open	Natural	Sed	1.07

4	15 Open	Natural	Sed	3.32
4	16 Open	Addition	Sed	1.21
4	16 Open	Addition	Sed	3.03
4	16 Open	Addition	Sed	3.16
4	16 Open	Addition	Sed	1.22
4	16 Open	Addition	Sed	2.36
4	16 Open	Addition	Sed	1.57
4	16 Open	Addition	Sed	1.49
4	16 Open	Addition	Sed	3.96
4	16 Open	Addition	Sed	1.60
4	16 Open	Addition	Sed	0.62
4	16 Open	Addition	Sed	1.36
4	16 Open	Addition	Sed	1.73
4	16 Open	Addition	Sed	2.11
4	16 Open	Addition	Sed	1.72
4	16 Open	Addition	Sed	2.36
4	16 Open	Addition	Sed	2.44
4	16 Open	Addition	Sed	1.22
4	16 Open	Addition	Sed	1.51
4	16 Open	Addition	Sed	3.22
4	16 Open	Addition	Sed	4.59
4	17 Open	Addition	Sed	1.52
4	17 Open	Addition	Sed	1.07
4	17 Open	Addition	Sed	6.30
4	17 Open	Addition	Sed	1.47
4	17 Open	Addition	Sed	1.09
4	17 Open	Addition	Sed	0.98
4	17 Open	Addition	Sed	2.89
4	17 Open	Addition	Sed	1.79
4	17 Open	Addition	Sed	2.47
4	17 Open	Addition	Sed	6.22
4	17 Open	Addition	Sed	1.95
4	17 Open	Addition	Sed	2.14
4	17 Open	Addition	Sed	2.91
4	17 Open	Addition	Sed	2.73
4	17 Open	Addition	Sed	3.91
4	17 Open	Addition	Sed	1.61
4	17 Open	Addition	Sed	1.23
4	17 Open	Addition	Sed	1.72
4	17 Open	Addition	Sed	1.47
4	17 Open	Addition	Sed	2.15
4	18 Open	Addition	Sed	2.04
4	18 Open	Addition	Sed	2.29
4	18 Open	Addition	Sed	8.62
4	18 Open	Addition	Sed	1.07
4	18 Open	Addition	Sed	1.06
4	18 Open	Addition	Sed	11.44
4	18 Open	Addition	Sed	1.36
4	18 Open	Addition	Sed	1.04
4	18 Open	Addition	Sed	1.77
4	18 Open	Addition	Sed	6.44
4	18 Open	Addition	Sed	2.11
4	18 Open	Addition	Sed	2.82

4	18	Open	Addition	Sed	1.21
4	18	Open	Addition	Sed	4.64
4	18	Open	Addition	Sed	1.69
4	18	Open	Addition	Sed	1.25
4	18	Open	Addition	Sed	2.60
4	18	Open	Addition	Sed	2.48
4	18	Open	Addition	Sed	6.32
4	18	Open	Addition	Sed	2.55
4	19	Cage	Natural	Sed	1.10
4	19	Cage	Natural	Sed	1.30
4	19	Cage	Natural	Sed	1.53
4	19	Cage	Natural	Sed	1.98
4	19	Cage	Natural	Sed	1.27
4	19	Cage	Natural	Sed	4.47
4	19	Cage	Natural	Sed	1.79
4	19	Cage	Natural	Sed	1.78
4	19	Cage	Natural	Sed	9.34
4	19	Cage	Natural	Sed	0.80
4	19	Cage	Natural	Sed	2.18
4	19	Cage	Natural	Sed	0.65
4	19	Cage	Natural	Sed	1.97
4	19	Cage	Natural	Sed	4.30
4	19	Cage	Natural	Sed	3.25
4	19	Cage	Natural	Sed	5.04
4	19	Cage	Natural	Sed	0.59
4	19	Cage	Natural	Sed	2.88
4	19	Cage	Natural	Sed	0.00
4	19	Cage	Natural	Sed	22.20
4	20	Cage	Natural	Sed	2.90
4	20	Cage	Natural	Sed	4.88
4	20	Cage	Natural	Sed	8.89
4	20	Cage	Natural	Sed	0.00
4	20	Cage	Natural	Sed	2.62
4	20	Cage	Natural	Sed	1.87
4	20	Cage	Natural	Sed	0.00
4	20	Cage	Natural	Sed	4.84
4	20	Cage	Natural	Sed	1.54
4	20	Cage	Natural	Sed	1.75
4	20	Cage	Natural	Sed	5.02
4	20	Cage	Natural	Sed	3.86
4	20	Cage	Natural	Sed	4.91
4	20	Cage	Natural	Sed	3.06
4	20	Cage	Natural	Sed	5.23
4	20	Cage	Natural	Sed	2.49
4	20	Cage	Natural	Sed	5.04
4	20	Cage	Natural	Sed	3.78
4	20	Cage	Natural	Sed	0.58
4	20	Cage	Natural	Sed	1.43
4	21	Cage	Natural	Sed	3.11
4	21	Cage	Natural	Sed	3.60
4	21	Cage	Natural	Sed	0.71
4	21	Cage	Natural	Sed	1.53
4	21	Cage	Natural	Sed	4.34

4	21	Cage	Natural	Sed	0.00
4	21	Cage	Natural	Sed	1.39
4	21	Cage	Natural	Sed	3.82
4	21	Cage	Natural	Sed	0.59
4	21	Cage	Natural	Sed	1.40
4	21	Cage	Natural	Sed	5.40
4	21	Cage	Natural	Sed	2.99
4	21	Cage	Natural	Sed	1.89
4	21	Cage	Natural	Sed	1.53
4	21	Cage	Natural	Sed	1.01
4	21	Cage	Natural	Sed	3.05
4	21	Cage	Natural	Sed	1.52
4	21	Cage	Natural	Sed	4.22
4	21	Cage	Natural	Sed	2.69
4	21	Cage	Natural	Sed	1.37
4	22	Cage	Addition	Sed	0.00
4	22	Cage	Addition	Sed	0.56
4	22	Cage	Addition	Sed	10.88
4	22	Cage	Addition	Sed	8.71
4	22	Cage	Addition	Sed	3.08
4	22	Cage	Addition	Sed	3.80
4	22	Cage	Addition	Sed	1.65
4	22	Cage	Addition	Sed	1.88
4	22	Cage	Addition	Sed	1.74
4	22	Cage	Addition	Sed	1.25
4	22	Cage	Addition	Sed	10.91
4	22	Cage	Addition	Sed	4.01
4	22	Cage	Addition	Sed	5.59
4	22	Cage	Addition	Sed	8.42
4	22	Cage	Addition	Sed	2.97
4	22	Cage	Addition	Sed	0.37
4	22	Cage	Addition	Sed	5.22
4	22	Cage	Addition	Sed	0.35
4	22	Cage	Addition	Sed	1.77
4	22	Cage	Addition	Sed	1.33
4	23	Cage	Addition	Sed	1.82
4	23	Cage	Addition	Sed	1.70
4	23	Cage	Addition	Sed	7.32
4	23	Cage	Addition	Sed	0.97
4	23	Cage	Addition	Sed	4.27
4	23	Cage	Addition	Sed	1.75
4	23	Cage	Addition	Sed	0.00
4	23	Cage	Addition	Sed	1.62
4	23	Cage	Addition	Sed	3.14
4	23	Cage	Addition	Sed	7.53
4	23	Cage	Addition	Sed	0.67
4	23	Cage	Addition	Sed	2.50
4	23	Cage	Addition	Sed	0.00
4	23	Cage	Addition	Sed	0.67
4	23	Cage	Addition	Sed	1.61
4	23	Cage	Addition	Sed	2.51
4	23	Cage	Addition	Sed	1.66
4	23	Cage	Addition	Sed	0.00

4	23 Cage	Addition	Sed	3.63
4	23 Cage	Addition	Sed	7.10
4	24 Cage	Addition	Sed	3.27
4	24 Cage	Addition	Sed	2.83
4	24 Cage	Addition	Sed	5.46
4	24 Cage	Addition	Sed	2.55
4	24 Cage	Addition	Sed	5.00
4	24 Cage	Addition	Sed	5.03
4	24 Cage	Addition	Sed	2.79
4	24 Cage	Addition	Sed	0.92
4	24 Cage	Addition	Sed	0.00
4	24 Cage	Addition	Sed	4.42
4	24 Cage	Addition	Sed	7.81
4	24 Cage	Addition	Sed	5.76
4	24 Cage	Addition	Sed	1.30
4	24 Cage	Addition	Sed	2.37
4	24 Cage	Addition	Sed	2.61
4	24 Cage	Addition	Sed	1.93
4	24 Cage	Addition	Sed	0.54
4	24 Cage	Addition	Sed	1.25
4	24 Cage	Addition	Sed	3.68
4	24 Cage	Addition	Sed	1.47
4	25 Half	Natural	Sed	3.02
4	25 Half	Natural	Sed	4.41
4	25 Half	Natural	Sed	1.24
4	25 Half	Natural	Sed	5.41
4	25 Half	Natural	Sed	2.50
4	25 Half	Natural	Sed	5.23
4	25 Half	Natural	Sed	2.62
4	25 Half	Natural	Sed	2.31
4	25 Half	Natural	Sed	10.08
4	25 Half	Natural	Sed	10.81
4	25 Half	Natural	Sed	2.47
4	25 Half	Natural	Sed	4.54
4	25 Half	Natural	Sed	1.85
4	25 Half	Natural	Sed	3.23
4	25 Half	Natural	Sed	2.20
4	25 Half	Natural	Sed	0.74
4	25 Half	Natural	Sed	2.33
4	25 Half	Natural	Sed	2.17
4	25 Half	Natural	Sed	2.52
4	25 Half	Natural	Sed	1.84
4	26 Half	Natural	Sed	1.80
4	26 Half	Natural	Sed	2.38
4	26 Half	Natural	Sed	15.28
4	26 Half	Natural	Sed	3.00
4	26 Half	Natural	Sed	0.00
4	26 Half	Natural	Sed	1.70
4	26 Half	Natural	Sed	1.80
4	26 Half	Natural	Sed	2.18
4	26 Half	Natural	Sed	2.69
4	26 Half	Natural	Sed	2.64
4	26 Half	Natural	Sed	1.97

4	26	Half	Natural	Sed	1.16
4	26	Half	Natural	Sed	2.04
4	26	Half	Natural	Sed	4.50
4	26	Half	Natural	Sed	1.79
4	26	Half	Natural	Sed	1.88
4	26	Half	Natural	Sed	2.35
4	26	Half	Natural	Sed	3.77
4	26	Half	Natural	Sed	1.57
4	26	Half	Natural	Sed	3.61
4	27	Half	Natural	Sed	2.02
4	27	Half	Natural	Sed	1.84
4	27	Half	Natural	Sed	1.79
4	27	Half	Natural	Sed	2.59
4	27	Half	Natural	Sed	1.89
4	27	Half	Natural	Sed	1.94
4	27	Half	Natural	Sed	2.17
4	27	Half	Natural	Sed	5.58
4	27	Half	Natural	Sed	2.25
4	27	Half	Natural	Sed	2.64
4	27	Half	Natural	Sed	1.50
4	27	Half	Natural	Sed	1.90
4	27	Half	Natural	Sed	2.30
4	27	Half	Natural	Sed	3.21
4	27	Half	Natural	Sed	0.86
4	27	Half	Natural	Sed	1.62
4	27	Half	Natural	Sed	2.60
4	27	Half	Natural	Sed	2.61
4	27	Half	Natural	Sed	3.32
4	27	Half	Natural	Sed	2.21
4	28	Half	Addition	Sed	1.83
4	28	Half	Addition	Sed	2.55
4	28	Half	Addition	Sed	2.80
4	28	Half	Addition	Sed	1.37
4	28	Half	Addition	Sed	2.32
4	28	Half	Addition	Sed	2.61
4	28	Half	Addition	Sed	1.60
4	28	Half	Addition	Sed	1.93
4	28	Half	Addition	Sed	0.00
4	28	Half	Addition	Sed	1.29
4	28	Half	Addition	Sed	0.48
4	28	Half	Addition	Sed	1.30
4	28	Half	Addition	Sed	0.00
4	28	Half	Addition	Sed	0.70
4	28	Half	Addition	Sed	1.66
4	28	Half	Addition	Sed	2.60
4	28	Half	Addition	Sed	1.73
4	28	Half	Addition	Sed	2.19
4	28	Half	Addition	Sed	0.00
4	28	Half	Addition	Sed	0.00
4	29	Half	Addition	Sed	0.00
4	29	Half	Addition	Sed	0.00
4	29	Half	Addition	Sed	0.00
4	29	Half	Addition	Sed	2.06

4	29	Half	Addition	Sed	4.51
4	29	Half	Addition	Sed	3.11
4	29	Half	Addition	Sed	1.46
4	29	Half	Addition	Sed	0.00
4	29	Half	Addition	Sed	1.54
4	29	Half	Addition	Sed	0.00
4	29	Half	Addition	Sed	3.78
4	29	Half	Addition	Sed	1.14
4	29	Half	Addition	Sed	0.86
4	29	Half	Addition	Sed	2.35
4	29	Half	Addition	Sed	1.95
4	29	Half	Addition	Sed	0.83
4	29	Half	Addition	Sed	5.21
4	29	Half	Addition	Sed	0.91
4	29	Half	Addition	Sed	2.10
4	29	Half	Addition	Sed	4.03
4	30	Half	Addition	Sed	2.42
4	30	Half	Addition	Sed	6.77
4	30	Half	Addition	Sed	2.32
4	30	Half	Addition	Sed	0.70
4	30	Half	Addition	Sed	1.66
4	30	Half	Addition	Sed	1.75
4	30	Half	Addition	Sed	7.13
4	30	Half	Addition	Sed	2.09
4	30	Half	Addition	Sed	1.67
4	30	Half	Addition	Sed	1.68
4	30	Half	Addition	Sed	0.00
4	30	Half	Addition	Sed	0.67
4	30	Half	Addition	Sed	2.39
4	30	Half	Addition	Sed	2.49
4	30	Half	Addition	Sed	4.31
4	30	Half	Addition	Sed	2.13
4	30	Half	Addition	Sed	3.93
4	30	Half	Addition	Sed	1.79
4	30	Half	Addition	Sed	2.34
4	30	Half	Addition	Sed	3.52
4	31	Open	Natural	Sed	1.67
4	31	Open	Natural	Sed	2.23
4	31	Open	Natural	Sed	2.29
4	31	Open	Natural	Sed	2.41
4	31	Open	Natural	Sed	1.87
4	31	Open	Natural	Sed	2.18
4	31	Open	Natural	Sed	1.77
4	31	Open	Natural	Sed	6.98
4	31	Open	Natural	Sed	1.52
4	31	Open	Natural	Sed	1.11
4	31	Open	Natural	Sed	0.00
4	31	Open	Natural	Sed	2.50
4	31	Open	Natural	Sed	2.48
4	31	Open	Natural	Sed	1.95
4	31	Open	Natural	Sed	2.29
4	31	Open	Natural	Sed	2.05
4	31	Open	Natural	Sed	2.19

4	31 Open	Natural	Sed	3.27
4	31 Open	Natural	Sed	1.12
4	31 Open	Natural	Sed	2.99
4	32 Open	Natural	Sed	2.78
4	32 Open	Natural	Sed	2.88
4	32 Open	Natural	Sed	2.85
4	32 Open	Natural	Sed	1.39
4	32 Open	Natural	Sed	1.84
4	32 Open	Natural	Sed	0.00
4	32 Open	Natural	Sed	2.18
4	32 Open	Natural	Sed	2.18
4	32 Open	Natural	Sed	1.22
4	32 Open	Natural	Sed	1.06
4	32 Open	Natural	Sed	2.11
4	32 Open	Natural	Sed	1.82
4	32 Open	Natural	Sed	2.14
4	32 Open	Natural	Sed	2.56
4	32 Open	Natural	Sed	1.21
4	32 Open	Natural	Sed	1.90
4	32 Open	Natural	Sed	2.43
4	32 Open	Natural	Sed	4.53
4	32 Open	Natural	Sed	1.58
4	32 Open	Natural	Sed	2.72
4	33 Open	Natural	Sed	2.38
4	33 Open	Natural	Sed	1.68
4	33 Open	Natural	Sed	1.05
4	33 Open	Natural	Sed	0.00
4	33 Open	Natural	Sed	3.29
4	33 Open	Natural	Sed	0.00
4	33 Open	Natural	Sed	2.38
4	33 Open	Natural	Sed	2.22
4	33 Open	Natural	Sed	1.69
4	33 Open	Natural	Sed	3.74
4	33 Open	Natural	Sed	1.28
4	33 Open	Natural	Sed	2.43
4	33 Open	Natural	Sed	3.78
4	33 Open	Natural	Sed	0.67
4	33 Open	Natural	Sed	1.48
4	33 Open	Natural	Sed	1.60
4	33 Open	Natural	Sed	2.08
4	33 Open	Natural	Sed	2.23
4	33 Open	Natural	Sed	2.19
4	33 Open	Natural	Sed	16.87
4	34 Open	Addition	Sed	9.24
4	34 Open	Addition	Sed	3.27
4	34 Open	Addition	Sed	6.34
4	34 Open	Addition	Sed	2.84
4	34 Open	Addition	Sed	4.01
4	34 Open	Addition	Sed	4.99
4	34 Open	Addition	Sed	5.31
4	34 Open	Addition	Sed	9.61
4	34 Open	Addition	Sed	2.46
4	34 Open	Addition	Sed	12.46

4	34	Open	Addition	Sed	6.85
4	34	Open	Addition	Sed	10.67
4	34	Open	Addition	Sed	4.95
4	34	Open	Addition	Sed	24.27
4	34	Open	Addition	Sed	23.25
4	34	Open	Addition	Sed	3.18
4	34	Open	Addition	Sed	3.97
4	34	Open	Addition	Sed	3.94
4	34	Open	Addition	Sed	5.54
4	34	Open	Addition	Sed	4.77
4	35	Open	Addition	Sed	3.59
4	35	Open	Addition	Sed	2.16
4	35	Open	Addition	Sed	4.46
4	35	Open	Addition	Sed	3.47
4	35	Open	Addition	Sed	4.00
4	35	Open	Addition	Sed	3.81
4	35	Open	Addition	Sed	3.61
4	35	Open	Addition	Sed	2.42
4	35	Open	Addition	Sed	3.44
4	35	Open	Addition	Sed	6.69
4	35	Open	Addition	Sed	4.85
4	35	Open	Addition	Sed	5.89
4	35	Open	Addition	Sed	10.16
4	35	Open	Addition	Sed	3.74
4	35	Open	Addition	Sed	3.21
4	35	Open	Addition	Sed	2.34
4	35	Open	Addition	Sed	7.84
4	35	Open	Addition	Sed	40.70
4	35	Open	Addition	Sed	34.13
4	35	Open	Addition	Sed	2.55
4	36	Open	Addition	Sed	1.98
4	36	Open	Addition	Sed	2.93
4	36	Open	Addition	Sed	2.08
4	36	Open	Addition	Sed	5.73
4	36	Open	Addition	Sed	2.25
4	36	Open	Addition	Sed	4.64
4	36	Open	Addition	Sed	1.91
4	36	Open	Addition	Sed	3.59
4	36	Open	Addition	Sed	3.51
4	36	Open	Addition	Sed	2.08
4	36	Open	Addition	Sed	3.55
4	36	Open	Addition	Sed	0.00
4	36	Open	Addition	Sed	2.18
4	36	Open	Addition	Sed	2.52
4	36	Open	Addition	Sed	3.46
4	36	Open	Addition	Sed	4.53
4	36	Open	Addition	Sed	2.50
4	36	Open	Addition	Sed	2.60
4	36	Open	Addition	Sed	4.45
4	36	Open	Addition	Sed	18.96
5	1	Full	Natural	Sed	0.88
5	1	Full	Natural	Sed	6.61
5	1	Full	Natural	Sed	11.55

5	1	Full	Natural	Sed	0.71
5	1	Full	Natural	Sed	2.45
5	1	Full	Natural	Sed	11.43
5	1	Full	Natural	Sed	2.95
5	1	Full	Natural	Sed	1.79
5	1	Full	Natural	Sed	4.40
5	1	Full	Natural	Sed	1.91
5	1	Full	Natural	Sed	17.32
5	1	Full	Natural	Sed	1.16
5	1	Full	Natural	Sed	1.04
5	1	Full	Natural	Sed	2.24
5	1	Full	Natural	Sed	5.04
5	1	Full	Natural	Sed	1.83
5	1	Full	Natural	Sed	3.05
5	1	Full	Natural	Sed	2.00
5	1	Full	Natural	Sed	2.84
5	1	Full	Natural	Sed	0.70
5	2	Full	Natural	Sed	2.53
5	2	Full	Natural	Sed	1.84
5	2	Full	Natural	Sed	3.47
5	2	Full	Natural	Sed	1.33
5	2	Full	Natural	Sed	4.46
5	2	Full	Natural	Sed	8.39
5	2	Full	Natural	Sed	3.06
5	2	Full	Natural	Sed	2.88
5	2	Full	Natural	Sed	2.46
5	2	Full	Natural	Sed	1.44
5	2	Full	Natural	Sed	2.92
5	2	Full	Natural	Sed	2.78
5	2	Full	Natural	Sed	3.02
5	2	Full	Natural	Sed	4.48
5	2	Full	Natural	Sed	3.34
5	2	Full	Natural	Sed	1.32
5	2	Full	Natural	Sed	2.61
5	2	Full	Natural	Sed	2.54
5	2	Full	Natural	Sed	2.89
5	2	Full	Natural	Sed	1.61
5	3	Full	Natural	Sed	8.41
5	3	Full	Natural	Sed	3.00
5	3	Full	Natural	Sed	6.73
5	3	Full	Natural	Sed	2.17
5	3	Full	Natural	Sed	4.15
5	3	Full	Natural	Sed	3.41
5	3	Full	Natural	Sed	1.97
5	3	Full	Natural	Sed	5.67
5	3	Full	Natural	Sed	7.47
5	3	Full	Natural	Sed	2.05
5	3	Full	Natural	Sed	1.73
5	3	Full	Natural	Sed	4.34
5	3	Full	Natural	Sed	1.52
5	3	Full	Natural	Sed	6.84
5	3	Full	Natural	Sed	2.29
5	3	Full	Natural	Sed	2.28

5	3	Full	Natural	Sed	3.27
5	3	Full	Natural	Sed	1.89
5	3	Full	Natural	Sed	2.37
5	3	Full	Natural	Sed	4.48
5	4	Full	Addition	Sed	2.01
5	4	Full	Addition	Sed	2.18
5	4	Full	Addition	Sed	2.09
5	4	Full	Addition	Sed	2.09
5	4	Full	Addition	Sed	7.24
5	4	Full	Addition	Sed	1.57
5	4	Full	Addition	Sed	2.20
5	4	Full	Addition	Sed	3.21
5	4	Full	Addition	Sed	4.11
5	4	Full	Addition	Sed	2.37
5	4	Full	Addition	Sed	1.93
5	4	Full	Addition	Sed	7.18
5	4	Full	Addition	Sed	2.53
5	4	Full	Addition	Sed	5.81
5	4	Full	Addition	Sed	2.17
5	4	Full	Addition	Sed	0.73
5	4	Full	Addition	Sed	1.39
5	4	Full	Addition	Sed	3.90
5	4	Full	Addition	Sed	4.70
5	4	Full	Addition	Sed	5.22
5	5	Full	Addition	Sed	2.48
5	5	Full	Addition	Sed	1.44
5	5	Full	Addition	Sed	3.82
5	5	Full	Addition	Sed	3.60
5	5	Full	Addition	Sed	2.96
5	5	Full	Addition	Sed	4.96
5	5	Full	Addition	Sed	2.58
5	5	Full	Addition	Sed	4.30
5	5	Full	Addition	Sed	2.98
5	5	Full	Addition	Sed	6.78
5	5	Full	Addition	Sed	2.50
5	5	Full	Addition	Sed	3.29
5	5	Full	Addition	Sed	1.34
5	5	Full	Addition	Sed	3.38
5	5	Full	Addition	Sed	1.03
5	5	Full	Addition	Sed	2.11
5	5	Full	Addition	Sed	2.94
5	5	Full	Addition	Sed	3.46
5	5	Full	Addition	Sed	2.34
5	5	Full	Addition	Sed	1.74
5	6	Full	Addition	Sed	2.69
5	6	Full	Addition	Sed	1.77
5	6	Full	Addition	Sed	4.88
5	6	Full	Addition	Sed	4.53
5	6	Full	Addition	Sed	1.25
5	6	Full	Addition	Sed	1.18
5	6	Full	Addition	Sed	2.21
5	6	Full	Addition	Sed	2.17
5	6	Full	Addition	Sed	3.06

5	6	Full	Addition	Sed	1.84
5	6	Full	Addition	Sed	1.59
5	6	Full	Addition	Sed	2.72
5	6	Full	Addition	Sed	2.79
5	6	Full	Addition	Sed	1.75
5	6	Full	Addition	Sed	3.64
5	6	Full	Addition	Sed	2.99
5	6	Full	Addition	Sed	1.75
5	6	Full	Addition	Sed	3.31
5	6	Full	Addition	Sed	5.62
5	6	Full	Addition	Sed	3.14
5	7	Half	Natural	Sed	2.16
5	7	Half	Natural	Sed	1.60
5	7	Half	Natural	Sed	1.95
5	7	Half	Natural	Sed	0.83
5	7	Half	Natural	Sed	0.70
5	7	Half	Natural	Sed	1.60
5	7	Half	Natural	Sed	1.22
5	7	Half	Natural	Sed	3.54
5	7	Half	Natural	Sed	2.66
5	7	Half	Natural	Sed	2.85
5	7	Half	Natural	Sed	4.41
5	7	Half	Natural	Sed	3.15
5	7	Half	Natural	Sed	2.72
5	7	Half	Natural	Sed	2.10
5	7	Half	Natural	Sed	3.06
5	7	Half	Natural	Sed	1.69
5	7	Half	Natural	Sed	3.10
5	7	Half	Natural	Sed	2.68
5	7	Half	Natural	Sed	2.46
5	7	Half	Natural	Sed	3.05
5	8	Half	Natural	Sed	1.23
5	8	Half	Natural	Sed	1.35
5	8	Half	Natural	Sed	3.19
5	8	Half	Natural	Sed	2.12
5	8	Half	Natural	Sed	0.68
5	8	Half	Natural	Sed	2.13
5	8	Half	Natural	Sed	1.83
5	8	Half	Natural	Sed	8.04
5	8	Half	Natural	Sed	2.30
5	8	Half	Natural	Sed	2.88
5	8	Half	Natural	Sed	1.53
5	8	Half	Natural	Sed	1.77
5	8	Half	Natural	Sed	1.82
5	8	Half	Natural	Sed	0.70
5	8	Half	Natural	Sed	0.97
5	8	Half	Natural	Sed	1.49
5	8	Half	Natural	Sed	1.46
5	8	Half	Natural	Sed	2.17
5	8	Half	Natural	Sed	1.27
5	8	Half	Natural	Sed	0.54
5	9	Half	Natural	Sed	1.36
5	9	Half	Natural	Sed	2.40

5	9	Half	Natural	Sed	1.44
5	9	Half	Natural	Sed	4.85
5	9	Half	Natural	Sed	1.17
5	9	Half	Natural	Sed	3.07
5	9	Half	Natural	Sed	2.26
5	9	Half	Natural	Sed	2.26
5	9	Half	Natural	Sed	4.50
5	9	Half	Natural	Sed	0.96
5	9	Half	Natural	Sed	1.78
5	9	Half	Natural	Sed	2.54
5	9	Half	Natural	Sed	3.56
5	9	Half	Natural	Sed	1.70
5	9	Half	Natural	Sed	4.06
5	9	Half	Natural	Sed	1.39
5	9	Half	Natural	Sed	6.45
5	9	Half	Natural	Sed	3.70
5	9	Half	Natural	Sed	1.27
5	9	Half	Natural	Sed	1.58
5	10	Half	Addition	Sed	3.12
5	10	Half	Addition	Sed	1.27
5	10	Half	Addition	Sed	1.65
5	10	Half	Addition	Sed	2.67
5	10	Half	Addition	Sed	0.43
5	10	Half	Addition	Sed	2.70
5	10	Half	Addition	Sed	2.68
5	10	Half	Addition	Sed	2.00
5	10	Half	Addition	Sed	3.42
5	10	Half	Addition	Sed	1.92
5	10	Half	Addition	Sed	1.29
5	10	Half	Addition	Sed	0.92
5	10	Half	Addition	Sed	0.92
5	10	Half	Addition	Sed	2.28
5	10	Half	Addition	Sed	1.78
5	10	Half	Addition	Sed	1.86
5	10	Half	Addition	Sed	1.53
5	10	Half	Addition	Sed	1.43
5	10	Half	Addition	Sed	3.36
5	10	Half	Addition	Sed	1.17
5	11	Half	Addition	Sed	2.88
5	11	Half	Addition	Sed	2.71
5	11	Half	Addition	Sed	2.56
5	11	Half	Addition	Sed	1.85
5	11	Half	Addition	Sed	1.95
5	11	Half	Addition	Sed	1.43
5	11	Half	Addition	Sed	1.87
5	11	Half	Addition	Sed	1.91
5	11	Half	Addition	Sed	2.98
5	11	Half	Addition	Sed	1.75
5	11	Half	Addition	Sed	3.82
5	11	Half	Addition	Sed	3.30
5	11	Half	Addition	Sed	1.05
5	11	Half	Addition	Sed	0.55
5	11	Half	Addition	Sed	1.75

5	11	Half	Addition	Sed	1.14
5	11	Half	Addition	Sed	1.38
5	11	Half	Addition	Sed	0.00
5	11	Half	Addition	Sed	1.86
5	11	Half	Addition	Sed	17.42
5	12	Half	Addition	Sed	2.86
5	12	Half	Addition	Sed	5.42
5	12	Half	Addition	Sed	2.83
5	12	Half	Addition	Sed	3.43
5	12	Half	Addition	Sed	2.90
5	12	Half	Addition	Sed	3.69
5	12	Half	Addition	Sed	2.45
5	12	Half	Addition	Sed	3.07
5	12	Half	Addition	Sed	2.78
5	12	Half	Addition	Sed	3.02
5	12	Half	Addition	Sed	1.71
5	12	Half	Addition	Sed	4.37
5	12	Half	Addition	Sed	1.89
5	12	Half	Addition	Sed	7.96
5	12	Half	Addition	Sed	2.52
5	12	Half	Addition	Sed	3.11
5	12	Half	Addition	Sed	4.48
5	12	Half	Addition	Sed	1.62
5	12	Half	Addition	Sed	3.05
5	12	Half	Addition	Sed	4.10
5	13	Open	Natural	Sed	7.89
5	13	Open	Natural	Sed	2.08
5	13	Open	Natural	Sed	0.95
5	13	Open	Natural	Sed	3.77
5	13	Open	Natural	Sed	1.81
5	13	Open	Natural	Sed	3.08
5	13	Open	Natural	Sed	1.81
5	13	Open	Natural	Sed	1.53
5	13	Open	Natural	Sed	1.80
5	13	Open	Natural	Sed	0.62
5	13	Open	Natural	Sed	1.80
5	13	Open	Natural	Sed	2.22
5	13	Open	Natural	Sed	3.48
5	13	Open	Natural	Sed	4.16
5	13	Open	Natural	Sed	1.41
5	13	Open	Natural	Sed	0.95
5	13	Open	Natural	Sed	1.44
5	13	Open	Natural	Sed	2.48
5	13	Open	Natural	Sed	1.64
5	13	Open	Natural	Sed	1.10
5	14	Open	Natural	Sed	3.14
5	14	Open	Natural	Sed	1.79
5	14	Open	Natural	Sed	2.32
5	14	Open	Natural	Sed	2.09
5	14	Open	Natural	Sed	1.89
5	14	Open	Natural	Sed	8.49
5	14	Open	Natural	Sed	1.15
5	14	Open	Natural	Sed	2.39

5	14	Open	Natural	Sed	3.01
5	14	Open	Natural	Sed	1.95
5	14	Open	Natural	Sed	0.65
5	14	Open	Natural	Sed	3.53
5	14	Open	Natural	Sed	3.48
5	14	Open	Natural	Sed	1.78
5	14	Open	Natural	Sed	0.91
5	14	Open	Natural	Sed	0.67
5	14	Open	Natural	Sed	1.80
5	14	Open	Natural	Sed	1.78
5	14	Open	Natural	Sed	0.00
5	14	Open	Natural	Sed	2.38
5	15	Open	Natural	Sed	0.00
5	15	Open	Natural	Sed	5.07
5	15	Open	Natural	Sed	1.36
5	15	Open	Natural	Sed	1.48
5	15	Open	Natural	Sed	2.64
5	15	Open	Natural	Sed	1.26
5	15	Open	Natural	Sed	0.90
5	15	Open	Natural	Sed	0.95
5	15	Open	Natural	Sed	1.14
5	15	Open	Natural	Sed	1.07
5	15	Open	Natural	Sed	1.33
5	15	Open	Natural	Sed	2.13
5	15	Open	Natural	Sed	1.82
5	15	Open	Natural	Sed	1.45
5	15	Open	Natural	Sed	1.46
5	15	Open	Natural	Sed	0.65
5	15	Open	Natural	Sed	6.09
5	15	Open	Natural	Sed	1.02
5	15	Open	Natural	Sed	1.79
5	15	Open	Natural	Sed	0.55
5	16	Open	Addition	Sed	2.10
5	16	Open	Addition	Sed	0.87
5	16	Open	Addition	Sed	0.97
5	16	Open	Addition	Sed	1.72
5	16	Open	Addition	Sed	1.81
5	16	Open	Addition	Sed	1.22
5	16	Open	Addition	Sed	2.11
5	16	Open	Addition	Sed	1.22
5	16	Open	Addition	Sed	1.67
5	16	Open	Addition	Sed	2.22
5	16	Open	Addition	Sed	1.95
5	16	Open	Addition	Sed	1.10
5	16	Open	Addition	Sed	0.53
5	16	Open	Addition	Sed	1.79
5	16	Open	Addition	Sed	3.49
5	16	Open	Addition	Sed	2.45
5	16	Open	Addition	Sed	1.67
5	16	Open	Addition	Sed	1.52
5	16	Open	Addition	Sed	2.12
5	16	Open	Addition	Sed	1.54
5	17	Open	Addition	Sed	1.33

5	17	Open	Addition	Sed	2.27
5	17	Open	Addition	Sed	5.44
5	17	Open	Addition	Sed	1.30
5	17	Open	Addition	Sed	1.24
5	17	Open	Addition	Sed	3.05
5	17	Open	Addition	Sed	2.16
5	17	Open	Addition	Sed	1.34
5	17	Open	Addition	Sed	1.39
5	17	Open	Addition	Sed	2.42
5	17	Open	Addition	Sed	1.78
5	17	Open	Addition	Sed	0.89
5	17	Open	Addition	Sed	1.84
5	17	Open	Addition	Sed	1.97
5	17	Open	Addition	Sed	5.73
5	17	Open	Addition	Sed	2.51
5	17	Open	Addition	Sed	2.38
5	17	Open	Addition	Sed	2.64
5	17	Open	Addition	Sed	2.09
5	17	Open	Addition	Sed	2.63
5	18	Open	Addition	Sed	1.20
5	18	Open	Addition	Sed	1.64
5	18	Open	Addition	Sed	1.95
5	18	Open	Addition	Sed	3.25
5	18	Open	Addition	Sed	1.96
5	18	Open	Addition	Sed	1.37
5	18	Open	Addition	Sed	1.14
5	18	Open	Addition	Sed	2.15
5	18	Open	Addition	Sed	2.15
5	18	Open	Addition	Sed	1.11
5	18	Open	Addition	Sed	5.14
5	18	Open	Addition	Sed	1.74
5	18	Open	Addition	Sed	2.43
5	18	Open	Addition	Sed	3.58
5	18	Open	Addition	Sed	1.77
5	18	Open	Addition	Sed	1.38
5	18	Open	Addition	Sed	1.35
5	18	Open	Addition	Sed	1.86
5	18	Open	Addition	Sed	3.85
5	18	Open	Addition	Sed	1.77
5	19	Cage	Natural	Sed	3.79
5	19	Cage	Natural	Sed	0.73
5	19	Cage	Natural	Sed	1.63
5	19	Cage	Natural	Sed	2.02
5	19	Cage	Natural	Sed	7.67
5	19	Cage	Natural	Sed	2.41
5	19	Cage	Natural	Sed	2.38
5	19	Cage	Natural	Sed	6.43
5	19	Cage	Natural	Sed	5.00
5	19	Cage	Natural	Sed	3.49
5	19	Cage	Natural	Sed	2.28
5	19	Cage	Natural	Sed	1.31
5	19	Cage	Natural	Sed	3.33
5	19	Cage	Natural	Sed	3.11

5	19	Cage	Natural	Sed	4.45
5	19	Cage	Natural	Sed	2.30
5	19	Cage	Natural	Sed	2.70
5	19	Cage	Natural	Sed	1.65
5	19	Cage	Natural	Sed	3.07
5	19	Cage	Natural	Sed	1.79
5	20	Cage	Natural	Sed	1.91
5	20	Cage	Natural	Sed	3.71
5	20	Cage	Natural	Sed	5.18
5	20	Cage	Natural	Sed	1.30
5	20	Cage	Natural	Sed	2.04
5	20	Cage	Natural	Sed	2.42
5	20	Cage	Natural	Sed	4.23
5	20	Cage	Natural	Sed	2.40
5	20	Cage	Natural	Sed	8.51
5	20	Cage	Natural	Sed	1.55
5	20	Cage	Natural	Sed	7.56
5	20	Cage	Natural	Sed	2.22
5	20	Cage	Natural	Sed	1.13
5	20	Cage	Natural	Sed	3.67
5	20	Cage	Natural	Sed	2.14
5	20	Cage	Natural	Sed	1.84
5	20	Cage	Natural	Sed	4.17
5	20	Cage	Natural	Sed	1.54
5	20	Cage	Natural	Sed	1.68
5	20	Cage	Natural	Sed	2.51
5	21	Cage	Natural	Sed	5.97
5	21	Cage	Natural	Sed	1.79
5	21	Cage	Natural	Sed	2.95
5	21	Cage	Natural	Sed	2.90
5	21	Cage	Natural	Sed	0.55
5	21	Cage	Natural	Sed	1.89
5	21	Cage	Natural	Sed	5.48
5	21	Cage	Natural	Sed	1.15
5	21	Cage	Natural	Sed	0.92
5	21	Cage	Natural	Sed	4.20
5	21	Cage	Natural	Sed	2.73
5	21	Cage	Natural	Sed	5.29
5	21	Cage	Natural	Sed	3.61
5	21	Cage	Natural	Sed	2.21
5	21	Cage	Natural	Sed	3.56
5	21	Cage	Natural	Sed	2.83
5	21	Cage	Natural	Sed	1.91
5	21	Cage	Natural	Sed	1.54
5	21	Cage	Natural	Sed	3.14
5	21	Cage	Natural	Sed	3.40
5	22	Cage	Addition	Sed	4.79
5	22	Cage	Addition	Sed	3.01
5	22	Cage	Addition	Sed	3.68
5	22	Cage	Addition	Sed	3.76
5	22	Cage	Addition	Sed	0.92
5	22	Cage	Addition	Sed	2.42
5	22	Cage	Addition	Sed	2.94

5	22	Cage	Addition	Sed	2.81
5	22	Cage	Addition	Sed	1.46
5	22	Cage	Addition	Sed	4.36
5	22	Cage	Addition	Sed	0.00
5	22	Cage	Addition	Sed	2.66
5	22	Cage	Addition	Sed	1.01
5	22	Cage	Addition	Sed	3.04
5	22	Cage	Addition	Sed	3.95
5	22	Cage	Addition	Sed	1.04
5	22	Cage	Addition	Sed	1.53
5	22	Cage	Addition	Sed	1.80
5	22	Cage	Addition	Sed	1.65
5	22	Cage	Addition	Sed	3.50
5	23	Cage	Addition	Sed	1.13
5	23	Cage	Addition	Sed	1.91
5	23	Cage	Addition	Sed	1.91
5	23	Cage	Addition	Sed	2.58
5	23	Cage	Addition	Sed	3.58
5	23	Cage	Addition	Sed	1.05
5	23	Cage	Addition	Sed	3.48
5	23	Cage	Addition	Sed	7.03
5	23	Cage	Addition	Sed	2.73
5	23	Cage	Addition	Sed	4.98
5	23	Cage	Addition	Sed	9.11
5	23	Cage	Addition	Sed	1.79
5	23	Cage	Addition	Sed	1.41
5	23	Cage	Addition	Sed	3.29
5	23	Cage	Addition	Sed	1.62
5	23	Cage	Addition	Sed	3.58
5	23	Cage	Addition	Sed	9.65
5	23	Cage	Addition	Sed	1.18
5	23	Cage	Addition	Sed	1.16
5	23	Cage	Addition	Sed	10.63
5	24	Cage	Addition	Sed	3.38
5	24	Cage	Addition	Sed	8.07
5	24	Cage	Addition	Sed	1.70
5	24	Cage	Addition	Sed	3.69
5	24	Cage	Addition	Sed	2.37
5	24	Cage	Addition	Sed	5.63
5	24	Cage	Addition	Sed	4.03
5	24	Cage	Addition	Sed	8.26
5	24	Cage	Addition	Sed	3.02
5	24	Cage	Addition	Sed	2.21
5	24	Cage	Addition	Sed	1.34
5	24	Cage	Addition	Sed	2.85
5	24	Cage	Addition	Sed	4.03
5	24	Cage	Addition	Sed	2.13
5	24	Cage	Addition	Sed	1.81
5	24	Cage	Addition	Sed	2.77
5	24	Cage	Addition	Sed	3.49
5	24	Cage	Addition	Sed	3.00
5	24	Cage	Addition	Sed	1.22
5	24	Cage	Addition	Sed	4.53

5	25	Half	Natural	Sed	1.31
5	25	Half	Natural	Sed	0.75
5	25	Half	Natural	Sed	1.93
5	25	Half	Natural	Sed	0.57
5	25	Half	Natural	Sed	1.30
5	25	Half	Natural	Sed	3.03
5	25	Half	Natural	Sed	1.57
5	25	Half	Natural	Sed	0.80
5	25	Half	Natural	Sed	3.07
5	25	Half	Natural	Sed	1.61
5	25	Half	Natural	Sed	16.57
5	25	Half	Natural	Sed	1.28
5	25	Half	Natural	Sed	3.66
5	25	Half	Natural	Sed	1.35
5	25	Half	Natural	Sed	2.71
5	25	Half	Natural	Sed	2.51
5	25	Half	Natural	Sed	1.55
5	25	Half	Natural	Sed	1.89
5	25	Half	Natural	Sed	1.22
5	25	Half	Natural	Sed	1.82
5	26	Half	Natural	Sed	2.62
5	26	Half	Natural	Sed	0.54
5	26	Half	Natural	Sed	0.00
5	26	Half	Natural	Sed	3.91
5	26	Half	Natural	Sed	2.42
5	26	Half	Natural	Sed	6.40
5	26	Half	Natural	Sed	0.00
5	26	Half	Natural	Sed	4.79
5	26	Half	Natural	Sed	1.78
5	26	Half	Natural	Sed	3.02
5	26	Half	Natural	Sed	1.61
5	26	Half	Natural	Sed	0.47
5	26	Half	Natural	Sed	1.28
5	26	Half	Natural	Sed	1.32
5	26	Half	Natural	Sed	0.67
5	26	Half	Natural	Sed	0.75
5	26	Half	Natural	Sed	0.00
5	26	Half	Natural	Sed	0.00
5	26	Half	Natural	Sed	1.02
5	26	Half	Natural	Sed	0.85
5	27	Half	Natural	Sed	1.50
5	27	Half	Natural	Sed	1.86
5	27	Half	Natural	Sed	0.00
5	27	Half	Natural	Sed	0.00
5	27	Half	Natural	Sed	2.44
5	27	Half	Natural	Sed	2.58
5	27	Half	Natural	Sed	1.68
5	27	Half	Natural	Sed	0.74
5	27	Half	Natural	Sed	1.10
5	27	Half	Natural	Sed	2.33
5	27	Half	Natural	Sed	0.98
5	27	Half	Natural	Sed	1.38
5	27	Half	Natural	Sed	1.65

5	27	Half	Natural	Sed	1.14
5	27	Half	Natural	Sed	0.64
5	27	Half	Natural	Sed	1.42
5	27	Half	Natural	Sed	2.19
5	27	Half	Natural	Sed	1.47
5	27	Half	Natural	Sed	2.14
5	27	Half	Natural	Sed	0.00
5	28	Half	Addition	Sed	2.31
5	28	Half	Addition	Sed	2.49
5	28	Half	Addition	Sed	1.01
5	28	Half	Addition	Sed	1.50
5	28	Half	Addition	Sed	2.94
5	28	Half	Addition	Sed	1.98
5	28	Half	Addition	Sed	1.73
5	28	Half	Addition	Sed	2.22
5	28	Half	Addition	Sed	1.83
5	28	Half	Addition	Sed	2.27
5	28	Half	Addition	Sed	0.00
5	28	Half	Addition	Sed	1.43
5	28	Half	Addition	Sed	0.80
5	28	Half	Addition	Sed	1.67
5	28	Half	Addition	Sed	0.00
5	28	Half	Addition	Sed	1.54
5	28	Half	Addition	Sed	1.82
5	28	Half	Addition	Sed	0.00
5	28	Half	Addition	Sed	2.55
5	28	Half	Addition	Sed	2.69
5	29	Half	Addition	Sed	2.17
5	29	Half	Addition	Sed	1.44
5	29	Half	Addition	Sed	1.96
5	29	Half	Addition	Sed	2.59
5	29	Half	Addition	Sed	2.29
5	29	Half	Addition	Sed	2.81
5	29	Half	Addition	Sed	2.63
5	29	Half	Addition	Sed	0.82
5	29	Half	Addition	Sed	1.98
5	29	Half	Addition	Sed	2.68
5	29	Half	Addition	Sed	2.42
5	29	Half	Addition	Sed	0.00
5	29	Half	Addition	Sed	0.00
5	29	Half	Addition	Sed	0.90
5	29	Half	Addition	Sed	0.83
5	29	Half	Addition	Sed	0.52
5	29	Half	Addition	Sed	2.52
5	29	Half	Addition	Sed	1.89
5	29	Half	Addition	Sed	1.87
5	29	Half	Addition	Sed	1.62
5	30	Half	Addition	Sed	2.53
5	30	Half	Addition	Sed	1.47
5	30	Half	Addition	Sed	1.48
5	30	Half	Addition	Sed	1.66
5	30	Half	Addition	Sed	0.80
5	30	Half	Addition	Sed	0.00

5	30	Half	Addition	Sed	9.47
5	30	Half	Addition	Sed	3.77
5	30	Half	Addition	Sed	7.85
5	30	Half	Addition	Sed	0.00
5	30	Half	Addition	Sed	1.80
5	30	Half	Addition	Sed	2.19
5	30	Half	Addition	Sed	3.39
5	30	Half	Addition	Sed	3.54
5	30	Half	Addition	Sed	1.43
5	30	Half	Addition	Sed	1.17
5	30	Half	Addition	Sed	2.19
5	30	Half	Addition	Sed	3.28
5	30	Half	Addition	Sed	2.78
5	30	Half	Addition	Sed	2.30
5	31	Open	Natural	Sed	1.00
5	31	Open	Natural	Sed	0.00
5	31	Open	Natural	Sed	2.09
5	31	Open	Natural	Sed	1.02
5	31	Open	Natural	Sed	2.69
5	31	Open	Natural	Sed	4.74
5	31	Open	Natural	Sed	0.00
5	31	Open	Natural	Sed	1.12
5	31	Open	Natural	Sed	1.50
5	31	Open	Natural	Sed	0.84
5	31	Open	Natural	Sed	0.81
5	31	Open	Natural	Sed	3.29
5	31	Open	Natural	Sed	0.90
5	31	Open	Natural	Sed	0.00
5	31	Open	Natural	Sed	1.21
5	31	Open	Natural	Sed	1.75
5	31	Open	Natural	Sed	1.65
5	31	Open	Natural	Sed	1.50
5	31	Open	Natural	Sed	1.67
5	31	Open	Natural	Sed	1.06
5	32	Open	Natural	Sed	3.75
5	32	Open	Natural	Sed	0.98
5	32	Open	Natural	Sed	2.09
5	32	Open	Natural	Sed	1.16
5	32	Open	Natural	Sed	1.68
5	32	Open	Natural	Sed	3.05
5	32	Open	Natural	Sed	1.57
5	32	Open	Natural	Sed	0.94
5	32	Open	Natural	Sed	2.86
5	32	Open	Natural	Sed	1.18
5	32	Open	Natural	Sed	1.31
5	32	Open	Natural	Sed	0.82
5	32	Open	Natural	Sed	0.99
5	32	Open	Natural	Sed	1.27
5	32	Open	Natural	Sed	1.78
5	32	Open	Natural	Sed	2.19
5	32	Open	Natural	Sed	0.00
5	32	Open	Natural	Sed	2.37
5	32	Open	Natural	Sed	1.48

5	32	Open	Natural	Sed	1.91
5	33	Open	Natural	Sed	0.77
5	33	Open	Natural	Sed	0.49
5	33	Open	Natural	Sed	1.67
5	33	Open	Natural	Sed	1.02
5	33	Open	Natural	Sed	1.13
5	33	Open	Natural	Sed	3.10
5	33	Open	Natural	Sed	1.73
5	33	Open	Natural	Sed	1.10
5	33	Open	Natural	Sed	1.30
5	33	Open	Natural	Sed	0.78
5	33	Open	Natural	Sed	0.73
5	33	Open	Natural	Sed	2.51
5	33	Open	Natural	Sed	0.00
5	33	Open	Natural	Sed	1.52
5	33	Open	Natural	Sed	0.00
5	33	Open	Natural	Sed	6.96
5	33	Open	Natural	Sed	1.20
5	33	Open	Natural	Sed	1.56
5	33	Open	Natural	Sed	3.83
5	33	Open	Natural	Sed	1.18
5	34	Open	Addition	Sed	3.21
5	34	Open	Addition	Sed	1.33
5	34	Open	Addition	Sed	1.84
5	34	Open	Addition	Sed	4.31
5	34	Open	Addition	Sed	10.50
5	34	Open	Addition	Sed	4.69
5	34	Open	Addition	Sed	0.97
5	34	Open	Addition	Sed	2.79
5	34	Open	Addition	Sed	3.63
5	34	Open	Addition	Sed	17.70
5	34	Open	Addition	Sed	11.84
5	34	Open	Addition	Sed	5.75
5	34	Open	Addition	Sed	2.05
5	34	Open	Addition	Sed	4.93
5	34	Open	Addition	Sed	5.57
5	34	Open	Addition	Sed	2.19
5	34	Open	Addition	Sed	1.76
5	34	Open	Addition	Sed	2.11
5	34	Open	Addition	Sed	3.44
5	34	Open	Addition	Sed	2.61
5	35	Open	Addition	Sed	4.61
5	35	Open	Addition	Sed	1.42
5	35	Open	Addition	Sed	7.64
5	35	Open	Addition	Sed	1.04
5	35	Open	Addition	Sed	2.27
5	35	Open	Addition	Sed	2.32
5	35	Open	Addition	Sed	2.66
5	35	Open	Addition	Sed	1.67
5	35	Open	Addition	Sed	1.22
5	35	Open	Addition	Sed	1.19
5	35	Open	Addition	Sed	1.85
5	35	Open	Addition	Sed	2.70

5	35	Open	Addition	Sed	2.55
5	35	Open	Addition	Sed	1.58
5	35	Open	Addition	Sed	0.00
5	35	Open	Addition	Sed	1.14
5	35	Open	Addition	Sed	2.49
5	35	Open	Addition	Sed	5.35
5	35	Open	Addition	Sed	4.55
5	35	Open	Addition	Sed	0.00
5	36	Open	Addition	Sed	2.26
5	36	Open	Addition	Sed	1.65
5	36	Open	Addition	Sed	2.46
5	36	Open	Addition	Sed	2.47
5	36	Open	Addition	Sed	0.90
5	36	Open	Addition	Sed	1.27
5	36	Open	Addition	Sed	0.98
5	36	Open	Addition	Sed	2.44
5	36	Open	Addition	Sed	1.23
5	36	Open	Addition	Sed	1.21
5	36	Open	Addition	Sed	1.29
5	36	Open	Addition	Sed	1.31
5	36	Open	Addition	Sed	1.20
5	36	Open	Addition	Sed	1.69
5	36	Open	Addition	Sed	0.00
5	36	Open	Addition	Sed	3.06
5	36	Open	Addition	Sed	1.08
5	36	Open	Addition	Sed	1.73
5	36	Open	Addition	Sed	0.67
5	36	Open	Addition	Sed	1.21
6	1	Full	Natural	Sed	1.22
6	1	Full	Natural	Sed	1.70
6	1	Full	Natural	Sed	2.44
6	1	Full	Natural	Sed	1.45
6	1	Full	Natural	Sed	3.15
6	1	Full	Natural	Sed	1.86
6	1	Full	Natural	Sed	2.74
6	1	Full	Natural	Sed	2.28
6	1	Full	Natural	Sed	1.42
6	1	Full	Natural	Sed	2.51
6	1	Full	Natural	Sed	2.49
6	1	Full	Natural	Sed	1.98
6	1	Full	Natural	Sed	2.46
6	1	Full	Natural	Sed	0.84
6	1	Full	Natural	Sed	1.88
6	1	Full	Natural	Sed	1.29
6	1	Full	Natural	Sed	2.09
6	1	Full	Natural	Sed	0.65
6	1	Full	Natural	Sed	1.98
6	1	Full	Natural	Sed	0.67
6	2	Full	Natural	Sed	1.53
6	2	Full	Natural	Sed	1.05
6	2	Full	Natural	Sed	3.61
6	2	Full	Natural	Sed	2.40
6	2	Full	Natural	Sed	3.57

6	2	Full	Natural	Sed	2.40
6	2	Full	Natural	Sed	0.44
6	2	Full	Natural	Sed	1.62
6	2	Full	Natural	Sed	2.12
6	2	Full	Natural	Sed	2.13
6	2	Full	Natural	Sed	2.11
6	2	Full	Natural	Sed	2.87
6	2	Full	Natural	Sed	3.44
6	2	Full	Natural	Sed	1.81
6	2	Full	Natural	Sed	2.06
6	2	Full	Natural	Sed	2.32
6	2	Full	Natural	Sed	2.17
6	2	Full	Natural	Sed	3.06
6	2	Full	Natural	Sed	1.97
6	2	Full	Natural	Sed	2.24
6	3	Full	Natural	Sed	2.64
6	3	Full	Natural	Sed	1.69
6	3	Full	Natural	Sed	2.13
6	3	Full	Natural	Sed	3.54
6	3	Full	Natural	Sed	1.83
6	3	Full	Natural	Sed	3.74
6	3	Full	Natural	Sed	1.64
6	3	Full	Natural	Sed	2.09
6	3	Full	Natural	Sed	3.67
6	3	Full	Natural	Sed	3.09
6	3	Full	Natural	Sed	1.82
6	3	Full	Natural	Sed	2.02
6	3	Full	Natural	Sed	1.53
6	3	Full	Natural	Sed	3.14
6	3	Full	Natural	Sed	1.76
6	3	Full	Natural	Sed	3.80
6	3	Full	Natural	Sed	1.98
6	3	Full	Natural	Sed	1.68
6	3	Full	Natural	Sed	1.97
6	3	Full	Natural	Sed	2.65
6	4	Full	Addition	Sed	1.24
6	4	Full	Addition	Sed	1.48
6	4	Full	Addition	Sed	2.40
6	4	Full	Addition	Sed	3.38
6	4	Full	Addition	Sed	2.60
6	4	Full	Addition	Sed	2.43
6	4	Full	Addition	Sed	2.25
6	4	Full	Addition	Sed	0.74
6	4	Full	Addition	Sed	3.88
6	4	Full	Addition	Sed	1.90
6	4	Full	Addition	Sed	3.18
6	4	Full	Addition	Sed	2.69
6	4	Full	Addition	Sed	0.69
6	4	Full	Addition	Sed	1.91
6	4	Full	Addition	Sed	2.04
6	4	Full	Addition	Sed	2.79
6	4	Full	Addition	Sed	1.76
6	4	Full	Addition	Sed	2.53

6	4	Full	Addition	Sed	3.07
6	4	Full	Addition	Sed	4.54
6	5	Full	Addition	Sed	2.36
6	5	Full	Addition	Sed	1.17
6	5	Full	Addition	Sed	1.36
6	5	Full	Addition	Sed	1.52
6	5	Full	Addition	Sed	2.06
6	5	Full	Addition	Sed	1.79
6	5	Full	Addition	Sed	2.13
6	5	Full	Addition	Sed	2.08
6	5	Full	Addition	Sed	1.59
6	5	Full	Addition	Sed	1.21
6	5	Full	Addition	Sed	1.37
6	5	Full	Addition	Sed	1.73
6	5	Full	Addition	Sed	1.80
6	5	Full	Addition	Sed	1.74
6	5	Full	Addition	Sed	2.60
6	5	Full	Addition	Sed	1.67
6	5	Full	Addition	Sed	2.99
6	5	Full	Addition	Sed	3.96
6	5	Full	Addition	Sed	2.99
6	5	Full	Addition	Sed	1.09
6	6	Full	Addition	Sed	3.36
6	6	Full	Addition	Sed	2.41
6	6	Full	Addition	Sed	2.09
6	6	Full	Addition	Sed	4.40
6	6	Full	Addition	Sed	2.97
6	6	Full	Addition	Sed	4.80
6	6	Full	Addition	Sed	5.42
6	6	Full	Addition	Sed	9.05
6	6	Full	Addition	Sed	2.49
6	6	Full	Addition	Sed	3.68
6	6	Full	Addition	Sed	2.19
6	6	Full	Addition	Sed	1.31
6	6	Full	Addition	Sed	2.46
6	6	Full	Addition	Sed	2.22
6	6	Full	Addition	Sed	0.73
6	6	Full	Addition	Sed	1.78
6	6	Full	Addition	Sed	1.98
6	6	Full	Addition	Sed	1.57
6	6	Full	Addition	Sed	1.21
6	6	Full	Addition	Sed	1.52
6	7	Half	Natural	Sed	2.47
6	7	Half	Natural	Sed	1.66
6	7	Half	Natural	Sed	2.37
6	7	Half	Natural	Sed	2.06
6	7	Half	Natural	Sed	4.36
6	7	Half	Natural	Sed	1.53
6	7	Half	Natural	Sed	3.50
6	7	Half	Natural	Sed	2.05
6	7	Half	Natural	Sed	1.39
6	7	Half	Natural	Sed	3.14
6	7	Half	Natural	Sed	2.61

6	7	Half	Natural	Sed	1.62
6	7	Half	Natural	Sed	4.78
6	7	Half	Natural	Sed	2.48
6	7	Half	Natural	Sed	5.91
6	7	Half	Natural	Sed	2.46
6	7	Half	Natural	Sed	2.13
6	7	Half	Natural	Sed	1.50
6	7	Half	Natural	Sed	2.16
6	7	Half	Natural	Sed	1.33
6	8	Half	Natural	Sed	0.79
6	8	Half	Natural	Sed	1.08
6	8	Half	Natural	Sed	2.63
6	8	Half	Natural	Sed	1.15
6	8	Half	Natural	Sed	1.41
6	8	Half	Natural	Sed	1.60
6	8	Half	Natural	Sed	1.49
6	8	Half	Natural	Sed	1.18
6	8	Half	Natural	Sed	1.65
6	8	Half	Natural	Sed	1.56
6	8	Half	Natural	Sed	1.12
6	8	Half	Natural	Sed	1.10
6	8	Half	Natural	Sed	1.52
6	8	Half	Natural	Sed	1.54
6	8	Half	Natural	Sed	0.64
6	8	Half	Natural	Sed	3.29
6	8	Half	Natural	Sed	1.80
6	8	Half	Natural	Sed	2.62
6	8	Half	Natural	Sed	1.47
6	8	Half	Natural	Sed	0.89
6	9	Half	Natural	Sed	1.40
6	9	Half	Natural	Sed	8.82
6	9	Half	Natural	Sed	2.84
6	9	Half	Natural	Sed	1.78
6	9	Half	Natural	Sed	1.50
6	9	Half	Natural	Sed	2.00
6	9	Half	Natural	Sed	4.04
6	9	Half	Natural	Sed	1.57
6	9	Half	Natural	Sed	2.08
6	9	Half	Natural	Sed	1.59
6	9	Half	Natural	Sed	1.59
6	9	Half	Natural	Sed	2.42
6	9	Half	Natural	Sed	8.60
6	9	Half	Natural	Sed	0.82
6	9	Half	Natural	Sed	1.50
6	9	Half	Natural	Sed	2.98
6	9	Half	Natural	Sed	2.82
6	9	Half	Natural	Sed	3.20
6	9	Half	Natural	Sed	1.79
6	9	Half	Natural	Sed	2.00
6	10	Half	Addition	Sed	2.47
6	10	Half	Addition	Sed	2.34
6	10	Half	Addition	Sed	0.76
6	10	Half	Addition	Sed	2.25

6	10	Half	Addition	Sed	2.17
6	10	Half	Addition	Sed	2.05
6	10	Half	Addition	Sed	1.68
6	10	Half	Addition	Sed	1.78
6	10	Half	Addition	Sed	1.32
6	10	Half	Addition	Sed	1.64
6	10	Half	Addition	Sed	3.05
6	10	Half	Addition	Sed	1.21
6	10	Half	Addition	Sed	2.64
6	10	Half	Addition	Sed	3.70
6	10	Half	Addition	Sed	2.07
6	10	Half	Addition	Sed	1.85
6	10	Half	Addition	Sed	1.77
6	10	Half	Addition	Sed	3.36
6	10	Half	Addition	Sed	3.81
6	10	Half	Addition	Sed	1.79
6	11	Half	Addition	Sed	5.34
6	11	Half	Addition	Sed	1.70
6	11	Half	Addition	Sed	2.87
6	11	Half	Addition	Sed	1.90
6	11	Half	Addition	Sed	14.57
6	11	Half	Addition	Sed	1.56
6	11	Half	Addition	Sed	1.69
6	11	Half	Addition	Sed	1.34
6	11	Half	Addition	Sed	1.41
6	11	Half	Addition	Sed	1.81
6	11	Half	Addition	Sed	2.30
6	11	Half	Addition	Sed	1.62
6	11	Half	Addition	Sed	0.67
6	11	Half	Addition	Sed	3.53
6	11	Half	Addition	Sed	1.40
6	11	Half	Addition	Sed	1.69
6	11	Half	Addition	Sed	1.39
6	11	Half	Addition	Sed	1.57
6	11	Half	Addition	Sed	1.01
6	11	Half	Addition	Sed	2.05
6	12	Half	Addition	Sed	2.44
6	12	Half	Addition	Sed	2.49
6	12	Half	Addition	Sed	4.53
6	12	Half	Addition	Sed	2.15
6	12	Half	Addition	Sed	4.26
6	12	Half	Addition	Sed	2.04
6	12	Half	Addition	Sed	3.11
6	12	Half	Addition	Sed	13.82
6	12	Half	Addition	Sed	1.68
6	12	Half	Addition	Sed	2.32
6	12	Half	Addition	Sed	4.37
6	12	Half	Addition	Sed	4.15
6	12	Half	Addition	Sed	1.72
6	12	Half	Addition	Sed	6.67
6	12	Half	Addition	Sed	2.99
6	12	Half	Addition	Sed	5.56
6	12	Half	Addition	Sed	3.74

6	12	Half	Addition	Sed	2.85
6	12	Half	Addition	Sed	1.33
6	12	Half	Addition	Sed	5.84
6	13	Open	Natural	Sed	1.77
6	13	Open	Natural	Sed	12.54
6	13	Open	Natural	Sed	1.76
6	13	Open	Natural	Sed	1.58
6	13	Open	Natural	Sed	3.34
6	13	Open	Natural	Sed	3.40
6	13	Open	Natural	Sed	2.64
6	13	Open	Natural	Sed	2.06
6	13	Open	Natural	Sed	2.49
6	13	Open	Natural	Sed	2.89
6	13	Open	Natural	Sed	1.56
6	13	Open	Natural	Sed	1.88
6	13	Open	Natural	Sed	2.88
6	13	Open	Natural	Sed	1.18
6	13	Open	Natural	Sed	0.00
6	13	Open	Natural	Sed	1.22
6	13	Open	Natural	Sed	1.60
6	13	Open	Natural	Sed	2.57
6	13	Open	Natural	Sed	1.41
6	13	Open	Natural	Sed	1.54
6	14	Open	Natural	Sed	1.21
6	14	Open	Natural	Sed	1.10
6	14	Open	Natural	Sed	1.56
6	14	Open	Natural	Sed	2.69
6	14	Open	Natural	Sed	2.86
6	14	Open	Natural	Sed	1.58
6	14	Open	Natural	Sed	2.41
6	14	Open	Natural	Sed	2.23
6	14	Open	Natural	Sed	1.83
6	14	Open	Natural	Sed	0.83
6	14	Open	Natural	Sed	2.25
6	14	Open	Natural	Sed	1.50
6	14	Open	Natural	Sed	1.43
6	14	Open	Natural	Sed	1.64
6	14	Open	Natural	Sed	6.09
6	14	Open	Natural	Sed	0.00
6	14	Open	Natural	Sed	1.36
6	14	Open	Natural	Sed	0.90
6	14	Open	Natural	Sed	2.08
6	14	Open	Natural	Sed	1.56
6	15	Open	Natural	Sed	1.31
6	15	Open	Natural	Sed	2.02
6	15	Open	Natural	Sed	3.42
6	15	Open	Natural	Sed	2.71
6	15	Open	Natural	Sed	1.80
6	15	Open	Natural	Sed	1.47
6	15	Open	Natural	Sed	1.76
6	15	Open	Natural	Sed	0.84
6	15	Open	Natural	Sed	1.80
6	15	Open	Natural	Sed	0.43

6	15	Open	Natural	Sed	1.61
6	15	Open	Natural	Sed	1.72
6	15	Open	Natural	Sed	5.27
6	15	Open	Natural	Sed	0.98
6	15	Open	Natural	Sed	2.29
6	15	Open	Natural	Sed	1.42
6	15	Open	Natural	Sed	1.98
6	15	Open	Natural	Sed	1.26
6	15	Open	Natural	Sed	1.89
6	15	Open	Natural	Sed	0.63
6	16	Open	Addition	Sed	1.67
6	16	Open	Addition	Sed	2.60
6	16	Open	Addition	Sed	1.02
6	16	Open	Addition	Sed	2.92
6	16	Open	Addition	Sed	2.06
6	16	Open	Addition	Sed	2.46
6	16	Open	Addition	Sed	1.15
6	16	Open	Addition	Sed	2.94
6	16	Open	Addition	Sed	1.94
6	16	Open	Addition	Sed	1.79
6	16	Open	Addition	Sed	0.51
6	16	Open	Addition	Sed	3.85
6	16	Open	Addition	Sed	2.48
6	16	Open	Addition	Sed	1.28
6	16	Open	Addition	Sed	3.16
6	16	Open	Addition	Sed	0.53
6	16	Open	Addition	Sed	1.28
6	16	Open	Addition	Sed	5.11
6	16	Open	Addition	Sed	1.44
6	16	Open	Addition	Sed	1.81
6	17	Open	Addition	Sed	2.17
6	17	Open	Addition	Sed	1.09
6	17	Open	Addition	Sed	1.56
6	17	Open	Addition	Sed	1.30
6	17	Open	Addition	Sed	2.51
6	17	Open	Addition	Sed	1.83
6	17	Open	Addition	Sed	3.20
6	17	Open	Addition	Sed	2.43
6	17	Open	Addition	Sed	2.75
6	17	Open	Addition	Sed	1.39
6	17	Open	Addition	Sed	1.48
6	17	Open	Addition	Sed	1.89
6	17	Open	Addition	Sed	1.95
6	17	Open	Addition	Sed	2.06
6	17	Open	Addition	Sed	3.29
6	17	Open	Addition	Sed	2.30
6	17	Open	Addition	Sed	2.37
6	17	Open	Addition	Sed	2.64
6	17	Open	Addition	Sed	4.88
6	17	Open	Addition	Sed	3.21
6	18	Open	Addition	Sed	1.28
6	18	Open	Addition	Sed	3.52
6	18	Open	Addition	Sed	2.38

6	18	Open	Addition	Sed	1.37
6	18	Open	Addition	Sed	1.64
6	18	Open	Addition	Sed	0.53
6	18	Open	Addition	Sed	1.13
6	18	Open	Addition	Sed	3.69
6	18	Open	Addition	Sed	1.78
6	18	Open	Addition	Sed	1.82
6	18	Open	Addition	Sed	1.57
6	18	Open	Addition	Sed	1.28
6	18	Open	Addition	Sed	1.83
6	18	Open	Addition	Sed	1.80
6	18	Open	Addition	Sed	1.75
6	18	Open	Addition	Sed	2.44
6	18	Open	Addition	Sed	1.85
6	18	Open	Addition	Sed	1.93
6	18	Open	Addition	Sed	2.10
6	18	Open	Addition	Sed	2.77
6	19	Cage	Natural	Sed	1.22
6	19	Cage	Natural	Sed	4.48
6	19	Cage	Natural	Sed	3.83
6	19	Cage	Natural	Sed	6.49
6	19	Cage	Natural	Sed	4.19
6	19	Cage	Natural	Sed	3.35
6	19	Cage	Natural	Sed	2.64
6	19	Cage	Natural	Sed	4.33
6	19	Cage	Natural	Sed	4.97
6	19	Cage	Natural	Sed	5.70
6	19	Cage	Natural	Sed	9.99
6	19	Cage	Natural	Sed	2.16
6	19	Cage	Natural	Sed	3.76
6	19	Cage	Natural	Sed	5.32
6	19	Cage	Natural	Sed	2.00
6	19	Cage	Natural	Sed	2.93
6	19	Cage	Natural	Sed	2.38
6	19	Cage	Natural	Sed	6.21
6	19	Cage	Natural	Sed	4.88
6	19	Cage	Natural	Sed	30.16
6	20	Cage	Natural	Sed	1.18
6	20	Cage	Natural	Sed	7.42
6	20	Cage	Natural	Sed	1.78
6	20	Cage	Natural	Sed	4.30
6	20	Cage	Natural	Sed	3.16
6	20	Cage	Natural	Sed	7.37
6	20	Cage	Natural	Sed	1.81
6	20	Cage	Natural	Sed	4.89
6	20	Cage	Natural	Sed	1.69
6	20	Cage	Natural	Sed	3.84
6	20	Cage	Natural	Sed	1.77
6	20	Cage	Natural	Sed	2.91
6	20	Cage	Natural	Sed	2.96
6	20	Cage	Natural	Sed	2.80
6	20	Cage	Natural	Sed	1.85
6	20	Cage	Natural	Sed	3.39

6	20	Cage	Natural	Sed	0.87
6	20	Cage	Natural	Sed	6.73
6	20	Cage	Natural	Sed	5.32
6	20	Cage	Natural	Sed	3.89
6	21	Cage	Natural	Sed	1.19
6	21	Cage	Natural	Sed	3.41
6	21	Cage	Natural	Sed	4.29
6	21	Cage	Natural	Sed	1.79
6	21	Cage	Natural	Sed	17.67
6	21	Cage	Natural	Sed	0.88
6	21	Cage	Natural	Sed	4.33
6	21	Cage	Natural	Sed	1.68
6	21	Cage	Natural	Sed	2.05
6	21	Cage	Natural	Sed	3.26
6	21	Cage	Natural	Sed	1.35
6	21	Cage	Natural	Sed	3.81
6	21	Cage	Natural	Sed	4.35
6	21	Cage	Natural	Sed	4.12
6	21	Cage	Natural	Sed	5.74
6	21	Cage	Natural	Sed	3.69
6	21	Cage	Natural	Sed	4.41
6	21	Cage	Natural	Sed	1.31
6	21	Cage	Natural	Sed	5.45
6	21	Cage	Natural	Sed	0.87
6	22	Cage	Addition	Sed	3.65
6	22	Cage	Addition	Sed	2.84
6	22	Cage	Addition	Sed	1.68
6	22	Cage	Addition	Sed	2.39
6	22	Cage	Addition	Sed	3.55
6	22	Cage	Addition	Sed	4.63
6	22	Cage	Addition	Sed	4.90
6	22	Cage	Addition	Sed	1.96
6	22	Cage	Addition	Sed	2.56
6	22	Cage	Addition	Sed	2.62
6	22	Cage	Addition	Sed	1.18
6	22	Cage	Addition	Sed	1.50
6	22	Cage	Addition	Sed	5.45
6	22	Cage	Addition	Sed	3.16
6	22	Cage	Addition	Sed	1.52
6	22	Cage	Addition	Sed	2.16
6	22	Cage	Addition	Sed	3.50
6	22	Cage	Addition	Sed	3.82
6	22	Cage	Addition	Sed	5.95
6	22	Cage	Addition	Sed	1.59
6	23	Cage	Addition	Sed	4.72
6	23	Cage	Addition	Sed	2.68
6	23	Cage	Addition	Sed	0.00
6	23	Cage	Addition	Sed	6.24
6	23	Cage	Addition	Sed	3.69
6	23	Cage	Addition	Sed	4.56
6	23	Cage	Addition	Sed	4.33
6	23	Cage	Addition	Sed	6.88
6	23	Cage	Addition	Sed	0.00

6	23	Cage	Addition	Sed	4.46
6	23	Cage	Addition	Sed	9.12
6	23	Cage	Addition	Sed	1.62
6	23	Cage	Addition	Sed	9.61
6	23	Cage	Addition	Sed	1.92
6	23	Cage	Addition	Sed	10.58
6	23	Cage	Addition	Sed	1.72
6	23	Cage	Addition	Sed	3.29
6	23	Cage	Addition	Sed	3.10
6	23	Cage	Addition	Sed	3.84
6	23	Cage	Addition	Sed	2.17
6	24	Cage	Addition	Sed	8.72
6	24	Cage	Addition	Sed	2.13
6	24	Cage	Addition	Sed	5.53
6	24	Cage	Addition	Sed	7.07
6	24	Cage	Addition	Sed	2.40
6	24	Cage	Addition	Sed	4.61
6	24	Cage	Addition	Sed	6.89
6	24	Cage	Addition	Sed	2.16
6	24	Cage	Addition	Sed	3.75
6	24	Cage	Addition	Sed	11.64
6	24	Cage	Addition	Sed	3.49
6	24	Cage	Addition	Sed	6.16
6	24	Cage	Addition	Sed	4.93
6	24	Cage	Addition	Sed	6.77
6	24	Cage	Addition	Sed	4.02
6	24	Cage	Addition	Sed	2.54
6	24	Cage	Addition	Sed	8.08
6	24	Cage	Addition	Sed	2.26
6	24	Cage	Addition	Sed	3.00
6	24	Cage	Addition	Sed	2.53
6	25	Half	Natural	Sed	5.05
6	25	Half	Natural	Sed	1.70
6	25	Half	Natural	Sed	7.42
6	25	Half	Natural	Sed	7.20
6	25	Half	Natural	Sed	2.50
6	25	Half	Natural	Sed	3.85
6	25	Half	Natural	Sed	4.56
6	25	Half	Natural	Sed	5.23
6	25	Half	Natural	Sed	3.23
6	25	Half	Natural	Sed	5.45
6	25	Half	Natural	Sed	2.85
6	25	Half	Natural	Sed	2.42
6	25	Half	Natural	Sed	2.75
6	25	Half	Natural	Sed	8.66
6	25	Half	Natural	Sed	2.40
6	25	Half	Natural	Sed	4.35
6	25	Half	Natural	Sed	3.87
6	25	Half	Natural	Sed	1.71
6	25	Half	Natural	Sed	3.35
6	25	Half	Natural	Sed	2.19
6	26	Half	Natural	Sed	2.67
6	26	Half	Natural	Sed	1.16

6	26	Half	Natural	Sed	3.72
6	26	Half	Natural	Sed	1.44
6	26	Half	Natural	Sed	2.53
6	26	Half	Natural	Sed	0.00
6	26	Half	Natural	Sed	4.81
6	26	Half	Natural	Sed	1.94
6	26	Half	Natural	Sed	3.93
6	26	Half	Natural	Sed	2.14
6	26	Half	Natural	Sed	1.51
6	26	Half	Natural	Sed	1.72
6	26	Half	Natural	Sed	2.40
6	26	Half	Natural	Sed	1.39
6	26	Half	Natural	Sed	2.43
6	26	Half	Natural	Sed	1.70
6	26	Half	Natural	Sed	1.36
6	26	Half	Natural	Sed	1.24
6	26	Half	Natural	Sed	1.87
6	26	Half	Natural	Sed	1.69
6	27	Half	Natural	Sed	0.65
6	27	Half	Natural	Sed	1.97
6	27	Half	Natural	Sed	1.42
6	27	Half	Natural	Sed	1.89
6	27	Half	Natural	Sed	1.23
6	27	Half	Natural	Sed	2.24
6	27	Half	Natural	Sed	1.34
6	27	Half	Natural	Sed	2.55
6	27	Half	Natural	Sed	1.15
6	27	Half	Natural	Sed	2.35
6	27	Half	Natural	Sed	1.26
6	27	Half	Natural	Sed	2.84
6	27	Half	Natural	Sed	2.13
6	27	Half	Natural	Sed	3.94
6	27	Half	Natural	Sed	2.86
6	27	Half	Natural	Sed	1.43
6	27	Half	Natural	Sed	1.49
6	27	Half	Natural	Sed	1.81
6	27	Half	Natural	Sed	2.91
6	27	Half	Natural	Sed	2.21
6	28	Half	Addition	Sed	1.44
6	28	Half	Addition	Sed	2.69
6	28	Half	Addition	Sed	1.59
6	28	Half	Addition	Sed	0.41
6	28	Half	Addition	Sed	3.32
6	28	Half	Addition	Sed	2.35
6	28	Half	Addition	Sed	0.55
6	28	Half	Addition	Sed	0.00
6	28	Half	Addition	Sed	2.45
6	28	Half	Addition	Sed	1.52
6	28	Half	Addition	Sed	4.60
6	28	Half	Addition	Sed	0.89
6	28	Half	Addition	Sed	4.88
6	28	Half	Addition	Sed	3.73
6	28	Half	Addition	Sed	2.74

6	28	Half	Addition	Sed	1.82
6	28	Half	Addition	Sed	2.03
6	28	Half	Addition	Sed	1.22
6	28	Half	Addition	Sed	2.81
6	28	Half	Addition	Sed	0.88
6	29	Half	Addition	Sed	1.51
6	29	Half	Addition	Sed	3.08
6	29	Half	Addition	Sed	0.79
6	29	Half	Addition	Sed	0.00
6	29	Half	Addition	Sed	1.41
6	29	Half	Addition	Sed	1.84
6	29	Half	Addition	Sed	1.40
6	29	Half	Addition	Sed	1.58
6	29	Half	Addition	Sed	2.67
6	29	Half	Addition	Sed	2.21
6	29	Half	Addition	Sed	0.00
6	29	Half	Addition	Sed	2.39
6	29	Half	Addition	Sed	3.61
6	29	Half	Addition	Sed	1.11
6	29	Half	Addition	Sed	0.53
6	29	Half	Addition	Sed	1.49
6	29	Half	Addition	Sed	1.42
6	29	Half	Addition	Sed	2.15
6	29	Half	Addition	Sed	1.96
6	29	Half	Addition	Sed	3.67
6	30	Half	Addition	Sed	3.66
6	30	Half	Addition	Sed	1.49
6	30	Half	Addition	Sed	12.91
6	30	Half	Addition	Sed	3.29
6	30	Half	Addition	Sed	5.39
6	30	Half	Addition	Sed	3.44
6	30	Half	Addition	Sed	2.15
6	30	Half	Addition	Sed	3.07
6	30	Half	Addition	Sed	2.50
6	30	Half	Addition	Sed	2.83
6	30	Half	Addition	Sed	2.21
6	30	Half	Addition	Sed	1.46
6	30	Half	Addition	Sed	2.61
6	30	Half	Addition	Sed	2.13
6	30	Half	Addition	Sed	2.21
6	30	Half	Addition	Sed	1.86
6	30	Half	Addition	Sed	2.15
6	30	Half	Addition	Sed	2.42
6	30	Half	Addition	Sed	1.43
6	30	Half	Addition	Sed	3.41
6	31	Open	Natural	Sed	1.89
6	31	Open	Natural	Sed	1.83
6	31	Open	Natural	Sed	1.68
6	31	Open	Natural	Sed	0.00
6	31	Open	Natural	Sed	1.30
6	31	Open	Natural	Sed	1.44
6	31	Open	Natural	Sed	2.17
6	31	Open	Natural	Sed	2.90

6	31	Open	Natural	Sed	0.57
6	31	Open	Natural	Sed	3.02
6	31	Open	Natural	Sed	0.92
6	31	Open	Natural	Sed	1.78
6	31	Open	Natural	Sed	2.46
6	31	Open	Natural	Sed	0.00
6	31	Open	Natural	Sed	0.64
6	31	Open	Natural	Sed	0.00
6	31	Open	Natural	Sed	1.65
6	31	Open	Natural	Sed	2.79
6	31	Open	Natural	Sed	0.89
6	31	Open	Natural	Sed	2.50
6	32	Open	Natural	Sed	1.28
6	32	Open	Natural	Sed	1.16
6	32	Open	Natural	Sed	1.42
6	32	Open	Natural	Sed	3.00
6	32	Open	Natural	Sed	1.21
6	32	Open	Natural	Sed	3.00
6	32	Open	Natural	Sed	1.63
6	32	Open	Natural	Sed	2.79
6	32	Open	Natural	Sed	1.21
6	32	Open	Natural	Sed	1.91
6	32	Open	Natural	Sed	0.00
6	32	Open	Natural	Sed	2.23
6	32	Open	Natural	Sed	0.00
6	32	Open	Natural	Sed	1.76
6	32	Open	Natural	Sed	1.92
6	32	Open	Natural	Sed	1.19
6	32	Open	Natural	Sed	2.86
6	32	Open	Natural	Sed	2.22
6	32	Open	Natural	Sed	2.56
6	32	Open	Natural	Sed	1.34
6	33	Open	Natural	Sed	4.72
6	33	Open	Natural	Sed	2.04
6	33	Open	Natural	Sed	0.67
6	33	Open	Natural	Sed	1.80
6	33	Open	Natural	Sed	1.99
6	33	Open	Natural	Sed	2.07
6	33	Open	Natural	Sed	6.17
6	33	Open	Natural	Sed	1.42
6	33	Open	Natural	Sed	1.85
6	33	Open	Natural	Sed	2.00
6	33	Open	Natural	Sed	2.88
6	33	Open	Natural	Sed	1.71
6	33	Open	Natural	Sed	1.16
6	33	Open	Natural	Sed	1.21
6	33	Open	Natural	Sed	2.46
6	33	Open	Natural	Sed	1.61
6	33	Open	Natural	Sed	1.03
6	33	Open	Natural	Sed	2.63
6	33	Open	Natural	Sed	3.77
6	33	Open	Natural	Sed	0.97
6	34	Open	Addition	Sed	10.74

6	34	Open	Addition	Sed	2.32
6	34	Open	Addition	Sed	6.26
6	34	Open	Addition	Sed	5.51
6	34	Open	Addition	Sed	5.10
6	34	Open	Addition	Sed	3.38
6	34	Open	Addition	Sed	1.09
6	34	Open	Addition	Sed	5.55
6	34	Open	Addition	Sed	4.58
6	34	Open	Addition	Sed	6.20
6	34	Open	Addition	Sed	2.50
6	34	Open	Addition	Sed	4.87
6	34	Open	Addition	Sed	9.43
6	34	Open	Addition	Sed	4.73
6	34	Open	Addition	Sed	6.97
6	34	Open	Addition	Sed	5.94
6	34	Open	Addition	Sed	6.84
6	34	Open	Addition	Sed	4.97
6	34	Open	Addition	Sed	7.71
6	34	Open	Addition	Sed	26.45
6	35	Open	Addition	Sed	1.19
6	35	Open	Addition	Sed	0.00
6	35	Open	Addition	Sed	2.45
6	35	Open	Addition	Sed	2.39
6	35	Open	Addition	Sed	2.49
6	35	Open	Addition	Sed	2.54
6	35	Open	Addition	Sed	4.66
6	35	Open	Addition	Sed	3.60
6	35	Open	Addition	Sed	2.08
6	35	Open	Addition	Sed	3.28
6	35	Open	Addition	Sed	2.50
6	35	Open	Addition	Sed	7.51
6	35	Open	Addition	Sed	2.70
6	35	Open	Addition	Sed	10.21
6	35	Open	Addition	Sed	4.51
6	35	Open	Addition	Sed	3.87
6	35	Open	Addition	Sed	3.60
6	35	Open	Addition	Sed	1.20
6	35	Open	Addition	Sed	2.82
6	35	Open	Addition	Sed	3.04
6	36	Open	Addition	Sed	0.54
6	36	Open	Addition	Sed	1.41
6	36	Open	Addition	Sed	0.00
6	36	Open	Addition	Sed	0.98
6	36	Open	Addition	Sed	1.55
6	36	Open	Addition	Sed	1.52
6	36	Open	Addition	Sed	0.49
6	36	Open	Addition	Sed	1.67
6	36	Open	Addition	Sed	2.83
6	36	Open	Addition	Sed	1.41
6	36	Open	Addition	Sed	16.77
6	36	Open	Addition	Sed	3.55
6	36	Open	Addition	Sed	4.15
6	36	Open	Addition	Sed	1.64

6	36	Open	Addition	Sed	1.36
6	36	Open	Addition	Sed	1.60
6	36	Open	Addition	Sed	1.80
6	36	Open	Addition	Sed	1.72
6	36	Open	Addition	Sed	2.67
6	36	Open	Addition	Sed	1.42
0	1	Full	Natural	Turf	3.56
0	1	Full	Natural	Turf	2.80
0	1	Full	Natural	Turf	3.04
0	1	Full	Natural	Turf	2.82
0	1	Full	Natural	Turf	2.90
0	1	Full	Natural	Turf	2.46
0	1	Full	Natural	Turf	3.20
0	1	Full	Natural	Turf	3.64
0	1	Full	Natural	Turf	3.80
0	1	Full	Natural	Turf	3.22
0	1	Full	Natural	Turf	4.48
0	1	Full	Natural	Turf	2.54
0	1	Full	Natural	Turf	2.30
0	1	Full	Natural	Turf	1.88
0	1	Full	Natural	Turf	3.42
0	1	Full	Natural	Turf	3.02
0	1	Full	Natural	Turf	2.76
0	1	Full	Natural	Turf	2.08
0	1	Full	Natural	Turf	2.84
0	1	Full	Natural	Turf	3.68
0	2	Full	Natural	Turf	4.27
0	2	Full	Natural	Turf	2.15
0	2	Full	Natural	Turf	10.61
0	2	Full	Natural	Turf	3.50
0	2	Full	Natural	Turf	1.96
0	2	Full	Natural	Turf	3.54
0	2	Full	Natural	Turf	2.98
0	2	Full	Natural	Turf	4.25
0	2	Full	Natural	Turf	4.38
0	2	Full	Natural	Turf	5.53
0	2	Full	Natural	Turf	3.02
0	2	Full	Natural	Turf	3.25
0	2	Full	Natural	Turf	3.32
0	2	Full	Natural	Turf	2.35
0	2	Full	Natural	Turf	0.00
0	2	Full	Natural	Turf	3.89
0	2	Full	Natural	Turf	4.57
0	2	Full	Natural	Turf	2.77
0	2	Full	Natural	Turf	2.73
0	2	Full	Natural	Turf	3.07
0	3	Full	Natural	Turf	2.44
0	3	Full	Natural	Turf	2.24
0	3	Full	Natural	Turf	1.65
0	3	Full	Natural	Turf	1.22
0	3	Full	Natural	Turf	2.32
0	3	Full	Natural	Turf	1.99
0	3	Full	Natural	Turf	1.64

0	3	Full	Natural	Turf	1.40
0	3	Full	Natural	Turf	3.44
0	3	Full	Natural	Turf	2.09
0	3	Full	Natural	Turf	2.93
0	3	Full	Natural	Turf	3.19
0	3	Full	Natural	Turf	3.90
0	3	Full	Natural	Turf	2.02
0	3	Full	Natural	Turf	1.92
0	3	Full	Natural	Turf	2.65
0	3	Full	Natural	Turf	2.68
0	3	Full	Natural	Turf	2.00
0	3	Full	Natural	Turf	3.70
0	3	Full	Natural	Turf	2.41
0	4	Full	Addition	Turf	2.09
0	4	Full	Addition	Turf	5.02
0	4	Full	Addition	Turf	2.19
0	4	Full	Addition	Turf	3.19
0	4	Full	Addition	Turf	6.98
0	4	Full	Addition	Turf	18.58
0	4	Full	Addition	Turf	1.53
0	4	Full	Addition	Turf	2.31
0	4	Full	Addition	Turf	2.60
0	4	Full	Addition	Turf	1.67
0	4	Full	Addition	Turf	2.73
0	4	Full	Addition	Turf	2.15
0	4	Full	Addition	Turf	2.31
0	4	Full	Addition	Turf	3.11
0	4	Full	Addition	Turf	1.89
0	4	Full	Addition	Turf	2.06
0	4	Full	Addition	Turf	1.88
0	4	Full	Addition	Turf	1.44
0	4	Full	Addition	Turf	2.79
0	4	Full	Addition	Turf	1.85
0	5	Full	Addition	Turf	1.58
0	5	Full	Addition	Turf	2.18
0	5	Full	Addition	Turf	3.04
0	5	Full	Addition	Turf	2.70
0	5	Full	Addition	Turf	2.24
0	5	Full	Addition	Turf	2.53
0	5	Full	Addition	Turf	1.76
0	5	Full	Addition	Turf	1.51
0	5	Full	Addition	Turf	4.34
0	5	Full	Addition	Turf	1.69
0	5	Full	Addition	Turf	2.12
0	5	Full	Addition	Turf	4.33
0	5	Full	Addition	Turf	1.94
0	5	Full	Addition	Turf	2.60
0	5	Full	Addition	Turf	2.04
0	5	Full	Addition	Turf	1.35
0	5	Full	Addition	Turf	1.73
0	5	Full	Addition	Turf	1.80
0	5	Full	Addition	Turf	4.59
0	5	Full	Addition	Turf	1.97

0	6	Full	Addition	Turf	1.79
0	6	Full	Addition	Turf	1.75
0	6	Full	Addition	Turf	3.59
0	6	Full	Addition	Turf	2.18
0	6	Full	Addition	Turf	3.27
0	6	Full	Addition	Turf	2.41
0	6	Full	Addition	Turf	1.72
0	6	Full	Addition	Turf	1.53
0	6	Full	Addition	Turf	1.43
0	6	Full	Addition	Turf	2.84
0	6	Full	Addition	Turf	2.50
0	6	Full	Addition	Turf	1.94
0	6	Full	Addition	Turf	1.16
0	6	Full	Addition	Turf	2.28
0	6	Full	Addition	Turf	1.71
0	6	Full	Addition	Turf	2.56
0	6	Full	Addition	Turf	1.58
0	6	Full	Addition	Turf	1.58
0	6	Full	Addition	Turf	3.04
0	6	Full	Addition	Turf	1.75
0	7	Half	Natural	Turf	3.90
0	7	Half	Natural	Turf	3.53
0	7	Half	Natural	Turf	3.03
0	7	Half	Natural	Turf	4.40
0	7	Half	Natural	Turf	3.26
0	7	Half	Natural	Turf	2.37
0	7	Half	Natural	Turf	8.26
0	7	Half	Natural	Turf	2.85
0	7	Half	Natural	Turf	3.09
0	7	Half	Natural	Turf	2.60
0	7	Half	Natural	Turf	4.57
0	7	Half	Natural	Turf	2.73
0	7	Half	Natural	Turf	2.04
0	7	Half	Natural	Turf	0.00
0	7	Half	Natural	Turf	2.69
0	7	Half	Natural	Turf	2.75
0	7	Half	Natural	Turf	3.77
0	7	Half	Natural	Turf	2.45
0	7	Half	Natural	Turf	1.95
0	7	Half	Natural	Turf	4.45
0	8	Half	Natural	Turf	5.36
0	8	Half	Natural	Turf	4.34
0	8	Half	Natural	Turf	10.13
0	8	Half	Natural	Turf	1.79
0	8	Half	Natural	Turf	2.27
0	8	Half	Natural	Turf	3.11
0	8	Half	Natural	Turf	1.44
0	8	Half	Natural	Turf	2.14
0	8	Half	Natural	Turf	2.16
0	8	Half	Natural	Turf	4.46
0	8	Half	Natural	Turf	2.63
0	8	Half	Natural	Turf	2.33
0	8	Half	Natural	Turf	2.77

0	8	Half	Natural	Turf	2.54
0	8	Half	Natural	Turf	3.43
0	8	Half	Natural	Turf	2.96
0	8	Half	Natural	Turf	2.71
0	8	Half	Natural	Turf	2.23
0	8	Half	Natural	Turf	1.70
0	8	Half	Natural	Turf	2.99
0	9	Half	Natural	Turf	4.44
0	9	Half	Natural	Turf	3.26
0	9	Half	Natural	Turf	2.81
0	9	Half	Natural	Turf	2.63
0	9	Half	Natural	Turf	4.87
0	9	Half	Natural	Turf	1.21
0	9	Half	Natural	Turf	2.31
0	9	Half	Natural	Turf	4.49
0	9	Half	Natural	Turf	10.58
0	9	Half	Natural	Turf	3.69
0	9	Half	Natural	Turf	18.99
0	9	Half	Natural	Turf	4.16
0	9	Half	Natural	Turf	3.05
0	9	Half	Natural	Turf	0.00
0	9	Half	Natural	Turf	4.29
0	9	Half	Natural	Turf	4.96
0	9	Half	Natural	Turf	1.62
0	9	Half	Natural	Turf	2.84
0	9	Half	Natural	Turf	2.66
0	9	Half	Natural	Turf	3.52
0	10	Half	Addition	Turf	2.45
0	10	Half	Addition	Turf	3.04
0	10	Half	Addition	Turf	2.99
0	10	Half	Addition	Turf	1.47
0	10	Half	Addition	Turf	2.59
0	10	Half	Addition	Turf	3.50
0	10	Half	Addition	Turf	3.09
0	10	Half	Addition	Turf	3.47
0	10	Half	Addition	Turf	2.66
0	10	Half	Addition	Turf	3.91
0	10	Half	Addition	Turf	2.37
0	10	Half	Addition	Turf	0.00
0	10	Half	Addition	Turf	3.83
0	10	Half	Addition	Turf	1.78
0	10	Half	Addition	Turf	6.03
0	10	Half	Addition	Turf	1.99
0	10	Half	Addition	Turf	5.46
0	10	Half	Addition	Turf	2.47
0	10	Half	Addition	Turf	6.31
0	10	Half	Addition	Turf	2.83
0	11	Half	Addition	Turf	4.22
0	11	Half	Addition	Turf	3.59
0	11	Half	Addition	Turf	1.76
0	11	Half	Addition	Turf	0.00
0	11	Half	Addition	Turf	3.92
0	11	Half	Addition	Turf	3.36

0	11	Half	Addition	Turf	3.00
0	11	Half	Addition	Turf	2.69
0	11	Half	Addition	Turf	1.86
0	11	Half	Addition	Turf	2.99
0	11	Half	Addition	Turf	3.31
0	11	Half	Addition	Turf	2.63
0	11	Half	Addition	Turf	2.46
0	11	Half	Addition	Turf	1.89
0	11	Half	Addition	Turf	2.00
0	11	Half	Addition	Turf	3.73
0	11	Half	Addition	Turf	1.67
0	11	Half	Addition	Turf	3.02
0	11	Half	Addition	Turf	1.30
0	11	Half	Addition	Turf	22.11
0	12	Half	Addition	Turf	2.98
0	12	Half	Addition	Turf	1.50
0	12	Half	Addition	Turf	2.15
0	12	Half	Addition	Turf	3.43
0	12	Half	Addition	Turf	4.29
0	12	Half	Addition	Turf	1.43
0	12	Half	Addition	Turf	1.94
0	12	Half	Addition	Turf	1.79
0	12	Half	Addition	Turf	2.54
0	12	Half	Addition	Turf	2.46
0	12	Half	Addition	Turf	2.75
0	12	Half	Addition	Turf	5.04
0	12	Half	Addition	Turf	2.02
0	12	Half	Addition	Turf	3.78
0	12	Half	Addition	Turf	1.34
0	12	Half	Addition	Turf	1.12
0	12	Half	Addition	Turf	1.75
0	12	Half	Addition	Turf	0.00
0	12	Half	Addition	Turf	2.88
0	12	Half	Addition	Turf	2.28
0	13	Open	Natural	Turf	1.80
0	13	Open	Natural	Turf	2.14
0	13	Open	Natural	Turf	2.72
0	13	Open	Natural	Turf	2.01
0	13	Open	Natural	Turf	0.70
0	13	Open	Natural	Turf	2.54
0	13	Open	Natural	Turf	8.31
0	13	Open	Natural	Turf	9.74
0	13	Open	Natural	Turf	3.18
0	13	Open	Natural	Turf	1.52
0	13	Open	Natural	Turf	3.25
0	13	Open	Natural	Turf	6.18
0	13	Open	Natural	Turf	3.05
0	13	Open	Natural	Turf	2.55
0	13	Open	Natural	Turf	3.65
0	13	Open	Natural	Turf	3.71
0	13	Open	Natural	Turf	1.72
0	13	Open	Natural	Turf	1.68
0	13	Open	Natural	Turf	2.21

0	13 Open	Natural	Turf	1.97
0	14 Open	Natural	Turf	1.74
0	14 Open	Natural	Turf	2.32
0	14 Open	Natural	Turf	1.73
0	14 Open	Natural	Turf	3.70
0	14 Open	Natural	Turf	2.24
0	14 Open	Natural	Turf	2.09
0	14 Open	Natural	Turf	3.04
0	14 Open	Natural	Turf	2.30
0	14 Open	Natural	Turf	3.59
0	14 Open	Natural	Turf	3.20
0	14 Open	Natural	Turf	1.97
0	14 Open	Natural	Turf	3.06
0	14 Open	Natural	Turf	1.79
0	14 Open	Natural	Turf	2.89
0	14 Open	Natural	Turf	1.46
0	14 Open	Natural	Turf	2.73
0	14 Open	Natural	Turf	2.47
0	14 Open	Natural	Turf	2.40
0	14 Open	Natural	Turf	4.47
0	14 Open	Natural	Turf	1.39
0	15 Open	Natural	Turf	2.47
0	15 Open	Natural	Turf	2.62
0	15 Open	Natural	Turf	1.21
0	15 Open	Natural	Turf	3.53
0	15 Open	Natural	Turf	1.83
0	15 Open	Natural	Turf	0.00
0	15 Open	Natural	Turf	1.70
0	15 Open	Natural	Turf	1.42
0	15 Open	Natural	Turf	2.41
0	15 Open	Natural	Turf	1.72
0	15 Open	Natural	Turf	2.16
0	15 Open	Natural	Turf	0.76
0	15 Open	Natural	Turf	2.96
0	15 Open	Natural	Turf	1.90
0	15 Open	Natural	Turf	2.13
0	15 Open	Natural	Turf	2.79
0	15 Open	Natural	Turf	1.17
0	15 Open	Natural	Turf	2.17
0	15 Open	Natural	Turf	1.38
0	15 Open	Natural	Turf	1.77
0	16 Open	Addition	Turf	2.77
0	16 Open	Addition	Turf	1.65
0	16 Open	Addition	Turf	1.92
0	16 Open	Addition	Turf	1.68
0	16 Open	Addition	Turf	1.89
0	16 Open	Addition	Turf	1.75
0	16 Open	Addition	Turf	1.75
0	16 Open	Addition	Turf	1.75
0	16 Open	Addition	Turf	2.29
0	16 Open	Addition	Turf	3.74
0	16 Open	Addition	Turf	0.00
0	16 Open	Addition	Turf	1.29

0	16	Open	Addition	Turf	1.54
0	16	Open	Addition	Turf	1.27
0	16	Open	Addition	Turf	1.14
0	16	Open	Addition	Turf	1.39
0	16	Open	Addition	Turf	0.87
0	16	Open	Addition	Turf	1.14
0	16	Open	Addition	Turf	1.53
0	16	Open	Addition	Turf	2.90
0	17	Open	Addition	Turf	2.48
0	17	Open	Addition	Turf	1.74
0	17	Open	Addition	Turf	4.36
0	17	Open	Addition	Turf	9.26
0	17	Open	Addition	Turf	3.58
0	17	Open	Addition	Turf	3.52
0	17	Open	Addition	Turf	11.09
0	17	Open	Addition	Turf	3.21
0	17	Open	Addition	Turf	10.00
0	17	Open	Addition	Turf	4.94
0	17	Open	Addition	Turf	2.62
0	17	Open	Addition	Turf	4.24
0	17	Open	Addition	Turf	2.96
0	17	Open	Addition	Turf	2.03
0	17	Open	Addition	Turf	2.46
0	17	Open	Addition	Turf	2.88
0	17	Open	Addition	Turf	2.40
0	17	Open	Addition	Turf	2.60
0	17	Open	Addition	Turf	2.71
0	17	Open	Addition	Turf	6.56
0	18	Open	Addition	Turf	1.24
0	18	Open	Addition	Turf	2.57
0	18	Open	Addition	Turf	1.78
0	18	Open	Addition	Turf	5.05
0	18	Open	Addition	Turf	2.03
0	18	Open	Addition	Turf	2.03
0	18	Open	Addition	Turf	1.73
0	18	Open	Addition	Turf	2.22
0	18	Open	Addition	Turf	3.79
0	18	Open	Addition	Turf	1.48
0	18	Open	Addition	Turf	2.49
0	18	Open	Addition	Turf	1.66
0	18	Open	Addition	Turf	6.37
0	18	Open	Addition	Turf	5.40
0	18	Open	Addition	Turf	2.36
0	18	Open	Addition	Turf	2.96
0	18	Open	Addition	Turf	3.24
0	18	Open	Addition	Turf	1.89
0	18	Open	Addition	Turf	2.09
0	18	Open	Addition	Turf	2.71
0	19	Full	Natural	Turf	6.21
0	19	Full	Natural	Turf	8.78
0	19	Full	Natural	Turf	4.94
0	19	Full	Natural	Turf	6.27
0	19	Full	Natural	Turf	3.92

0	19 Full	Natural	Turf	4.38
0	19 Full	Natural	Turf	4.64
0	19 Full	Natural	Turf	4.00
0	19 Full	Natural	Turf	4.49
0	19 Full	Natural	Turf	4.23
0	19 Full	Natural	Turf	3.72
0	19 Full	Natural	Turf	8.32
0	19 Full	Natural	Turf	9.57
0	19 Full	Natural	Turf	2.43
0	19 Full	Natural	Turf	2.25
0	19 Full	Natural	Turf	3.32
0	19 Full	Natural	Turf	4.22
0	19 Full	Natural	Turf	6.05
0	19 Full	Natural	Turf	2.58
0	19 Full	Natural	Turf	4.86
0	20 Full	Natural	Turf	3.23
0	20 Full	Natural	Turf	2.41
0	20 Full	Natural	Turf	4.10
0	20 Full	Natural	Turf	2.13
0	20 Full	Natural	Turf	1.79
0	20 Full	Natural	Turf	2.66
0	20 Full	Natural	Turf	1.91
0	20 Full	Natural	Turf	2.65
0	20 Full	Natural	Turf	3.16
0	20 Full	Natural	Turf	2.18
0	20 Full	Natural	Turf	2.13
0	20 Full	Natural	Turf	2.43
0	20 Full	Natural	Turf	3.64
0	20 Full	Natural	Turf	2.41
0	20 Full	Natural	Turf	3.00
0	20 Full	Natural	Turf	1.65
0	20 Full	Natural	Turf	1.77
0	20 Full	Natural	Turf	3.12
0	20 Full	Natural	Turf	4.22
0	20 Full	Natural	Turf	3.18
0	21 Full	Natural	Turf	2.40
0	21 Full	Natural	Turf	2.40
0	21 Full	Natural	Turf	3.11
0	21 Full	Natural	Turf	3.05
0	21 Full	Natural	Turf	2.53
0	21 Full	Natural	Turf	4.59
0	21 Full	Natural	Turf	4.14
0	21 Full	Natural	Turf	2.99
0	21 Full	Natural	Turf	2.46
0	21 Full	Natural	Turf	4.34
0	21 Full	Natural	Turf	1.22
0	21 Full	Natural	Turf	3.79
0	21 Full	Natural	Turf	2.21
0	21 Full	Natural	Turf	1.82
0	21 Full	Natural	Turf	3.33
0	21 Full	Natural	Turf	2.46
0	21 Full	Natural	Turf	2.84
0	21 Full	Natural	Turf	3.23

0	21	Full	Natural	Turf	2.91
0	21	Full	Natural	Turf	0.89
0	22	Full	Addition	Turf	1.82
0	22	Full	Addition	Turf	4.89
0	22	Full	Addition	Turf	3.45
0	22	Full	Addition	Turf	3.41
0	22	Full	Addition	Turf	2.86
0	22	Full	Addition	Turf	1.98
0	22	Full	Addition	Turf	3.04
0	22	Full	Addition	Turf	3.80
0	22	Full	Addition	Turf	2.73
0	22	Full	Addition	Turf	2.37
0	22	Full	Addition	Turf	1.64
0	22	Full	Addition	Turf	3.65
0	22	Full	Addition	Turf	2.94
0	22	Full	Addition	Turf	5.51
0	22	Full	Addition	Turf	2.56
0	22	Full	Addition	Turf	4.96
0	22	Full	Addition	Turf	3.48
0	22	Full	Addition	Turf	3.69
0	22	Full	Addition	Turf	3.41
0	22	Full	Addition	Turf	2.90
0	23	Full	Addition	Turf	3.80
0	23	Full	Addition	Turf	2.53
0	23	Full	Addition	Turf	3.42
0	23	Full	Addition	Turf	4.09
0	23	Full	Addition	Turf	3.37
0	23	Full	Addition	Turf	1.40
0	23	Full	Addition	Turf	4.58
0	23	Full	Addition	Turf	4.30
0	23	Full	Addition	Turf	4.05
0	23	Full	Addition	Turf	3.21
0	23	Full	Addition	Turf	3.64
0	23	Full	Addition	Turf	8.84
0	23	Full	Addition	Turf	3.84
0	23	Full	Addition	Turf	1.81
0	23	Full	Addition	Turf	2.34
0	23	Full	Addition	Turf	3.08
0	23	Full	Addition	Turf	4.41
0	23	Full	Addition	Turf	2.39
0	23	Full	Addition	Turf	3.55
0	23	Full	Addition	Turf	5.14
0	24	Full	Addition	Turf	2.42
0	24	Full	Addition	Turf	2.42
0	24	Full	Addition	Turf	2.10
0	24	Full	Addition	Turf	3.05
0	24	Full	Addition	Turf	2.78
0	24	Full	Addition	Turf	3.96
0	24	Full	Addition	Turf	1.79
0	24	Full	Addition	Turf	2.66
0	24	Full	Addition	Turf	3.89
0	24	Full	Addition	Turf	4.06
0	24	Full	Addition	Turf	3.84

0	24	Full	Addition	Turf	2.30
0	24	Full	Addition	Turf	1.23
0	24	Full	Addition	Turf	3.01
0	24	Full	Addition	Turf	1.95
0	24	Full	Addition	Turf	2.83
0	24	Full	Addition	Turf	3.11
0	24	Full	Addition	Turf	2.20
0	24	Full	Addition	Turf	3.67
0	24	Full	Addition	Turf	3.91
0	25	Half	Natural	Turf	2.05
0	25	Half	Natural	Turf	2.10
0	25	Half	Natural	Turf	3.18
0	25	Half	Natural	Turf	4.89
0	25	Half	Natural	Turf	4.47
0	25	Half	Natural	Turf	2.52
0	25	Half	Natural	Turf	2.33
0	25	Half	Natural	Turf	2.17
0	25	Half	Natural	Turf	2.33
0	25	Half	Natural	Turf	2.97
0	25	Half	Natural	Turf	2.50
0	25	Half	Natural	Turf	6.59
0	25	Half	Natural	Turf	2.96
0	25	Half	Natural	Turf	3.39
0	25	Half	Natural	Turf	2.22
0	25	Half	Natural	Turf	1.88
0	25	Half	Natural	Turf	3.73
0	25	Half	Natural	Turf	3.64
0	25	Half	Natural	Turf	3.15
0	25	Half	Natural	Turf	2.89
0	26	Half	Natural	Turf	2.91
0	26	Half	Natural	Turf	3.02
0	26	Half	Natural	Turf	3.31
0	26	Half	Natural	Turf	2.43
0	26	Half	Natural	Turf	3.61
0	26	Half	Natural	Turf	1.66
0	26	Half	Natural	Turf	1.90
0	26	Half	Natural	Turf	2.17
0	26	Half	Natural	Turf	2.11
0	26	Half	Natural	Turf	2.70
0	26	Half	Natural	Turf	2.04
0	26	Half	Natural	Turf	1.69
0	26	Half	Natural	Turf	2.70
0	26	Half	Natural	Turf	2.90
0	26	Half	Natural	Turf	5.31
0	26	Half	Natural	Turf	2.09
0	26	Half	Natural	Turf	4.42
0	26	Half	Natural	Turf	2.94
0	26	Half	Natural	Turf	2.20
0	26	Half	Natural	Turf	2.29
0	27	Half	Natural	Turf	2.81
0	27	Half	Natural	Turf	2.69
0	27	Half	Natural	Turf	2.18
0	27	Half	Natural	Turf	3.06

0	27	Half	Natural	Turf	3.10
0	27	Half	Natural	Turf	3.61
0	27	Half	Natural	Turf	3.17
0	27	Half	Natural	Turf	2.45
0	27	Half	Natural	Turf	1.37
0	27	Half	Natural	Turf	3.40
0	27	Half	Natural	Turf	3.41
0	27	Half	Natural	Turf	3.16
0	27	Half	Natural	Turf	1.83
0	27	Half	Natural	Turf	4.69
0	27	Half	Natural	Turf	2.18
0	27	Half	Natural	Turf	2.72
0	27	Half	Natural	Turf	3.97
0	27	Half	Natural	Turf	3.48
0	27	Half	Natural	Turf	3.50
0	27	Half	Natural	Turf	1.45
0	28	Half	Addition	Turf	3.09
0	28	Half	Addition	Turf	1.69
0	28	Half	Addition	Turf	2.68
0	28	Half	Addition	Turf	3.64
0	28	Half	Addition	Turf	2.30
0	28	Half	Addition	Turf	2.75
0	28	Half	Addition	Turf	4.32
0	28	Half	Addition	Turf	1.32
0	28	Half	Addition	Turf	3.14
0	28	Half	Addition	Turf	2.33
0	28	Half	Addition	Turf	4.59
0	28	Half	Addition	Turf	2.21
0	28	Half	Addition	Turf	2.28
0	28	Half	Addition	Turf	2.84
0	28	Half	Addition	Turf	2.09
0	28	Half	Addition	Turf	2.28
0	28	Half	Addition	Turf	2.49
0	28	Half	Addition	Turf	2.84
0	28	Half	Addition	Turf	2.47
0	28	Half	Addition	Turf	2.38
0	29	Half	Addition	Turf	3.53
0	29	Half	Addition	Turf	3.97
0	29	Half	Addition	Turf	1.24
0	29	Half	Addition	Turf	3.33
0	29	Half	Addition	Turf	2.84
0	29	Half	Addition	Turf	6.29
0	29	Half	Addition	Turf	3.00
0	29	Half	Addition	Turf	2.41
0	29	Half	Addition	Turf	1.20
0	29	Half	Addition	Turf	1.87
0	29	Half	Addition	Turf	3.76
0	29	Half	Addition	Turf	4.95
0	29	Half	Addition	Turf	3.15
0	29	Half	Addition	Turf	5.89
0	29	Half	Addition	Turf	4.69
0	29	Half	Addition	Turf	3.01
0	29	Half	Addition	Turf	4.45

0	29	Half	Addition	Turf	2.80
0	29	Half	Addition	Turf	2.50
0	29	Half	Addition	Turf	2.69
0	30	Half	Addition	Turf	1.39
0	30	Half	Addition	Turf	2.71
0	30	Half	Addition	Turf	1.81
0	30	Half	Addition	Turf	2.54
0	30	Half	Addition	Turf	3.40
0	30	Half	Addition	Turf	2.42
0	30	Half	Addition	Turf	4.64
0	30	Half	Addition	Turf	2.25
0	30	Half	Addition	Turf	2.63
0	30	Half	Addition	Turf	2.40
0	30	Half	Addition	Turf	2.03
0	30	Half	Addition	Turf	2.13
0	30	Half	Addition	Turf	2.93
0	30	Half	Addition	Turf	4.55
0	30	Half	Addition	Turf	1.45
0	30	Half	Addition	Turf	2.28
0	30	Half	Addition	Turf	2.20
0	30	Half	Addition	Turf	1.53
0	30	Half	Addition	Turf	2.43
0	30	Half	Addition	Turf	2.16
0	31	Open	Natural	Turf	5.92
0	31	Open	Natural	Turf	3.78
0	31	Open	Natural	Turf	4.29
0	31	Open	Natural	Turf	3.05
0	31	Open	Natural	Turf	2.74
0	31	Open	Natural	Turf	4.71
0	31	Open	Natural	Turf	3.41
0	31	Open	Natural	Turf	2.03
0	31	Open	Natural	Turf	4.29
0	31	Open	Natural	Turf	3.69
0	31	Open	Natural	Turf	1.17
0	31	Open	Natural	Turf	4.28
0	31	Open	Natural	Turf	3.37
0	31	Open	Natural	Turf	1.73
0	31	Open	Natural	Turf	3.96
0	31	Open	Natural	Turf	3.54
0	31	Open	Natural	Turf	3.58
0	31	Open	Natural	Turf	2.83
0	31	Open	Natural	Turf	1.81
0	31	Open	Natural	Turf	4.66
0	32	Open	Natural	Turf	2.17
0	32	Open	Natural	Turf	7.52
0	32	Open	Natural	Turf	3.13
0	32	Open	Natural	Turf	2.05
0	32	Open	Natural	Turf	2.65
0	32	Open	Natural	Turf	2.40
0	32	Open	Natural	Turf	1.69
0	32	Open	Natural	Turf	2.89
0	32	Open	Natural	Turf	3.05
0	32	Open	Natural	Turf	3.37

0	32	Open	Natural	Turf	2.13
0	32	Open	Natural	Turf	2.37
0	32	Open	Natural	Turf	2.96
0	32	Open	Natural	Turf	3.54
0	32	Open	Natural	Turf	2.60
0	32	Open	Natural	Turf	2.40
0	32	Open	Natural	Turf	4.32
0	32	Open	Natural	Turf	2.93
0	32	Open	Natural	Turf	9.82
0	32	Open	Natural	Turf	3.63
0	33	Open	Natural	Turf	1.27
0	33	Open	Natural	Turf	2.44
0	33	Open	Natural	Turf	2.38
0	33	Open	Natural	Turf	2.21
0	33	Open	Natural	Turf	3.00
0	33	Open	Natural	Turf	2.52
0	33	Open	Natural	Turf	2.75
0	33	Open	Natural	Turf	1.67
0	33	Open	Natural	Turf	2.29
0	33	Open	Natural	Turf	2.03
0	33	Open	Natural	Turf	2.57
0	33	Open	Natural	Turf	4.58
0	33	Open	Natural	Turf	2.64
0	33	Open	Natural	Turf	1.49
0	33	Open	Natural	Turf	1.43
0	33	Open	Natural	Turf	2.09
0	33	Open	Natural	Turf	1.19
0	33	Open	Natural	Turf	1.51
0	33	Open	Natural	Turf	2.26
0	33	Open	Natural	Turf	2.17
0	34	Open	Addition	Turf	3.95
0	34	Open	Addition	Turf	1.69
0	34	Open	Addition	Turf	5.94
0	34	Open	Addition	Turf	3.60
0	34	Open	Addition	Turf	1.51
0	34	Open	Addition	Turf	3.09
0	34	Open	Addition	Turf	5.73
0	34	Open	Addition	Turf	4.31
0	34	Open	Addition	Turf	7.54
0	34	Open	Addition	Turf	3.70
0	34	Open	Addition	Turf	3.83
0	34	Open	Addition	Turf	2.87
0	34	Open	Addition	Turf	3.31
0	34	Open	Addition	Turf	3.14
0	34	Open	Addition	Turf	4.97
0	34	Open	Addition	Turf	3.91
0	34	Open	Addition	Turf	3.21
0	34	Open	Addition	Turf	3.02
0	34	Open	Addition	Turf	3.02
0	34	Open	Addition	Turf	4.70
0	35	Open	Addition	Turf	2.09
0	35	Open	Addition	Turf	1.94
0	35	Open	Addition	Turf	3.69

0	35	Open	Addition	Turf	1.91
0	35	Open	Addition	Turf	4.56
0	35	Open	Addition	Turf	2.92
0	35	Open	Addition	Turf	2.79
0	35	Open	Addition	Turf	4.04
0	35	Open	Addition	Turf	2.41
0	35	Open	Addition	Turf	4.46
0	35	Open	Addition	Turf	2.08
0	35	Open	Addition	Turf	2.94
0	35	Open	Addition	Turf	3.47
0	35	Open	Addition	Turf	2.21
0	35	Open	Addition	Turf	2.49
0	35	Open	Addition	Turf	2.33
0	35	Open	Addition	Turf	1.19
0	35	Open	Addition	Turf	6.30
0	35	Open	Addition	Turf	2.37
0	35	Open	Addition	Turf	3.68
0	36	Open	Addition	Turf	1.39
0	36	Open	Addition	Turf	4.05
0	36	Open	Addition	Turf	2.18
0	36	Open	Addition	Turf	2.25
0	36	Open	Addition	Turf	1.83
0	36	Open	Addition	Turf	1.91
0	36	Open	Addition	Turf	3.15
0	36	Open	Addition	Turf	1.93
0	36	Open	Addition	Turf	1.34
0	36	Open	Addition	Turf	2.02
0	36	Open	Addition	Turf	1.27
0	36	Open	Addition	Turf	2.17
0	36	Open	Addition	Turf	1.01
0	36	Open	Addition	Turf	1.72
0	36	Open	Addition	Turf	2.37
0	36	Open	Addition	Turf	3.62
0	36	Open	Addition	Turf	2.56
0	36	Open	Addition	Turf	0.86
0	36	Open	Addition	Turf	1.20
0	36	Open	Addition	Turf	0.57
1	1	Full	Natural	Turf	3.67
1	1	Full	Natural	Turf	3.49
1	1	Full	Natural	Turf	4.79
1	1	Full	Natural	Turf	2.32
1	1	Full	Natural	Turf	1.95
1	1	Full	Natural	Turf	2.24
1	1	Full	Natural	Turf	3.20
1	1	Full	Natural	Turf	4.99
1	1	Full	Natural	Turf	2.03
1	1	Full	Natural	Turf	3.41
1	1	Full	Natural	Turf	3.61
1	1	Full	Natural	Turf	3.00
1	1	Full	Natural	Turf	4.18
1	1	Full	Natural	Turf	2.00
1	1	Full	Natural	Turf	4.10
1	1	Full	Natural	Turf	2.14

1	1	Full	Natural	Turf	2.48
1	1	Full	Natural	Turf	2.84
1	1	Full	Natural	Turf	2.45
1	1	Full	Natural	Turf	2.35
1	2	Full	Natural	Turf	4.36
1	2	Full	Natural	Turf	2.23
1	2	Full	Natural	Turf	0.00
1	2	Full	Natural	Turf	3.64
1	2	Full	Natural	Turf	4.98
1	2	Full	Natural	Turf	3.69
1	2	Full	Natural	Turf	2.86
1	2	Full	Natural	Turf	2.36
1	2	Full	Natural	Turf	3.02
1	2	Full	Natural	Turf	5.55
1	2	Full	Natural	Turf	4.08
1	2	Full	Natural	Turf	7.68
1	2	Full	Natural	Turf	3.01
1	2	Full	Natural	Turf	3.05
1	2	Full	Natural	Turf	3.63
1	2	Full	Natural	Turf	4.59
1	2	Full	Natural	Turf	3.83
1	2	Full	Natural	Turf	2.24
1	2	Full	Natural	Turf	2.20
1	2	Full	Natural	Turf	1.49
1	3	Full	Natural	Turf	2.85
1	3	Full	Natural	Turf	2.92
1	3	Full	Natural	Turf	3.14
1	3	Full	Natural	Turf	3.60
1	3	Full	Natural	Turf	3.52
1	3	Full	Natural	Turf	3.48
1	3	Full	Natural	Turf	2.78
1	3	Full	Natural	Turf	2.93
1	3	Full	Natural	Turf	3.82
1	3	Full	Natural	Turf	1.91
1	3	Full	Natural	Turf	4.31
1	3	Full	Natural	Turf	1.76
1	3	Full	Natural	Turf	2.70
1	3	Full	Natural	Turf	2.88
1	3	Full	Natural	Turf	3.17
1	3	Full	Natural	Turf	3.68
1	3	Full	Natural	Turf	4.25
1	3	Full	Natural	Turf	1.58
1	3	Full	Natural	Turf	3.46
1	3	Full	Natural	Turf	2.32
1	4	Full	Addition	Turf	5.23
1	4	Full	Addition	Turf	3.84
1	4	Full	Addition	Turf	4.50
1	4	Full	Addition	Turf	3.40
1	4	Full	Addition	Turf	4.12
1	4	Full	Addition	Turf	3.06
1	4	Full	Addition	Turf	7.62
1	4	Full	Addition	Turf	2.17
1	4	Full	Addition	Turf	7.49

1	4	Full	Addition	Turf	1.87
1	4	Full	Addition	Turf	3.47
1	4	Full	Addition	Turf	2.81
1	4	Full	Addition	Turf	3.08
1	4	Full	Addition	Turf	3.03
1	4	Full	Addition	Turf	2.38
1	4	Full	Addition	Turf	2.02
1	4	Full	Addition	Turf	2.10
1	4	Full	Addition	Turf	4.26
1	4	Full	Addition	Turf	3.06
1	4	Full	Addition	Turf	4.11
1	5	Full	Addition	Turf	2.56
1	5	Full	Addition	Turf	2.78
1	5	Full	Addition	Turf	3.14
1	5	Full	Addition	Turf	2.25
1	5	Full	Addition	Turf	1.63
1	5	Full	Addition	Turf	3.37
1	5	Full	Addition	Turf	3.53
1	5	Full	Addition	Turf	2.85
1	5	Full	Addition	Turf	2.60
1	5	Full	Addition	Turf	1.41
1	5	Full	Addition	Turf	5.08
1	5	Full	Addition	Turf	4.75
1	5	Full	Addition	Turf	2.04
1	5	Full	Addition	Turf	0.00
1	5	Full	Addition	Turf	2.01
1	5	Full	Addition	Turf	2.46
1	5	Full	Addition	Turf	8.39
1	5	Full	Addition	Turf	4.45
1	5	Full	Addition	Turf	3.87
1	5	Full	Addition	Turf	3.63
1	6	Full	Addition	Turf	2.42
1	6	Full	Addition	Turf	1.49
1	6	Full	Addition	Turf	3.64
1	6	Full	Addition	Turf	0.00
1	6	Full	Addition	Turf	1.41
1	6	Full	Addition	Turf	1.95
1	6	Full	Addition	Turf	2.45
1	6	Full	Addition	Turf	5.46
1	6	Full	Addition	Turf	1.73
1	6	Full	Addition	Turf	2.78
1	6	Full	Addition	Turf	8.04
1	6	Full	Addition	Turf	3.25
1	6	Full	Addition	Turf	2.81
1	6	Full	Addition	Turf	4.36
1	6	Full	Addition	Turf	5.25
1	6	Full	Addition	Turf	5.26
1	6	Full	Addition	Turf	2.20
1	6	Full	Addition	Turf	4.43
1	6	Full	Addition	Turf	0.00
1	6	Full	Addition	Turf	0.00
1	7	Half	Natural	Turf	2.24
1	7	Half	Natural	Turf	3.30

1	7	Half	Natural	Turf	3.50
1	7	Half	Natural	Turf	2.69
1	7	Half	Natural	Turf	5.67
1	7	Half	Natural	Turf	1.56
1	7	Half	Natural	Turf	9.98
1	7	Half	Natural	Turf	2.42
1	7	Half	Natural	Turf	1.97
1	7	Half	Natural	Turf	10.37
1	7	Half	Natural	Turf	4.08
1	7	Half	Natural	Turf	1.81
1	7	Half	Natural	Turf	2.41
1	7	Half	Natural	Turf	2.00
1	7	Half	Natural	Turf	3.59
1	7	Half	Natural	Turf	4.05
1	7	Half	Natural	Turf	4.20
1	7	Half	Natural	Turf	4.05
1	7	Half	Natural	Turf	1.58
1	7	Half	Natural	Turf	3.95
1	8	Half	Natural	Turf	2.49
1	8	Half	Natural	Turf	5.05
1	8	Half	Natural	Turf	1.43
1	8	Half	Natural	Turf	1.57
1	8	Half	Natural	Turf	1.75
1	8	Half	Natural	Turf	2.93
1	8	Half	Natural	Turf	2.14
1	8	Half	Natural	Turf	2.05
1	8	Half	Natural	Turf	5.20
1	8	Half	Natural	Turf	2.05
1	8	Half	Natural	Turf	2.64
1	8	Half	Natural	Turf	2.30
1	8	Half	Natural	Turf	5.08
1	8	Half	Natural	Turf	1.73
1	8	Half	Natural	Turf	3.61
1	8	Half	Natural	Turf	1.43
1	8	Half	Natural	Turf	2.30
1	8	Half	Natural	Turf	2.50
1	8	Half	Natural	Turf	2.67
1	8	Half	Natural	Turf	1.78
1	9	Half	Natural	Turf	3.30
1	9	Half	Natural	Turf	4.05
1	9	Half	Natural	Turf	2.64
1	9	Half	Natural	Turf	1.64
1	9	Half	Natural	Turf	2.91
1	9	Half	Natural	Turf	3.98
1	9	Half	Natural	Turf	3.28
1	9	Half	Natural	Turf	2.30
1	9	Half	Natural	Turf	3.07
1	9	Half	Natural	Turf	2.21
1	9	Half	Natural	Turf	1.09
1	9	Half	Natural	Turf	2.22
1	9	Half	Natural	Turf	3.37
1	9	Half	Natural	Turf	1.71
1	9	Half	Natural	Turf	2.38

1	9	Half	Natural	Turf	1.29
1	9	Half	Natural	Turf	2.97
1	9	Half	Natural	Turf	2.38
1	9	Half	Natural	Turf	2.83
1	9	Half	Natural	Turf	2.63
1	10	Half	Addition	Turf	3.63
1	10	Half	Addition	Turf	1.86
1	10	Half	Addition	Turf	2.09
1	10	Half	Addition	Turf	3.52
1	10	Half	Addition	Turf	1.03
1	10	Half	Addition	Turf	3.10
1	10	Half	Addition	Turf	3.59
1	10	Half	Addition	Turf	0.00
1	10	Half	Addition	Turf	6.12
1	10	Half	Addition	Turf	2.41
1	10	Half	Addition	Turf	2.44
1	10	Half	Addition	Turf	1.79
1	10	Half	Addition	Turf	2.08
1	10	Half	Addition	Turf	5.00
1	10	Half	Addition	Turf	1.52
1	10	Half	Addition	Turf	1.78
1	10	Half	Addition	Turf	2.32
1	10	Half	Addition	Turf	1.27
1	10	Half	Addition	Turf	2.89
1	10	Half	Addition	Turf	3.74
1	11	Half	Addition	Turf	2.83
1	11	Half	Addition	Turf	3.95
1	11	Half	Addition	Turf	1.78
1	11	Half	Addition	Turf	4.07
1	11	Half	Addition	Turf	7.27
1	11	Half	Addition	Turf	4.19
1	11	Half	Addition	Turf	7.19
1	11	Half	Addition	Turf	4.78
1	11	Half	Addition	Turf	2.61
1	11	Half	Addition	Turf	2.63
1	11	Half	Addition	Turf	6.80
1	11	Half	Addition	Turf	2.30
1	11	Half	Addition	Turf	3.78
1	11	Half	Addition	Turf	2.10
1	11	Half	Addition	Turf	2.73
1	11	Half	Addition	Turf	2.39
1	11	Half	Addition	Turf	2.86
1	11	Half	Addition	Turf	1.95
1	11	Half	Addition	Turf	2.22
1	11	Half	Addition	Turf	3.94
1	12	Half	Addition	Turf	1.24
1	12	Half	Addition	Turf	1.21
1	12	Half	Addition	Turf	2.74
1	12	Half	Addition	Turf	1.38
1	12	Half	Addition	Turf	1.78
1	12	Half	Addition	Turf	3.98
1	12	Half	Addition	Turf	5.49
1	12	Half	Addition	Turf	2.42

1	12	Half	Addition	Turf	2.09
1	12	Half	Addition	Turf	1.43
1	12	Half	Addition	Turf	3.92
1	12	Half	Addition	Turf	2.53
1	12	Half	Addition	Turf	4.12
1	12	Half	Addition	Turf	2.15
1	12	Half	Addition	Turf	3.53
1	12	Half	Addition	Turf	3.06
1	12	Half	Addition	Turf	5.70
1	12	Half	Addition	Turf	2.03
1	12	Half	Addition	Turf	2.32
1	12	Half	Addition	Turf	2.94
1	13	Open	Natural	Turf	2.47
1	13	Open	Natural	Turf	4.14
1	13	Open	Natural	Turf	3.27
1	13	Open	Natural	Turf	2.06
1	13	Open	Natural	Turf	2.96
1	13	Open	Natural	Turf	2.91
1	13	Open	Natural	Turf	4.90
1	13	Open	Natural	Turf	2.32
1	13	Open	Natural	Turf	4.81
1	13	Open	Natural	Turf	3.07
1	13	Open	Natural	Turf	2.36
1	13	Open	Natural	Turf	2.23
1	13	Open	Natural	Turf	2.02
1	13	Open	Natural	Turf	2.10
1	13	Open	Natural	Turf	2.49
1	13	Open	Natural	Turf	1.94
1	13	Open	Natural	Turf	2.81
1	13	Open	Natural	Turf	2.95
1	13	Open	Natural	Turf	1.16
1	13	Open	Natural	Turf	5.17
1	14	Open	Natural	Turf	3.53
1	14	Open	Natural	Turf	3.23
1	14	Open	Natural	Turf	3.80
1	14	Open	Natural	Turf	1.50
1	14	Open	Natural	Turf	1.79
1	14	Open	Natural	Turf	2.46
1	14	Open	Natural	Turf	2.74
1	14	Open	Natural	Turf	4.39
1	14	Open	Natural	Turf	1.58
1	14	Open	Natural	Turf	2.58
1	14	Open	Natural	Turf	2.13
1	14	Open	Natural	Turf	2.58
1	14	Open	Natural	Turf	5.82
1	14	Open	Natural	Turf	2.50
1	14	Open	Natural	Turf	2.39
1	14	Open	Natural	Turf	2.68
1	14	Open	Natural	Turf	3.54
1	14	Open	Natural	Turf	1.91
1	14	Open	Natural	Turf	2.82
1	14	Open	Natural	Turf	2.85
1	15	Open	Natural	Turf	2.20

1	15	Open	Natural	Turf	3.18
1	15	Open	Natural	Turf	1.64
1	15	Open	Natural	Turf	2.16
1	15	Open	Natural	Turf	2.23
1	15	Open	Natural	Turf	2.92
1	15	Open	Natural	Turf	4.32
1	15	Open	Natural	Turf	2.62
1	15	Open	Natural	Turf	4.28
1	15	Open	Natural	Turf	1.93
1	15	Open	Natural	Turf	2.48
1	15	Open	Natural	Turf	1.79
1	15	Open	Natural	Turf	2.04
1	15	Open	Natural	Turf	3.16
1	15	Open	Natural	Turf	2.38
1	15	Open	Natural	Turf	3.35
1	15	Open	Natural	Turf	1.83
1	15	Open	Natural	Turf	2.58
1	15	Open	Natural	Turf	2.43
1	15	Open	Natural	Turf	3.76
1	16	Open	Addition	Turf	1.38
1	16	Open	Addition	Turf	1.21
1	16	Open	Addition	Turf	1.29
1	16	Open	Addition	Turf	4.62
1	16	Open	Addition	Turf	4.56
1	16	Open	Addition	Turf	5.27
1	16	Open	Addition	Turf	2.69
1	16	Open	Addition	Turf	3.64
1	16	Open	Addition	Turf	2.63
1	16	Open	Addition	Turf	5.13
1	16	Open	Addition	Turf	2.98
1	16	Open	Addition	Turf	1.66
1	16	Open	Addition	Turf	2.92
1	16	Open	Addition	Turf	5.12
1	16	Open	Addition	Turf	3.94
1	16	Open	Addition	Turf	4.98
1	16	Open	Addition	Turf	1.96
1	16	Open	Addition	Turf	2.40
1	16	Open	Addition	Turf	2.12
1	16	Open	Addition	Turf	2.97
1	17	Open	Addition	Turf	4.45
1	17	Open	Addition	Turf	5.79
1	17	Open	Addition	Turf	3.18
1	17	Open	Addition	Turf	7.14
1	17	Open	Addition	Turf	4.93
1	17	Open	Addition	Turf	3.92
1	17	Open	Addition	Turf	10.18
1	17	Open	Addition	Turf	3.58
1	17	Open	Addition	Turf	4.36
1	17	Open	Addition	Turf	3.81
1	17	Open	Addition	Turf	4.60
1	17	Open	Addition	Turf	6.12
1	17	Open	Addition	Turf	1.97
1	17	Open	Addition	Turf	5.37

1	17	Open	Addition	Turf	4.92
1	17	Open	Addition	Turf	4.05
1	17	Open	Addition	Turf	8.09
1	17	Open	Addition	Turf	1.36
1	17	Open	Addition	Turf	0.91
1	17	Open	Addition	Turf	2.60
1	18	Open	Addition	Turf	4.98
1	18	Open	Addition	Turf	1.17
1	18	Open	Addition	Turf	3.27
1	18	Open	Addition	Turf	1.90
1	18	Open	Addition	Turf	2.87
1	18	Open	Addition	Turf	4.18
1	18	Open	Addition	Turf	4.67
1	18	Open	Addition	Turf	6.45
1	18	Open	Addition	Turf	7.72
1	18	Open	Addition	Turf	5.17
1	18	Open	Addition	Turf	5.30
1	18	Open	Addition	Turf	1.35
1	18	Open	Addition	Turf	2.17
1	18	Open	Addition	Turf	5.74
1	18	Open	Addition	Turf	5.29
1	18	Open	Addition	Turf	5.15
1	18	Open	Addition	Turf	2.34
1	18	Open	Addition	Turf	1.95
1	18	Open	Addition	Turf	5.30
1	18	Open	Addition	Turf	2.39
1	19	Cage	Natural	Turf	2.33
1	19	Cage	Natural	Turf	5.12
1	19	Cage	Natural	Turf	4.76
1	19	Cage	Natural	Turf	2.50
1	19	Cage	Natural	Turf	10.95
1	19	Cage	Natural	Turf	5.96
1	19	Cage	Natural	Turf	5.54
1	19	Cage	Natural	Turf	3.90
1	19	Cage	Natural	Turf	6.40
1	19	Cage	Natural	Turf	3.17
1	19	Cage	Natural	Turf	11.16
1	19	Cage	Natural	Turf	7.40
1	19	Cage	Natural	Turf	4.48
1	19	Cage	Natural	Turf	4.84
1	19	Cage	Natural	Turf	4.19
1	19	Cage	Natural	Turf	7.27
1	19	Cage	Natural	Turf	5.19
1	19	Cage	Natural	Turf	4.18
1	19	Cage	Natural	Turf	5.93
1	19	Cage	Natural	Turf	3.24
1	20	Cage	Natural	Turf	2.98
1	20	Cage	Natural	Turf	2.03
1	20	Cage	Natural	Turf	4.89
1	20	Cage	Natural	Turf	2.95
1	20	Cage	Natural	Turf	3.72
1	20	Cage	Natural	Turf	6.86
1	20	Cage	Natural	Turf	5.12

1	20	Cage	Natural	Turf	5.30
1	20	Cage	Natural	Turf	4.39
1	20	Cage	Natural	Turf	4.61
1	20	Cage	Natural	Turf	2.50
1	20	Cage	Natural	Turf	2.59
1	20	Cage	Natural	Turf	2.93
1	20	Cage	Natural	Turf	3.44
1	20	Cage	Natural	Turf	3.25
1	20	Cage	Natural	Turf	1.80
1	20	Cage	Natural	Turf	1.84
1	20	Cage	Natural	Turf	5.57
1	20	Cage	Natural	Turf	4.89
1	20	Cage	Natural	Turf	5.71
1	21	Cage	Natural	Turf	2.82
1	21	Cage	Natural	Turf	8.58
1	21	Cage	Natural	Turf	4.50
1	21	Cage	Natural	Turf	3.15
1	21	Cage	Natural	Turf	3.61
1	21	Cage	Natural	Turf	4.52
1	21	Cage	Natural	Turf	3.91
1	21	Cage	Natural	Turf	3.26
1	21	Cage	Natural	Turf	5.65
1	21	Cage	Natural	Turf	3.80
1	21	Cage	Natural	Turf	3.65
1	21	Cage	Natural	Turf	4.26
1	21	Cage	Natural	Turf	3.00
1	21	Cage	Natural	Turf	4.99
1	21	Cage	Natural	Turf	4.94
1	21	Cage	Natural	Turf	4.32
1	21	Cage	Natural	Turf	3.46
1	21	Cage	Natural	Turf	4.17
1	21	Cage	Natural	Turf	2.88
1	21	Cage	Natural	Turf	2.85
1	22	Cage	Addition	Turf	2.23
1	22	Cage	Addition	Turf	3.44
1	22	Cage	Addition	Turf	5.13
1	22	Cage	Addition	Turf	4.54
1	22	Cage	Addition	Turf	5.38
1	22	Cage	Addition	Turf	8.04
1	22	Cage	Addition	Turf	8.99
1	22	Cage	Addition	Turf	7.86
1	22	Cage	Addition	Turf	2.78
1	22	Cage	Addition	Turf	2.59
1	22	Cage	Addition	Turf	3.48
1	22	Cage	Addition	Turf	3.21
1	22	Cage	Addition	Turf	4.37
1	22	Cage	Addition	Turf	4.39
1	22	Cage	Addition	Turf	3.60
1	22	Cage	Addition	Turf	5.26
1	22	Cage	Addition	Turf	3.65
1	22	Cage	Addition	Turf	4.75
1	22	Cage	Addition	Turf	4.88
1	22	Cage	Addition	Turf	3.32

1	23	Cage	Addition	Turf	5.10
1	23	Cage	Addition	Turf	16.83
1	23	Cage	Addition	Turf	4.64
1	23	Cage	Addition	Turf	4.20
1	23	Cage	Addition	Turf	2.49
1	23	Cage	Addition	Turf	4.71
1	23	Cage	Addition	Turf	3.83
1	23	Cage	Addition	Turf	3.83
1	23	Cage	Addition	Turf	3.94
1	23	Cage	Addition	Turf	6.87
1	23	Cage	Addition	Turf	3.45
1	23	Cage	Addition	Turf	1.86
1	23	Cage	Addition	Turf	5.36
1	23	Cage	Addition	Turf	5.44
1	23	Cage	Addition	Turf	4.49
1	23	Cage	Addition	Turf	3.01
1	23	Cage	Addition	Turf	3.52
1	23	Cage	Addition	Turf	6.94
1	23	Cage	Addition	Turf	8.68
1	23	Cage	Addition	Turf	4.50
1	24	Cage	Addition	Turf	5.03
1	24	Cage	Addition	Turf	7.20
1	24	Cage	Addition	Turf	3.26
1	24	Cage	Addition	Turf	3.82
1	24	Cage	Addition	Turf	5.54
1	24	Cage	Addition	Turf	9.57
1	24	Cage	Addition	Turf	4.90
1	24	Cage	Addition	Turf	4.19
1	24	Cage	Addition	Turf	6.62
1	24	Cage	Addition	Turf	3.01
1	24	Cage	Addition	Turf	2.63
1	24	Cage	Addition	Turf	4.45
1	24	Cage	Addition	Turf	2.27
1	24	Cage	Addition	Turf	3.14
1	24	Cage	Addition	Turf	6.80
1	24	Cage	Addition	Turf	3.99
1	24	Cage	Addition	Turf	2.81
1	24	Cage	Addition	Turf	5.90
1	24	Cage	Addition	Turf	5.58
1	24	Cage	Addition	Turf	2.58
1	25	Half	Natural	Turf	3.27
1	25	Half	Natural	Turf	5.51
1	25	Half	Natural	Turf	4.41
1	25	Half	Natural	Turf	6.54
1	25	Half	Natural	Turf	2.75
1	25	Half	Natural	Turf	7.97
1	25	Half	Natural	Turf	4.80
1	25	Half	Natural	Turf	3.87
1	25	Half	Natural	Turf	3.45
1	25	Half	Natural	Turf	2.97
1	25	Half	Natural	Turf	3.69
1	25	Half	Natural	Turf	2.38
1	25	Half	Natural	Turf	1.81

1	25	Half	Natural	Turf	3.57
1	25	Half	Natural	Turf	2.85
1	25	Half	Natural	Turf	3.32
1	25	Half	Natural	Turf	4.22
1	25	Half	Natural	Turf	5.65
1	25	Half	Natural	Turf	5.74
1	25	Half	Natural	Turf	4.29
1	26	Half	Natural	Turf	4.86
1	26	Half	Natural	Turf	4.53
1	26	Half	Natural	Turf	3.66
1	26	Half	Natural	Turf	2.61
1	26	Half	Natural	Turf	2.39
1	26	Half	Natural	Turf	2.09
1	26	Half	Natural	Turf	7.62
1	26	Half	Natural	Turf	3.00
1	26	Half	Natural	Turf	3.02
1	26	Half	Natural	Turf	2.27
1	26	Half	Natural	Turf	3.98
1	26	Half	Natural	Turf	2.80
1	26	Half	Natural	Turf	3.01
1	26	Half	Natural	Turf	4.08
1	26	Half	Natural	Turf	2.02
1	26	Half	Natural	Turf	1.71
1	26	Half	Natural	Turf	3.66
1	26	Half	Natural	Turf	1.98
1	26	Half	Natural	Turf	1.62
1	26	Half	Natural	Turf	2.12
1	27	Half	Natural	Turf	2.46
1	27	Half	Natural	Turf	4.58
1	27	Half	Natural	Turf	3.47
1	27	Half	Natural	Turf	4.43
1	27	Half	Natural	Turf	2.96
1	27	Half	Natural	Turf	3.16
1	27	Half	Natural	Turf	4.27
1	27	Half	Natural	Turf	4.00
1	27	Half	Natural	Turf	1.83
1	27	Half	Natural	Turf	3.83
1	27	Half	Natural	Turf	3.36
1	27	Half	Natural	Turf	4.17
1	27	Half	Natural	Turf	2.44
1	27	Half	Natural	Turf	3.71
1	27	Half	Natural	Turf	3.05
1	27	Half	Natural	Turf	1.75
1	27	Half	Natural	Turf	4.79
1	27	Half	Natural	Turf	4.30
1	27	Half	Natural	Turf	3.33
1	27	Half	Natural	Turf	2.68
1	28	Half	Addition	Turf	3.03
1	28	Half	Addition	Turf	3.13
1	28	Half	Addition	Turf	4.21
1	28	Half	Addition	Turf	2.02
1	28	Half	Addition	Turf	4.69
1	28	Half	Addition	Turf	5.69

1	28	Half	Addition	Turf	2.73
1	28	Half	Addition	Turf	3.35
1	28	Half	Addition	Turf	3.03
1	28	Half	Addition	Turf	1.64
1	28	Half	Addition	Turf	3.14
1	28	Half	Addition	Turf	3.48
1	28	Half	Addition	Turf	6.26
1	28	Half	Addition	Turf	2.18
1	28	Half	Addition	Turf	2.80
1	28	Half	Addition	Turf	1.89
1	28	Half	Addition	Turf	2.57
1	28	Half	Addition	Turf	2.96
1	28	Half	Addition	Turf	3.33
1	28	Half	Addition	Turf	2.65
1	29	Half	Addition	Turf	4.56
1	29	Half	Addition	Turf	3.11
1	29	Half	Addition	Turf	3.26
1	29	Half	Addition	Turf	1.91
1	29	Half	Addition	Turf	2.27
1	29	Half	Addition	Turf	8.47
1	29	Half	Addition	Turf	5.14
1	29	Half	Addition	Turf	6.31
1	29	Half	Addition	Turf	4.20
1	29	Half	Addition	Turf	4.13
1	29	Half	Addition	Turf	3.97
1	29	Half	Addition	Turf	3.45
1	29	Half	Addition	Turf	5.38
1	29	Half	Addition	Turf	2.98
1	29	Half	Addition	Turf	4.47
1	29	Half	Addition	Turf	5.40
1	29	Half	Addition	Turf	4.11
1	29	Half	Addition	Turf	2.85
1	29	Half	Addition	Turf	4.47
1	29	Half	Addition	Turf	4.03
1	30	Half	Addition	Turf	2.48
1	30	Half	Addition	Turf	4.41
1	30	Half	Addition	Turf	2.68
1	30	Half	Addition	Turf	2.56
1	30	Half	Addition	Turf	0.00
1	30	Half	Addition	Turf	3.02
1	30	Half	Addition	Turf	3.90
1	30	Half	Addition	Turf	3.45
1	30	Half	Addition	Turf	2.87
1	30	Half	Addition	Turf	7.19
1	30	Half	Addition	Turf	4.55
1	30	Half	Addition	Turf	5.13
1	30	Half	Addition	Turf	5.42
1	30	Half	Addition	Turf	6.05
1	30	Half	Addition	Turf	5.52
1	30	Half	Addition	Turf	5.05
1	30	Half	Addition	Turf	4.77
1	30	Half	Addition	Turf	6.36
1	30	Half	Addition	Turf	4.68

1	30 Half	Addition	Turf	23.32
1	31 Open	Natural	Turf	3.54
1	31 Open	Natural	Turf	2.22
1	31 Open	Natural	Turf	3.67
1	31 Open	Natural	Turf	0.00
1	31 Open	Natural	Turf	3.07
1	31 Open	Natural	Turf	3.12
1	31 Open	Natural	Turf	4.83
1	31 Open	Natural	Turf	4.00
1	31 Open	Natural	Turf	5.87
1	31 Open	Natural	Turf	3.72
1	31 Open	Natural	Turf	2.94
1	31 Open	Natural	Turf	4.83
1	31 Open	Natural	Turf	4.90
1	31 Open	Natural	Turf	4.08
1	31 Open	Natural	Turf	3.98
1	31 Open	Natural	Turf	5.23
1	31 Open	Natural	Turf	1.83
1	31 Open	Natural	Turf	4.87
1	31 Open	Natural	Turf	6.92
1	31 Open	Natural	Turf	3.06
1	32 Open	Natural	Turf	4.43
1	32 Open	Natural	Turf	2.57
1	32 Open	Natural	Turf	3.08
1	32 Open	Natural	Turf	3.29
1	32 Open	Natural	Turf	3.60
1	32 Open	Natural	Turf	3.79
1	32 Open	Natural	Turf	3.54
1	32 Open	Natural	Turf	2.99
1	32 Open	Natural	Turf	4.09
1	32 Open	Natural	Turf	2.62
1	32 Open	Natural	Turf	2.37
1	32 Open	Natural	Turf	2.84
1	32 Open	Natural	Turf	4.06
1	32 Open	Natural	Turf	2.56
1	32 Open	Natural	Turf	2.58
1	32 Open	Natural	Turf	3.21
1	32 Open	Natural	Turf	2.31
1	32 Open	Natural	Turf	2.69
1	32 Open	Natural	Turf	2.32
1	32 Open	Natural	Turf	3.81
1	33 Open	Natural	Turf	2.07
1	33 Open	Natural	Turf	1.33
1	33 Open	Natural	Turf	2.15
1	33 Open	Natural	Turf	1.55
1	33 Open	Natural	Turf	2.25
1	33 Open	Natural	Turf	2.06
1	33 Open	Natural	Turf	2.51
1	33 Open	Natural	Turf	4.68
1	33 Open	Natural	Turf	2.14
1	33 Open	Natural	Turf	3.43
1	33 Open	Natural	Turf	1.80
1	33 Open	Natural	Turf	2.36

1	33	Open	Natural	Turf	2.30
1	33	Open	Natural	Turf	3.88
1	33	Open	Natural	Turf	3.11
1	33	Open	Natural	Turf	2.80
1	33	Open	Natural	Turf	1.63
1	33	Open	Natural	Turf	2.94
1	33	Open	Natural	Turf	2.24
1	33	Open	Natural	Turf	2.30
1	34	Open	Addition	Turf	4.95
1	34	Open	Addition	Turf	2.68
1	34	Open	Addition	Turf	7.88
1	34	Open	Addition	Turf	7.60
1	34	Open	Addition	Turf	5.80
1	34	Open	Addition	Turf	4.31
1	34	Open	Addition	Turf	7.05
1	34	Open	Addition	Turf	13.94
1	34	Open	Addition	Turf	7.71
1	34	Open	Addition	Turf	4.00
1	34	Open	Addition	Turf	4.99
1	34	Open	Addition	Turf	10.69
1	34	Open	Addition	Turf	8.24
1	34	Open	Addition	Turf	2.54
1	34	Open	Addition	Turf	4.31
1	34	Open	Addition	Turf	7.37
1	34	Open	Addition	Turf	6.93
1	34	Open	Addition	Turf	4.14
1	34	Open	Addition	Turf	6.13
1	34	Open	Addition	Turf	5.19
1	35	Open	Addition	Turf	7.27
1	35	Open	Addition	Turf	5.54
1	35	Open	Addition	Turf	3.38
1	35	Open	Addition	Turf	3.03
1	35	Open	Addition	Turf	6.18
1	35	Open	Addition	Turf	5.63
1	35	Open	Addition	Turf	6.42
1	35	Open	Addition	Turf	6.37
1	35	Open	Addition	Turf	3.52
1	35	Open	Addition	Turf	14.45
1	35	Open	Addition	Turf	2.49
1	35	Open	Addition	Turf	5.19
1	35	Open	Addition	Turf	2.64
1	35	Open	Addition	Turf	3.17
1	35	Open	Addition	Turf	5.64
1	35	Open	Addition	Turf	5.65
1	35	Open	Addition	Turf	4.13
1	35	Open	Addition	Turf	3.53
1	35	Open	Addition	Turf	3.22
1	35	Open	Addition	Turf	6.62
1	36	Open	Addition	Turf	4.04
1	36	Open	Addition	Turf	4.63
1	36	Open	Addition	Turf	3.84
1	36	Open	Addition	Turf	2.50
1	36	Open	Addition	Turf	2.54

1	36	Open	Addition	Turf	3.99
1	36	Open	Addition	Turf	1.50
1	36	Open	Addition	Turf	1.43
1	36	Open	Addition	Turf	1.48
1	36	Open	Addition	Turf	3.40
1	36	Open	Addition	Turf	2.41
1	36	Open	Addition	Turf	2.47
1	36	Open	Addition	Turf	1.38
1	36	Open	Addition	Turf	2.70
1	36	Open	Addition	Turf	3.24
1	36	Open	Addition	Turf	4.28
1	36	Open	Addition	Turf	3.84
1	36	Open	Addition	Turf	1.61
1	36	Open	Addition	Turf	3.40
1	36	Open	Addition	Turf	3.69
2	1	Full	Natural	Turf	3.38
2	1	Full	Natural	Turf	2.37
2	1	Full	Natural	Turf	2.95
2	1	Full	Natural	Turf	13.38
2	1	Full	Natural	Turf	3.88
2	1	Full	Natural	Turf	5.61
2	1	Full	Natural	Turf	2.27
2	1	Full	Natural	Turf	2.51
2	1	Full	Natural	Turf	6.70
2	1	Full	Natural	Turf	8.71
2	1	Full	Natural	Turf	3.08
2	1	Full	Natural	Turf	3.15
2	1	Full	Natural	Turf	2.72
2	1	Full	Natural	Turf	3.53
2	1	Full	Natural	Turf	6.17
2	1	Full	Natural	Turf	2.23
2	1	Full	Natural	Turf	2.46
2	1	Full	Natural	Turf	3.29
2	1	Full	Natural	Turf	1.72
2	1	Full	Natural	Turf	3.06
2	2	Full	Natural	Turf	3.66
2	2	Full	Natural	Turf	6.05
2	2	Full	Natural	Turf	3.58
2	2	Full	Natural	Turf	2.65
2	2	Full	Natural	Turf	3.05
2	2	Full	Natural	Turf	3.32
2	2	Full	Natural	Turf	4.58
2	2	Full	Natural	Turf	11.04
2	2	Full	Natural	Turf	3.69
2	2	Full	Natural	Turf	3.21
2	2	Full	Natural	Turf	3.15
2	2	Full	Natural	Turf	4.15
2	2	Full	Natural	Turf	3.11
2	2	Full	Natural	Turf	3.29
2	2	Full	Natural	Turf	3.91
2	2	Full	Natural	Turf	4.86
2	2	Full	Natural	Turf	4.17
2	2	Full	Natural	Turf	3.18

2	2	Full	Natural	Turf	2.84
2	2	Full	Natural	Turf	6.63
2	3	Full	Natural	Turf	2.87
2	3	Full	Natural	Turf	2.99
2	3	Full	Natural	Turf	2.37
2	3	Full	Natural	Turf	2.66
2	3	Full	Natural	Turf	10.73
2	3	Full	Natural	Turf	5.17
2	3	Full	Natural	Turf	3.07
2	3	Full	Natural	Turf	3.03
2	3	Full	Natural	Turf	2.38
2	3	Full	Natural	Turf	2.22
2	3	Full	Natural	Turf	2.45
2	3	Full	Natural	Turf	3.83
2	3	Full	Natural	Turf	3.71
2	3	Full	Natural	Turf	4.08
2	3	Full	Natural	Turf	5.75
2	3	Full	Natural	Turf	5.54
2	3	Full	Natural	Turf	4.40
2	3	Full	Natural	Turf	2.20
2	3	Full	Natural	Turf	3.60
2	3	Full	Natural	Turf	2.04
2	4	Full	Addition	Turf	1.93
2	4	Full	Addition	Turf	3.64
2	4	Full	Addition	Turf	3.14
2	4	Full	Addition	Turf	6.44
2	4	Full	Addition	Turf	4.16
2	4	Full	Addition	Turf	6.17
2	4	Full	Addition	Turf	3.29
2	4	Full	Addition	Turf	6.03
2	4	Full	Addition	Turf	6.21
2	4	Full	Addition	Turf	4.43
2	4	Full	Addition	Turf	2.96
2	4	Full	Addition	Turf	6.98
2	4	Full	Addition	Turf	11.77
2	4	Full	Addition	Turf	10.42
2	4	Full	Addition	Turf	8.84
2	4	Full	Addition	Turf	4.65
2	4	Full	Addition	Turf	19.85
2	4	Full	Addition	Turf	5.00
2	4	Full	Addition	Turf	2.20
2	4	Full	Addition	Turf	5.02
2	5	Full	Addition	Turf	6.01
2	5	Full	Addition	Turf	4.06
2	5	Full	Addition	Turf	3.65
2	5	Full	Addition	Turf	3.40
2	5	Full	Addition	Turf	4.19
2	5	Full	Addition	Turf	4.28
2	5	Full	Addition	Turf	8.43
2	5	Full	Addition	Turf	5.89
2	5	Full	Addition	Turf	5.87
2	5	Full	Addition	Turf	4.99
2	5	Full	Addition	Turf	3.18

2	5	Full	Addition	Turf	7.56
2	5	Full	Addition	Turf	4.33
2	5	Full	Addition	Turf	3.57
2	5	Full	Addition	Turf	1.56
2	5	Full	Addition	Turf	1.43
2	5	Full	Addition	Turf	2.75
2	5	Full	Addition	Turf	4.52
2	5	Full	Addition	Turf	1.83
2	5	Full	Addition	Turf	4.55
2	6	Full	Addition	Turf	6.07
2	6	Full	Addition	Turf	1.43
2	6	Full	Addition	Turf	2.87
2	6	Full	Addition	Turf	1.86
2	6	Full	Addition	Turf	1.80
2	6	Full	Addition	Turf	4.45
2	6	Full	Addition	Turf	4.94
2	6	Full	Addition	Turf	2.97
2	6	Full	Addition	Turf	2.72
2	6	Full	Addition	Turf	3.76
2	6	Full	Addition	Turf	9.09
2	6	Full	Addition	Turf	4.02
2	6	Full	Addition	Turf	5.24
2	6	Full	Addition	Turf	3.06
2	6	Full	Addition	Turf	6.26
2	6	Full	Addition	Turf	6.66
2	6	Full	Addition	Turf	4.08
2	6	Full	Addition	Turf	4.59
2	6	Full	Addition	Turf	2.92
2	6	Full	Addition	Turf	5.95
2	7	Half	Natural	Turf	3.34
2	7	Half	Natural	Turf	6.83
2	7	Half	Natural	Turf	5.20
2	7	Half	Natural	Turf	4.32
2	7	Half	Natural	Turf	5.80
2	7	Half	Natural	Turf	5.20
2	7	Half	Natural	Turf	3.42
2	7	Half	Natural	Turf	2.32
2	7	Half	Natural	Turf	4.35
2	7	Half	Natural	Turf	6.72
2	7	Half	Natural	Turf	3.54
2	7	Half	Natural	Turf	1.89
2	7	Half	Natural	Turf	3.69
2	7	Half	Natural	Turf	2.93
2	7	Half	Natural	Turf	4.99
2	7	Half	Natural	Turf	6.05
2	7	Half	Natural	Turf	8.61
2	7	Half	Natural	Turf	5.62
2	7	Half	Natural	Turf	3.42
2	7	Half	Natural	Turf	3.20
2	8	Half	Natural	Turf	7.31
2	8	Half	Natural	Turf	2.90
2	8	Half	Natural	Turf	3.66
2	8	Half	Natural	Turf	5.31

2	8	Half	Natural	Turf	3.62
2	8	Half	Natural	Turf	6.53
2	8	Half	Natural	Turf	3.23
2	8	Half	Natural	Turf	3.23
2	8	Half	Natural	Turf	1.83
2	8	Half	Natural	Turf	3.95
2	8	Half	Natural	Turf	13.34
2	8	Half	Natural	Turf	3.16
2	8	Half	Natural	Turf	2.36
2	8	Half	Natural	Turf	4.25
2	8	Half	Natural	Turf	4.26
2	8	Half	Natural	Turf	3.31
2	8	Half	Natural	Turf	2.62
2	8	Half	Natural	Turf	2.67
2	8	Half	Natural	Turf	2.23
2	8	Half	Natural	Turf	4.02
2	9	Half	Natural	Turf	3.14
2	9	Half	Natural	Turf	7.87
2	9	Half	Natural	Turf	6.28
2	9	Half	Natural	Turf	7.46
2	9	Half	Natural	Turf	5.40
2	9	Half	Natural	Turf	9.50
2	9	Half	Natural	Turf	4.56
2	9	Half	Natural	Turf	2.97
2	9	Half	Natural	Turf	5.03
2	9	Half	Natural	Turf	8.01
2	9	Half	Natural	Turf	3.20
2	9	Half	Natural	Turf	2.47
2	9	Half	Natural	Turf	3.10
2	9	Half	Natural	Turf	3.01
2	9	Half	Natural	Turf	4.42
2	9	Half	Natural	Turf	3.54
2	9	Half	Natural	Turf	4.09
2	9	Half	Natural	Turf	4.46
2	9	Half	Natural	Turf	5.05
2	9	Half	Natural	Turf	2.76
2	10	Half	Addition	Turf	4.58
2	10	Half	Addition	Turf	2.21
2	10	Half	Addition	Turf	1.48
2	10	Half	Addition	Turf	1.54
2	10	Half	Addition	Turf	3.02
2	10	Half	Addition	Turf	3.09
2	10	Half	Addition	Turf	1.91
2	10	Half	Addition	Turf	2.87
2	10	Half	Addition	Turf	1.46
2	10	Half	Addition	Turf	1.42
2	10	Half	Addition	Turf	0.00
2	10	Half	Addition	Turf	4.92
2	10	Half	Addition	Turf	3.36
2	10	Half	Addition	Turf	2.36
2	10	Half	Addition	Turf	2.37
2	10	Half	Addition	Turf	1.67
2	10	Half	Addition	Turf	3.76

2	10	Half	Addition	Turf	3.22
2	10	Half	Addition	Turf	2.96
2	10	Half	Addition	Turf	2.33
2	11	Half	Addition	Turf	2.68
2	11	Half	Addition	Turf	4.52
2	11	Half	Addition	Turf	0.00
2	11	Half	Addition	Turf	2.19
2	11	Half	Addition	Turf	3.97
2	11	Half	Addition	Turf	5.96
2	11	Half	Addition	Turf	3.17
2	11	Half	Addition	Turf	4.37
2	11	Half	Addition	Turf	4.72
2	11	Half	Addition	Turf	6.85
2	11	Half	Addition	Turf	13.75
2	11	Half	Addition	Turf	4.33
2	11	Half	Addition	Turf	6.42
2	11	Half	Addition	Turf	5.68
2	11	Half	Addition	Turf	1.98
2	11	Half	Addition	Turf	1.90
2	11	Half	Addition	Turf	3.52
2	11	Half	Addition	Turf	2.49
2	11	Half	Addition	Turf	3.04
2	11	Half	Addition	Turf	3.91
2	12	Half	Addition	Turf	2.80
2	12	Half	Addition	Turf	3.08
2	12	Half	Addition	Turf	6.24
2	12	Half	Addition	Turf	6.55
2	12	Half	Addition	Turf	3.48
2	12	Half	Addition	Turf	3.81
2	12	Half	Addition	Turf	3.40
2	12	Half	Addition	Turf	7.94
2	12	Half	Addition	Turf	6.33
2	12	Half	Addition	Turf	2.70
2	12	Half	Addition	Turf	4.57
2	12	Half	Addition	Turf	4.11
2	12	Half	Addition	Turf	4.64
2	12	Half	Addition	Turf	3.70
2	12	Half	Addition	Turf	5.69
2	12	Half	Addition	Turf	5.81
2	12	Half	Addition	Turf	2.39
2	12	Half	Addition	Turf	3.33
2	12	Half	Addition	Turf	2.73
2	12	Half	Addition	Turf	5.43
2	13	Open	Natural	Turf	2.39
2	13	Open	Natural	Turf	3.48
2	13	Open	Natural	Turf	4.15
2	13	Open	Natural	Turf	3.08
2	13	Open	Natural	Turf	3.09
2	13	Open	Natural	Turf	4.10
2	13	Open	Natural	Turf	4.08
2	13	Open	Natural	Turf	2.58
2	13	Open	Natural	Turf	4.06
2	13	Open	Natural	Turf	2.07

2	13	Open	Natural	Turf	6.58
2	13	Open	Natural	Turf	2.15
2	13	Open	Natural	Turf	3.07
2	13	Open	Natural	Turf	2.49
2	13	Open	Natural	Turf	2.98
2	13	Open	Natural	Turf	2.12
2	13	Open	Natural	Turf	2.39
2	13	Open	Natural	Turf	5.69
2	13	Open	Natural	Turf	4.13
2	13	Open	Natural	Turf	4.83
2	14	Open	Natural	Turf	3.93
2	14	Open	Natural	Turf	2.15
2	14	Open	Natural	Turf	4.89
2	14	Open	Natural	Turf	2.99
2	14	Open	Natural	Turf	2.54
2	14	Open	Natural	Turf	3.42
2	14	Open	Natural	Turf	2.60
2	14	Open	Natural	Turf	3.66
2	14	Open	Natural	Turf	3.81
2	14	Open	Natural	Turf	2.37
2	14	Open	Natural	Turf	3.29
2	14	Open	Natural	Turf	3.85
2	14	Open	Natural	Turf	2.97
2	14	Open	Natural	Turf	3.42
2	14	Open	Natural	Turf	3.44
2	14	Open	Natural	Turf	2.49
2	14	Open	Natural	Turf	3.01
2	14	Open	Natural	Turf	3.00
2	14	Open	Natural	Turf	2.75
2	14	Open	Natural	Turf	2.85
2	15	Open	Natural	Turf	2.13
2	15	Open	Natural	Turf	3.33
2	15	Open	Natural	Turf	2.70
2	15	Open	Natural	Turf	2.05
2	15	Open	Natural	Turf	3.33
2	15	Open	Natural	Turf	2.13
2	15	Open	Natural	Turf	4.49
2	15	Open	Natural	Turf	3.82
2	15	Open	Natural	Turf	3.61
2	15	Open	Natural	Turf	1.68
2	15	Open	Natural	Turf	2.48
2	15	Open	Natural	Turf	3.42
2	15	Open	Natural	Turf	2.63
2	15	Open	Natural	Turf	2.13
2	15	Open	Natural	Turf	2.75
2	15	Open	Natural	Turf	3.47
2	15	Open	Natural	Turf	2.20
2	15	Open	Natural	Turf	2.51
2	15	Open	Natural	Turf	2.05
2	15	Open	Natural	Turf	4.09
2	16	Open	Addition	Turf	3.07
2	16	Open	Addition	Turf	3.24
2	16	Open	Addition	Turf	5.72

2	16	Open	Addition	Turf	6.12
2	16	Open	Addition	Turf	7.21
2	16	Open	Addition	Turf	2.62
2	16	Open	Addition	Turf	3.41
2	16	Open	Addition	Turf	10.81
2	16	Open	Addition	Turf	2.42
2	16	Open	Addition	Turf	4.93
2	16	Open	Addition	Turf	4.39
2	16	Open	Addition	Turf	7.40
2	16	Open	Addition	Turf	5.43
2	16	Open	Addition	Turf	3.64
2	16	Open	Addition	Turf	2.82
2	16	Open	Addition	Turf	3.54
2	16	Open	Addition	Turf	3.12
2	16	Open	Addition	Turf	2.81
2	16	Open	Addition	Turf	2.54
2	16	Open	Addition	Turf	5.84
2	17	Open	Addition	Turf	3.64
2	17	Open	Addition	Turf	7.02
2	17	Open	Addition	Turf	3.50
2	17	Open	Addition	Turf	9.33
2	17	Open	Addition	Turf	5.58
2	17	Open	Addition	Turf	4.33
2	17	Open	Addition	Turf	2.42
2	17	Open	Addition	Turf	7.34
2	17	Open	Addition	Turf	4.30
2	17	Open	Addition	Turf	7.94
2	17	Open	Addition	Turf	3.44
2	17	Open	Addition	Turf	3.60
2	17	Open	Addition	Turf	2.89
2	17	Open	Addition	Turf	2.38
2	17	Open	Addition	Turf	4.64
2	17	Open	Addition	Turf	4.98
2	17	Open	Addition	Turf	1.82
2	17	Open	Addition	Turf	3.26
2	17	Open	Addition	Turf	6.68
2	17	Open	Addition	Turf	6.25
2	18	Open	Addition	Turf	4.04
2	18	Open	Addition	Turf	3.60
2	18	Open	Addition	Turf	5.04
2	18	Open	Addition	Turf	3.46
2	18	Open	Addition	Turf	4.92
2	18	Open	Addition	Turf	6.61
2	18	Open	Addition	Turf	2.76
2	18	Open	Addition	Turf	4.17
2	18	Open	Addition	Turf	3.96
2	18	Open	Addition	Turf	14.33
2	18	Open	Addition	Turf	6.45
2	18	Open	Addition	Turf	5.17
2	18	Open	Addition	Turf	2.68
2	18	Open	Addition	Turf	3.94
2	18	Open	Addition	Turf	4.57
2	18	Open	Addition	Turf	2.94

2	18	Open	Addition	Turf	3.64
2	18	Open	Addition	Turf	4.77
2	18	Open	Addition	Turf	5.66
2	18	Open	Addition	Turf	2.60
2	19	Cage	Natural	Turf	2.45
2	19	Cage	Natural	Turf	2.50
2	19	Cage	Natural	Turf	5.73
2	19	Cage	Natural	Turf	4.33
2	19	Cage	Natural	Turf	4.62
2	19	Cage	Natural	Turf	4.11
2	19	Cage	Natural	Turf	7.00
2	19	Cage	Natural	Turf	7.51
2	19	Cage	Natural	Turf	2.30
2	19	Cage	Natural	Turf	2.41
2	19	Cage	Natural	Turf	6.08
2	19	Cage	Natural	Turf	5.67
2	19	Cage	Natural	Turf	4.52
2	19	Cage	Natural	Turf	4.36
2	19	Cage	Natural	Turf	2.43
2	19	Cage	Natural	Turf	2.82
2	19	Cage	Natural	Turf	5.48
2	19	Cage	Natural	Turf	4.32
2	19	Cage	Natural	Turf	4.48
2	19	Cage	Natural	Turf	5.33
2	20	Cage	Natural	Turf	8.35
2	20	Cage	Natural	Turf	2.24
2	20	Cage	Natural	Turf	3.51
2	20	Cage	Natural	Turf	3.32
2	20	Cage	Natural	Turf	3.27
2	20	Cage	Natural	Turf	4.70
2	20	Cage	Natural	Turf	4.13
2	20	Cage	Natural	Turf	4.61
2	20	Cage	Natural	Turf	3.04
2	20	Cage	Natural	Turf	3.72
2	20	Cage	Natural	Turf	4.68
2	20	Cage	Natural	Turf	4.57
2	20	Cage	Natural	Turf	2.82
2	20	Cage	Natural	Turf	3.61
2	20	Cage	Natural	Turf	4.07
2	20	Cage	Natural	Turf	2.68
2	20	Cage	Natural	Turf	5.07
2	20	Cage	Natural	Turf	4.72
2	20	Cage	Natural	Turf	9.16
2	20	Cage	Natural	Turf	5.65
2	21	Cage	Natural	Turf	5.76
2	21	Cage	Natural	Turf	3.41
2	21	Cage	Natural	Turf	3.28
2	21	Cage	Natural	Turf	3.81
2	21	Cage	Natural	Turf	4.13
2	21	Cage	Natural	Turf	4.14
2	21	Cage	Natural	Turf	3.02
2	21	Cage	Natural	Turf	3.83
2	21	Cage	Natural	Turf	11.15

2	21	Cage	Natural	Turf	6.08
2	21	Cage	Natural	Turf	6.32
2	21	Cage	Natural	Turf	5.02
2	21	Cage	Natural	Turf	2.44
2	21	Cage	Natural	Turf	2.70
2	21	Cage	Natural	Turf	5.39
2	21	Cage	Natural	Turf	4.19
2	21	Cage	Natural	Turf	4.89
2	21	Cage	Natural	Turf	3.43
2	21	Cage	Natural	Turf	5.14
2	21	Cage	Natural	Turf	4.11
2	22	Cage	Addition	Turf	5.25
2	22	Cage	Addition	Turf	0.00
2	22	Cage	Addition	Turf	4.02
2	22	Cage	Addition	Turf	3.27
2	22	Cage	Addition	Turf	2.31
2	22	Cage	Addition	Turf	2.97
2	22	Cage	Addition	Turf	10.66
2	22	Cage	Addition	Turf	2.49
2	22	Cage	Addition	Turf	5.81
2	22	Cage	Addition	Turf	1.82
2	22	Cage	Addition	Turf	21.59
2	22	Cage	Addition	Turf	2.92
2	22	Cage	Addition	Turf	2.48
2	22	Cage	Addition	Turf	7.39
2	22	Cage	Addition	Turf	3.21
2	22	Cage	Addition	Turf	1.87
2	22	Cage	Addition	Turf	5.95
2	22	Cage	Addition	Turf	4.95
2	22	Cage	Addition	Turf	6.97
2	22	Cage	Addition	Turf	4.26
2	23	Cage	Addition	Turf	4.65
2	23	Cage	Addition	Turf	5.72
2	23	Cage	Addition	Turf	11.13
2	23	Cage	Addition	Turf	6.33
2	23	Cage	Addition	Turf	8.34
2	23	Cage	Addition	Turf	4.83
2	23	Cage	Addition	Turf	8.05
2	23	Cage	Addition	Turf	3.54
2	23	Cage	Addition	Turf	7.78
2	23	Cage	Addition	Turf	14.82
2	23	Cage	Addition	Turf	6.21
2	23	Cage	Addition	Turf	3.42
2	23	Cage	Addition	Turf	4.91
2	23	Cage	Addition	Turf	0.00
2	23	Cage	Addition	Turf	2.58
2	23	Cage	Addition	Turf	5.13
2	23	Cage	Addition	Turf	6.51
2	23	Cage	Addition	Turf	6.47
2	23	Cage	Addition	Turf	3.03
2	23	Cage	Addition	Turf	7.98
2	24	Cage	Addition	Turf	6.52
2	24	Cage	Addition	Turf	4.66

2	24	Cage	Addition	Turf	2.30
2	24	Cage	Addition	Turf	4.28
2	24	Cage	Addition	Turf	4.15
2	24	Cage	Addition	Turf	5.60
2	24	Cage	Addition	Turf	4.10
2	24	Cage	Addition	Turf	2.46
2	24	Cage	Addition	Turf	5.66
2	24	Cage	Addition	Turf	0.00
2	24	Cage	Addition	Turf	3.93
2	24	Cage	Addition	Turf	10.07
2	24	Cage	Addition	Turf	4.61
2	24	Cage	Addition	Turf	3.18
2	24	Cage	Addition	Turf	10.08
2	24	Cage	Addition	Turf	10.26
2	24	Cage	Addition	Turf	2.83
2	24	Cage	Addition	Turf	5.58
2	24	Cage	Addition	Turf	4.72
2	24	Cage	Addition	Turf	6.27
2	25	Half	Natural	Turf	2.22
2	25	Half	Natural	Turf	9.24
2	25	Half	Natural	Turf	0.96
2	25	Half	Natural	Turf	3.02
2	25	Half	Natural	Turf	2.69
2	25	Half	Natural	Turf	3.62
2	25	Half	Natural	Turf	3.42
2	25	Half	Natural	Turf	4.31
2	25	Half	Natural	Turf	2.85
2	25	Half	Natural	Turf	6.75
2	25	Half	Natural	Turf	8.70
2	25	Half	Natural	Turf	4.49
2	25	Half	Natural	Turf	3.61
2	25	Half	Natural	Turf	3.65
2	25	Half	Natural	Turf	2.12
2	25	Half	Natural	Turf	4.06
2	25	Half	Natural	Turf	4.65
2	25	Half	Natural	Turf	3.47
2	25	Half	Natural	Turf	4.38
2	25	Half	Natural	Turf	5.01
2	26	Half	Natural	Turf	2.58
2	26	Half	Natural	Turf	2.49
2	26	Half	Natural	Turf	2.30
2	26	Half	Natural	Turf	4.47
2	26	Half	Natural	Turf	1.46
2	26	Half	Natural	Turf	3.80
2	26	Half	Natural	Turf	4.77
2	26	Half	Natural	Turf	4.53
2	26	Half	Natural	Turf	3.73
2	26	Half	Natural	Turf	2.83
2	26	Half	Natural	Turf	4.28
2	26	Half	Natural	Turf	3.47
2	26	Half	Natural	Turf	2.35
2	26	Half	Natural	Turf	2.42
2	26	Half	Natural	Turf	3.56

2	26	Half	Natural	Turf	3.73
2	26	Half	Natural	Turf	3.80
2	26	Half	Natural	Turf	2.76
2	26	Half	Natural	Turf	2.57
2	26	Half	Natural	Turf	1.90
2	27	Half	Natural	Turf	2.11
2	27	Half	Natural	Turf	3.44
2	27	Half	Natural	Turf	4.31
2	27	Half	Natural	Turf	3.06
2	27	Half	Natural	Turf	2.22
2	27	Half	Natural	Turf	3.06
2	27	Half	Natural	Turf	2.18
2	27	Half	Natural	Turf	1.44
2	27	Half	Natural	Turf	0.00
2	27	Half	Natural	Turf	1.83
2	27	Half	Natural	Turf	3.34
2	27	Half	Natural	Turf	1.78
2	27	Half	Natural	Turf	2.15
2	27	Half	Natural	Turf	1.88
2	27	Half	Natural	Turf	3.11
2	27	Half	Natural	Turf	5.00
2	27	Half	Natural	Turf	3.35
2	27	Half	Natural	Turf	1.66
2	27	Half	Natural	Turf	4.07
2	27	Half	Natural	Turf	2.94
2	28	Half	Addition	Turf	3.79
2	28	Half	Addition	Turf	3.03
2	28	Half	Addition	Turf	3.84
2	28	Half	Addition	Turf	4.11
2	28	Half	Addition	Turf	1.97
2	28	Half	Addition	Turf	3.59
2	28	Half	Addition	Turf	4.40
2	28	Half	Addition	Turf	6.76
2	28	Half	Addition	Turf	10.84
2	28	Half	Addition	Turf	8.06
2	28	Half	Addition	Turf	6.29
2	28	Half	Addition	Turf	4.26
2	28	Half	Addition	Turf	9.20
2	28	Half	Addition	Turf	6.07
2	28	Half	Addition	Turf	3.88
2	28	Half	Addition	Turf	2.62
2	28	Half	Addition	Turf	3.37
2	28	Half	Addition	Turf	3.63
2	28	Half	Addition	Turf	2.12
2	28	Half	Addition	Turf	2.86
2	29	Half	Addition	Turf	6.87
2	29	Half	Addition	Turf	5.12
2	29	Half	Addition	Turf	3.26
2	29	Half	Addition	Turf	3.96
2	29	Half	Addition	Turf	2.03
2	29	Half	Addition	Turf	4.90
2	29	Half	Addition	Turf	5.72
2	29	Half	Addition	Turf	0.00

2	29	Half	Addition	Turf	9.76
2	29	Half	Addition	Turf	3.20
2	29	Half	Addition	Turf	2.36
2	29	Half	Addition	Turf	5.40
2	29	Half	Addition	Turf	1.88
2	29	Half	Addition	Turf	2.93
2	29	Half	Addition	Turf	8.37
2	29	Half	Addition	Turf	3.98
2	29	Half	Addition	Turf	3.34
2	29	Half	Addition	Turf	4.08
2	29	Half	Addition	Turf	6.96
2	29	Half	Addition	Turf	4.99
2	30	Half	Addition	Turf	3.89
2	30	Half	Addition	Turf	4.31
2	30	Half	Addition	Turf	4.43
2	30	Half	Addition	Turf	0.00
2	30	Half	Addition	Turf	0.00
2	30	Half	Addition	Turf	4.85
2	30	Half	Addition	Turf	9.57
2	30	Half	Addition	Turf	7.11
2	30	Half	Addition	Turf	5.56
2	30	Half	Addition	Turf	15.12
2	30	Half	Addition	Turf	19.13
2	30	Half	Addition	Turf	15.30
2	30	Half	Addition	Turf	11.37
2	30	Half	Addition	Turf	6.94
2	30	Half	Addition	Turf	8.73
2	30	Half	Addition	Turf	2.11
2	30	Half	Addition	Turf	2.03
2	30	Half	Addition	Turf	4.20
2	30	Half	Addition	Turf	2.04
2	30	Half	Addition	Turf	8.44
2	31	Open	Natural	Turf	3.95
2	31	Open	Natural	Turf	1.91
2	31	Open	Natural	Turf	5.17
2	31	Open	Natural	Turf	4.51
2	31	Open	Natural	Turf	6.95
2	31	Open	Natural	Turf	3.45
2	31	Open	Natural	Turf	3.08
2	31	Open	Natural	Turf	3.50
2	31	Open	Natural	Turf	4.53
2	31	Open	Natural	Turf	3.36
2	31	Open	Natural	Turf	4.15
2	31	Open	Natural	Turf	3.46
2	31	Open	Natural	Turf	3.05
2	31	Open	Natural	Turf	4.33
2	31	Open	Natural	Turf	11.37
2	31	Open	Natural	Turf	2.90
2	31	Open	Natural	Turf	4.30
2	31	Open	Natural	Turf	3.15
2	31	Open	Natural	Turf	1.94
2	31	Open	Natural	Turf	2.74
2	32	Open	Natural	Turf	1.80

2	32	Open	Natural	Turf	3.16
2	32	Open	Natural	Turf	3.03
2	32	Open	Natural	Turf	2.44
2	32	Open	Natural	Turf	3.39
2	32	Open	Natural	Turf	3.76
2	32	Open	Natural	Turf	4.28
2	32	Open	Natural	Turf	3.92
2	32	Open	Natural	Turf	3.09
2	32	Open	Natural	Turf	3.25
2	32	Open	Natural	Turf	3.04
2	32	Open	Natural	Turf	3.56
2	32	Open	Natural	Turf	4.78
2	32	Open	Natural	Turf	4.55
2	32	Open	Natural	Turf	2.60
2	32	Open	Natural	Turf	3.08
2	32	Open	Natural	Turf	2.23
2	32	Open	Natural	Turf	2.08
2	32	Open	Natural	Turf	3.56
2	32	Open	Natural	Turf	3.69
2	33	Open	Natural	Turf	3.69
2	33	Open	Natural	Turf	4.77
2	33	Open	Natural	Turf	2.88
2	33	Open	Natural	Turf	1.76
2	33	Open	Natural	Turf	2.68
2	33	Open	Natural	Turf	2.94
2	33	Open	Natural	Turf	2.41
2	33	Open	Natural	Turf	3.40
2	33	Open	Natural	Turf	3.53
2	33	Open	Natural	Turf	1.91
2	33	Open	Natural	Turf	2.49
2	33	Open	Natural	Turf	2.08
2	33	Open	Natural	Turf	3.18
2	33	Open	Natural	Turf	2.10
2	33	Open	Natural	Turf	3.63
2	33	Open	Natural	Turf	3.68
2	33	Open	Natural	Turf	2.10
2	33	Open	Natural	Turf	2.30
2	33	Open	Natural	Turf	3.27
2	33	Open	Natural	Turf	3.56
2	34	Open	Addition	Turf	4.24
2	34	Open	Addition	Turf	8.55
2	34	Open	Addition	Turf	9.57
2	34	Open	Addition	Turf	2.01
2	34	Open	Addition	Turf	6.28
2	34	Open	Addition	Turf	5.56
2	34	Open	Addition	Turf	3.70
2	34	Open	Addition	Turf	18.29
2	34	Open	Addition	Turf	5.73
2	34	Open	Addition	Turf	12.15
2	34	Open	Addition	Turf	6.91
2	34	Open	Addition	Turf	5.23
2	34	Open	Addition	Turf	5.05
2	34	Open	Addition	Turf	4.79

2	34	Open	Addition	Turf	7.44
2	34	Open	Addition	Turf	3.03
2	34	Open	Addition	Turf	4.89
2	34	Open	Addition	Turf	7.44
2	34	Open	Addition	Turf	3.68
2	34	Open	Addition	Turf	5.97
2	35	Open	Addition	Turf	7.20
2	35	Open	Addition	Turf	2.95
2	35	Open	Addition	Turf	4.24
2	35	Open	Addition	Turf	3.66
2	35	Open	Addition	Turf	5.94
2	35	Open	Addition	Turf	3.20
2	35	Open	Addition	Turf	1.92
2	35	Open	Addition	Turf	4.00
2	35	Open	Addition	Turf	5.00
2	35	Open	Addition	Turf	3.64
2	35	Open	Addition	Turf	6.43
2	35	Open	Addition	Turf	5.98
2	35	Open	Addition	Turf	7.18
2	35	Open	Addition	Turf	5.10
2	35	Open	Addition	Turf	6.77
2	35	Open	Addition	Turf	2.62
2	35	Open	Addition	Turf	3.18
2	35	Open	Addition	Turf	2.89
2	35	Open	Addition	Turf	3.41
2	35	Open	Addition	Turf	5.91
2	36	Open	Addition	Turf	2.67
2	36	Open	Addition	Turf	4.34
2	36	Open	Addition	Turf	2.46
2	36	Open	Addition	Turf	2.40
2	36	Open	Addition	Turf	3.44
2	36	Open	Addition	Turf	2.95
2	36	Open	Addition	Turf	0.00
2	36	Open	Addition	Turf	3.81
2	36	Open	Addition	Turf	2.34
2	36	Open	Addition	Turf	1.27
2	36	Open	Addition	Turf	2.82
2	36	Open	Addition	Turf	2.72
2	36	Open	Addition	Turf	2.43
2	36	Open	Addition	Turf	5.54
2	36	Open	Addition	Turf	4.22
2	36	Open	Addition	Turf	3.84
2	36	Open	Addition	Turf	2.25
2	36	Open	Addition	Turf	3.22
2	36	Open	Addition	Turf	3.37
2	36	Open	Addition	Turf	3.54
3	1	Full	Natural	Turf	3.72
3	1	Full	Natural	Turf	5.23
3	1	Full	Natural	Turf	2.58
3	1	Full	Natural	Turf	8.12
3	1	Full	Natural	Turf	4.18
3	1	Full	Natural	Turf	3.15
3	1	Full	Natural	Turf	4.56

3	1	Full	Natural	Turf	4.62
3	1	Full	Natural	Turf	4.76
3	1	Full	Natural	Turf	4.78
3	1	Full	Natural	Turf	4.11
3	1	Full	Natural	Turf	6.08
3	1	Full	Natural	Turf	11.57
3	1	Full	Natural	Turf	3.34
3	1	Full	Natural	Turf	2.92
3	1	Full	Natural	Turf	3.10
3	1	Full	Natural	Turf	4.49
3	1	Full	Natural	Turf	3.37
3	1	Full	Natural	Turf	3.53
3	1	Full	Natural	Turf	2.93
3	2	Full	Natural	Turf	5.96
3	2	Full	Natural	Turf	3.07
3	2	Full	Natural	Turf	4.08
3	2	Full	Natural	Turf	3.34
3	2	Full	Natural	Turf	5.93
3	2	Full	Natural	Turf	3.98
3	2	Full	Natural	Turf	2.79
3	2	Full	Natural	Turf	3.94
3	2	Full	Natural	Turf	4.76
3	2	Full	Natural	Turf	8.03
3	2	Full	Natural	Turf	4.05
3	2	Full	Natural	Turf	6.70
3	2	Full	Natural	Turf	11.06
3	2	Full	Natural	Turf	8.20
3	2	Full	Natural	Turf	3.97
3	2	Full	Natural	Turf	3.26
3	2	Full	Natural	Turf	3.94
3	2	Full	Natural	Turf	6.70
3	2	Full	Natural	Turf	5.86
3	2	Full	Natural	Turf	3.16
3	3	Full	Natural	Turf	3.88
3	3	Full	Natural	Turf	3.04
3	3	Full	Natural	Turf	4.21
3	3	Full	Natural	Turf	2.70
3	3	Full	Natural	Turf	9.97
3	3	Full	Natural	Turf	2.19
3	3	Full	Natural	Turf	4.44
3	3	Full	Natural	Turf	2.65
3	3	Full	Natural	Turf	3.42
3	3	Full	Natural	Turf	2.81
3	3	Full	Natural	Turf	3.79
3	3	Full	Natural	Turf	4.25
3	3	Full	Natural	Turf	5.56
3	3	Full	Natural	Turf	4.46
3	3	Full	Natural	Turf	3.38
3	3	Full	Natural	Turf	3.95
3	3	Full	Natural	Turf	2.50
3	3	Full	Natural	Turf	2.15
3	3	Full	Natural	Turf	2.48
3	3	Full	Natural	Turf	3.97

3	4	Full	Addition	Turf	13.81
3	4	Full	Addition	Turf	3.69
3	4	Full	Addition	Turf	8.11
3	4	Full	Addition	Turf	4.57
3	4	Full	Addition	Turf	4.17
3	4	Full	Addition	Turf	5.11
3	4	Full	Addition	Turf	12.17
3	4	Full	Addition	Turf	8.73
3	4	Full	Addition	Turf	4.30
3	4	Full	Addition	Turf	8.20
3	4	Full	Addition	Turf	7.47
3	4	Full	Addition	Turf	5.02
3	4	Full	Addition	Turf	4.62
3	4	Full	Addition	Turf	4.68
3	4	Full	Addition	Turf	6.43
3	4	Full	Addition	Turf	5.81
3	4	Full	Addition	Turf	8.08
3	4	Full	Addition	Turf	5.55
3	4	Full	Addition	Turf	4.29
3	4	Full	Addition	Turf	5.12
3	5	Full	Addition	Turf	7.50
3	5	Full	Addition	Turf	4.52
3	5	Full	Addition	Turf	4.96
3	5	Full	Addition	Turf	4.91
3	5	Full	Addition	Turf	4.71
3	5	Full	Addition	Turf	6.50
3	5	Full	Addition	Turf	3.01
3	5	Full	Addition	Turf	6.16
3	5	Full	Addition	Turf	4.62
3	5	Full	Addition	Turf	3.78
3	5	Full	Addition	Turf	3.44
3	5	Full	Addition	Turf	5.94
3	5	Full	Addition	Turf	6.54
3	5	Full	Addition	Turf	3.37
3	5	Full	Addition	Turf	5.48
3	5	Full	Addition	Turf	3.58
3	5	Full	Addition	Turf	2.40
3	5	Full	Addition	Turf	4.95
3	5	Full	Addition	Turf	4.20
3	5	Full	Addition	Turf	3.04
3	6	Full	Addition	Turf	4.45
3	6	Full	Addition	Turf	3.73
3	6	Full	Addition	Turf	6.13
3	6	Full	Addition	Turf	6.15
3	6	Full	Addition	Turf	5.36
3	6	Full	Addition	Turf	2.54
3	6	Full	Addition	Turf	3.51
3	6	Full	Addition	Turf	6.13
3	6	Full	Addition	Turf	4.97
3	6	Full	Addition	Turf	3.68
3	6	Full	Addition	Turf	2.41
3	6	Full	Addition	Turf	4.66
3	6	Full	Addition	Turf	3.82

3	6	Full	Addition	Turf	3.70
3	6	Full	Addition	Turf	4.01
3	6	Full	Addition	Turf	5.31
3	6	Full	Addition	Turf	2.96
3	6	Full	Addition	Turf	2.94
3	6	Full	Addition	Turf	4.06
3	6	Full	Addition	Turf	5.67
3	7	Half	Natural	Turf	1.91
3	7	Half	Natural	Turf	1.67
3	7	Half	Natural	Turf	2.38
3	7	Half	Natural	Turf	2.81
3	7	Half	Natural	Turf	2.16
3	7	Half	Natural	Turf	2.67
3	7	Half	Natural	Turf	5.55
3	7	Half	Natural	Turf	6.75
3	7	Half	Natural	Turf	3.65
3	7	Half	Natural	Turf	1.65
3	7	Half	Natural	Turf	1.67
3	7	Half	Natural	Turf	7.69
3	7	Half	Natural	Turf	2.40
3	7	Half	Natural	Turf	2.56
3	7	Half	Natural	Turf	3.82
3	7	Half	Natural	Turf	3.76
3	7	Half	Natural	Turf	6.01
3	7	Half	Natural	Turf	2.65
3	7	Half	Natural	Turf	2.01
3	7	Half	Natural	Turf	4.18
3	8	Half	Natural	Turf	1.00
3	8	Half	Natural	Turf	2.72
3	8	Half	Natural	Turf	5.00
3	8	Half	Natural	Turf	4.14
3	8	Half	Natural	Turf	4.64
3	8	Half	Natural	Turf	3.60
3	8	Half	Natural	Turf	3.24
3	8	Half	Natural	Turf	2.42
3	8	Half	Natural	Turf	5.11
3	8	Half	Natural	Turf	5.34
3	8	Half	Natural	Turf	2.84
3	8	Half	Natural	Turf	4.50
3	8	Half	Natural	Turf	3.68
3	8	Half	Natural	Turf	3.39
3	8	Half	Natural	Turf	2.26
3	8	Half	Natural	Turf	3.86
3	8	Half	Natural	Turf	2.96
3	8	Half	Natural	Turf	2.20
3	8	Half	Natural	Turf	4.66
3	8	Half	Natural	Turf	2.34
3	9	Half	Natural	Turf	4.03
3	9	Half	Natural	Turf	5.10
3	9	Half	Natural	Turf	3.89
3	9	Half	Natural	Turf	3.08
3	9	Half	Natural	Turf	6.06
3	9	Half	Natural	Turf	2.41

3	9	Half	Natural	Turf	3.59
3	9	Half	Natural	Turf	2.78
3	9	Half	Natural	Turf	1.77
3	9	Half	Natural	Turf	2.45
3	9	Half	Natural	Turf	3.95
3	9	Half	Natural	Turf	2.64
3	9	Half	Natural	Turf	2.98
3	9	Half	Natural	Turf	2.96
3	9	Half	Natural	Turf	3.02
3	9	Half	Natural	Turf	4.68
3	9	Half	Natural	Turf	3.69
3	9	Half	Natural	Turf	1.73
3	9	Half	Natural	Turf	4.32
3	9	Half	Natural	Turf	3.13
3	10	Half	Addition	Turf	4.20
3	10	Half	Addition	Turf	0.65
3	10	Half	Addition	Turf	2.85
3	10	Half	Addition	Turf	2.51
3	10	Half	Addition	Turf	2.99
3	10	Half	Addition	Turf	1.65
3	10	Half	Addition	Turf	6.28
3	10	Half	Addition	Turf	4.48
3	10	Half	Addition	Turf	4.62
3	10	Half	Addition	Turf	3.14
3	10	Half	Addition	Turf	1.57
3	10	Half	Addition	Turf	3.46
3	10	Half	Addition	Turf	4.62
3	10	Half	Addition	Turf	11.26
3	10	Half	Addition	Turf	3.52
3	10	Half	Addition	Turf	2.55
3	10	Half	Addition	Turf	1.49
3	10	Half	Addition	Turf	6.14
3	10	Half	Addition	Turf	3.57
3	10	Half	Addition	Turf	4.98
3	11	Half	Addition	Turf	3.06
3	11	Half	Addition	Turf	4.78
3	11	Half	Addition	Turf	5.37
3	11	Half	Addition	Turf	3.27
3	11	Half	Addition	Turf	5.40
3	11	Half	Addition	Turf	11.43
3	11	Half	Addition	Turf	2.06
3	11	Half	Addition	Turf	3.14
3	11	Half	Addition	Turf	3.72
3	11	Half	Addition	Turf	3.78
3	11	Half	Addition	Turf	2.93
3	11	Half	Addition	Turf	2.26
3	11	Half	Addition	Turf	4.52
3	11	Half	Addition	Turf	2.79
3	11	Half	Addition	Turf	4.22
3	11	Half	Addition	Turf	4.33
3	11	Half	Addition	Turf	2.71
3	11	Half	Addition	Turf	2.23
3	11	Half	Addition	Turf	4.49

3	11	Half	Addition	Turf	2.96
3	12	Half	Addition	Turf	3.06
3	12	Half	Addition	Turf	3.33
3	12	Half	Addition	Turf	3.35
3	12	Half	Addition	Turf	4.33
3	12	Half	Addition	Turf	3.38
3	12	Half	Addition	Turf	3.67
3	12	Half	Addition	Turf	5.01
3	12	Half	Addition	Turf	12.76
3	12	Half	Addition	Turf	9.23
3	12	Half	Addition	Turf	6.78
3	12	Half	Addition	Turf	4.48
3	12	Half	Addition	Turf	6.05
3	12	Half	Addition	Turf	3.67
3	12	Half	Addition	Turf	2.32
3	12	Half	Addition	Turf	5.72
3	12	Half	Addition	Turf	2.50
3	12	Half	Addition	Turf	3.69
3	12	Half	Addition	Turf	3.61
3	12	Half	Addition	Turf	4.81
3	12	Half	Addition	Turf	3.53
3	13	Open	Natural	Turf	2.09
3	13	Open	Natural	Turf	2.27
3	13	Open	Natural	Turf	6.10
3	13	Open	Natural	Turf	3.18
3	13	Open	Natural	Turf	6.66
3	13	Open	Natural	Turf	2.38
3	13	Open	Natural	Turf	2.21
3	13	Open	Natural	Turf	2.02
3	13	Open	Natural	Turf	4.04
3	13	Open	Natural	Turf	5.53
3	13	Open	Natural	Turf	2.27
3	13	Open	Natural	Turf	2.28
3	13	Open	Natural	Turf	2.61
3	13	Open	Natural	Turf	1.86
3	13	Open	Natural	Turf	5.45
3	13	Open	Natural	Turf	2.39
3	13	Open	Natural	Turf	2.88
3	13	Open	Natural	Turf	9.31
3	13	Open	Natural	Turf	1.63
3	13	Open	Natural	Turf	3.03
3	14	Open	Natural	Turf	2.38
3	14	Open	Natural	Turf	6.61
3	14	Open	Natural	Turf	2.95
3	14	Open	Natural	Turf	2.50
3	14	Open	Natural	Turf	2.52
3	14	Open	Natural	Turf	4.91
3	14	Open	Natural	Turf	1.90
3	14	Open	Natural	Turf	3.15
3	14	Open	Natural	Turf	3.85
3	14	Open	Natural	Turf	2.56
3	14	Open	Natural	Turf	4.32
3	14	Open	Natural	Turf	1.76

3	14	Open	Natural	Turf	3.08
3	14	Open	Natural	Turf	5.59
3	14	Open	Natural	Turf	3.98
3	14	Open	Natural	Turf	6.58
3	14	Open	Natural	Turf	3.02
3	14	Open	Natural	Turf	1.97
3	14	Open	Natural	Turf	3.33
3	14	Open	Natural	Turf	2.84
3	15	Open	Natural	Turf	3.08
3	15	Open	Natural	Turf	2.35
3	15	Open	Natural	Turf	1.48
3	15	Open	Natural	Turf	2.42
3	15	Open	Natural	Turf	2.99
3	15	Open	Natural	Turf	1.53
3	15	Open	Natural	Turf	1.26
3	15	Open	Natural	Turf	1.77
3	15	Open	Natural	Turf	0.00
3	15	Open	Natural	Turf	2.66
3	15	Open	Natural	Turf	4.14
3	15	Open	Natural	Turf	4.81
3	15	Open	Natural	Turf	2.48
3	15	Open	Natural	Turf	2.56
3	15	Open	Natural	Turf	4.59
3	15	Open	Natural	Turf	2.11
3	15	Open	Natural	Turf	3.31
3	15	Open	Natural	Turf	2.60
3	15	Open	Natural	Turf	2.31
3	15	Open	Natural	Turf	4.15
3	16	Open	Addition	Turf	4.26
3	16	Open	Addition	Turf	3.59
3	16	Open	Addition	Turf	3.18
3	16	Open	Addition	Turf	4.18
3	16	Open	Addition	Turf	2.27
3	16	Open	Addition	Turf	3.67
3	16	Open	Addition	Turf	3.10
3	16	Open	Addition	Turf	3.64
3	16	Open	Addition	Turf	2.75
3	16	Open	Addition	Turf	3.76
3	16	Open	Addition	Turf	3.70
3	16	Open	Addition	Turf	1.50
3	16	Open	Addition	Turf	5.48
3	16	Open	Addition	Turf	3.47
3	16	Open	Addition	Turf	3.38
3	16	Open	Addition	Turf	2.03
3	16	Open	Addition	Turf	1.58
3	16	Open	Addition	Turf	3.92
3	16	Open	Addition	Turf	3.95
3	16	Open	Addition	Turf	4.85
3	17	Open	Addition	Turf	3.37
3	17	Open	Addition	Turf	4.89
3	17	Open	Addition	Turf	6.03
3	17	Open	Addition	Turf	4.37
3	17	Open	Addition	Turf	4.74

3	17	Open	Addition	Turf	3.93
3	17	Open	Addition	Turf	7.96
3	17	Open	Addition	Turf	6.28
3	17	Open	Addition	Turf	3.71
3	17	Open	Addition	Turf	3.83
3	17	Open	Addition	Turf	6.34
3	17	Open	Addition	Turf	6.75
3	17	Open	Addition	Turf	3.70
3	17	Open	Addition	Turf	3.53
3	17	Open	Addition	Turf	6.67
3	17	Open	Addition	Turf	4.94
3	17	Open	Addition	Turf	7.07
3	17	Open	Addition	Turf	2.85
3	17	Open	Addition	Turf	8.13
3	17	Open	Addition	Turf	4.63
3	18	Open	Addition	Turf	6.79
3	18	Open	Addition	Turf	3.17
3	18	Open	Addition	Turf	4.27
3	18	Open	Addition	Turf	3.55
3	18	Open	Addition	Turf	3.10
3	18	Open	Addition	Turf	1.78
3	18	Open	Addition	Turf	2.85
3	18	Open	Addition	Turf	3.37
3	18	Open	Addition	Turf	8.33
3	18	Open	Addition	Turf	7.01
3	18	Open	Addition	Turf	9.85
3	18	Open	Addition	Turf	5.33
3	18	Open	Addition	Turf	2.46
3	18	Open	Addition	Turf	5.04
3	18	Open	Addition	Turf	4.94
3	18	Open	Addition	Turf	2.69
3	18	Open	Addition	Turf	2.99
3	18	Open	Addition	Turf	3.04
3	18	Open	Addition	Turf	3.13
3	18	Open	Addition	Turf	4.21
3	19	Cage	Natural	Turf	6.04
3	19	Cage	Natural	Turf	5.22
3	19	Cage	Natural	Turf	3.51
3	19	Cage	Natural	Turf	10.27
3	19	Cage	Natural	Turf	3.13
3	19	Cage	Natural	Turf	4.54
3	19	Cage	Natural	Turf	1.48
3	19	Cage	Natural	Turf	4.83
3	19	Cage	Natural	Turf	2.81
3	19	Cage	Natural	Turf	7.81
3	19	Cage	Natural	Turf	4.62
3	19	Cage	Natural	Turf	6.62
3	19	Cage	Natural	Turf	11.10
3	19	Cage	Natural	Turf	7.99
3	19	Cage	Natural	Turf	5.21
3	19	Cage	Natural	Turf	1.25
3	19	Cage	Natural	Turf	3.16
3	19	Cage	Natural	Turf	1.38

3	19	Cage	Natural	Turf	7.26
3	19	Cage	Natural	Turf	7.24
3	20	Cage	Natural	Turf	4.81
3	20	Cage	Natural	Turf	4.37
3	20	Cage	Natural	Turf	6.09
3	20	Cage	Natural	Turf	1.64
3	20	Cage	Natural	Turf	2.73
3	20	Cage	Natural	Turf	3.98
3	20	Cage	Natural	Turf	3.43
3	20	Cage	Natural	Turf	3.13
3	20	Cage	Natural	Turf	2.42
3	20	Cage	Natural	Turf	3.48
3	20	Cage	Natural	Turf	8.00
3	20	Cage	Natural	Turf	2.60
3	20	Cage	Natural	Turf	3.65
3	20	Cage	Natural	Turf	1.48
3	20	Cage	Natural	Turf	4.90
3	20	Cage	Natural	Turf	5.42
3	20	Cage	Natural	Turf	5.42
3	20	Cage	Natural	Turf	2.27
3	20	Cage	Natural	Turf	5.40
3	20	Cage	Natural	Turf	4.62
3	21	Cage	Natural	Turf	1.72
3	21	Cage	Natural	Turf	2.76
3	21	Cage	Natural	Turf	7.06
3	21	Cage	Natural	Turf	3.16
3	21	Cage	Natural	Turf	7.10
3	21	Cage	Natural	Turf	5.53
3	21	Cage	Natural	Turf	1.45
3	21	Cage	Natural	Turf	7.59
3	21	Cage	Natural	Turf	1.55
3	21	Cage	Natural	Turf	3.77
3	21	Cage	Natural	Turf	5.38
3	21	Cage	Natural	Turf	4.06
3	21	Cage	Natural	Turf	4.23
3	21	Cage	Natural	Turf	5.56
3	21	Cage	Natural	Turf	5.61
3	21	Cage	Natural	Turf	3.58
3	21	Cage	Natural	Turf	2.92
3	21	Cage	Natural	Turf	6.39
3	21	Cage	Natural	Turf	4.79
3	21	Cage	Natural	Turf	4.52
3	22	Cage	Addition	Turf	4.82
3	22	Cage	Addition	Turf	0.00
3	22	Cage	Addition	Turf	4.47
3	22	Cage	Addition	Turf	4.14
3	22	Cage	Addition	Turf	1.00
3	22	Cage	Addition	Turf	1.24
3	22	Cage	Addition	Turf	6.69
3	22	Cage	Addition	Turf	1.84
3	22	Cage	Addition	Turf	5.63
3	22	Cage	Addition	Turf	8.16
3	22	Cage	Addition	Turf	6.22

3	22	Cage	Addition	Turf	5.51
3	22	Cage	Addition	Turf	0.00
3	22	Cage	Addition	Turf	1.43
3	22	Cage	Addition	Turf	6.17
3	22	Cage	Addition	Turf	2.55
3	22	Cage	Addition	Turf	0.00
3	22	Cage	Addition	Turf	3.45
3	22	Cage	Addition	Turf	3.13
3	22	Cage	Addition	Turf	7.46
3	23	Cage	Addition	Turf	4.31
3	23	Cage	Addition	Turf	0.00
3	23	Cage	Addition	Turf	3.82
3	23	Cage	Addition	Turf	0.00
3	23	Cage	Addition	Turf	1.32
3	23	Cage	Addition	Turf	4.14
3	23	Cage	Addition	Turf	14.17
3	23	Cage	Addition	Turf	0.00
3	23	Cage	Addition	Turf	3.77
3	23	Cage	Addition	Turf	13.73
3	23	Cage	Addition	Turf	3.26
3	23	Cage	Addition	Turf	5.52
3	23	Cage	Addition	Turf	3.17
3	23	Cage	Addition	Turf	3.40
3	23	Cage	Addition	Turf	7.54
3	23	Cage	Addition	Turf	2.47
3	23	Cage	Addition	Turf	3.85
3	23	Cage	Addition	Turf	4.54
3	23	Cage	Addition	Turf	6.65
3	23	Cage	Addition	Turf	1.27
3	24	Cage	Addition	Turf	3.50
3	24	Cage	Addition	Turf	2.27
3	24	Cage	Addition	Turf	8.33
3	24	Cage	Addition	Turf	9.60
3	24	Cage	Addition	Turf	2.25
3	24	Cage	Addition	Turf	8.37
3	24	Cage	Addition	Turf	2.18
3	24	Cage	Addition	Turf	0.00
3	24	Cage	Addition	Turf	3.71
3	24	Cage	Addition	Turf	11.45
3	24	Cage	Addition	Turf	4.56
3	24	Cage	Addition	Turf	5.76
3	24	Cage	Addition	Turf	11.89
3	24	Cage	Addition	Turf	5.23
3	24	Cage	Addition	Turf	3.20
3	24	Cage	Addition	Turf	1.76
3	24	Cage	Addition	Turf	5.17
3	24	Cage	Addition	Turf	6.47
3	24	Cage	Addition	Turf	1.98
3	24	Cage	Addition	Turf	3.84
3	25	Half	Natural	Turf	3.14
3	25	Half	Natural	Turf	4.32
3	25	Half	Natural	Turf	2.50
3	25	Half	Natural	Turf	1.93

3	25	Half	Natural	Turf	3.19
3	25	Half	Natural	Turf	1.81
3	25	Half	Natural	Turf	6.08
3	25	Half	Natural	Turf	3.95
3	25	Half	Natural	Turf	3.74
3	25	Half	Natural	Turf	1.69
3	25	Half	Natural	Turf	3.52
3	25	Half	Natural	Turf	4.84
3	25	Half	Natural	Turf	1.05
3	25	Half	Natural	Turf	2.36
3	25	Half	Natural	Turf	2.44
3	25	Half	Natural	Turf	2.31
3	25	Half	Natural	Turf	3.79
3	25	Half	Natural	Turf	3.81
3	25	Half	Natural	Turf	2.67
3	25	Half	Natural	Turf	2.73
3	26	Half	Natural	Turf	2.17
3	26	Half	Natural	Turf	2.29
3	26	Half	Natural	Turf	1.95
3	26	Half	Natural	Turf	1.43
3	26	Half	Natural	Turf	3.02
3	26	Half	Natural	Turf	5.46
3	26	Half	Natural	Turf	2.26
3	26	Half	Natural	Turf	2.52
3	26	Half	Natural	Turf	1.26
3	26	Half	Natural	Turf	2.78
3	26	Half	Natural	Turf	3.67
3	26	Half	Natural	Turf	4.22
3	26	Half	Natural	Turf	2.80
3	26	Half	Natural	Turf	4.88
3	26	Half	Natural	Turf	4.58
3	26	Half	Natural	Turf	3.12
3	26	Half	Natural	Turf	2.89
3	26	Half	Natural	Turf	3.18
3	26	Half	Natural	Turf	2.57
3	26	Half	Natural	Turf	1.99
3	27	Half	Natural	Turf	2.21
3	27	Half	Natural	Turf	5.05
3	27	Half	Natural	Turf	2.92
3	27	Half	Natural	Turf	1.91
3	27	Half	Natural	Turf	2.17
3	27	Half	Natural	Turf	3.45
3	27	Half	Natural	Turf	1.89
3	27	Half	Natural	Turf	2.34
3	27	Half	Natural	Turf	4.68
3	27	Half	Natural	Turf	3.21
3	27	Half	Natural	Turf	6.96
3	27	Half	Natural	Turf	3.74
3	27	Half	Natural	Turf	2.89
3	27	Half	Natural	Turf	1.73
3	27	Half	Natural	Turf	4.13
3	27	Half	Natural	Turf	1.82
3	27	Half	Natural	Turf	1.46

3	27	Half	Natural	Turf	2.48
3	27	Half	Natural	Turf	2.91
3	27	Half	Natural	Turf	1.91
3	28	Half	Addition	Turf	1.23
3	28	Half	Addition	Turf	2.38
3	28	Half	Addition	Turf	2.75
3	28	Half	Addition	Turf	0.00
3	28	Half	Addition	Turf	1.17
3	28	Half	Addition	Turf	3.64
3	28	Half	Addition	Turf	2.46
3	28	Half	Addition	Turf	4.58
3	28	Half	Addition	Turf	3.26
3	28	Half	Addition	Turf	6.97
3	28	Half	Addition	Turf	8.13
3	28	Half	Addition	Turf	2.45
3	28	Half	Addition	Turf	4.29
3	28	Half	Addition	Turf	1.31
3	28	Half	Addition	Turf	4.41
3	28	Half	Addition	Turf	3.83
3	28	Half	Addition	Turf	4.49
3	28	Half	Addition	Turf	2.53
3	28	Half	Addition	Turf	2.53
3	28	Half	Addition	Turf	2.84
3	29	Half	Addition	Turf	4.69
3	29	Half	Addition	Turf	5.67
3	29	Half	Addition	Turf	5.80
3	29	Half	Addition	Turf	1.37
3	29	Half	Addition	Turf	3.23
3	29	Half	Addition	Turf	7.91
3	29	Half	Addition	Turf	1.15
3	29	Half	Addition	Turf	1.60
3	29	Half	Addition	Turf	6.60
3	29	Half	Addition	Turf	6.01
3	29	Half	Addition	Turf	9.71
3	29	Half	Addition	Turf	0.00
3	29	Half	Addition	Turf	0.00
3	29	Half	Addition	Turf	3.76
3	29	Half	Addition	Turf	5.16
3	29	Half	Addition	Turf	0.00
3	29	Half	Addition	Turf	2.81
3	29	Half	Addition	Turf	2.01
3	29	Half	Addition	Turf	1.99
3	29	Half	Addition	Turf	2.80
3	30	Half	Addition	Turf	1.14
3	30	Half	Addition	Turf	1.72
3	30	Half	Addition	Turf	5.23
3	30	Half	Addition	Turf	3.33
3	30	Half	Addition	Turf	0.00
3	30	Half	Addition	Turf	2.27
3	30	Half	Addition	Turf	1.26
3	30	Half	Addition	Turf	6.15
3	30	Half	Addition	Turf	1.91
3	30	Half	Addition	Turf	11.54

3	30	Half	Addition	Turf	3.94
3	30	Half	Addition	Turf	1.47
3	30	Half	Addition	Turf	2.40
3	30	Half	Addition	Turf	2.19
3	30	Half	Addition	Turf	3.06
3	30	Half	Addition	Turf	1.71
3	30	Half	Addition	Turf	5.60
3	30	Half	Addition	Turf	5.34
3	30	Half	Addition	Turf	2.76
3	30	Half	Addition	Turf	7.28
3	31	Open	Natural	Turf	2.67
3	31	Open	Natural	Turf	5.92
3	31	Open	Natural	Turf	2.82
3	31	Open	Natural	Turf	3.95
3	31	Open	Natural	Turf	4.98
3	31	Open	Natural	Turf	6.20
3	31	Open	Natural	Turf	1.63
3	31	Open	Natural	Turf	4.18
3	31	Open	Natural	Turf	5.10
3	31	Open	Natural	Turf	5.48
3	31	Open	Natural	Turf	4.06
3	31	Open	Natural	Turf	5.08
3	31	Open	Natural	Turf	7.12
3	31	Open	Natural	Turf	4.95
3	31	Open	Natural	Turf	3.20
3	31	Open	Natural	Turf	2.43
3	31	Open	Natural	Turf	2.01
3	31	Open	Natural	Turf	1.38
3	31	Open	Natural	Turf	3.30
3	31	Open	Natural	Turf	2.19
3	32	Open	Natural	Turf	2.38
3	32	Open	Natural	Turf	2.28
3	32	Open	Natural	Turf	3.14
3	32	Open	Natural	Turf	4.27
3	32	Open	Natural	Turf	2.15
3	32	Open	Natural	Turf	3.12
3	32	Open	Natural	Turf	2.80
3	32	Open	Natural	Turf	2.48
3	32	Open	Natural	Turf	2.38
3	32	Open	Natural	Turf	2.72
3	32	Open	Natural	Turf	2.74
3	32	Open	Natural	Turf	1.60
3	32	Open	Natural	Turf	2.18
3	32	Open	Natural	Turf	2.06
3	32	Open	Natural	Turf	2.58
3	32	Open	Natural	Turf	2.37
3	32	Open	Natural	Turf	2.51
3	32	Open	Natural	Turf	3.55
3	32	Open	Natural	Turf	3.00
3	32	Open	Natural	Turf	1.74
3	33	Open	Natural	Turf	1.51
3	33	Open	Natural	Turf	0.89
3	33	Open	Natural	Turf	2.37

3	33	Open	Natural	Turf	2.42
3	33	Open	Natural	Turf	2.95
3	33	Open	Natural	Turf	2.71
3	33	Open	Natural	Turf	2.05
3	33	Open	Natural	Turf	2.05
3	33	Open	Natural	Turf	1.81
3	33	Open	Natural	Turf	1.52
3	33	Open	Natural	Turf	3.18
3	33	Open	Natural	Turf	3.59
3	33	Open	Natural	Turf	2.02
3	33	Open	Natural	Turf	0.92
3	33	Open	Natural	Turf	2.21
3	33	Open	Natural	Turf	2.29
3	33	Open	Natural	Turf	1.69
3	33	Open	Natural	Turf	2.71
3	33	Open	Natural	Turf	1.10
3	33	Open	Natural	Turf	2.22
3	34	Open	Addition	Turf	14.94
3	34	Open	Addition	Turf	7.63
3	34	Open	Addition	Turf	3.44
3	34	Open	Addition	Turf	2.15
3	34	Open	Addition	Turf	3.27
3	34	Open	Addition	Turf	3.43
3	34	Open	Addition	Turf	5.71
3	34	Open	Addition	Turf	2.97
3	34	Open	Addition	Turf	1.77
3	34	Open	Addition	Turf	6.16
3	34	Open	Addition	Turf	4.77
3	34	Open	Addition	Turf	5.99
3	34	Open	Addition	Turf	3.58
3	34	Open	Addition	Turf	4.33
3	34	Open	Addition	Turf	0.00
3	34	Open	Addition	Turf	11.11
3	34	Open	Addition	Turf	4.75
3	34	Open	Addition	Turf	5.21
3	34	Open	Addition	Turf	5.96
3	34	Open	Addition	Turf	9.11
3	35	Open	Addition	Turf	7.03
3	35	Open	Addition	Turf	4.28
3	35	Open	Addition	Turf	0.00
3	35	Open	Addition	Turf	2.16
3	35	Open	Addition	Turf	5.08
3	35	Open	Addition	Turf	2.02
3	35	Open	Addition	Turf	1.55
3	35	Open	Addition	Turf	6.69
3	35	Open	Addition	Turf	4.25
3	35	Open	Addition	Turf	2.48
3	35	Open	Addition	Turf	3.68
3	35	Open	Addition	Turf	2.67
3	35	Open	Addition	Turf	10.61
3	35	Open	Addition	Turf	1.12
3	35	Open	Addition	Turf	6.82
3	35	Open	Addition	Turf	7.71

3	35	Open	Addition	Turf	4.53
3	35	Open	Addition	Turf	7.73
3	35	Open	Addition	Turf	2.35
3	35	Open	Addition	Turf	3.91
3	36	Open	Addition	Turf	3.73
3	36	Open	Addition	Turf	4.75
3	36	Open	Addition	Turf	4.91
3	36	Open	Addition	Turf	4.22
3	36	Open	Addition	Turf	3.60
3	36	Open	Addition	Turf	4.10
3	36	Open	Addition	Turf	5.70
3	36	Open	Addition	Turf	1.55
3	36	Open	Addition	Turf	2.50
3	36	Open	Addition	Turf	4.75
3	36	Open	Addition	Turf	2.91
3	36	Open	Addition	Turf	3.09
3	36	Open	Addition	Turf	2.78
3	36	Open	Addition	Turf	2.66
3	36	Open	Addition	Turf	4.14
3	36	Open	Addition	Turf	4.18
3	36	Open	Addition	Turf	6.43
3	36	Open	Addition	Turf	5.01
3	36	Open	Addition	Turf	4.30
3	36	Open	Addition	Turf	4.45
4	1	Full	Natural	Turf	2.03
4	1	Full	Natural	Turf	1.83
4	1	Full	Natural	Turf	2.18
4	1	Full	Natural	Turf	2.32
4	1	Full	Natural	Turf	1.88
4	1	Full	Natural	Turf	1.82
4	1	Full	Natural	Turf	4.20
4	1	Full	Natural	Turf	15.72
4	1	Full	Natural	Turf	1.54
4	1	Full	Natural	Turf	5.89
4	1	Full	Natural	Turf	1.58
4	1	Full	Natural	Turf	2.32
4	1	Full	Natural	Turf	3.96
4	1	Full	Natural	Turf	1.85
4	1	Full	Natural	Turf	2.01
4	1	Full	Natural	Turf	1.90
4	1	Full	Natural	Turf	3.58
4	1	Full	Natural	Turf	3.03
4	1	Full	Natural	Turf	2.52
4	1	Full	Natural	Turf	5.54
4	2	Full	Natural	Turf	4.13
4	2	Full	Natural	Turf	1.68
4	2	Full	Natural	Turf	5.27
4	2	Full	Natural	Turf	2.54
4	2	Full	Natural	Turf	4.24
4	2	Full	Natural	Turf	2.25
4	2	Full	Natural	Turf	3.38
4	2	Full	Natural	Turf	7.17
4	2	Full	Natural	Turf	4.22

4	2	Full	Natural	Turf	4.80
4	2	Full	Natural	Turf	4.26
4	2	Full	Natural	Turf	1.86
4	2	Full	Natural	Turf	3.10
4	2	Full	Natural	Turf	4.95
4	2	Full	Natural	Turf	5.28
4	2	Full	Natural	Turf	4.53
4	2	Full	Natural	Turf	2.03
4	2	Full	Natural	Turf	3.10
4	2	Full	Natural	Turf	3.04
4	2	Full	Natural	Turf	2.49
4	3	Full	Natural	Turf	3.69
4	3	Full	Natural	Turf	3.70
4	3	Full	Natural	Turf	3.28
4	3	Full	Natural	Turf	2.16
4	3	Full	Natural	Turf	3.38
4	3	Full	Natural	Turf	2.21
4	3	Full	Natural	Turf	4.50
4	3	Full	Natural	Turf	4.26
4	3	Full	Natural	Turf	3.04
4	3	Full	Natural	Turf	2.04
4	3	Full	Natural	Turf	3.66
4	3	Full	Natural	Turf	4.95
4	3	Full	Natural	Turf	3.38
4	3	Full	Natural	Turf	8.68
4	3	Full	Natural	Turf	0.84
4	3	Full	Natural	Turf	3.36
4	3	Full	Natural	Turf	5.87
4	3	Full	Natural	Turf	8.06
4	3	Full	Natural	Turf	2.49
4	3	Full	Natural	Turf	3.38
4	4	Full	Addition	Turf	1.71
4	4	Full	Addition	Turf	4.55
4	4	Full	Addition	Turf	5.41
4	4	Full	Addition	Turf	2.64
4	4	Full	Addition	Turf	3.62
4	4	Full	Addition	Turf	4.65
4	4	Full	Addition	Turf	7.36
4	4	Full	Addition	Turf	2.09
4	4	Full	Addition	Turf	3.68
4	4	Full	Addition	Turf	2.60
4	4	Full	Addition	Turf	8.04
4	4	Full	Addition	Turf	6.51
4	4	Full	Addition	Turf	4.22
4	4	Full	Addition	Turf	4.45
4	4	Full	Addition	Turf	2.79
4	4	Full	Addition	Turf	2.33
4	4	Full	Addition	Turf	4.02
4	4	Full	Addition	Turf	2.64
4	4	Full	Addition	Turf	3.59
4	4	Full	Addition	Turf	2.98
4	5	Full	Addition	Turf	2.58
4	5	Full	Addition	Turf	2.77

4	5	Full	Addition	Turf	3.48
4	5	Full	Addition	Turf	4.71
4	5	Full	Addition	Turf	2.20
4	5	Full	Addition	Turf	5.41
4	5	Full	Addition	Turf	2.04
4	5	Full	Addition	Turf	5.76
4	5	Full	Addition	Turf	2.05
4	5	Full	Addition	Turf	2.30
4	5	Full	Addition	Turf	3.02
4	5	Full	Addition	Turf	2.12
4	5	Full	Addition	Turf	2.03
4	5	Full	Addition	Turf	3.00
4	5	Full	Addition	Turf	2.89
4	5	Full	Addition	Turf	8.60
4	5	Full	Addition	Turf	0.89
4	5	Full	Addition	Turf	0.65
4	5	Full	Addition	Turf	3.16
4	5	Full	Addition	Turf	20.49
4	6	Full	Addition	Turf	5.47
4	6	Full	Addition	Turf	3.82
4	6	Full	Addition	Turf	5.38
4	6	Full	Addition	Turf	9.77
4	6	Full	Addition	Turf	6.21
4	6	Full	Addition	Turf	2.17
4	6	Full	Addition	Turf	1.51
4	6	Full	Addition	Turf	4.55
4	6	Full	Addition	Turf	7.35
4	6	Full	Addition	Turf	1.71
4	6	Full	Addition	Turf	11.27
4	6	Full	Addition	Turf	3.39
4	6	Full	Addition	Turf	2.94
4	6	Full	Addition	Turf	3.77
4	6	Full	Addition	Turf	7.98
4	6	Full	Addition	Turf	5.88
4	6	Full	Addition	Turf	4.92
4	6	Full	Addition	Turf	6.86
4	6	Full	Addition	Turf	4.06
4	6	Full	Addition	Turf	5.02
4	7	Half	Natural	Turf	1.46
4	7	Half	Natural	Turf	1.66
4	7	Half	Natural	Turf	2.77
4	7	Half	Natural	Turf	2.68
4	7	Half	Natural	Turf	3.17
4	7	Half	Natural	Turf	1.13
4	7	Half	Natural	Turf	0.87
4	7	Half	Natural	Turf	1.19
4	7	Half	Natural	Turf	7.26
4	7	Half	Natural	Turf	0.48
4	7	Half	Natural	Turf	1.45
4	7	Half	Natural	Turf	1.76
4	7	Half	Natural	Turf	2.49
4	7	Half	Natural	Turf	0.83
4	7	Half	Natural	Turf	2.70

4	7	Half	Natural	Turf	1.81
4	7	Half	Natural	Turf	1.73
4	7	Half	Natural	Turf	1.72
4	7	Half	Natural	Turf	3.93
4	7	Half	Natural	Turf	1.49
4	8	Half	Natural	Turf	1.36
4	8	Half	Natural	Turf	1.20
4	8	Half	Natural	Turf	1.81
4	8	Half	Natural	Turf	2.44
4	8	Half	Natural	Turf	5.91
4	8	Half	Natural	Turf	1.90
4	8	Half	Natural	Turf	3.65
4	8	Half	Natural	Turf	1.73
4	8	Half	Natural	Turf	1.08
4	8	Half	Natural	Turf	1.12
4	8	Half	Natural	Turf	11.27
4	8	Half	Natural	Turf	2.04
4	8	Half	Natural	Turf	2.48
4	8	Half	Natural	Turf	2.10
4	8	Half	Natural	Turf	2.08
4	8	Half	Natural	Turf	2.89
4	8	Half	Natural	Turf	2.41
4	8	Half	Natural	Turf	1.28
4	8	Half	Natural	Turf	3.65
4	8	Half	Natural	Turf	1.90
4	9	Half	Natural	Turf	2.56
4	9	Half	Natural	Turf	2.75
4	9	Half	Natural	Turf	2.50
4	9	Half	Natural	Turf	3.66
4	9	Half	Natural	Turf	2.90
4	9	Half	Natural	Turf	0.00
4	9	Half	Natural	Turf	3.98
4	9	Half	Natural	Turf	5.06
4	9	Half	Natural	Turf	1.82
4	9	Half	Natural	Turf	3.35
4	9	Half	Natural	Turf	3.59
4	9	Half	Natural	Turf	3.76
4	9	Half	Natural	Turf	3.49
4	9	Half	Natural	Turf	3.82
4	9	Half	Natural	Turf	5.08
4	9	Half	Natural	Turf	4.59
4	9	Half	Natural	Turf	2.91
4	9	Half	Natural	Turf	3.87
4	9	Half	Natural	Turf	2.73
4	9	Half	Natural	Turf	1.46
4	10	Half	Addition	Turf	2.89
4	10	Half	Addition	Turf	2.80
4	10	Half	Addition	Turf	1.34
4	10	Half	Addition	Turf	3.09
4	10	Half	Addition	Turf	2.53
4	10	Half	Addition	Turf	1.81
4	10	Half	Addition	Turf	0.00
4	10	Half	Addition	Turf	3.83

4	10	Half	Addition	Turf	5.02
4	10	Half	Addition	Turf	1.24
4	10	Half	Addition	Turf	3.33
4	10	Half	Addition	Turf	3.75
4	10	Half	Addition	Turf	9.24
4	10	Half	Addition	Turf	4.08
4	10	Half	Addition	Turf	2.28
4	10	Half	Addition	Turf	2.40
4	10	Half	Addition	Turf	9.32
4	10	Half	Addition	Turf	4.18
4	10	Half	Addition	Turf	1.79
4	10	Half	Addition	Turf	2.06
4	11	Half	Addition	Turf	2.89
4	11	Half	Addition	Turf	7.22
4	11	Half	Addition	Turf	2.90
4	11	Half	Addition	Turf	2.20
4	11	Half	Addition	Turf	3.65
4	11	Half	Addition	Turf	2.51
4	11	Half	Addition	Turf	2.07
4	11	Half	Addition	Turf	2.48
4	11	Half	Addition	Turf	7.54
4	11	Half	Addition	Turf	2.24
4	11	Half	Addition	Turf	2.89
4	11	Half	Addition	Turf	5.76
4	11	Half	Addition	Turf	4.30
4	11	Half	Addition	Turf	2.63
4	11	Half	Addition	Turf	4.25
4	11	Half	Addition	Turf	1.16
4	11	Half	Addition	Turf	2.43
4	11	Half	Addition	Turf	4.39
4	11	Half	Addition	Turf	2.73
4	11	Half	Addition	Turf	4.26
4	12	Half	Addition	Turf	2.27
4	12	Half	Addition	Turf	3.45
4	12	Half	Addition	Turf	1.86
4	12	Half	Addition	Turf	3.24
4	12	Half	Addition	Turf	2.66
4	12	Half	Addition	Turf	4.01
4	12	Half	Addition	Turf	1.49
4	12	Half	Addition	Turf	4.34
4	12	Half	Addition	Turf	3.88
4	12	Half	Addition	Turf	3.45
4	12	Half	Addition	Turf	1.30
4	12	Half	Addition	Turf	2.32
4	12	Half	Addition	Turf	4.25
4	12	Half	Addition	Turf	1.85
4	12	Half	Addition	Turf	1.97
4	12	Half	Addition	Turf	2.43
4	12	Half	Addition	Turf	2.59
4	12	Half	Addition	Turf	2.40
4	12	Half	Addition	Turf	2.62
4	12	Half	Addition	Turf	4.82
4	13	Open	Natural	Turf	2.56

4	13	Open	Natural	Turf	2.66
4	13	Open	Natural	Turf	3.36
4	13	Open	Natural	Turf	2.33
4	13	Open	Natural	Turf	2.93
4	13	Open	Natural	Turf	2.50
4	13	Open	Natural	Turf	4.04
4	13	Open	Natural	Turf	2.92
4	13	Open	Natural	Turf	2.41
4	13	Open	Natural	Turf	2.35
4	13	Open	Natural	Turf	2.47
4	13	Open	Natural	Turf	1.57
4	13	Open	Natural	Turf	2.61
4	13	Open	Natural	Turf	4.27
4	13	Open	Natural	Turf	3.55
4	13	Open	Natural	Turf	2.12
4	13	Open	Natural	Turf	1.46
4	13	Open	Natural	Turf	2.29
4	13	Open	Natural	Turf	1.71
4	13	Open	Natural	Turf	2.00
4	14	Open	Natural	Turf	2.04
4	14	Open	Natural	Turf	3.06
4	14	Open	Natural	Turf	1.14
4	14	Open	Natural	Turf	1.99
4	14	Open	Natural	Turf	2.86
4	14	Open	Natural	Turf	2.27
4	14	Open	Natural	Turf	1.06
4	14	Open	Natural	Turf	1.81
4	14	Open	Natural	Turf	2.47
4	14	Open	Natural	Turf	2.43
4	14	Open	Natural	Turf	5.18
4	14	Open	Natural	Turf	5.39
4	14	Open	Natural	Turf	4.19
4	14	Open	Natural	Turf	1.72
4	14	Open	Natural	Turf	2.63
4	14	Open	Natural	Turf	2.78
4	14	Open	Natural	Turf	2.44
4	14	Open	Natural	Turf	3.57
4	14	Open	Natural	Turf	2.34
4	14	Open	Natural	Turf	4.30
4	15	Open	Natural	Turf	1.95
4	15	Open	Natural	Turf	2.97
4	15	Open	Natural	Turf	2.50
4	15	Open	Natural	Turf	1.76
4	15	Open	Natural	Turf	1.41
4	15	Open	Natural	Turf	1.71
4	15	Open	Natural	Turf	2.98
4	15	Open	Natural	Turf	2.90
4	15	Open	Natural	Turf	1.09
4	15	Open	Natural	Turf	1.92
4	15	Open	Natural	Turf	1.68
4	15	Open	Natural	Turf	1.90
4	15	Open	Natural	Turf	2.39
4	15	Open	Natural	Turf	3.51

4	15	Open	Natural	Turf	2.19
4	15	Open	Natural	Turf	2.74
4	15	Open	Natural	Turf	1.74
4	15	Open	Natural	Turf	2.43
4	15	Open	Natural	Turf	1.42
4	15	Open	Natural	Turf	6.04
4	16	Open	Addition	Turf	2.99
4	16	Open	Addition	Turf	2.79
4	16	Open	Addition	Turf	10.66
4	16	Open	Addition	Turf	1.40
4	16	Open	Addition	Turf	4.20
4	16	Open	Addition	Turf	6.34
4	16	Open	Addition	Turf	2.73
4	16	Open	Addition	Turf	5.33
4	16	Open	Addition	Turf	6.46
4	16	Open	Addition	Turf	6.50
4	16	Open	Addition	Turf	2.76
4	16	Open	Addition	Turf	2.45
4	16	Open	Addition	Turf	7.45
4	16	Open	Addition	Turf	6.48
4	16	Open	Addition	Turf	3.05
4	16	Open	Addition	Turf	3.88
4	16	Open	Addition	Turf	2.08
4	16	Open	Addition	Turf	4.59
4	16	Open	Addition	Turf	4.14
4	16	Open	Addition	Turf	32.33
4	17	Open	Addition	Turf	5.82
4	17	Open	Addition	Turf	6.97
4	17	Open	Addition	Turf	4.99
4	17	Open	Addition	Turf	1.24
4	17	Open	Addition	Turf	5.96
4	17	Open	Addition	Turf	4.03
4	17	Open	Addition	Turf	3.28
4	17	Open	Addition	Turf	6.24
4	17	Open	Addition	Turf	3.34
4	17	Open	Addition	Turf	10.96
4	17	Open	Addition	Turf	4.32
4	17	Open	Addition	Turf	3.83
4	17	Open	Addition	Turf	5.88
4	17	Open	Addition	Turf	7.20
4	17	Open	Addition	Turf	2.63
4	17	Open	Addition	Turf	4.05
4	17	Open	Addition	Turf	5.90
4	17	Open	Addition	Turf	1.12
4	17	Open	Addition	Turf	4.34
4	17	Open	Addition	Turf	2.32
4	18	Open	Addition	Turf	5.16
4	18	Open	Addition	Turf	9.34
4	18	Open	Addition	Turf	3.73
4	18	Open	Addition	Turf	2.67
4	18	Open	Addition	Turf	7.37
4	18	Open	Addition	Turf	4.38
4	18	Open	Addition	Turf	2.49

4	18	Open	Addition	Turf	1.40
4	18	Open	Addition	Turf	9.22
4	18	Open	Addition	Turf	1.37
4	18	Open	Addition	Turf	6.94
4	18	Open	Addition	Turf	12.52
4	18	Open	Addition	Turf	9.65
4	18	Open	Addition	Turf	2.98
4	18	Open	Addition	Turf	4.31
4	18	Open	Addition	Turf	1.62
4	18	Open	Addition	Turf	3.03
4	18	Open	Addition	Turf	3.45
4	18	Open	Addition	Turf	2.65
4	18	Open	Addition	Turf	1.51
4	19	Cage	Natural	Turf	2.29
4	19	Cage	Natural	Turf	5.40
4	19	Cage	Natural	Turf	5.41
4	19	Cage	Natural	Turf	2.71
4	19	Cage	Natural	Turf	7.19
4	19	Cage	Natural	Turf	2.43
4	19	Cage	Natural	Turf	4.46
4	19	Cage	Natural	Turf	16.61
4	19	Cage	Natural	Turf	3.43
4	19	Cage	Natural	Turf	7.75
4	19	Cage	Natural	Turf	5.67
4	19	Cage	Natural	Turf	8.08
4	19	Cage	Natural	Turf	1.63
4	19	Cage	Natural	Turf	4.45
4	19	Cage	Natural	Turf	4.91
4	19	Cage	Natural	Turf	2.27
4	19	Cage	Natural	Turf	5.78
4	19	Cage	Natural	Turf	4.31
4	19	Cage	Natural	Turf	3.99
4	19	Cage	Natural	Turf	3.64
4	20	Cage	Natural	Turf	3.71
4	20	Cage	Natural	Turf	9.02
4	20	Cage	Natural	Turf	2.06
4	20	Cage	Natural	Turf	6.06
4	20	Cage	Natural	Turf	1.04
4	20	Cage	Natural	Turf	7.42
4	20	Cage	Natural	Turf	3.24
4	20	Cage	Natural	Turf	2.09
4	20	Cage	Natural	Turf	2.44
4	20	Cage	Natural	Turf	2.07
4	20	Cage	Natural	Turf	2.21
4	20	Cage	Natural	Turf	2.77
4	20	Cage	Natural	Turf	2.01
4	20	Cage	Natural	Turf	3.16
4	20	Cage	Natural	Turf	4.00
4	20	Cage	Natural	Turf	3.69
4	20	Cage	Natural	Turf	3.32
4	20	Cage	Natural	Turf	5.38
4	20	Cage	Natural	Turf	2.77
4	20	Cage	Natural	Turf	5.16

4	21	Cage	Natural	Turf	3.64
4	21	Cage	Natural	Turf	1.44
4	21	Cage	Natural	Turf	2.34
4	21	Cage	Natural	Turf	3.63
4	21	Cage	Natural	Turf	2.51
4	21	Cage	Natural	Turf	2.01
4	21	Cage	Natural	Turf	2.28
4	21	Cage	Natural	Turf	3.57
4	21	Cage	Natural	Turf	4.52
4	21	Cage	Natural	Turf	4.65
4	21	Cage	Natural	Turf	8.55
4	21	Cage	Natural	Turf	3.43
4	21	Cage	Natural	Turf	4.75
4	21	Cage	Natural	Turf	5.47
4	21	Cage	Natural	Turf	7.65
4	21	Cage	Natural	Turf	4.51
4	21	Cage	Natural	Turf	3.95
4	21	Cage	Natural	Turf	1.08
4	21	Cage	Natural	Turf	4.79
4	21	Cage	Natural	Turf	2.69
4	22	Cage	Addition	Turf	3.67
4	22	Cage	Addition	Turf	0.33
4	22	Cage	Addition	Turf	7.24
4	22	Cage	Addition	Turf	5.72
4	22	Cage	Addition	Turf	6.63
4	22	Cage	Addition	Turf	8.35
4	22	Cage	Addition	Turf	6.93
4	22	Cage	Addition	Turf	8.37
4	22	Cage	Addition	Turf	6.75
4	22	Cage	Addition	Turf	5.58
4	22	Cage	Addition	Turf	4.91
4	22	Cage	Addition	Turf	5.46
4	22	Cage	Addition	Turf	1.77
4	22	Cage	Addition	Turf	2.49
4	22	Cage	Addition	Turf	7.40
4	22	Cage	Addition	Turf	0.00
4	22	Cage	Addition	Turf	0.44
4	22	Cage	Addition	Turf	0.00
4	22	Cage	Addition	Turf	0.51
4	22	Cage	Addition	Turf	0.00
4	23	Cage	Addition	Turf	0.00
4	23	Cage	Addition	Turf	3.75
4	23	Cage	Addition	Turf	3.72
4	23	Cage	Addition	Turf	0.99
4	23	Cage	Addition	Turf	1.64
4	23	Cage	Addition	Turf	3.20
4	23	Cage	Addition	Turf	0.00
4	23	Cage	Addition	Turf	3.16
4	23	Cage	Addition	Turf	1.33
4	23	Cage	Addition	Turf	13.55
4	23	Cage	Addition	Turf	7.40
4	23	Cage	Addition	Turf	1.24
4	23	Cage	Addition	Turf	0.00

4	23	Cage	Addition	Turf	1.84
4	23	Cage	Addition	Turf	1.88
4	23	Cage	Addition	Turf	1.16
4	23	Cage	Addition	Turf	0.00
4	23	Cage	Addition	Turf	4.26
4	23	Cage	Addition	Turf	4.97
4	23	Cage	Addition	Turf	4.71
4	24	Cage	Addition	Turf	10.59
4	24	Cage	Addition	Turf	1.37
4	24	Cage	Addition	Turf	6.78
4	24	Cage	Addition	Turf	4.12
4	24	Cage	Addition	Turf	2.63
4	24	Cage	Addition	Turf	18.16
4	24	Cage	Addition	Turf	7.98
4	24	Cage	Addition	Turf	6.71
4	24	Cage	Addition	Turf	3.84
4	24	Cage	Addition	Turf	2.85
4	24	Cage	Addition	Turf	0.00
4	24	Cage	Addition	Turf	3.69
4	24	Cage	Addition	Turf	1.70
4	24	Cage	Addition	Turf	0.00
4	24	Cage	Addition	Turf	8.59
4	24	Cage	Addition	Turf	4.96
4	24	Cage	Addition	Turf	3.84
4	24	Cage	Addition	Turf	6.11
4	24	Cage	Addition	Turf	21.18
4	24	Cage	Addition	Turf	7.49
4	25	Half	Natural	Turf	1.95
4	25	Half	Natural	Turf	3.83
4	25	Half	Natural	Turf	7.50
4	25	Half	Natural	Turf	5.79
4	25	Half	Natural	Turf	4.97
4	25	Half	Natural	Turf	4.69
4	25	Half	Natural	Turf	8.07
4	25	Half	Natural	Turf	10.63
4	25	Half	Natural	Turf	4.73
4	25	Half	Natural	Turf	5.36
4	25	Half	Natural	Turf	2.50
4	25	Half	Natural	Turf	2.39
4	25	Half	Natural	Turf	2.40
4	25	Half	Natural	Turf	2.37
4	25	Half	Natural	Turf	3.06
4	25	Half	Natural	Turf	5.40
4	25	Half	Natural	Turf	5.01
4	25	Half	Natural	Turf	2.24
4	25	Half	Natural	Turf	2.48
4	25	Half	Natural	Turf	3.91
4	26	Half	Natural	Turf	3.11
4	26	Half	Natural	Turf	1.42
4	26	Half	Natural	Turf	3.91
4	26	Half	Natural	Turf	5.50
4	26	Half	Natural	Turf	3.98
4	26	Half	Natural	Turf	2.83

4	26	Half	Natural	Turf	8.84
4	26	Half	Natural	Turf	4.22
4	26	Half	Natural	Turf	2.82
4	26	Half	Natural	Turf	2.22
4	26	Half	Natural	Turf	3.56
4	26	Half	Natural	Turf	4.80
4	26	Half	Natural	Turf	6.27
4	26	Half	Natural	Turf	2.44
4	26	Half	Natural	Turf	2.26
4	26	Half	Natural	Turf	3.37
4	26	Half	Natural	Turf	2.61
4	26	Half	Natural	Turf	2.53
4	26	Half	Natural	Turf	2.93
4	26	Half	Natural	Turf	4.18
4	27	Half	Natural	Turf	2.19
4	27	Half	Natural	Turf	1.74
4	27	Half	Natural	Turf	4.24
4	27	Half	Natural	Turf	4.19
4	27	Half	Natural	Turf	4.67
4	27	Half	Natural	Turf	2.54
4	27	Half	Natural	Turf	1.77
4	27	Half	Natural	Turf	7.62
4	27	Half	Natural	Turf	1.51
4	27	Half	Natural	Turf	2.18
4	27	Half	Natural	Turf	2.76
4	27	Half	Natural	Turf	3.72
4	27	Half	Natural	Turf	2.86
4	27	Half	Natural	Turf	2.19
4	27	Half	Natural	Turf	1.78
4	27	Half	Natural	Turf	2.76
4	27	Half	Natural	Turf	2.02
4	27	Half	Natural	Turf	2.75
4	27	Half	Natural	Turf	3.12
4	27	Half	Natural	Turf	3.24
4	28	Half	Addition	Turf	1.91
4	28	Half	Addition	Turf	2.27
4	28	Half	Addition	Turf	2.63
4	28	Half	Addition	Turf	1.82
4	28	Half	Addition	Turf	3.69
4	28	Half	Addition	Turf	3.71
4	28	Half	Addition	Turf	1.46
4	28	Half	Addition	Turf	0.53
4	28	Half	Addition	Turf	1.71
4	28	Half	Addition	Turf	2.17
4	28	Half	Addition	Turf	0.91
4	28	Half	Addition	Turf	0.87
4	28	Half	Addition	Turf	0.39
4	28	Half	Addition	Turf	0.00
4	28	Half	Addition	Turf	2.93
4	28	Half	Addition	Turf	1.66
4	28	Half	Addition	Turf	0.00
4	28	Half	Addition	Turf	2.63
4	28	Half	Addition	Turf	3.23

4	28	Half	Addition	Turf	7.38
4	29	Half	Addition	Turf	5.35
4	29	Half	Addition	Turf	1.64
4	29	Half	Addition	Turf	2.76
4	29	Half	Addition	Turf	3.99
4	29	Half	Addition	Turf	2.87
4	29	Half	Addition	Turf	2.57
4	29	Half	Addition	Turf	5.52
4	29	Half	Addition	Turf	1.80
4	29	Half	Addition	Turf	1.60
4	29	Half	Addition	Turf	3.25
4	29	Half	Addition	Turf	3.52
4	29	Half	Addition	Turf	4.70
4	29	Half	Addition	Turf	0.00
4	29	Half	Addition	Turf	1.80
4	29	Half	Addition	Turf	2.91
4	29	Half	Addition	Turf	3.78
4	29	Half	Addition	Turf	1.78
4	29	Half	Addition	Turf	4.92
4	29	Half	Addition	Turf	1.29
4	29	Half	Addition	Turf	3.35
4	30	Half	Addition	Turf	2.52
4	30	Half	Addition	Turf	2.65
4	30	Half	Addition	Turf	1.14
4	30	Half	Addition	Turf	2.35
4	30	Half	Addition	Turf	1.76
4	30	Half	Addition	Turf	4.72
4	30	Half	Addition	Turf	0.00
4	30	Half	Addition	Turf	0.00
4	30	Half	Addition	Turf	3.27
4	30	Half	Addition	Turf	17.13
4	30	Half	Addition	Turf	5.13
4	30	Half	Addition	Turf	4.02
4	30	Half	Addition	Turf	7.19
4	30	Half	Addition	Turf	1.67
4	30	Half	Addition	Turf	1.57
4	30	Half	Addition	Turf	2.61
4	30	Half	Addition	Turf	2.22
4	30	Half	Addition	Turf	4.98
4	30	Half	Addition	Turf	6.40
4	30	Half	Addition	Turf	7.48
4	31	Open	Natural	Turf	2.65
4	31	Open	Natural	Turf	5.10
4	31	Open	Natural	Turf	2.74
4	31	Open	Natural	Turf	4.71
4	31	Open	Natural	Turf	2.87
4	31	Open	Natural	Turf	3.58
4	31	Open	Natural	Turf	6.26
4	31	Open	Natural	Turf	5.16
4	31	Open	Natural	Turf	14.30
4	31	Open	Natural	Turf	4.24
4	31	Open	Natural	Turf	4.34
4	31	Open	Natural	Turf	5.68

4	31	Open	Natural	Turf	3.36
4	31	Open	Natural	Turf	2.70
4	31	Open	Natural	Turf	3.03
4	31	Open	Natural	Turf	3.12
4	31	Open	Natural	Turf	5.00
4	31	Open	Natural	Turf	2.23
4	31	Open	Natural	Turf	2.97
4	31	Open	Natural	Turf	6.28
4	32	Open	Natural	Turf	2.70
4	32	Open	Natural	Turf	2.09
4	32	Open	Natural	Turf	1.66
4	32	Open	Natural	Turf	4.29
4	32	Open	Natural	Turf	2.14
4	32	Open	Natural	Turf	3.73
4	32	Open	Natural	Turf	2.10
4	32	Open	Natural	Turf	2.33
4	32	Open	Natural	Turf	2.70
4	32	Open	Natural	Turf	2.43
4	32	Open	Natural	Turf	4.85
4	32	Open	Natural	Turf	2.69
4	32	Open	Natural	Turf	2.48
4	32	Open	Natural	Turf	4.73
4	32	Open	Natural	Turf	2.82
4	32	Open	Natural	Turf	2.36
4	32	Open	Natural	Turf	3.25
4	32	Open	Natural	Turf	2.82
4	32	Open	Natural	Turf	2.38
4	32	Open	Natural	Turf	3.12
4	33	Open	Natural	Turf	4.45
4	33	Open	Natural	Turf	12.64
4	33	Open	Natural	Turf	2.22
4	33	Open	Natural	Turf	2.00
4	33	Open	Natural	Turf	3.34
4	33	Open	Natural	Turf	1.70
4	33	Open	Natural	Turf	3.18
4	33	Open	Natural	Turf	1.78
4	33	Open	Natural	Turf	2.11
4	33	Open	Natural	Turf	5.11
4	33	Open	Natural	Turf	1.93
4	33	Open	Natural	Turf	3.02
4	33	Open	Natural	Turf	2.28
4	33	Open	Natural	Turf	2.00
4	33	Open	Natural	Turf	2.14
4	33	Open	Natural	Turf	2.60
4	33	Open	Natural	Turf	2.35
4	33	Open	Natural	Turf	2.48
4	33	Open	Natural	Turf	2.94
4	33	Open	Natural	Turf	1.97
4	34	Open	Addition	Turf	5.75
4	34	Open	Addition	Turf	8.36
4	34	Open	Addition	Turf	6.43
4	34	Open	Addition	Turf	4.68
4	34	Open	Addition	Turf	4.94

4	34 Open	Addition	Turf	0.00
4	34 Open	Addition	Turf	9.05
4	34 Open	Addition	Turf	12.10
4	34 Open	Addition	Turf	5.60
4	34 Open	Addition	Turf	11.50
4	34 Open	Addition	Turf	6.73
4	34 Open	Addition	Turf	9.10
4	34 Open	Addition	Turf	7.05
4	34 Open	Addition	Turf	5.80
4	34 Open	Addition	Turf	3.98
4	34 Open	Addition	Turf	9.91
4	34 Open	Addition	Turf	7.90
4	34 Open	Addition	Turf	4.53
4	34 Open	Addition	Turf	4.80
4	34 Open	Addition	Turf	5.75
4	35 Open	Addition	Turf	3.36
4	35 Open	Addition	Turf	9.76
4	35 Open	Addition	Turf	2.41
4	35 Open	Addition	Turf	1.56
4	35 Open	Addition	Turf	2.32
4	35 Open	Addition	Turf	4.45
4	35 Open	Addition	Turf	2.66
4	35 Open	Addition	Turf	10.47
4	35 Open	Addition	Turf	0.00
4	35 Open	Addition	Turf	17.07
4	35 Open	Addition	Turf	11.16
4	35 Open	Addition	Turf	9.65
4	35 Open	Addition	Turf	2.91
4	35 Open	Addition	Turf	0.00
4	35 Open	Addition	Turf	2.42
4	35 Open	Addition	Turf	2.32
4	35 Open	Addition	Turf	2.24
4	35 Open	Addition	Turf	1.94
4	35 Open	Addition	Turf	1.86
4	35 Open	Addition	Turf	3.64
4	36 Open	Addition	Turf	2.36
4	36 Open	Addition	Turf	3.71
4	36 Open	Addition	Turf	6.66
4	36 Open	Addition	Turf	4.19
4	36 Open	Addition	Turf	3.03
4	36 Open	Addition	Turf	4.94
4	36 Open	Addition	Turf	2.64
4	36 Open	Addition	Turf	7.50
4	36 Open	Addition	Turf	4.52
4	36 Open	Addition	Turf	3.61
4	36 Open	Addition	Turf	1.66
4	36 Open	Addition	Turf	3.75
4	36 Open	Addition	Turf	3.13
4	36 Open	Addition	Turf	3.99
4	36 Open	Addition	Turf	2.91
4	36 Open	Addition	Turf	3.97
4	36 Open	Addition	Turf	3.12
4	36 Open	Addition	Turf	5.76

4	36	Open	Addition	Turf	7.08
4	36	Open	Addition	Turf	2.08
5	1	Full	Natural	Turf	3.03
5	1	Full	Natural	Turf	1.95
5	1	Full	Natural	Turf	2.90
5	1	Full	Natural	Turf	3.39
5	1	Full	Natural	Turf	3.99
5	1	Full	Natural	Turf	1.70
5	1	Full	Natural	Turf	2.18
5	1	Full	Natural	Turf	6.58
5	1	Full	Natural	Turf	2.38
5	1	Full	Natural	Turf	3.33
5	1	Full	Natural	Turf	11.09
5	1	Full	Natural	Turf	1.86
5	1	Full	Natural	Turf	1.10
5	1	Full	Natural	Turf	3.27
5	1	Full	Natural	Turf	3.82
5	1	Full	Natural	Turf	4.14
5	1	Full	Natural	Turf	5.18
5	1	Full	Natural	Turf	2.29
5	1	Full	Natural	Turf	2.01
5	1	Full	Natural	Turf	8.97
5	2	Full	Natural	Turf	3.66
5	2	Full	Natural	Turf	2.67
5	2	Full	Natural	Turf	3.76
5	2	Full	Natural	Turf	3.89
5	2	Full	Natural	Turf	2.43
5	2	Full	Natural	Turf	2.58
5	2	Full	Natural	Turf	3.18
5	2	Full	Natural	Turf	2.19
5	2	Full	Natural	Turf	3.69
5	2	Full	Natural	Turf	3.67
5	2	Full	Natural	Turf	2.99
5	2	Full	Natural	Turf	5.01
5	2	Full	Natural	Turf	4.80
5	2	Full	Natural	Turf	2.42
5	2	Full	Natural	Turf	6.06
5	2	Full	Natural	Turf	6.21
5	2	Full	Natural	Turf	2.70
5	2	Full	Natural	Turf	3.37
5	2	Full	Natural	Turf	4.16
5	2	Full	Natural	Turf	3.24
5	3	Full	Natural	Turf	6.28
5	3	Full	Natural	Turf	2.77
5	3	Full	Natural	Turf	4.27
5	3	Full	Natural	Turf	2.39
5	3	Full	Natural	Turf	3.71
5	3	Full	Natural	Turf	5.38
5	3	Full	Natural	Turf	2.65
5	3	Full	Natural	Turf	5.58
5	3	Full	Natural	Turf	2.78
5	3	Full	Natural	Turf	3.65
5	3	Full	Natural	Turf	4.25

5	3	Full	Natural	Turf	3.52
5	3	Full	Natural	Turf	3.13
5	3	Full	Natural	Turf	3.63
5	3	Full	Natural	Turf	2.76
5	3	Full	Natural	Turf	6.20
5	3	Full	Natural	Turf	5.80
5	3	Full	Natural	Turf	2.79
5	3	Full	Natural	Turf	1.87
5	3	Full	Natural	Turf	6.12
5	4	Full	Addition	Turf	3.06
5	4	Full	Addition	Turf	3.38
5	4	Full	Addition	Turf	4.08
5	4	Full	Addition	Turf	2.33
5	4	Full	Addition	Turf	3.66
5	4	Full	Addition	Turf	4.51
5	4	Full	Addition	Turf	13.62
5	4	Full	Addition	Turf	8.76
5	4	Full	Addition	Turf	10.46
5	4	Full	Addition	Turf	2.97
5	4	Full	Addition	Turf	3.16
5	4	Full	Addition	Turf	6.91
5	4	Full	Addition	Turf	13.53
5	4	Full	Addition	Turf	6.25
5	4	Full	Addition	Turf	19.75
5	4	Full	Addition	Turf	1.42
5	4	Full	Addition	Turf	2.88
5	4	Full	Addition	Turf	3.67
5	4	Full	Addition	Turf	3.78
5	4	Full	Addition	Turf	2.51
5	5	Full	Addition	Turf	4.97
5	5	Full	Addition	Turf	3.65
5	5	Full	Addition	Turf	4.98
5	5	Full	Addition	Turf	1.86
5	5	Full	Addition	Turf	2.51
5	5	Full	Addition	Turf	2.87
5	5	Full	Addition	Turf	2.65
5	5	Full	Addition	Turf	3.62
5	5	Full	Addition	Turf	2.76
5	5	Full	Addition	Turf	7.66
5	5	Full	Addition	Turf	19.02
5	5	Full	Addition	Turf	7.42
5	5	Full	Addition	Turf	3.92
5	5	Full	Addition	Turf	3.47
5	5	Full	Addition	Turf	3.80
5	5	Full	Addition	Turf	3.68
5	5	Full	Addition	Turf	2.95
5	5	Full	Addition	Turf	3.11
5	5	Full	Addition	Turf	4.20
5	5	Full	Addition	Turf	6.35
5	6	Full	Addition	Turf	6.99
5	6	Full	Addition	Turf	1.95
5	6	Full	Addition	Turf	4.03
5	6	Full	Addition	Turf	7.27

5	6	Full	Addition	Turf	2.93
5	6	Full	Addition	Turf	8.06
5	6	Full	Addition	Turf	3.61
5	6	Full	Addition	Turf	3.56
5	6	Full	Addition	Turf	3.54
5	6	Full	Addition	Turf	8.16
5	6	Full	Addition	Turf	3.64
5	6	Full	Addition	Turf	2.15
5	6	Full	Addition	Turf	1.84
5	6	Full	Addition	Turf	3.59
5	6	Full	Addition	Turf	1.81
5	6	Full	Addition	Turf	1.77
5	6	Full	Addition	Turf	5.04
5	6	Full	Addition	Turf	5.44
5	6	Full	Addition	Turf	1.14
5	6	Full	Addition	Turf	3.03
5	7	Half	Natural	Turf	1.65
5	7	Half	Natural	Turf	3.12
5	7	Half	Natural	Turf	3.78
5	7	Half	Natural	Turf	2.12
5	7	Half	Natural	Turf	1.63
5	7	Half	Natural	Turf	3.68
5	7	Half	Natural	Turf	4.19
5	7	Half	Natural	Turf	3.77
5	7	Half	Natural	Turf	2.80
5	7	Half	Natural	Turf	1.94
5	7	Half	Natural	Turf	2.06
5	7	Half	Natural	Turf	2.03
5	7	Half	Natural	Turf	3.06
5	7	Half	Natural	Turf	6.89
5	7	Half	Natural	Turf	1.19
5	7	Half	Natural	Turf	1.50
5	7	Half	Natural	Turf	4.75
5	7	Half	Natural	Turf	2.55
5	7	Half	Natural	Turf	1.40
5	7	Half	Natural	Turf	2.65
5	8	Half	Natural	Turf	1.46
5	8	Half	Natural	Turf	1.80
5	8	Half	Natural	Turf	2.09
5	8	Half	Natural	Turf	13.53
5	8	Half	Natural	Turf	2.28
5	8	Half	Natural	Turf	2.43
5	8	Half	Natural	Turf	1.85
5	8	Half	Natural	Turf	1.33
5	8	Half	Natural	Turf	2.10
5	8	Half	Natural	Turf	1.32
5	8	Half	Natural	Turf	2.38
5	8	Half	Natural	Turf	4.15
5	8	Half	Natural	Turf	1.51
5	8	Half	Natural	Turf	3.97
5	8	Half	Natural	Turf	5.23
5	8	Half	Natural	Turf	1.52
5	8	Half	Natural	Turf	3.17

5	8	Half	Natural	Turf	1.16
5	8	Half	Natural	Turf	1.60
5	8	Half	Natural	Turf	1.94
5	9	Half	Natural	Turf	4.09
5	9	Half	Natural	Turf	2.48
5	9	Half	Natural	Turf	1.82
5	9	Half	Natural	Turf	2.16
5	9	Half	Natural	Turf	3.68
5	9	Half	Natural	Turf	4.77
5	9	Half	Natural	Turf	2.43
5	9	Half	Natural	Turf	1.60
5	9	Half	Natural	Turf	2.22
5	9	Half	Natural	Turf	2.46
5	9	Half	Natural	Turf	4.03
5	9	Half	Natural	Turf	2.22
5	9	Half	Natural	Turf	5.09
5	9	Half	Natural	Turf	1.72
5	9	Half	Natural	Turf	1.75
5	9	Half	Natural	Turf	2.78
5	9	Half	Natural	Turf	3.42
5	9	Half	Natural	Turf	1.35
5	9	Half	Natural	Turf	1.59
5	9	Half	Natural	Turf	18.62
5	10	Half	Addition	Turf	2.73
5	10	Half	Addition	Turf	3.14
5	10	Half	Addition	Turf	1.90
5	10	Half	Addition	Turf	9.01
5	10	Half	Addition	Turf	2.58
5	10	Half	Addition	Turf	3.37
5	10	Half	Addition	Turf	1.38
5	10	Half	Addition	Turf	2.18
5	10	Half	Addition	Turf	1.82
5	10	Half	Addition	Turf	1.59
5	10	Half	Addition	Turf	2.32
5	10	Half	Addition	Turf	4.12
5	10	Half	Addition	Turf	2.16
5	10	Half	Addition	Turf	3.04
5	10	Half	Addition	Turf	3.55
5	10	Half	Addition	Turf	3.58
5	10	Half	Addition	Turf	2.70
5	10	Half	Addition	Turf	3.11
5	10	Half	Addition	Turf	1.36
5	10	Half	Addition	Turf	1.59
5	11	Half	Addition	Turf	3.16
5	11	Half	Addition	Turf	2.51
5	11	Half	Addition	Turf	3.64
5	11	Half	Addition	Turf	6.86
5	11	Half	Addition	Turf	4.31
5	11	Half	Addition	Turf	9.87
5	11	Half	Addition	Turf	4.76
5	11	Half	Addition	Turf	2.61
5	11	Half	Addition	Turf	3.94
5	11	Half	Addition	Turf	4.34

5	11	Half	Addition	Turf	2.18
5	11	Half	Addition	Turf	2.05
5	11	Half	Addition	Turf	1.82
5	11	Half	Addition	Turf	1.71
5	11	Half	Addition	Turf	2.48
5	11	Half	Addition	Turf	2.38
5	11	Half	Addition	Turf	1.20
5	11	Half	Addition	Turf	0.00
5	11	Half	Addition	Turf	2.08
5	11	Half	Addition	Turf	1.15
5	12	Half	Addition	Turf	3.14
5	12	Half	Addition	Turf	1.61
5	12	Half	Addition	Turf	3.78
5	12	Half	Addition	Turf	2.57
5	12	Half	Addition	Turf	4.28
5	12	Half	Addition	Turf	4.98
5	12	Half	Addition	Turf	3.16
5	12	Half	Addition	Turf	3.72
5	12	Half	Addition	Turf	3.89
5	12	Half	Addition	Turf	2.17
5	12	Half	Addition	Turf	3.54
5	12	Half	Addition	Turf	4.78
5	12	Half	Addition	Turf	2.01
5	12	Half	Addition	Turf	4.55
5	12	Half	Addition	Turf	2.37
5	12	Half	Addition	Turf	2.50
5	12	Half	Addition	Turf	1.90
5	12	Half	Addition	Turf	2.60
5	12	Half	Addition	Turf	3.31
5	12	Half	Addition	Turf	5.54
5	13	Open	Natural	Turf	2.50
5	13	Open	Natural	Turf	2.16
5	13	Open	Natural	Turf	1.23
5	13	Open	Natural	Turf	2.35
5	13	Open	Natural	Turf	2.79
5	13	Open	Natural	Turf	2.04
5	13	Open	Natural	Turf	1.20
5	13	Open	Natural	Turf	9.73
5	13	Open	Natural	Turf	3.19
5	13	Open	Natural	Turf	1.65
5	13	Open	Natural	Turf	1.57
5	13	Open	Natural	Turf	1.73
5	13	Open	Natural	Turf	1.76
5	13	Open	Natural	Turf	1.98
5	13	Open	Natural	Turf	3.08
5	13	Open	Natural	Turf	1.14
5	13	Open	Natural	Turf	2.16
5	13	Open	Natural	Turf	2.00
5	13	Open	Natural	Turf	3.03
5	13	Open	Natural	Turf	2.76
5	14	Open	Natural	Turf	5.33
5	14	Open	Natural	Turf	2.82
5	14	Open	Natural	Turf	1.74

5	14	Open	Natural	Turf	1.46
5	14	Open	Natural	Turf	1.79
5	14	Open	Natural	Turf	2.81
5	14	Open	Natural	Turf	3.29
5	14	Open	Natural	Turf	2.81
5	14	Open	Natural	Turf	2.12
5	14	Open	Natural	Turf	3.75
5	14	Open	Natural	Turf	14.99
5	14	Open	Natural	Turf	2.02
5	14	Open	Natural	Turf	1.99
5	14	Open	Natural	Turf	2.93
5	14	Open	Natural	Turf	3.73
5	14	Open	Natural	Turf	1.42
5	14	Open	Natural	Turf	1.40
5	14	Open	Natural	Turf	2.93
5	14	Open	Natural	Turf	1.80
5	14	Open	Natural	Turf	2.56
5	15	Open	Natural	Turf	2.65
5	15	Open	Natural	Turf	2.09
5	15	Open	Natural	Turf	0.79
5	15	Open	Natural	Turf	2.06
5	15	Open	Natural	Turf	4.37
5	15	Open	Natural	Turf	1.41
5	15	Open	Natural	Turf	2.25
5	15	Open	Natural	Turf	2.01
5	15	Open	Natural	Turf	2.38
5	15	Open	Natural	Turf	2.27
5	15	Open	Natural	Turf	2.12
5	15	Open	Natural	Turf	1.42
5	15	Open	Natural	Turf	1.82
5	15	Open	Natural	Turf	2.09
5	15	Open	Natural	Turf	3.67
5	15	Open	Natural	Turf	1.68
5	15	Open	Natural	Turf	2.39
5	15	Open	Natural	Turf	2.00
5	15	Open	Natural	Turf	1.92
5	15	Open	Natural	Turf	2.07
5	16	Open	Addition	Turf	2.77
5	16	Open	Addition	Turf	3.17
5	16	Open	Addition	Turf	2.97
5	16	Open	Addition	Turf	1.81
5	16	Open	Addition	Turf	1.23
5	16	Open	Addition	Turf	2.43
5	16	Open	Addition	Turf	1.28
5	16	Open	Addition	Turf	2.76
5	16	Open	Addition	Turf	1.93
5	16	Open	Addition	Turf	2.38
5	16	Open	Addition	Turf	2.80
5	16	Open	Addition	Turf	6.20
5	16	Open	Addition	Turf	1.82
5	16	Open	Addition	Turf	2.78
5	16	Open	Addition	Turf	1.14
5	16	Open	Addition	Turf	4.11

5	16	Open	Addition	Turf	2.33
5	16	Open	Addition	Turf	3.84
5	16	Open	Addition	Turf	3.16
5	16	Open	Addition	Turf	4.50
5	17	Open	Addition	Turf	3.61
5	17	Open	Addition	Turf	5.89
5	17	Open	Addition	Turf	4.22
5	17	Open	Addition	Turf	2.49
5	17	Open	Addition	Turf	4.38
5	17	Open	Addition	Turf	2.09
5	17	Open	Addition	Turf	2.23
5	17	Open	Addition	Turf	2.38
5	17	Open	Addition	Turf	5.79
5	17	Open	Addition	Turf	2.96
5	17	Open	Addition	Turf	3.19
5	17	Open	Addition	Turf	4.82
5	17	Open	Addition	Turf	3.78
5	17	Open	Addition	Turf	4.58
5	17	Open	Addition	Turf	4.27
5	17	Open	Addition	Turf	4.68
5	17	Open	Addition	Turf	2.39
5	17	Open	Addition	Turf	0.00
5	17	Open	Addition	Turf	3.19
5	17	Open	Addition	Turf	6.12
5	18	Open	Addition	Turf	2.40
5	18	Open	Addition	Turf	0.93
5	18	Open	Addition	Turf	1.40
5	18	Open	Addition	Turf	1.88
5	18	Open	Addition	Turf	3.37
5	18	Open	Addition	Turf	1.16
5	18	Open	Addition	Turf	1.78
5	18	Open	Addition	Turf	3.06
5	18	Open	Addition	Turf	5.31
5	18	Open	Addition	Turf	3.53
5	18	Open	Addition	Turf	0.85
5	18	Open	Addition	Turf	0.93
5	18	Open	Addition	Turf	1.61
5	18	Open	Addition	Turf	1.26
5	18	Open	Addition	Turf	1.38
5	18	Open	Addition	Turf	3.50
5	18	Open	Addition	Turf	2.27
5	18	Open	Addition	Turf	2.28
5	18	Open	Addition	Turf	4.35
5	18	Open	Addition	Turf	3.26
5	19	Cage	Natural	Turf	4.11
5	19	Cage	Natural	Turf	2.23
5	19	Cage	Natural	Turf	4.45
5	19	Cage	Natural	Turf	6.16
5	19	Cage	Natural	Turf	4.85
5	19	Cage	Natural	Turf	1.87
5	19	Cage	Natural	Turf	2.12
5	19	Cage	Natural	Turf	3.09
5	19	Cage	Natural	Turf	4.83

5	19	Cage	Natural	Turf	12.28
5	19	Cage	Natural	Turf	8.95
5	19	Cage	Natural	Turf	7.42
5	19	Cage	Natural	Turf	3.63
5	19	Cage	Natural	Turf	1.65
5	19	Cage	Natural	Turf	4.21
5	19	Cage	Natural	Turf	3.27
5	19	Cage	Natural	Turf	3.81
5	19	Cage	Natural	Turf	1.51
5	19	Cage	Natural	Turf	2.23
5	19	Cage	Natural	Turf	3.88
5	20	Cage	Natural	Turf	2.80
5	20	Cage	Natural	Turf	1.36
5	20	Cage	Natural	Turf	4.06
5	20	Cage	Natural	Turf	3.33
5	20	Cage	Natural	Turf	4.49
5	20	Cage	Natural	Turf	2.83
5	20	Cage	Natural	Turf	2.28
5	20	Cage	Natural	Turf	3.27
5	20	Cage	Natural	Turf	0.00
5	20	Cage	Natural	Turf	2.87
5	20	Cage	Natural	Turf	1.60
5	20	Cage	Natural	Turf	2.35
5	20	Cage	Natural	Turf	6.73
5	20	Cage	Natural	Turf	2.41
5	20	Cage	Natural	Turf	1.93
5	20	Cage	Natural	Turf	3.41
5	20	Cage	Natural	Turf	2.15
5	20	Cage	Natural	Turf	3.26
5	20	Cage	Natural	Turf	4.38
5	20	Cage	Natural	Turf	1.45
5	21	Cage	Natural	Turf	3.07
5	21	Cage	Natural	Turf	5.27
5	21	Cage	Natural	Turf	3.62
5	21	Cage	Natural	Turf	3.86
5	21	Cage	Natural	Turf	13.46
5	21	Cage	Natural	Turf	1.67
5	21	Cage	Natural	Turf	1.21
5	21	Cage	Natural	Turf	1.95
5	21	Cage	Natural	Turf	4.15
5	21	Cage	Natural	Turf	1.23
5	21	Cage	Natural	Turf	3.62
5	21	Cage	Natural	Turf	3.53
5	21	Cage	Natural	Turf	1.33
5	21	Cage	Natural	Turf	3.70
5	21	Cage	Natural	Turf	4.49
5	21	Cage	Natural	Turf	4.05
5	21	Cage	Natural	Turf	6.22
5	21	Cage	Natural	Turf	1.05
5	21	Cage	Natural	Turf	1.24
5	21	Cage	Natural	Turf	2.16
5	22	Cage	Addition	Turf	2.49
5	22	Cage	Addition	Turf	0.70

5	22	Cage	Addition	Turf	4.15
5	22	Cage	Addition	Turf	3.25
5	22	Cage	Addition	Turf	5.38
5	22	Cage	Addition	Turf	3.06
5	22	Cage	Addition	Turf	2.25
5	22	Cage	Addition	Turf	2.62
5	22	Cage	Addition	Turf	1.68
5	22	Cage	Addition	Turf	5.82
5	22	Cage	Addition	Turf	13.85
5	22	Cage	Addition	Turf	9.92
5	22	Cage	Addition	Turf	7.34
5	22	Cage	Addition	Turf	6.20
5	22	Cage	Addition	Turf	4.91
5	22	Cage	Addition	Turf	4.94
5	22	Cage	Addition	Turf	1.14
5	22	Cage	Addition	Turf	4.87
5	22	Cage	Addition	Turf	1.93
5	22	Cage	Addition	Turf	5.39
5	23	Cage	Addition	Turf	3.39
5	23	Cage	Addition	Turf	5.59
5	23	Cage	Addition	Turf	16.04
5	23	Cage	Addition	Turf	7.20
5	23	Cage	Addition	Turf	3.71
5	23	Cage	Addition	Turf	1.53
5	23	Cage	Addition	Turf	3.03
5	23	Cage	Addition	Turf	4.12
5	23	Cage	Addition	Turf	2.97
5	23	Cage	Addition	Turf	4.01
5	23	Cage	Addition	Turf	16.19
5	23	Cage	Addition	Turf	3.50
5	23	Cage	Addition	Turf	1.43
5	23	Cage	Addition	Turf	0.65
5	23	Cage	Addition	Turf	1.09
5	23	Cage	Addition	Turf	1.09
5	23	Cage	Addition	Turf	3.40
5	23	Cage	Addition	Turf	2.81
5	23	Cage	Addition	Turf	4.22
5	23	Cage	Addition	Turf	2.94
5	24	Cage	Addition	Turf	5.05
5	24	Cage	Addition	Turf	3.71
5	24	Cage	Addition	Turf	4.42
5	24	Cage	Addition	Turf	2.31
5	24	Cage	Addition	Turf	10.82
5	24	Cage	Addition	Turf	2.25
5	24	Cage	Addition	Turf	4.16
5	24	Cage	Addition	Turf	4.81
5	24	Cage	Addition	Turf	2.58
5	24	Cage	Addition	Turf	8.37
5	24	Cage	Addition	Turf	11.72
5	24	Cage	Addition	Turf	2.08
5	24	Cage	Addition	Turf	4.66
5	24	Cage	Addition	Turf	5.51
5	24	Cage	Addition	Turf	3.31

5	24	Cage	Addition	Turf	5.27
5	24	Cage	Addition	Turf	8.13
5	24	Cage	Addition	Turf	1.77
5	24	Cage	Addition	Turf	6.20
5	24	Cage	Addition	Turf	2.37
5	25	Half	Natural	Turf	2.30
5	25	Half	Natural	Turf	2.32
5	25	Half	Natural	Turf	3.05
5	25	Half	Natural	Turf	3.55
5	25	Half	Natural	Turf	3.43
5	25	Half	Natural	Turf	3.13
5	25	Half	Natural	Turf	1.82
5	25	Half	Natural	Turf	4.37
5	25	Half	Natural	Turf	0.88
5	25	Half	Natural	Turf	3.59
5	25	Half	Natural	Turf	3.59
5	25	Half	Natural	Turf	4.44
5	25	Half	Natural	Turf	1.66
5	25	Half	Natural	Turf	4.13
5	25	Half	Natural	Turf	4.17
5	25	Half	Natural	Turf	3.30
5	25	Half	Natural	Turf	5.33
5	25	Half	Natural	Turf	1.70
5	25	Half	Natural	Turf	1.39
5	25	Half	Natural	Turf	6.43
5	26	Half	Natural	Turf	5.85
5	26	Half	Natural	Turf	2.68
5	26	Half	Natural	Turf	1.12
5	26	Half	Natural	Turf	2.30
5	26	Half	Natural	Turf	4.85
5	26	Half	Natural	Turf	6.12
5	26	Half	Natural	Turf	2.54
5	26	Half	Natural	Turf	3.57
5	26	Half	Natural	Turf	2.13
5	26	Half	Natural	Turf	2.77
5	26	Half	Natural	Turf	8.32
5	26	Half	Natural	Turf	2.46
5	26	Half	Natural	Turf	2.41
5	26	Half	Natural	Turf	4.61
5	26	Half	Natural	Turf	3.08
5	26	Half	Natural	Turf	1.45
5	26	Half	Natural	Turf	2.27
5	26	Half	Natural	Turf	2.79
5	26	Half	Natural	Turf	4.83
5	26	Half	Natural	Turf	2.94
5	27	Half	Natural	Turf	1.51
5	27	Half	Natural	Turf	1.79
5	27	Half	Natural	Turf	2.99
5	27	Half	Natural	Turf	2.26
5	27	Half	Natural	Turf	3.05
5	27	Half	Natural	Turf	2.85
5	27	Half	Natural	Turf	1.00
5	27	Half	Natural	Turf	1.76

5	27	Half	Natural	Turf	2.41
5	27	Half	Natural	Turf	2.16
5	27	Half	Natural	Turf	1.83
5	27	Half	Natural	Turf	1.61
5	27	Half	Natural	Turf	1.69
5	27	Half	Natural	Turf	1.98
5	27	Half	Natural	Turf	1.13
5	27	Half	Natural	Turf	2.28
5	27	Half	Natural	Turf	2.05
5	27	Half	Natural	Turf	3.25
5	27	Half	Natural	Turf	2.29
5	27	Half	Natural	Turf	2.52
5	28	Half	Addition	Turf	1.94
5	28	Half	Addition	Turf	5.64
5	28	Half	Addition	Turf	2.47
5	28	Half	Addition	Turf	2.56
5	28	Half	Addition	Turf	2.79
5	28	Half	Addition	Turf	3.21
5	28	Half	Addition	Turf	2.39
5	28	Half	Addition	Turf	1.17
5	28	Half	Addition	Turf	2.24
5	28	Half	Addition	Turf	3.28
5	28	Half	Addition	Turf	1.94
5	28	Half	Addition	Turf	2.18
5	28	Half	Addition	Turf	0.90
5	28	Half	Addition	Turf	2.34
5	28	Half	Addition	Turf	7.59
5	28	Half	Addition	Turf	2.43
5	28	Half	Addition	Turf	1.98
5	28	Half	Addition	Turf	4.27
5	28	Half	Addition	Turf	2.69
5	28	Half	Addition	Turf	2.78
5	29	Half	Addition	Turf	5.84
5	29	Half	Addition	Turf	3.93
5	29	Half	Addition	Turf	1.87
5	29	Half	Addition	Turf	6.13
5	29	Half	Addition	Turf	6.83
5	29	Half	Addition	Turf	3.63
5	29	Half	Addition	Turf	1.29
5	29	Half	Addition	Turf	4.08
5	29	Half	Addition	Turf	1.46
5	29	Half	Addition	Turf	2.74
5	29	Half	Addition	Turf	1.27
5	29	Half	Addition	Turf	1.88
5	29	Half	Addition	Turf	1.95
5	29	Half	Addition	Turf	2.95
5	29	Half	Addition	Turf	1.12
5	29	Half	Addition	Turf	1.85
5	29	Half	Addition	Turf	3.16
5	29	Half	Addition	Turf	3.48
5	29	Half	Addition	Turf	2.39
5	29	Half	Addition	Turf	7.07
5	30	Half	Addition	Turf	6.49

5	30	Half	Addition	Turf	6.97
5	30	Half	Addition	Turf	3.37
5	30	Half	Addition	Turf	1.83
5	30	Half	Addition	Turf	1.78
5	30	Half	Addition	Turf	3.90
5	30	Half	Addition	Turf	4.96
5	30	Half	Addition	Turf	3.24
5	30	Half	Addition	Turf	2.76
5	30	Half	Addition	Turf	5.31
5	30	Half	Addition	Turf	5.97
5	30	Half	Addition	Turf	2.95
5	30	Half	Addition	Turf	4.62
5	30	Half	Addition	Turf	1.07
5	30	Half	Addition	Turf	4.27
5	30	Half	Addition	Turf	8.79
5	30	Half	Addition	Turf	3.43
5	30	Half	Addition	Turf	1.18
5	30	Half	Addition	Turf	5.51
5	30	Half	Addition	Turf	4.41
5	31	Open	Natural	Turf	5.02
5	31	Open	Natural	Turf	3.70
5	31	Open	Natural	Turf	3.91
5	31	Open	Natural	Turf	1.77
5	31	Open	Natural	Turf	6.38
5	31	Open	Natural	Turf	1.58
5	31	Open	Natural	Turf	2.78
5	31	Open	Natural	Turf	6.49
5	31	Open	Natural	Turf	3.32
5	31	Open	Natural	Turf	2.26
5	31	Open	Natural	Turf	2.37
5	31	Open	Natural	Turf	5.10
5	31	Open	Natural	Turf	3.36
5	31	Open	Natural	Turf	4.95
5	31	Open	Natural	Turf	5.45
5	31	Open	Natural	Turf	5.00
5	31	Open	Natural	Turf	1.49
5	31	Open	Natural	Turf	5.51
5	31	Open	Natural	Turf	2.40
5	31	Open	Natural	Turf	1.91
5	32	Open	Natural	Turf	1.70
5	32	Open	Natural	Turf	1.77
5	32	Open	Natural	Turf	2.26
5	32	Open	Natural	Turf	2.59
5	32	Open	Natural	Turf	7.22
5	32	Open	Natural	Turf	2.75
5	32	Open	Natural	Turf	2.31
5	32	Open	Natural	Turf	7.51
5	32	Open	Natural	Turf	2.50
5	32	Open	Natural	Turf	3.13
5	32	Open	Natural	Turf	2.54
5	32	Open	Natural	Turf	2.60
5	32	Open	Natural	Turf	3.91
5	32	Open	Natural	Turf	4.39

5	32	Open	Natural	Turf	3.52
5	32	Open	Natural	Turf	2.44
5	32	Open	Natural	Turf	1.54
5	32	Open	Natural	Turf	2.72
5	32	Open	Natural	Turf	6.10
5	32	Open	Natural	Turf	3.00
5	33	Open	Natural	Turf	3.66
5	33	Open	Natural	Turf	2.03
5	33	Open	Natural	Turf	1.75
5	33	Open	Natural	Turf	2.04
5	33	Open	Natural	Turf	2.46
5	33	Open	Natural	Turf	9.47
5	33	Open	Natural	Turf	2.43
5	33	Open	Natural	Turf	2.36
5	33	Open	Natural	Turf	3.43
5	33	Open	Natural	Turf	1.94
5	33	Open	Natural	Turf	1.93
5	33	Open	Natural	Turf	1.65
5	33	Open	Natural	Turf	1.05
5	33	Open	Natural	Turf	1.94
5	33	Open	Natural	Turf	1.22
5	33	Open	Natural	Turf	1.93
5	33	Open	Natural	Turf	1.90
5	33	Open	Natural	Turf	3.09
5	33	Open	Natural	Turf	0.96
5	33	Open	Natural	Turf	3.68
5	34	Open	Addition	Turf	7.12
5	34	Open	Addition	Turf	7.47
5	34	Open	Addition	Turf	3.87
5	34	Open	Addition	Turf	3.87
5	34	Open	Addition	Turf	6.24
5	34	Open	Addition	Turf	1.43
5	34	Open	Addition	Turf	10.61
5	34	Open	Addition	Turf	4.30
5	34	Open	Addition	Turf	0.00
5	34	Open	Addition	Turf	6.32
5	34	Open	Addition	Turf	3.53
5	34	Open	Addition	Turf	3.43
5	34	Open	Addition	Turf	7.75
5	34	Open	Addition	Turf	3.52
5	34	Open	Addition	Turf	2.69
5	34	Open	Addition	Turf	7.04
5	34	Open	Addition	Turf	4.93
5	34	Open	Addition	Turf	2.97
5	34	Open	Addition	Turf	8.65
5	34	Open	Addition	Turf	11.21
5	35	Open	Addition	Turf	4.29
5	35	Open	Addition	Turf	0.00
5	35	Open	Addition	Turf	3.30
5	35	Open	Addition	Turf	2.12
5	35	Open	Addition	Turf	10.49
5	35	Open	Addition	Turf	1.81
5	35	Open	Addition	Turf	13.23

5	35	Open	Addition	Turf	3.01
5	35	Open	Addition	Turf	2.99
5	35	Open	Addition	Turf	3.68
5	35	Open	Addition	Turf	1.83
5	35	Open	Addition	Turf	2.59
5	35	Open	Addition	Turf	2.66
5	35	Open	Addition	Turf	1.70
5	35	Open	Addition	Turf	1.87
5	35	Open	Addition	Turf	3.10
5	35	Open	Addition	Turf	1.37
5	35	Open	Addition	Turf	3.88
5	35	Open	Addition	Turf	6.24
5	35	Open	Addition	Turf	9.10
5	36	Open	Addition	Turf	3.33
5	36	Open	Addition	Turf	2.77
5	36	Open	Addition	Turf	2.70
5	36	Open	Addition	Turf	0.60
5	36	Open	Addition	Turf	3.21
5	36	Open	Addition	Turf	7.93
5	36	Open	Addition	Turf	3.31
5	36	Open	Addition	Turf	3.11
5	36	Open	Addition	Turf	3.11
5	36	Open	Addition	Turf	3.50
5	36	Open	Addition	Turf	4.06
5	36	Open	Addition	Turf	2.78
5	36	Open	Addition	Turf	4.47
5	36	Open	Addition	Turf	2.44
5	36	Open	Addition	Turf	1.59
5	36	Open	Addition	Turf	2.39
5	36	Open	Addition	Turf	3.20
5	36	Open	Addition	Turf	1.79
5	36	Open	Addition	Turf	6.48
5	36	Open	Addition	Turf	5.17
6	1	Full	Natural	Turf	1.73
6	1	Full	Natural	Turf	1.64
6	1	Full	Natural	Turf	3.54
6	1	Full	Natural	Turf	2.73
6	1	Full	Natural	Turf	2.41
6	1	Full	Natural	Turf	9.94
6	1	Full	Natural	Turf	1.33
6	1	Full	Natural	Turf	2.66
6	1	Full	Natural	Turf	6.32
6	1	Full	Natural	Turf	3.11
6	1	Full	Natural	Turf	17.74
6	1	Full	Natural	Turf	3.25
6	1	Full	Natural	Turf	3.85
6	1	Full	Natural	Turf	1.63
6	1	Full	Natural	Turf	1.54
6	1	Full	Natural	Turf	6.20
6	1	Full	Natural	Turf	1.88
6	1	Full	Natural	Turf	4.94
6	1	Full	Natural	Turf	1.27
6	1	Full	Natural	Turf	2.86

6	2	Full	Natural	Turf	2.28
6	2	Full	Natural	Turf	1.52
6	2	Full	Natural	Turf	2.57
6	2	Full	Natural	Turf	9.21
6	2	Full	Natural	Turf	2.77
6	2	Full	Natural	Turf	2.21
6	2	Full	Natural	Turf	2.39
6	2	Full	Natural	Turf	3.36
6	2	Full	Natural	Turf	4.02
6	2	Full	Natural	Turf	2.37
6	2	Full	Natural	Turf	3.21
6	2	Full	Natural	Turf	3.61
6	2	Full	Natural	Turf	3.51
6	2	Full	Natural	Turf	2.16
6	2	Full	Natural	Turf	5.54
6	2	Full	Natural	Turf	4.40
6	2	Full	Natural	Turf	3.23
6	2	Full	Natural	Turf	3.48
6	2	Full	Natural	Turf	2.74
6	2	Full	Natural	Turf	3.08
6	3	Full	Natural	Turf	3.32
6	3	Full	Natural	Turf	2.64
6	3	Full	Natural	Turf	1.99
6	3	Full	Natural	Turf	2.54
6	3	Full	Natural	Turf	3.11
6	3	Full	Natural	Turf	1.25
6	3	Full	Natural	Turf	2.45
6	3	Full	Natural	Turf	4.40
6	3	Full	Natural	Turf	4.13
6	3	Full	Natural	Turf	3.04
6	3	Full	Natural	Turf	5.22
6	3	Full	Natural	Turf	3.19
6	3	Full	Natural	Turf	3.02
6	3	Full	Natural	Turf	5.08
6	3	Full	Natural	Turf	2.02
6	3	Full	Natural	Turf	4.47
6	3	Full	Natural	Turf	3.26
6	3	Full	Natural	Turf	2.95
6	3	Full	Natural	Turf	2.55
6	3	Full	Natural	Turf	1.98
6	4	Full	Addition	Turf	2.42
6	4	Full	Addition	Turf	3.25
6	4	Full	Addition	Turf	4.25
6	4	Full	Addition	Turf	3.45
6	4	Full	Addition	Turf	3.23
6	4	Full	Addition	Turf	11.80
6	4	Full	Addition	Turf	1.80
6	4	Full	Addition	Turf	3.55
6	4	Full	Addition	Turf	3.83
6	4	Full	Addition	Turf	4.34
6	4	Full	Addition	Turf	10.57
6	4	Full	Addition	Turf	4.07
6	4	Full	Addition	Turf	3.68

6	4 Full	Addition	Turf	4.77
6	4 Full	Addition	Turf	4.88
6	4 Full	Addition	Turf	2.16
6	4 Full	Addition	Turf	2.78
6	4 Full	Addition	Turf	4.14
6	4 Full	Addition	Turf	5.61
6	4 Full	Addition	Turf	4.53
6	5 Full	Addition	Turf	5.45
6	5 Full	Addition	Turf	3.17
6	5 Full	Addition	Turf	4.22
6	5 Full	Addition	Turf	5.64
6	5 Full	Addition	Turf	3.38
6	5 Full	Addition	Turf	1.58
6	5 Full	Addition	Turf	6.33
6	5 Full	Addition	Turf	5.00
6	5 Full	Addition	Turf	3.69
6	5 Full	Addition	Turf	4.33
6	5 Full	Addition	Turf	5.58
6	5 Full	Addition	Turf	6.20
6	5 Full	Addition	Turf	2.38
6	5 Full	Addition	Turf	1.12
6	5 Full	Addition	Turf	1.91
6	5 Full	Addition	Turf	7.22
6	5 Full	Addition	Turf	2.53
6	5 Full	Addition	Turf	3.24
6	5 Full	Addition	Turf	2.86
6	5 Full	Addition	Turf	4.35
6	6 Full	Addition	Turf	2.09
6	6 Full	Addition	Turf	3.18
6	6 Full	Addition	Turf	1.89
6	6 Full	Addition	Turf	2.19
6	6 Full	Addition	Turf	2.15
6	6 Full	Addition	Turf	0.00
6	6 Full	Addition	Turf	3.00
6	6 Full	Addition	Turf	2.46
6	6 Full	Addition	Turf	5.65
6	6 Full	Addition	Turf	4.45
6	6 Full	Addition	Turf	2.29
6	6 Full	Addition	Turf	4.34
6	6 Full	Addition	Turf	6.99
6	6 Full	Addition	Turf	5.43
6	6 Full	Addition	Turf	2.79
6	6 Full	Addition	Turf	7.88
6	6 Full	Addition	Turf	7.54
6	6 Full	Addition	Turf	4.51
6	6 Full	Addition	Turf	2.86
6	6 Full	Addition	Turf	0.00
6	7 Half	Natural	Turf	3.65
6	7 Half	Natural	Turf	1.77
6	7 Half	Natural	Turf	4.96
6	7 Half	Natural	Turf	2.26
6	7 Half	Natural	Turf	3.60
6	7 Half	Natural	Turf	2.91

6	7	Half	Natural	Turf	4.44
6	7	Half	Natural	Turf	9.20
6	7	Half	Natural	Turf	4.89
6	7	Half	Natural	Turf	7.69
6	7	Half	Natural	Turf	1.96
6	7	Half	Natural	Turf	2.23
6	7	Half	Natural	Turf	2.88
6	7	Half	Natural	Turf	2.00
6	7	Half	Natural	Turf	3.20
6	7	Half	Natural	Turf	2.53
6	7	Half	Natural	Turf	1.97
6	7	Half	Natural	Turf	2.19
6	7	Half	Natural	Turf	3.76
6	7	Half	Natural	Turf	4.33
6	8	Half	Natural	Turf	1.66
6	8	Half	Natural	Turf	1.85
6	8	Half	Natural	Turf	2.03
6	8	Half	Natural	Turf	3.33
6	8	Half	Natural	Turf	2.45
6	8	Half	Natural	Turf	3.65
6	8	Half	Natural	Turf	3.03
6	8	Half	Natural	Turf	2.63
6	8	Half	Natural	Turf	2.18
6	8	Half	Natural	Turf	2.43
6	8	Half	Natural	Turf	3.69
6	8	Half	Natural	Turf	1.30
6	8	Half	Natural	Turf	1.86
6	8	Half	Natural	Turf	2.75
6	8	Half	Natural	Turf	2.94
6	8	Half	Natural	Turf	2.98
6	8	Half	Natural	Turf	2.76
6	8	Half	Natural	Turf	2.41
6	8	Half	Natural	Turf	2.70
6	8	Half	Natural	Turf	1.79
6	9	Half	Natural	Turf	7.91
6	9	Half	Natural	Turf	3.27
6	9	Half	Natural	Turf	8.41
6	9	Half	Natural	Turf	4.26
6	9	Half	Natural	Turf	2.92
6	9	Half	Natural	Turf	3.77
6	9	Half	Natural	Turf	1.80
6	9	Half	Natural	Turf	3.80
6	9	Half	Natural	Turf	3.71
6	9	Half	Natural	Turf	3.93
6	9	Half	Natural	Turf	1.95
6	9	Half	Natural	Turf	2.17
6	9	Half	Natural	Turf	4.80
6	9	Half	Natural	Turf	1.96
6	9	Half	Natural	Turf	0.49
6	9	Half	Natural	Turf	3.71
6	9	Half	Natural	Turf	0.00
6	9	Half	Natural	Turf	2.41
6	9	Half	Natural	Turf	2.30

6	9	Half	Natural	Turf	1.42
6	10	Half	Addition	Turf	1.59
6	10	Half	Addition	Turf	1.82
6	10	Half	Addition	Turf	2.78
6	10	Half	Addition	Turf	1.84
6	10	Half	Addition	Turf	2.45
6	10	Half	Addition	Turf	2.80
6	10	Half	Addition	Turf	2.31
6	10	Half	Addition	Turf	3.39
6	10	Half	Addition	Turf	1.55
6	10	Half	Addition	Turf	2.89
6	10	Half	Addition	Turf	3.89
6	10	Half	Addition	Turf	4.92
6	10	Half	Addition	Turf	2.25
6	10	Half	Addition	Turf	2.46
6	10	Half	Addition	Turf	2.23
6	10	Half	Addition	Turf	2.25
6	10	Half	Addition	Turf	2.45
6	10	Half	Addition	Turf	3.42
6	10	Half	Addition	Turf	2.77
6	10	Half	Addition	Turf	3.01
6	11	Half	Addition	Turf	4.84
6	11	Half	Addition	Turf	2.59
6	11	Half	Addition	Turf	3.34
6	11	Half	Addition	Turf	3.38
6	11	Half	Addition	Turf	2.72
6	11	Half	Addition	Turf	4.56
6	11	Half	Addition	Turf	4.91
6	11	Half	Addition	Turf	1.80
6	11	Half	Addition	Turf	3.70
6	11	Half	Addition	Turf	3.52
6	11	Half	Addition	Turf	4.83
6	11	Half	Addition	Turf	3.71
6	11	Half	Addition	Turf	4.63
6	11	Half	Addition	Turf	2.12
6	11	Half	Addition	Turf	2.64
6	11	Half	Addition	Turf	1.97
6	11	Half	Addition	Turf	3.60
6	11	Half	Addition	Turf	5.01
6	11	Half	Addition	Turf	2.53
6	11	Half	Addition	Turf	3.17
6	12	Half	Addition	Turf	1.69
6	12	Half	Addition	Turf	2.15
6	12	Half	Addition	Turf	4.60
6	12	Half	Addition	Turf	3.65
6	12	Half	Addition	Turf	2.83
6	12	Half	Addition	Turf	1.73
6	12	Half	Addition	Turf	4.03
6	12	Half	Addition	Turf	3.76
6	12	Half	Addition	Turf	4.84
6	12	Half	Addition	Turf	2.91
6	12	Half	Addition	Turf	2.25
6	12	Half	Addition	Turf	2.61

6	12	Half	Addition	Turf	1.74
6	12	Half	Addition	Turf	2.40
6	12	Half	Addition	Turf	5.01
6	12	Half	Addition	Turf	2.86
6	12	Half	Addition	Turf	3.22
6	12	Half	Addition	Turf	2.27
6	12	Half	Addition	Turf	3.17
6	12	Half	Addition	Turf	7.72
6	13	Open	Natural	Turf	3.71
6	13	Open	Natural	Turf	1.43
6	13	Open	Natural	Turf	1.66
6	13	Open	Natural	Turf	3.69
6	13	Open	Natural	Turf	4.01
6	13	Open	Natural	Turf	3.15
6	13	Open	Natural	Turf	1.72
6	13	Open	Natural	Turf	2.73
6	13	Open	Natural	Turf	1.67
6	13	Open	Natural	Turf	2.33
6	13	Open	Natural	Turf	3.16
6	13	Open	Natural	Turf	3.34
6	13	Open	Natural	Turf	2.70
6	13	Open	Natural	Turf	2.13
6	13	Open	Natural	Turf	2.08
6	13	Open	Natural	Turf	2.88
6	13	Open	Natural	Turf	4.36
6	13	Open	Natural	Turf	2.40
6	13	Open	Natural	Turf	3.05
6	13	Open	Natural	Turf	6.81
6	14	Open	Natural	Turf	3.12
6	14	Open	Natural	Turf	1.72
6	14	Open	Natural	Turf	1.75
6	14	Open	Natural	Turf	2.60
6	14	Open	Natural	Turf	3.68
6	14	Open	Natural	Turf	3.72
6	14	Open	Natural	Turf	2.11
6	14	Open	Natural	Turf	2.30
6	14	Open	Natural	Turf	1.23
6	14	Open	Natural	Turf	4.32
6	14	Open	Natural	Turf	2.63
6	14	Open	Natural	Turf	1.81
6	14	Open	Natural	Turf	2.40
6	14	Open	Natural	Turf	2.74
6	14	Open	Natural	Turf	2.21
6	14	Open	Natural	Turf	4.36
6	14	Open	Natural	Turf	1.77
6	14	Open	Natural	Turf	2.85
6	14	Open	Natural	Turf	3.12
6	14	Open	Natural	Turf	1.48
6	15	Open	Natural	Turf	3.24
6	15	Open	Natural	Turf	1.45
6	15	Open	Natural	Turf	4.44
6	15	Open	Natural	Turf	2.47
6	15	Open	Natural	Turf	2.67

6	15	Open	Natural	Turf	1.56
6	15	Open	Natural	Turf	1.81
6	15	Open	Natural	Turf	4.09
6	15	Open	Natural	Turf	2.76
6	15	Open	Natural	Turf	1.97
6	15	Open	Natural	Turf	1.89
6	15	Open	Natural	Turf	2.72
6	15	Open	Natural	Turf	2.43
6	15	Open	Natural	Turf	2.97
6	15	Open	Natural	Turf	4.41
6	15	Open	Natural	Turf	5.00
6	15	Open	Natural	Turf	3.06
6	15	Open	Natural	Turf	3.06
6	15	Open	Natural	Turf	3.28
6	15	Open	Natural	Turf	1.96
6	16	Open	Addition	Turf	2.51
6	16	Open	Addition	Turf	1.96
6	16	Open	Addition	Turf	2.21
6	16	Open	Addition	Turf	3.04
6	16	Open	Addition	Turf	3.23
6	16	Open	Addition	Turf	2.28
6	16	Open	Addition	Turf	3.95
6	16	Open	Addition	Turf	13.86
6	16	Open	Addition	Turf	4.29
6	16	Open	Addition	Turf	1.81
6	16	Open	Addition	Turf	2.40
6	16	Open	Addition	Turf	1.81
6	16	Open	Addition	Turf	2.57
6	16	Open	Addition	Turf	2.42
6	16	Open	Addition	Turf	3.45
6	16	Open	Addition	Turf	2.97
6	16	Open	Addition	Turf	1.61
6	16	Open	Addition	Turf	3.72
6	16	Open	Addition	Turf	2.04
6	16	Open	Addition	Turf	1.93
6	17	Open	Addition	Turf	9.21
6	17	Open	Addition	Turf	4.92
6	17	Open	Addition	Turf	2.58
6	17	Open	Addition	Turf	6.03
6	17	Open	Addition	Turf	5.47
6	17	Open	Addition	Turf	6.44
6	17	Open	Addition	Turf	4.93
6	17	Open	Addition	Turf	2.48
6	17	Open	Addition	Turf	3.02
6	17	Open	Addition	Turf	4.52
6	17	Open	Addition	Turf	5.00
6	17	Open	Addition	Turf	2.92
6	17	Open	Addition	Turf	4.59
6	17	Open	Addition	Turf	4.47
6	17	Open	Addition	Turf	4.67
6	17	Open	Addition	Turf	1.96
6	17	Open	Addition	Turf	2.78
6	17	Open	Addition	Turf	6.69

6	17 Open	Addition	Turf	2.40
6	17 Open	Addition	Turf	4.40
6	18 Open	Addition	Turf	2.54
6	18 Open	Addition	Turf	5.45
6	18 Open	Addition	Turf	3.03
6	18 Open	Addition	Turf	2.19
6	18 Open	Addition	Turf	3.80
6	18 Open	Addition	Turf	2.83
6	18 Open	Addition	Turf	4.06
6	18 Open	Addition	Turf	2.16
6	18 Open	Addition	Turf	2.63
6	18 Open	Addition	Turf	4.08
6	18 Open	Addition	Turf	4.06
6	18 Open	Addition	Turf	3.45
6	18 Open	Addition	Turf	2.40
6	18 Open	Addition	Turf	2.59
6	18 Open	Addition	Turf	2.62
6	18 Open	Addition	Turf	3.10
6	18 Open	Addition	Turf	4.78
6	18 Open	Addition	Turf	1.21
6	18 Open	Addition	Turf	4.08
6	18 Open	Addition	Turf	8.14
6	19 Cage	Natural	Turf	6.34
6	19 Cage	Natural	Turf	5.50
6	19 Cage	Natural	Turf	7.28
6	19 Cage	Natural	Turf	6.47
6	19 Cage	Natural	Turf	8.71
6	19 Cage	Natural	Turf	4.48
6	19 Cage	Natural	Turf	3.01
6	19 Cage	Natural	Turf	4.94
6	19 Cage	Natural	Turf	5.72
6	19 Cage	Natural	Turf	5.61
6	19 Cage	Natural	Turf	5.05
6	19 Cage	Natural	Turf	11.64
6	19 Cage	Natural	Turf	3.95
6	19 Cage	Natural	Turf	5.53
6	19 Cage	Natural	Turf	3.72
6	19 Cage	Natural	Turf	4.05
6	19 Cage	Natural	Turf	7.31
6	19 Cage	Natural	Turf	5.26
6	19 Cage	Natural	Turf	6.24
6	19 Cage	Natural	Turf	8.30
6	20 Cage	Natural	Turf	5.67
6	20 Cage	Natural	Turf	3.01
6	20 Cage	Natural	Turf	2.47
6	20 Cage	Natural	Turf	3.58
6	20 Cage	Natural	Turf	6.43
6	20 Cage	Natural	Turf	5.23
6	20 Cage	Natural	Turf	6.22
6	20 Cage	Natural	Turf	3.62
6	20 Cage	Natural	Turf	6.03
6	20 Cage	Natural	Turf	11.88
6	20 Cage	Natural	Turf	12.53

6	20	Cage	Natural	Turf	6.58
6	20	Cage	Natural	Turf	4.11
6	20	Cage	Natural	Turf	2.71
6	20	Cage	Natural	Turf	3.90
6	20	Cage	Natural	Turf	3.71
6	20	Cage	Natural	Turf	5.87
6	20	Cage	Natural	Turf	6.45
6	20	Cage	Natural	Turf	3.46
6	20	Cage	Natural	Turf	2.06
6	21	Cage	Natural	Turf	4.88
6	21	Cage	Natural	Turf	1.94
6	21	Cage	Natural	Turf	5.88
6	21	Cage	Natural	Turf	9.39
6	21	Cage	Natural	Turf	8.26
6	21	Cage	Natural	Turf	3.70
6	21	Cage	Natural	Turf	1.17
6	21	Cage	Natural	Turf	7.04
6	21	Cage	Natural	Turf	2.95
6	21	Cage	Natural	Turf	2.42
6	21	Cage	Natural	Turf	13.37
6	21	Cage	Natural	Turf	3.39
6	21	Cage	Natural	Turf	3.40
6	21	Cage	Natural	Turf	4.96
6	21	Cage	Natural	Turf	2.21
6	21	Cage	Natural	Turf	6.48
6	21	Cage	Natural	Turf	2.74
6	21	Cage	Natural	Turf	5.64
6	21	Cage	Natural	Turf	10.04
6	21	Cage	Natural	Turf	3.15
6	22	Cage	Addition	Turf	12.25
6	22	Cage	Addition	Turf	4.29
6	22	Cage	Addition	Turf	7.63
6	22	Cage	Addition	Turf	5.95
6	22	Cage	Addition	Turf	5.38
6	22	Cage	Addition	Turf	3.62
6	22	Cage	Addition	Turf	4.13
6	22	Cage	Addition	Turf	4.56
6	22	Cage	Addition	Turf	5.74
6	22	Cage	Addition	Turf	3.92
6	22	Cage	Addition	Turf	10.60
6	22	Cage	Addition	Turf	6.54
6	22	Cage	Addition	Turf	2.96
6	22	Cage	Addition	Turf	4.61
6	22	Cage	Addition	Turf	4.34
6	22	Cage	Addition	Turf	5.90
6	22	Cage	Addition	Turf	5.86
6	22	Cage	Addition	Turf	4.91
6	22	Cage	Addition	Turf	5.96
6	22	Cage	Addition	Turf	5.95
6	23	Cage	Addition	Turf	7.41
6	23	Cage	Addition	Turf	3.01
6	23	Cage	Addition	Turf	3.89
6	23	Cage	Addition	Turf	2.51

6	23	Cage	Addition	Turf	5.63
6	23	Cage	Addition	Turf	2.70
6	23	Cage	Addition	Turf	3.15
6	23	Cage	Addition	Turf	2.63
6	23	Cage	Addition	Turf	14.16
6	23	Cage	Addition	Turf	8.63
6	23	Cage	Addition	Turf	4.96
6	23	Cage	Addition	Turf	6.79
6	23	Cage	Addition	Turf	4.88
6	23	Cage	Addition	Turf	6.92
6	23	Cage	Addition	Turf	2.79
6	23	Cage	Addition	Turf	2.93
6	23	Cage	Addition	Turf	2.85
6	23	Cage	Addition	Turf	2.03
6	23	Cage	Addition	Turf	3.62
6	23	Cage	Addition	Turf	5.18
6	24	Cage	Addition	Turf	15.78
6	24	Cage	Addition	Turf	10.13
6	24	Cage	Addition	Turf	18.06
6	24	Cage	Addition	Turf	7.24
6	24	Cage	Addition	Turf	5.43
6	24	Cage	Addition	Turf	8.23
6	24	Cage	Addition	Turf	7.80
6	24	Cage	Addition	Turf	10.84
6	24	Cage	Addition	Turf	3.57
6	24	Cage	Addition	Turf	4.23
6	24	Cage	Addition	Turf	5.33
6	24	Cage	Addition	Turf	10.10
6	24	Cage	Addition	Turf	3.34
6	24	Cage	Addition	Turf	3.00
6	24	Cage	Addition	Turf	4.54
6	24	Cage	Addition	Turf	6.28
6	24	Cage	Addition	Turf	2.80
6	24	Cage	Addition	Turf	5.97
6	24	Cage	Addition	Turf	3.58
6	24	Cage	Addition	Turf	4.52
6	25	Half	Natural	Turf	3.02
6	25	Half	Natural	Turf	7.79
6	25	Half	Natural	Turf	1.82
6	25	Half	Natural	Turf	5.08
6	25	Half	Natural	Turf	8.74
6	25	Half	Natural	Turf	2.18
6	25	Half	Natural	Turf	2.78
6	25	Half	Natural	Turf	2.86
6	25	Half	Natural	Turf	8.60
6	25	Half	Natural	Turf	4.47
6	25	Half	Natural	Turf	4.64
6	25	Half	Natural	Turf	5.65
6	25	Half	Natural	Turf	4.84
6	25	Half	Natural	Turf	3.22
6	25	Half	Natural	Turf	3.11
6	25	Half	Natural	Turf	1.97
6	25	Half	Natural	Turf	4.72

6	25	Half	Natural	Turf	5.31
6	25	Half	Natural	Turf	6.11
6	25	Half	Natural	Turf	30.05
6	26	Half	Natural	Turf	2.64
6	26	Half	Natural	Turf	3.47
6	26	Half	Natural	Turf	2.22
6	26	Half	Natural	Turf	2.32
6	26	Half	Natural	Turf	2.89
6	26	Half	Natural	Turf	2.66
6	26	Half	Natural	Turf	6.86
6	26	Half	Natural	Turf	4.65
6	26	Half	Natural	Turf	2.46
6	26	Half	Natural	Turf	3.06
6	26	Half	Natural	Turf	6.54
6	26	Half	Natural	Turf	11.15
6	26	Half	Natural	Turf	12.04
6	26	Half	Natural	Turf	1.40
6	26	Half	Natural	Turf	3.92
6	26	Half	Natural	Turf	3.42
6	26	Half	Natural	Turf	2.50
6	26	Half	Natural	Turf	1.72
6	26	Half	Natural	Turf	4.39
6	26	Half	Natural	Turf	3.97
6	27	Half	Natural	Turf	3.04
6	27	Half	Natural	Turf	3.05
6	27	Half	Natural	Turf	0.94
6	27	Half	Natural	Turf	1.91
6	27	Half	Natural	Turf	3.07
6	27	Half	Natural	Turf	1.67
6	27	Half	Natural	Turf	1.42
6	27	Half	Natural	Turf	2.37
6	27	Half	Natural	Turf	2.82
6	27	Half	Natural	Turf	3.91
6	27	Half	Natural	Turf	2.31
6	27	Half	Natural	Turf	1.23
6	27	Half	Natural	Turf	5.09
6	27	Half	Natural	Turf	1.64
6	27	Half	Natural	Turf	3.05
6	27	Half	Natural	Turf	4.99
6	27	Half	Natural	Turf	2.46
6	27	Half	Natural	Turf	2.44
6	27	Half	Natural	Turf	3.67
6	27	Half	Natural	Turf	4.29
6	28	Half	Addition	Turf	2.20
6	28	Half	Addition	Turf	3.30
6	28	Half	Addition	Turf	1.72
6	28	Half	Addition	Turf	3.65
6	28	Half	Addition	Turf	2.41
6	28	Half	Addition	Turf	3.16
6	28	Half	Addition	Turf	2.55
6	28	Half	Addition	Turf	4.22
6	28	Half	Addition	Turf	4.38
6	28	Half	Addition	Turf	2.45

6	28	Half	Addition	Turf	1.51
6	28	Half	Addition	Turf	2.59
6	28	Half	Addition	Turf	4.30
6	28	Half	Addition	Turf	1.93
6	28	Half	Addition	Turf	2.28
6	28	Half	Addition	Turf	3.29
6	28	Half	Addition	Turf	2.99
6	28	Half	Addition	Turf	1.81
6	28	Half	Addition	Turf	3.16
6	28	Half	Addition	Turf	3.60
6	29	Half	Addition	Turf	1.76
6	29	Half	Addition	Turf	3.29
6	29	Half	Addition	Turf	2.38
6	29	Half	Addition	Turf	6.63
6	29	Half	Addition	Turf	2.55
6	29	Half	Addition	Turf	1.35
6	29	Half	Addition	Turf	2.31
6	29	Half	Addition	Turf	2.55
6	29	Half	Addition	Turf	2.26
6	29	Half	Addition	Turf	2.53
6	29	Half	Addition	Turf	1.71
6	29	Half	Addition	Turf	2.84
6	29	Half	Addition	Turf	2.37
6	29	Half	Addition	Turf	2.59
6	29	Half	Addition	Turf	3.13
6	29	Half	Addition	Turf	1.91
6	29	Half	Addition	Turf	7.30
6	29	Half	Addition	Turf	4.84
6	29	Half	Addition	Turf	2.93
6	29	Half	Addition	Turf	2.75
6	30	Half	Addition	Turf	2.39
6	30	Half	Addition	Turf	5.13
6	30	Half	Addition	Turf	4.45
6	30	Half	Addition	Turf	6.96
6	30	Half	Addition	Turf	5.59
6	30	Half	Addition	Turf	3.39
6	30	Half	Addition	Turf	4.08
6	30	Half	Addition	Turf	5.23
6	30	Half	Addition	Turf	2.58
6	30	Half	Addition	Turf	3.79
6	30	Half	Addition	Turf	15.96
6	30	Half	Addition	Turf	3.60
6	30	Half	Addition	Turf	6.37
6	30	Half	Addition	Turf	6.12
6	30	Half	Addition	Turf	6.88
6	30	Half	Addition	Turf	4.07
6	30	Half	Addition	Turf	10.12
6	30	Half	Addition	Turf	8.51
6	30	Half	Addition	Turf	3.38
6	30	Half	Addition	Turf	4.59
6	31	Open	Natural	Turf	2.34
6	31	Open	Natural	Turf	2.95
6	31	Open	Natural	Turf	3.61

6	31	Open	Natural	Turf	4.24
6	31	Open	Natural	Turf	4.78
6	31	Open	Natural	Turf	2.43
6	31	Open	Natural	Turf	4.03
6	31	Open	Natural	Turf	0.00
6	31	Open	Natural	Turf	3.08
6	31	Open	Natural	Turf	4.08
6	31	Open	Natural	Turf	3.16
6	31	Open	Natural	Turf	4.14
6	31	Open	Natural	Turf	4.36
6	31	Open	Natural	Turf	2.50
6	31	Open	Natural	Turf	1.93
6	31	Open	Natural	Turf	2.98
6	31	Open	Natural	Turf	3.63
6	31	Open	Natural	Turf	3.09
6	31	Open	Natural	Turf	5.25
6	31	Open	Natural	Turf	2.52
6	32	Open	Natural	Turf	4.07
6	32	Open	Natural	Turf	2.05
6	32	Open	Natural	Turf	1.80
6	32	Open	Natural	Turf	4.43
6	32	Open	Natural	Turf	1.74
6	32	Open	Natural	Turf	3.05
6	32	Open	Natural	Turf	2.02
6	32	Open	Natural	Turf	1.89
6	32	Open	Natural	Turf	2.47
6	32	Open	Natural	Turf	1.38
6	32	Open	Natural	Turf	2.13
6	32	Open	Natural	Turf	1.34
6	32	Open	Natural	Turf	3.57
6	32	Open	Natural	Turf	1.48
6	32	Open	Natural	Turf	1.72
6	32	Open	Natural	Turf	2.16
6	32	Open	Natural	Turf	2.67
6	32	Open	Natural	Turf	4.97
6	32	Open	Natural	Turf	2.22
6	32	Open	Natural	Turf	3.05
6	33	Open	Natural	Turf	1.76
6	33	Open	Natural	Turf	2.06
6	33	Open	Natural	Turf	1.19
6	33	Open	Natural	Turf	3.47
6	33	Open	Natural	Turf	1.97
6	33	Open	Natural	Turf	2.48
6	33	Open	Natural	Turf	2.68
6	33	Open	Natural	Turf	1.88
6	33	Open	Natural	Turf	2.74
6	33	Open	Natural	Turf	4.01
6	33	Open	Natural	Turf	2.65
6	33	Open	Natural	Turf	2.91
6	33	Open	Natural	Turf	1.09
6	33	Open	Natural	Turf	2.04
6	33	Open	Natural	Turf	2.58
6	33	Open	Natural	Turf	1.87

6	33	Open	Natural	Turf	1.36
6	33	Open	Natural	Turf	1.67
6	33	Open	Natural	Turf	2.57
6	33	Open	Natural	Turf	3.04
6	34	Open	Addition	Turf	2.47
6	34	Open	Addition	Turf	3.66
6	34	Open	Addition	Turf	1.72
6	34	Open	Addition	Turf	4.97
6	34	Open	Addition	Turf	4.91
6	34	Open	Addition	Turf	1.98
6	34	Open	Addition	Turf	1.79
6	34	Open	Addition	Turf	13.70
6	34	Open	Addition	Turf	5.93
6	34	Open	Addition	Turf	2.44
6	34	Open	Addition	Turf	2.67
6	34	Open	Addition	Turf	12.37
6	34	Open	Addition	Turf	6.49
6	34	Open	Addition	Turf	13.83
6	34	Open	Addition	Turf	9.53
6	34	Open	Addition	Turf	6.73
6	34	Open	Addition	Turf	10.89
6	34	Open	Addition	Turf	5.02
6	34	Open	Addition	Turf	11.57
6	34	Open	Addition	Turf	11.58
6	35	Open	Addition	Turf	5.53
6	35	Open	Addition	Turf	10.95
6	35	Open	Addition	Turf	2.41
6	35	Open	Addition	Turf	7.00
6	35	Open	Addition	Turf	7.98
6	35	Open	Addition	Turf	2.74
6	35	Open	Addition	Turf	4.75
6	35	Open	Addition	Turf	2.82
6	35	Open	Addition	Turf	3.64
6	35	Open	Addition	Turf	17.70
6	35	Open	Addition	Turf	3.50
6	35	Open	Addition	Turf	3.81
6	35	Open	Addition	Turf	3.36
6	35	Open	Addition	Turf	4.40
6	35	Open	Addition	Turf	2.95
6	35	Open	Addition	Turf	2.67
6	35	Open	Addition	Turf	7.51
6	35	Open	Addition	Turf	5.62
6	35	Open	Addition	Turf	3.36
6	35	Open	Addition	Turf	5.50
6	36	Open	Addition	Turf	3.28
6	36	Open	Addition	Turf	3.74
6	36	Open	Addition	Turf	3.68
6	36	Open	Addition	Turf	3.09
6	36	Open	Addition	Turf	2.80
6	36	Open	Addition	Turf	5.72
6	36	Open	Addition	Turf	2.65
6	36	Open	Addition	Turf	2.49
6	36	Open	Addition	Turf	3.54

6	36	Open	Addition	Turf	1.83
6	36	Open	Addition	Turf	3.20
6	36	Open	Addition	Turf	2.81
6	36	Open	Addition	Turf	2.86
6	36	Open	Addition	Turf	2.01
6	36	Open	Addition	Turf	1.47
6	36	Open	Addition	Turf	1.93
6	36	Open	Addition	Turf	2.69
6	36	Open	Addition	Turf	3.85
6	36	Open	Addition	Turf	2.59
6	36	Open	Addition	Turf	1.98
7	27	Half	Natural	Turf	6.85
7	27	Half	Natural	Turf	2.77
7	27	Half	Natural	Turf	2.14
7	27	Half	Natural	Turf	8.59
7	27	Half	Natural	Turf	3.78
7	27	Half	Natural	Turf	3.71
7	27	Half	Natural	Turf	13.16
7	27	Half	Natural	Turf	3.71
7	27	Half	Natural	Turf	2.23
7	27	Half	Natural	Turf	1.52
7	27	Half	Natural	Turf	3.18
7	27	Half	Natural	Turf	3.03
7	27	Half	Natural	Turf	2.29
7	27	Half	Natural	Turf	2.12
7	27	Half	Natural	Turf	1.57
7	27	Half	Natural	Turf	2.69
7	27	Half	Natural	Turf	2.67
7	27	Half	Natural	Turf	2.04
7	27	Half	Natural	Turf	1.96
7	27	Half	Natural	Turf	2.85
7	27	Half	Natural	Sed	1.10
7	27	Half	Natural	Sed	3.74
7	27	Half	Natural	Sed	2.40
7	27	Half	Natural	Sed	2.25
7	27	Half	Natural	Sed	2.71
7	27	Half	Natural	Sed	4.03
7	27	Half	Natural	Sed	10.39
7	27	Half	Natural	Sed	0.75
7	27	Half	Natural	Sed	4.07
7	27	Half	Natural	Sed	1.82
7	27	Half	Natural	Sed	1.09
7	27	Half	Natural	Sed	2.76
7	27	Half	Natural	Sed	10.80
7	27	Half	Natural	Sed	3.18
7	27	Half	Natural	Sed	0.84
7	27	Half	Natural	Sed	0.56
7	27	Half	Natural	Sed	5.20
7	27	Half	Natural	Sed	2.94
7	27	Half	Natural	Sed	1.18
7	27	Half	Natural	Sed	0.87
7	23	Cage	Addition	Turf	17.86
7	23	Cage	Addition	Turf	11.65

7	23	Cage	Addition	Turf	3.93
7	23	Cage	Addition	Turf	0.87
7	23	Cage	Addition	Turf	5.72
7	23	Cage	Addition	Turf	5.82
7	23	Cage	Addition	Turf	4.34
7	23	Cage	Addition	Turf	0.00
7	23	Cage	Addition	Turf	11.50
7	23	Cage	Addition	Turf	11.77
7	23	Cage	Addition	Turf	14.12
7	23	Cage	Addition	Turf	5.70
7	23	Cage	Addition	Turf	5.41
7	23	Cage	Addition	Turf	3.05
7	23	Cage	Addition	Turf	2.42
7	23	Cage	Addition	Turf	3.71
7	23	Cage	Addition	Turf	2.13
7	23	Cage	Addition	Turf	6.02
7	23	Cage	Addition	Turf	8.45
7	23	Cage	Addition	Turf	13.32
7	23	Cage	Addition	Sed	12.32
7	23	Cage	Addition	Sed	5.82
7	23	Cage	Addition	Sed	4.08
7	23	Cage	Addition	Sed	3.56
7	23	Cage	Addition	Sed	4.01
7	23	Cage	Addition	Sed	3.98
7	23	Cage	Addition	Sed	7.41
7	23	Cage	Addition	Sed	1.32
7	23	Cage	Addition	Sed	1.98
7	23	Cage	Addition	Sed	10.60
7	23	Cage	Addition	Sed	4.35
7	23	Cage	Addition	Sed	2.03
7	23	Cage	Addition	Sed	3.81
7	23	Cage	Addition	Sed	2.82
7	23	Cage	Addition	Sed	2.32
7	23	Cage	Addition	Sed	3.30
7	23	Cage	Addition	Sed	5.92
7	23	Cage	Addition	Sed	3.69
7	23	Cage	Addition	Sed	2.63
7	23	Cage	Addition	Sed	3.21
7	28	Half	Addition	Turf	3.03
7	28	Half	Addition	Turf	0.62
7	28	Half	Addition	Turf	2.55
7	28	Half	Addition	Turf	1.41
7	28	Half	Addition	Turf	1.76
7	28	Half	Addition	Turf	1.92
7	28	Half	Addition	Turf	2.38
7	28	Half	Addition	Turf	2.05
7	28	Half	Addition	Turf	4.71
7	28	Half	Addition	Turf	1.29
7	28	Half	Addition	Turf	2.56
7	28	Half	Addition	Turf	0.91
7	28	Half	Addition	Turf	0.74
7	28	Half	Addition	Turf	1.18
7	28	Half	Addition	Turf	2.20

7	28	Half	Addition	Turf	3.01
7	28	Half	Addition	Turf	1.28
7	28	Half	Addition	Turf	2.43
7	28	Half	Addition	Turf	4.69
7	28	Half	Addition	Turf	2.23
7	28	Half	Addition	Sed	0.00
7	28	Half	Addition	Sed	1.14
7	28	Half	Addition	Sed	0.00
7	28	Half	Addition	Sed	0.93
7	28	Half	Addition	Sed	0.00
7	28	Half	Addition	Sed	2.31
7	28	Half	Addition	Sed	2.46
7	28	Half	Addition	Sed	0.00
7	28	Half	Addition	Sed	0.00
7	28	Half	Addition	Sed	1.50
7	28	Half	Addition	Sed	0.42
7	28	Half	Addition	Sed	1.06
7	28	Half	Addition	Sed	1.01
7	28	Half	Addition	Sed	0.73
7	28	Half	Addition	Sed	0.00
7	28	Half	Addition	Sed	1.59
7	28	Half	Addition	Sed	0.80
7	28	Half	Addition	Sed	0.86
7	28	Half	Addition	Sed	2.05
7	28	Half	Addition	Sed	6.07
7	33	Open	Natural	Turf	3.83
7	33	Open	Natural	Turf	2.62
7	33	Open	Natural	Turf	3.02
7	33	Open	Natural	Turf	4.58
7	33	Open	Natural	Turf	1.41
7	33	Open	Natural	Turf	2.61
7	33	Open	Natural	Turf	1.22
7	33	Open	Natural	Turf	1.55
7	33	Open	Natural	Turf	1.37
7	33	Open	Natural	Turf	2.03
7	33	Open	Natural	Turf	1.54
7	33	Open	Natural	Turf	2.43
7	33	Open	Natural	Turf	2.04
7	33	Open	Natural	Turf	2.47
7	33	Open	Natural	Turf	2.46
7	33	Open	Natural	Turf	1.69
7	33	Open	Natural	Turf	2.24
7	33	Open	Natural	Turf	4.16
7	33	Open	Natural	Turf	1.38
7	33	Open	Natural	Turf	2.45
7	33	Open	Natural	Sed	0.91
7	33	Open	Natural	Sed	1.14
7	33	Open	Natural	Sed	0.71
7	33	Open	Natural	Sed	0.00
7	33	Open	Natural	Sed	1.41
7	33	Open	Natural	Sed	1.35
7	33	Open	Natural	Sed	3.09
7	33	Open	Natural	Sed	0.00

7	33	Open	Natural	Sed	0.00
7	33	Open	Natural	Sed	0.00
7	33	Open	Natural	Sed	0.00
7	33	Open	Natural	Sed	3.07
7	33	Open	Natural	Sed	0.00
7	33	Open	Natural	Sed	1.24
7	33	Open	Natural	Sed	1.91
7	33	Open	Natural	Sed	0.00
7	33	Open	Natural	Sed	0.64
7	33	Open	Natural	Sed	1.30
7	33	Open	Natural	Sed	0.93
7	33	Open	Natural	Sed	0.77
7	26	Half	Natural	Turf	1.41
7	26	Half	Natural	Turf	2.03
7	26	Half	Natural	Turf	3.06
7	26	Half	Natural	Turf	5.64
7	26	Half	Natural	Turf	4.72
7	26	Half	Natural	Turf	3.34
7	26	Half	Natural	Turf	2.76
7	26	Half	Natural	Turf	3.72
7	26	Half	Natural	Turf	1.58
7	26	Half	Natural	Turf	1.82
7	26	Half	Natural	Turf	3.05
7	26	Half	Natural	Turf	2.63
7	26	Half	Natural	Turf	2.75
7	26	Half	Natural	Turf	2.58
7	26	Half	Natural	Turf	3.01
7	26	Half	Natural	Turf	1.79
7	26	Half	Natural	Turf	1.40
7	26	Half	Natural	Turf	3.68
7	26	Half	Natural	Turf	2.35
7	26	Half	Natural	Turf	3.46
7	26	Half	Natural	Sed	1.59
7	26	Half	Natural	Sed	1.46
7	26	Half	Natural	Sed	1.31
7	26	Half	Natural	Sed	2.31
7	26	Half	Natural	Sed	1.50
7	26	Half	Natural	Sed	1.18
7	26	Half	Natural	Sed	2.37
7	26	Half	Natural	Sed	2.00
7	26	Half	Natural	Sed	0.98
7	26	Half	Natural	Sed	2.25
7	26	Half	Natural	Sed	4.01
7	26	Half	Natural	Sed	1.02
7	26	Half	Natural	Sed	1.54
7	26	Half	Natural	Sed	1.40
7	26	Half	Natural	Sed	1.41
7	26	Half	Natural	Sed	1.97
7	26	Half	Natural	Sed	2.19
7	26	Half	Natural	Sed	1.26
7	26	Half	Natural	Sed	1.48
7	26	Half	Natural	Sed	2.22
7	1	Cage	Natural	Turf	3.54

7	1	Cage	Natural	Turf	4.04
7	1	Cage	Natural	Turf	3.20
7	1	Cage	Natural	Turf	5.60
7	1	Cage	Natural	Turf	5.97
7	1	Cage	Natural	Turf	2.58
7	1	Cage	Natural	Turf	1.90
7	1	Cage	Natural	Turf	5.65
7	1	Cage	Natural	Turf	4.96
7	1	Cage	Natural	Turf	8.82
7	1	Cage	Natural	Turf	2.73
7	1	Cage	Natural	Turf	6.04
7	1	Cage	Natural	Turf	4.12
7	1	Cage	Natural	Turf	5.21
7	1	Cage	Natural	Turf	3.01
7	1	Cage	Natural	Turf	3.51
7	1	Cage	Natural	Turf	2.85
7	1	Cage	Natural	Turf	3.08
7	1	Cage	Natural	Turf	3.02
7	1	Cage	Natural	Turf	4.71
7	1	Cage	Natural	Sed	1.57
7	1	Cage	Natural	Sed	1.38
7	1	Cage	Natural	Sed	2.21
7	1	Cage	Natural	Sed	2.62
7	1	Cage	Natural	Sed	2.62
7	1	Cage	Natural	Sed	3.79
7	1	Cage	Natural	Sed	2.28
7	1	Cage	Natural	Sed	2.51
7	1	Cage	Natural	Sed	2.82
7	1	Cage	Natural	Sed	1.65
7	1	Cage	Natural	Sed	3.75
7	1	Cage	Natural	Sed	12.65
7	1	Cage	Natural	Sed	1.32
7	1	Cage	Natural	Sed	0.00
7	1	Cage	Natural	Sed	7.49
7	1	Cage	Natural	Sed	2.09
7	1	Cage	Natural	Sed	1.17
7	1	Cage	Natural	Sed	2.60
7	1	Cage	Natural	Sed	1.80
7	1	Cage	Natural	Sed	1.94
7	7	Half	Natural	Turf	2.14
7	7	Half	Natural	Turf	3.55
7	7	Half	Natural	Turf	2.72
7	7	Half	Natural	Turf	10.77
7	7	Half	Natural	Turf	5.13
7	7	Half	Natural	Turf	3.51
7	7	Half	Natural	Turf	4.09
7	7	Half	Natural	Turf	3.49
7	7	Half	Natural	Turf	2.16
7	7	Half	Natural	Turf	2.49
7	7	Half	Natural	Turf	2.49
7	7	Half	Natural	Turf	1.83
7	7	Half	Natural	Turf	1.37
7	7	Half	Natural	Turf	3.15

7	7 Half	Natural	Turf	3.47
7	7 Half	Natural	Turf	2.44
7	7 Half	Natural	Turf	4.83
7	7 Half	Natural	Turf	1.68
7	7 Half	Natural	Turf	2.35
7	7 Half	Natural	Turf	2.53
7	7 Half	Natural	Sed	2.82
7	7 Half	Natural	Sed	5.16
7	7 Half	Natural	Sed	0.97
7	7 Half	Natural	Sed	2.46
7	7 Half	Natural	Sed	1.49
7	7 Half	Natural	Sed	4.79
7	7 Half	Natural	Sed	1.24
7	7 Half	Natural	Sed	1.90
7	7 Half	Natural	Sed	1.84
7	7 Half	Natural	Sed	2.49
7	7 Half	Natural	Sed	3.61
7	7 Half	Natural	Sed	3.65
7	7 Half	Natural	Sed	2.36
7	7 Half	Natural	Sed	3.00
7	7 Half	Natural	Sed	2.40
7	7 Half	Natural	Sed	2.14
7	7 Half	Natural	Sed	2.29
7	7 Half	Natural	Sed	3.16
7	7 Half	Natural	Sed	2.43
7	7 Half	Natural	Sed	1.84
7	13 Open	Natural	Turf	5.05
7	13 Open	Natural	Turf	3.50
7	13 Open	Natural	Turf	2.26
7	13 Open	Natural	Turf	1.95
7	13 Open	Natural	Turf	1.51
7	13 Open	Natural	Turf	3.51
7	13 Open	Natural	Turf	3.22
7	13 Open	Natural	Turf	3.08
7	13 Open	Natural	Turf	5.92
7	13 Open	Natural	Turf	3.12
7	13 Open	Natural	Turf	1.62
7	13 Open	Natural	Turf	1.76
7	13 Open	Natural	Turf	2.45
7	13 Open	Natural	Turf	3.18
7	13 Open	Natural	Turf	1.69
7	13 Open	Natural	Turf	2.56
7	13 Open	Natural	Turf	1.79
7	13 Open	Natural	Turf	2.39
7	13 Open	Natural	Turf	1.24
7	13 Open	Natural	Turf	5.83
7	13 Open	Natural	Sed	1.48
7	13 Open	Natural	Sed	1.16
7	13 Open	Natural	Sed	1.92
7	13 Open	Natural	Sed	2.14
7	13 Open	Natural	Sed	1.25
7	13 Open	Natural	Sed	2.60
7	13 Open	Natural	Sed	2.52

7	13	Open	Natural	Sed	1.55
7	13	Open	Natural	Sed	1.98
7	13	Open	Natural	Sed	2.29
7	13	Open	Natural	Sed	1.42
7	13	Open	Natural	Sed	2.48
7	13	Open	Natural	Sed	2.67
7	13	Open	Natural	Sed	4.17
7	13	Open	Natural	Sed	3.04
7	13	Open	Natural	Sed	3.25
7	13	Open	Natural	Sed	1.61
7	13	Open	Natural	Sed	1.66
7	13	Open	Natural	Sed	3.68
7	13	Open	Natural	Sed	5.06
7	17	Open	Addition	Turf	6.27
7	17	Open	Addition	Turf	4.31
7	17	Open	Addition	Turf	7.00
7	17	Open	Addition	Turf	4.67
7	17	Open	Addition	Turf	3.71
7	17	Open	Addition	Turf	4.97
7	17	Open	Addition	Turf	5.86
7	17	Open	Addition	Turf	6.45
7	17	Open	Addition	Turf	6.60
7	17	Open	Addition	Turf	6.77
7	17	Open	Addition	Turf	3.90
7	17	Open	Addition	Turf	7.51
7	17	Open	Addition	Turf	4.17
7	17	Open	Addition	Turf	3.39
7	17	Open	Addition	Turf	5.53
7	17	Open	Addition	Turf	6.93
7	17	Open	Addition	Turf	4.45
7	17	Open	Addition	Turf	5.66
7	17	Open	Addition	Turf	2.32
7	17	Open	Addition	Turf	2.26
7	17	Open	Addition	Sed	1.17
7	17	Open	Addition	Sed	6.67
7	17	Open	Addition	Sed	3.64
7	17	Open	Addition	Sed	1.65
7	17	Open	Addition	Sed	0.91
7	17	Open	Addition	Sed	0.93
7	17	Open	Addition	Sed	1.93
7	17	Open	Addition	Sed	3.92
7	17	Open	Addition	Sed	2.20
7	17	Open	Addition	Sed	2.01
7	17	Open	Addition	Sed	2.04
7	17	Open	Addition	Sed	1.80
7	17	Open	Addition	Sed	5.58
7	17	Open	Addition	Sed	2.63
7	17	Open	Addition	Sed	2.54
7	17	Open	Addition	Sed	1.41
7	17	Open	Addition	Sed	2.50
7	17	Open	Addition	Sed	1.25
7	17	Open	Addition	Sed	1.34
7	17	Open	Addition	Sed	2.78

7	2	Cage	Natural	Turf	8.34
7	2	Cage	Natural	Turf	3.90
7	2	Cage	Natural	Turf	3.86
7	2	Cage	Natural	Turf	4.39
7	2	Cage	Natural	Turf	2.99
7	2	Cage	Natural	Turf	2.74
7	2	Cage	Natural	Turf	3.20
7	2	Cage	Natural	Turf	3.49
7	2	Cage	Natural	Turf	3.70
7	2	Cage	Natural	Turf	3.71
7	2	Cage	Natural	Turf	4.98
7	2	Cage	Natural	Turf	3.86
7	2	Cage	Natural	Turf	4.46
7	2	Cage	Natural	Turf	5.40
7	2	Cage	Natural	Turf	3.97
7	2	Cage	Natural	Turf	4.23
7	2	Cage	Natural	Turf	3.45
7	2	Cage	Natural	Turf	4.73
7	2	Cage	Natural	Turf	4.09
7	2	Cage	Natural	Turf	3.47
7	2	Cage	Natural	Sed	2.93
7	2	Cage	Natural	Sed	6.38
7	2	Cage	Natural	Sed	2.45
7	2	Cage	Natural	Sed	1.74
7	2	Cage	Natural	Sed	3.33
7	2	Cage	Natural	Sed	4.99
7	2	Cage	Natural	Sed	2.20
7	2	Cage	Natural	Sed	3.67
7	2	Cage	Natural	Sed	3.50
7	2	Cage	Natural	Sed	3.34
7	2	Cage	Natural	Sed	1.41
7	2	Cage	Natural	Sed	0.86
7	2	Cage	Natural	Sed	3.49
7	2	Cage	Natural	Sed	2.39
7	2	Cage	Natural	Sed	1.98
7	2	Cage	Natural	Sed	2.72
7	2	Cage	Natural	Sed	2.14
7	2	Cage	Natural	Sed	4.27
7	2	Cage	Natural	Sed	1.10
7	2	Cage	Natural	Sed	3.11
7	6	Cage	Addition	Turf	4.70
7	6	Cage	Addition	Turf	8.50
7	6	Cage	Addition	Turf	3.16
7	6	Cage	Addition	Turf	3.00
7	6	Cage	Addition	Turf	3.54
7	6	Cage	Addition	Turf	3.71
7	6	Cage	Addition	Turf	2.77
7	6	Cage	Addition	Turf	1.95
7	6	Cage	Addition	Turf	3.47
7	6	Cage	Addition	Turf	2.40
7	6	Cage	Addition	Turf	6.46
7	6	Cage	Addition	Turf	7.25
7	6	Cage	Addition	Turf	8.87

7	6	Cage	Addition	Turf	4.95
7	6	Cage	Addition	Turf	6.53
7	6	Cage	Addition	Turf	4.40
7	6	Cage	Addition	Turf	3.05
7	6	Cage	Addition	Turf	3.05
7	6	Cage	Addition	Turf	5.68
7	6	Cage	Addition	Turf	6.91
7	6	Cage	Addition	Sed	9.51
7	6	Cage	Addition	Sed	3.05
7	6	Cage	Addition	Sed	4.18
7	6	Cage	Addition	Sed	1.62
7	6	Cage	Addition	Sed	5.94
7	6	Cage	Addition	Sed	7.28
7	6	Cage	Addition	Sed	3.90
7	6	Cage	Addition	Sed	2.35
7	6	Cage	Addition	Sed	2.58
7	6	Cage	Addition	Sed	3.65
7	6	Cage	Addition	Sed	2.69
7	6	Cage	Addition	Sed	7.28
7	6	Cage	Addition	Sed	1.74
7	6	Cage	Addition	Sed	1.86
7	6	Cage	Addition	Sed	3.35
7	6	Cage	Addition	Sed	1.66
7	6	Cage	Addition	Sed	1.48
7	6	Cage	Addition	Sed	4.21
7	6	Cage	Addition	Sed	1.28
7	6	Cage	Addition	Sed	1.76
7	10	Half	Addition	Turf	5.45
7	10	Half	Addition	Turf	2.66
7	10	Half	Addition	Turf	2.21
7	10	Half	Addition	Turf	4.38
7	10	Half	Addition	Turf	3.12
7	10	Half	Addition	Turf	3.76
7	10	Half	Addition	Turf	1.90
7	10	Half	Addition	Turf	2.62
7	10	Half	Addition	Turf	2.23
7	10	Half	Addition	Turf	2.45
7	10	Half	Addition	Turf	2.38
7	10	Half	Addition	Turf	3.36
7	10	Half	Addition	Turf	1.65
7	10	Half	Addition	Turf	6.46
7	10	Half	Addition	Turf	2.02
7	10	Half	Addition	Turf	3.93
7	10	Half	Addition	Turf	2.30
7	10	Half	Addition	Turf	2.42
7	10	Half	Addition	Turf	2.57
7	10	Half	Addition	Turf	2.38
7	10	Half	Addition	Sed	2.05
7	10	Half	Addition	Sed	0.84
7	10	Half	Addition	Sed	1.83
7	10	Half	Addition	Sed	2.98
7	10	Half	Addition	Sed	1.22
7	10	Half	Addition	Sed	1.04

7	10	Half	Addition	Sed	0.72
7	10	Half	Addition	Sed	2.47
7	10	Half	Addition	Sed	1.62
7	10	Half	Addition	Sed	0.89
7	10	Half	Addition	Sed	1.54
7	10	Half	Addition	Sed	8.85
7	10	Half	Addition	Sed	1.23
7	10	Half	Addition	Sed	1.31
7	10	Half	Addition	Sed	2.41
7	10	Half	Addition	Sed	6.41
7	10	Half	Addition	Sed	1.75
7	10	Half	Addition	Sed	1.55
7	10	Half	Addition	Sed	1.40
7	10	Half	Addition	Sed	2.10
7	9	Half	Natural	Turf	1.92
7	9	Half	Natural	Turf	3.99
7	9	Half	Natural	Turf	4.98
7	9	Half	Natural	Turf	1.67
7	9	Half	Natural	Turf	3.68
7	9	Half	Natural	Turf	6.67
7	9	Half	Natural	Turf	2.66
7	9	Half	Natural	Turf	6.78
7	9	Half	Natural	Turf	5.56
7	9	Half	Natural	Turf	3.04
7	9	Half	Natural	Turf	3.08
7	9	Half	Natural	Turf	5.90
7	9	Half	Natural	Turf	2.43
7	9	Half	Natural	Turf	4.63
7	9	Half	Natural	Turf	3.37
7	9	Half	Natural	Turf	5.46
7	9	Half	Natural	Turf	7.14
7	9	Half	Natural	Turf	2.04
7	9	Half	Natural	Turf	1.80
7	9	Half	Natural	Turf	3.25
7	9	Half	Natural	Sed	3.07
7	9	Half	Natural	Sed	1.30
7	9	Half	Natural	Sed	10.69
7	9	Half	Natural	Sed	1.44
7	9	Half	Natural	Sed	6.03
7	9	Half	Natural	Sed	2.45
7	9	Half	Natural	Sed	1.70
7	9	Half	Natural	Sed	3.36
7	9	Half	Natural	Sed	1.74
7	9	Half	Natural	Sed	2.86
7	9	Half	Natural	Sed	1.67
7	9	Half	Natural	Sed	3.23
7	9	Half	Natural	Sed	1.99
7	9	Half	Natural	Sed	2.40
7	9	Half	Natural	Sed	2.44
7	9	Half	Natural	Sed	2.78
7	9	Half	Natural	Sed	1.77
7	9	Half	Natural	Sed	1.82
7	9	Half	Natural	Sed	16.37

7	9	Half	Natural	Sed	1.86
7	21	Cage	Natural	Turf	3.31
7	21	Cage	Natural	Turf	2.92
7	21	Cage	Natural	Turf	3.39
7	21	Cage	Natural	Turf	5.50
7	21	Cage	Natural	Turf	2.93
7	21	Cage	Natural	Turf	2.46
7	21	Cage	Natural	Turf	4.95
7	21	Cage	Natural	Turf	2.02
7	21	Cage	Natural	Turf	4.69
7	21	Cage	Natural	Turf	4.21
7	21	Cage	Natural	Turf	5.87
7	21	Cage	Natural	Turf	4.24
7	21	Cage	Natural	Turf	4.18
7	21	Cage	Natural	Turf	4.02
7	21	Cage	Natural	Turf	4.91
7	21	Cage	Natural	Turf	7.98
7	21	Cage	Natural	Turf	3.48
7	21	Cage	Natural	Turf	6.25
7	21	Cage	Natural	Turf	5.15
7	21	Cage	Natural	Turf	20.09
7	21	Cage	Natural	Sed	2.98
7	21	Cage	Natural	Sed	1.12
7	21	Cage	Natural	Sed	3.32
7	21	Cage	Natural	Sed	2.43
7	21	Cage	Natural	Sed	2.44
7	21	Cage	Natural	Sed	2.83
7	21	Cage	Natural	Sed	1.68
7	21	Cage	Natural	Sed	2.23
7	21	Cage	Natural	Sed	1.37
7	21	Cage	Natural	Sed	0.63
7	21	Cage	Natural	Sed	2.55
7	21	Cage	Natural	Sed	3.09
7	21	Cage	Natural	Sed	2.32
7	21	Cage	Natural	Sed	3.38
7	21	Cage	Natural	Sed	2.04
7	21	Cage	Natural	Sed	5.44
7	21	Cage	Natural	Sed	1.05
7	21	Cage	Natural	Sed	1.57
7	21	Cage	Natural	Sed	3.67
7	21	Cage	Natural	Sed	8.27
7	22	Cage	Addition	Turf	4.29
7	22	Cage	Addition	Turf	4.92
7	22	Cage	Addition	Turf	2.24
7	22	Cage	Addition	Turf	4.79
7	22	Cage	Addition	Turf	4.05
7	22	Cage	Addition	Turf	4.39
7	22	Cage	Addition	Turf	1.76
7	22	Cage	Addition	Turf	5.65
7	22	Cage	Addition	Turf	4.14
7	22	Cage	Addition	Turf	4.65
7	22	Cage	Addition	Turf	15.99
7	22	Cage	Addition	Turf	12.60

7	22	Cage	Addition	Turf	3.62
7	22	Cage	Addition	Turf	4.53
7	22	Cage	Addition	Turf	0.00
7	22	Cage	Addition	Turf	3.00
7	22	Cage	Addition	Turf	4.70
7	22	Cage	Addition	Turf	3.78
7	22	Cage	Addition	Turf	5.11
7	22	Cage	Addition	Turf	3.82
7	22	Cage	Addition	Sed	2.00
7	22	Cage	Addition	Sed	1.14
7	22	Cage	Addition	Sed	6.53
7	22	Cage	Addition	Sed	2.01
7	22	Cage	Addition	Sed	2.66
7	22	Cage	Addition	Sed	2.86
7	22	Cage	Addition	Sed	12.07
7	22	Cage	Addition	Sed	3.29
7	22	Cage	Addition	Sed	3.14
7	22	Cage	Addition	Sed	1.59
7	22	Cage	Addition	Sed	5.54
7	22	Cage	Addition	Sed	1.73
7	22	Cage	Addition	Sed	1.17
7	22	Cage	Addition	Sed	4.34
7	22	Cage	Addition	Sed	1.15
7	22	Cage	Addition	Sed	1.87
7	22	Cage	Addition	Sed	1.88
7	22	Cage	Addition	Sed	1.18
7	22	Cage	Addition	Sed	1.81
7	22	Cage	Addition	Sed	3.25
7	36	Open	Addition	Turf	3.37
7	36	Open	Addition	Turf	3.05
7	36	Open	Addition	Turf	2.57
7	36	Open	Addition	Turf	3.34
7	36	Open	Addition	Turf	2.97
7	36	Open	Addition	Turf	1.82
7	36	Open	Addition	Turf	3.38
7	36	Open	Addition	Turf	3.70
7	36	Open	Addition	Turf	2.80
7	36	Open	Addition	Turf	2.14
7	36	Open	Addition	Turf	2.46
7	36	Open	Addition	Turf	9.05
7	36	Open	Addition	Turf	4.53
7	36	Open	Addition	Turf	4.46
7	36	Open	Addition	Turf	3.94
7	36	Open	Addition	Turf	2.74
7	36	Open	Addition	Turf	2.15
7	36	Open	Addition	Turf	3.34
7	36	Open	Addition	Turf	3.43
7	36	Open	Addition	Turf	3.09
7	36	Open	Addition	Sed	1.88
7	36	Open	Addition	Sed	0.63
7	36	Open	Addition	Sed	2.32
7	36	Open	Addition	Sed	0.00
7	36	Open	Addition	Sed	0.00

7	36	Open	Addition	Sed	1.52
7	36	Open	Addition	Sed	2.43
7	36	Open	Addition	Sed	1.18
7	36	Open	Addition	Sed	1.81
7	36	Open	Addition	Sed	1.58
7	36	Open	Addition	Sed	1.70
7	36	Open	Addition	Sed	0.98
7	36	Open	Addition	Sed	5.00
7	36	Open	Addition	Sed	1.76
7	36	Open	Addition	Sed	0.74
7	36	Open	Addition	Sed	2.05
7	36	Open	Addition	Sed	0.80
7	36	Open	Addition	Sed	2.00
7	36	Open	Addition	Sed	1.00
7	36	Open	Addition	Sed	1.49
7	19	Cage	Natural	Turf	3.65
7	19	Cage	Natural	Turf	4.16
7	19	Cage	Natural	Turf	9.60
7	19	Cage	Natural	Turf	9.43
7	19	Cage	Natural	Turf	6.84
7	19	Cage	Natural	Turf	6.49
7	19	Cage	Natural	Turf	13.34
7	19	Cage	Natural	Turf	5.97
7	19	Cage	Natural	Turf	6.43
7	19	Cage	Natural	Turf	6.95
7	19	Cage	Natural	Turf	9.25
7	19	Cage	Natural	Turf	15.16
7	19	Cage	Natural	Turf	8.22
7	19	Cage	Natural	Turf	7.50
7	19	Cage	Natural	Turf	7.23
7	19	Cage	Natural	Turf	25.99
7	19	Cage	Natural	Turf	3.25
7	19	Cage	Natural	Turf	3.02
7	19	Cage	Natural	Turf	4.11
7	19	Cage	Natural	Turf	5.45
7	19	Cage	Natural	Sed	4.94
7	19	Cage	Natural	Sed	11.77
7	19	Cage	Natural	Sed	2.50
7	19	Cage	Natural	Sed	3.83
7	19	Cage	Natural	Sed	3.54
7	19	Cage	Natural	Sed	3.59
7	19	Cage	Natural	Sed	9.51
7	19	Cage	Natural	Sed	6.73
7	19	Cage	Natural	Sed	2.64
7	19	Cage	Natural	Sed	5.80
7	19	Cage	Natural	Sed	7.88
7	19	Cage	Natural	Sed	8.81
7	19	Cage	Natural	Sed	4.09
7	19	Cage	Natural	Sed	6.79
7	19	Cage	Natural	Sed	3.71
7	19	Cage	Natural	Sed	6.45
7	19	Cage	Natural	Sed	4.92
7	19	Cage	Natural	Sed	5.51

7	19	Cage	Natural	Sed	1.87
7	19	Cage	Natural	Sed	1.74
7	34	Open	Addition	Turf	9.22
7	34	Open	Addition	Turf	3.68
7	34	Open	Addition	Turf	4.28
7	34	Open	Addition	Turf	8.56
7	34	Open	Addition	Turf	7.48
7	34	Open	Addition	Turf	10.20
7	34	Open	Addition	Turf	2.77
7	34	Open	Addition	Turf	3.27
7	34	Open	Addition	Turf	4.40
7	34	Open	Addition	Turf	9.76
7	34	Open	Addition	Turf	5.97
7	34	Open	Addition	Turf	8.26
7	34	Open	Addition	Turf	6.00
7	34	Open	Addition	Turf	2.75
7	34	Open	Addition	Turf	4.60
7	34	Open	Addition	Turf	4.44
7	34	Open	Addition	Turf	7.54
7	34	Open	Addition	Turf	7.75
7	34	Open	Addition	Turf	4.83
7	34	Open	Addition	Turf	2.49
7	34	Open	Addition	Sed	11.46
7	34	Open	Addition	Sed	5.28
7	34	Open	Addition	Sed	3.01
7	34	Open	Addition	Sed	4.48
7	34	Open	Addition	Sed	1.96
7	34	Open	Addition	Sed	2.61
7	34	Open	Addition	Sed	11.36
7	34	Open	Addition	Sed	5.39
7	34	Open	Addition	Sed	4.95
7	34	Open	Addition	Sed	4.27
7	34	Open	Addition	Sed	9.67
7	34	Open	Addition	Sed	4.62
7	34	Open	Addition	Sed	6.97
7	34	Open	Addition	Sed	16.06
7	34	Open	Addition	Sed	2.68
7	34	Open	Addition	Sed	2.72
7	34	Open	Addition	Sed	2.78
7	34	Open	Addition	Sed	2.28
7	34	Open	Addition	Sed	1.52
7	34	Open	Addition	Sed	17.50
7	25	Half	Natural	Turf	9.93
7	25	Half	Natural	Turf	2.89
7	25	Half	Natural	Turf	5.50
7	25	Half	Natural	Turf	3.76
7	25	Half	Natural	Turf	5.50
7	25	Half	Natural	Turf	2.49
7	25	Half	Natural	Turf	2.55
7	25	Half	Natural	Turf	8.50
7	25	Half	Natural	Turf	5.50
7	25	Half	Natural	Turf	2.56
7	25	Half	Natural	Turf	2.44

7	25 Half	Natural	Turf	9.59
7	25 Half	Natural	Turf	10.04
7	25 Half	Natural	Turf	11.66
7	25 Half	Natural	Turf	4.64
7	25 Half	Natural	Turf	6.37
7	25 Half	Natural	Turf	5.44
7	25 Half	Natural	Turf	2.12
7	25 Half	Natural	Turf	4.30
7	25 Half	Natural	Turf	6.98
7	25 Half	Natural	Sed	4.33
7	25 Half	Natural	Sed	7.01
7	25 Half	Natural	Sed	5.01
7	25 Half	Natural	Sed	1.92
7	25 Half	Natural	Sed	5.29
7	25 Half	Natural	Sed	3.55
7	25 Half	Natural	Sed	2.78
7	25 Half	Natural	Sed	1.08
7	25 Half	Natural	Sed	1.76
7	25 Half	Natural	Sed	2.10
7	25 Half	Natural	Sed	2.45
7	25 Half	Natural	Sed	2.18
7	25 Half	Natural	Sed	4.26
7	25 Half	Natural	Sed	6.38
7	25 Half	Natural	Sed	2.59
7	25 Half	Natural	Sed	3.22
7	25 Half	Natural	Sed	4.20
7	25 Half	Natural	Sed	7.28
7	25 Half	Natural	Sed	1.96
7	25 Half	Natural	Sed	2.23
7	24 Cage	Addition	Turf	4.39
7	24 Cage	Addition	Turf	5.66
7	24 Cage	Addition	Turf	4.65
7	24 Cage	Addition	Turf	14.12
7	24 Cage	Addition	Turf	5.11
7	24 Cage	Addition	Turf	12.58
7	24 Cage	Addition	Turf	5.15
7	24 Cage	Addition	Turf	4.69
7	24 Cage	Addition	Turf	13.45
7	24 Cage	Addition	Turf	5.29
7	24 Cage	Addition	Turf	14.09
7	24 Cage	Addition	Turf	4.65
7	24 Cage	Addition	Turf	8.34
7	24 Cage	Addition	Turf	6.07
7	24 Cage	Addition	Turf	6.01
7	24 Cage	Addition	Turf	6.58
7	24 Cage	Addition	Turf	12.12
7	24 Cage	Addition	Turf	5.75
7	24 Cage	Addition	Turf	21.92
7	24 Cage	Addition	Turf	19.11
7	24 Cage	Addition	Sed	3.43
7	24 Cage	Addition	Sed	14.34
7	24 Cage	Addition	Sed	5.72
7	24 Cage	Addition	Sed	11.47

7	24 Cage	Addition	Sed	4.61
7	24 Cage	Addition	Sed	4.27
7	24 Cage	Addition	Sed	1.80
7	24 Cage	Addition	Sed	5.03
7	24 Cage	Addition	Sed	1.80
7	24 Cage	Addition	Sed	2.05
7	24 Cage	Addition	Sed	4.27
7	24 Cage	Addition	Sed	3.97
7	24 Cage	Addition	Sed	2.88
7	24 Cage	Addition	Sed	4.07
7	24 Cage	Addition	Sed	2.77
7	24 Cage	Addition	Sed	3.77
7	24 Cage	Addition	Sed	4.18
7	24 Cage	Addition	Sed	3.16
7	24 Cage	Addition	Sed	2.49
7	24 Cage	Addition	Sed	2.88
7	29 Half	Addition	Turf	4.24
7	29 Half	Addition	Turf	4.01
7	29 Half	Addition	Turf	5.02
7	29 Half	Addition	Turf	4.44
7	29 Half	Addition	Turf	2.67
7	29 Half	Addition	Turf	2.12
7	29 Half	Addition	Turf	2.85
7	29 Half	Addition	Turf	2.78
7	29 Half	Addition	Turf	2.04
7	29 Half	Addition	Turf	2.75
7	29 Half	Addition	Turf	1.72
7	29 Half	Addition	Turf	6.21
7	29 Half	Addition	Turf	2.45
7	29 Half	Addition	Turf	2.08
7	29 Half	Addition	Turf	5.10
7	29 Half	Addition	Turf	3.71
7	29 Half	Addition	Turf	2.34
7	29 Half	Addition	Turf	4.04
7	29 Half	Addition	Turf	3.02
7	29 Half	Addition	Turf	2.24
7	29 Half	Addition	Sed	1.38
7	29 Half	Addition	Sed	3.08
7	29 Half	Addition	Sed	0.91
7	29 Half	Addition	Sed	1.20
7	29 Half	Addition	Sed	2.89
7	29 Half	Addition	Sed	1.24
7	29 Half	Addition	Sed	0.58
7	29 Half	Addition	Sed	1.78
7	29 Half	Addition	Sed	2.77
7	29 Half	Addition	Sed	1.37
7	29 Half	Addition	Sed	1.71
7	29 Half	Addition	Sed	2.26
7	29 Half	Addition	Sed	2.03
7	29 Half	Addition	Sed	3.56
7	29 Half	Addition	Sed	0.81
7	29 Half	Addition	Sed	1.91
7	29 Half	Addition	Sed	3.23

7	29	Half	Addition	Sed	1.56
7	29	Half	Addition	Sed	1.60
7	29	Half	Addition	Sed	1.31
7	3	Cage	Natural	Turf	5.39
7	3	Cage	Natural	Turf	3.12
7	3	Cage	Natural	Turf	3.16
7	3	Cage	Natural	Turf	3.05
7	3	Cage	Natural	Turf	3.33
7	3	Cage	Natural	Turf	4.07
7	3	Cage	Natural	Turf	5.40
7	3	Cage	Natural	Turf	2.84
7	3	Cage	Natural	Turf	2.86
7	3	Cage	Natural	Turf	2.96
7	3	Cage	Natural	Turf	8.20
7	3	Cage	Natural	Turf	1.87
7	3	Cage	Natural	Turf	3.87
7	3	Cage	Natural	Turf	3.28
7	3	Cage	Natural	Turf	2.49
7	3	Cage	Natural	Turf	7.54
7	3	Cage	Natural	Turf	2.91
7	3	Cage	Natural	Turf	2.89
7	3	Cage	Natural	Turf	2.06
7	3	Cage	Natural	Turf	2.91
7	3	Cage	Natural	Sed	2.02
7	3	Cage	Natural	Sed	1.53
7	3	Cage	Natural	Sed	2.54
7	3	Cage	Natural	Sed	1.82
7	3	Cage	Natural	Sed	2.38
7	3	Cage	Natural	Sed	3.18
7	3	Cage	Natural	Sed	3.48
7	3	Cage	Natural	Sed	0.61
7	3	Cage	Natural	Sed	2.47
7	3	Cage	Natural	Sed	2.72
7	3	Cage	Natural	Sed	4.97
7	3	Cage	Natural	Sed	1.42
7	3	Cage	Natural	Sed	1.20
7	3	Cage	Natural	Sed	3.82
7	3	Cage	Natural	Sed	1.87
7	3	Cage	Natural	Sed	2.55
7	3	Cage	Natural	Sed	1.50
7	3	Cage	Natural	Sed	1.18
7	3	Cage	Natural	Sed	1.90
7	3	Cage	Natural	Sed	2.59
7	16	Open	Addition	Turf	2.47
7	16	Open	Addition	Turf	4.90
7	16	Open	Addition	Turf	2.02
7	16	Open	Addition	Turf	7.42
7	16	Open	Addition	Turf	8.82
7	16	Open	Addition	Turf	4.95
7	16	Open	Addition	Turf	2.18
7	16	Open	Addition	Turf	2.75
7	16	Open	Addition	Turf	2.94
7	16	Open	Addition	Turf	3.39

7	16	Open	Addition	Turf	4.77
7	16	Open	Addition	Turf	3.66
7	16	Open	Addition	Turf	1.60
7	16	Open	Addition	Turf	2.62
7	16	Open	Addition	Turf	2.24
7	16	Open	Addition	Turf	3.35
7	16	Open	Addition	Turf	5.53
7	16	Open	Addition	Turf	4.67
7	16	Open	Addition	Turf	2.13
7	16	Open	Addition	Turf	6.75
7	16	Open	Addition	Sed	1.00
7	16	Open	Addition	Sed	2.11
7	16	Open	Addition	Sed	1.51
7	16	Open	Addition	Sed	1.16
7	16	Open	Addition	Sed	2.76
7	16	Open	Addition	Sed	1.98
7	16	Open	Addition	Sed	1.98
7	16	Open	Addition	Sed	1.28
7	16	Open	Addition	Sed	2.19
7	16	Open	Addition	Sed	1.45
7	16	Open	Addition	Sed	1.46
7	16	Open	Addition	Sed	1.76
7	16	Open	Addition	Sed	1.56
7	16	Open	Addition	Sed	2.88
7	16	Open	Addition	Sed	1.66
7	16	Open	Addition	Sed	2.42
7	16	Open	Addition	Sed	1.52
7	16	Open	Addition	Sed	1.12
7	16	Open	Addition	Sed	1.51
7	16	Open	Addition	Sed	5.68
7	11	Half	Addition	Turf	3.09
7	11	Half	Addition	Turf	2.74
7	11	Half	Addition	Turf	4.44
7	11	Half	Addition	Turf	2.87
7	11	Half	Addition	Turf	2.36
7	11	Half	Addition	Turf	12.63
7	11	Half	Addition	Turf	8.22
7	11	Half	Addition	Turf	2.90
7	11	Half	Addition	Turf	6.27
7	11	Half	Addition	Turf	13.83
7	11	Half	Addition	Turf	1.82
7	11	Half	Addition	Turf	2.55
7	11	Half	Addition	Turf	4.73
7	11	Half	Addition	Turf	6.76
7	11	Half	Addition	Turf	2.18
7	11	Half	Addition	Turf	3.70
7	11	Half	Addition	Turf	3.11
7	11	Half	Addition	Turf	2.89
7	11	Half	Addition	Turf	3.20
7	11	Half	Addition	Turf	7.51
7	11	Half	Addition	Sed	1.72
7	11	Half	Addition	Sed	2.25
7	11	Half	Addition	Sed	0.76

7	11	Half	Addition	Sed	1.10
7	11	Half	Addition	Sed	1.86
7	11	Half	Addition	Sed	2.44
7	11	Half	Addition	Sed	2.41
7	11	Half	Addition	Sed	1.36
7	11	Half	Addition	Sed	2.46
7	11	Half	Addition	Sed	1.45
7	11	Half	Addition	Sed	3.68
7	11	Half	Addition	Sed	2.21
7	11	Half	Addition	Sed	2.07
7	11	Half	Addition	Sed	2.44
7	11	Half	Addition	Sed	1.17
7	11	Half	Addition	Sed	3.92
7	11	Half	Addition	Sed	2.00
7	11	Half	Addition	Sed	1.78
7	11	Half	Addition	Sed	1.43
7	11	Half	Addition	Sed	1.73
7	18	Open	Addition	Turf	4.69
7	18	Open	Addition	Turf	5.28
7	18	Open	Addition	Turf	2.17
7	18	Open	Addition	Turf	2.06
7	18	Open	Addition	Turf	2.83
7	18	Open	Addition	Turf	3.51
7	18	Open	Addition	Turf	3.05
7	18	Open	Addition	Turf	3.35
7	18	Open	Addition	Turf	3.98
7	18	Open	Addition	Turf	2.55
7	18	Open	Addition	Turf	3.15
7	18	Open	Addition	Turf	5.47
7	18	Open	Addition	Turf	3.09
7	18	Open	Addition	Turf	3.36
7	18	Open	Addition	Turf	2.28
7	18	Open	Addition	Turf	4.58
7	18	Open	Addition	Turf	3.86
7	18	Open	Addition	Turf	2.37
7	18	Open	Addition	Turf	2.85
7	18	Open	Addition	Turf	6.82
7	18	Open	Addition	Sed	0.55
7	18	Open	Addition	Sed	1.53
7	18	Open	Addition	Sed	2.43
7	18	Open	Addition	Sed	1.86
7	18	Open	Addition	Sed	2.85
7	18	Open	Addition	Sed	1.53
7	18	Open	Addition	Sed	1.84
7	18	Open	Addition	Sed	2.89
7	18	Open	Addition	Sed	5.61
7	18	Open	Addition	Sed	3.16
7	18	Open	Addition	Sed	1.06
7	18	Open	Addition	Sed	3.79
7	18	Open	Addition	Sed	1.80
7	18	Open	Addition	Sed	1.96
7	18	Open	Addition	Sed	2.17
7	18	Open	Addition	Sed	2.58

7	18	Open	Addition	Sed	2.58
7	18	Open	Addition	Sed	0.85
7	18	Open	Addition	Sed	1.44
7	18	Open	Addition	Sed	1.78
7	4	Cage	Addition	Turf	4.62
7	4	Cage	Addition	Turf	4.27
7	4	Cage	Addition	Turf	13.41
7	4	Cage	Addition	Turf	5.34
7	4	Cage	Addition	Turf	3.66
7	4	Cage	Addition	Turf	5.38
7	4	Cage	Addition	Turf	3.30
7	4	Cage	Addition	Turf	6.35
7	4	Cage	Addition	Turf	5.16
7	4	Cage	Addition	Turf	4.57
7	4	Cage	Addition	Turf	1.87
7	4	Cage	Addition	Turf	2.04
7	4	Cage	Addition	Turf	3.79
7	4	Cage	Addition	Turf	4.23
7	4	Cage	Addition	Turf	2.92
7	4	Cage	Addition	Turf	3.29
7	4	Cage	Addition	Turf	2.72
7	4	Cage	Addition	Turf	3.13
7	4	Cage	Addition	Turf	2.49
7	4	Cage	Addition	Turf	7.69
7	4	Cage	Addition	Sed	5.18
7	4	Cage	Addition	Sed	1.47
7	4	Cage	Addition	Sed	3.10
7	4	Cage	Addition	Sed	2.68
7	4	Cage	Addition	Sed	2.00
7	4	Cage	Addition	Sed	1.78
7	4	Cage	Addition	Sed	0.64
7	4	Cage	Addition	Sed	5.73
7	4	Cage	Addition	Sed	2.35
7	4	Cage	Addition	Sed	1.47
7	4	Cage	Addition	Sed	4.42
7	4	Cage	Addition	Sed	2.00
7	4	Cage	Addition	Sed	1.45
7	4	Cage	Addition	Sed	2.32
7	4	Cage	Addition	Sed	2.59
7	4	Cage	Addition	Sed	5.95
7	4	Cage	Addition	Sed	2.50
7	4	Cage	Addition	Sed	1.81
7	4	Cage	Addition	Sed	8.28
7	4	Cage	Addition	Sed	9.31
7	14	Open	Natural	Turf	1.67
7	14	Open	Natural	Turf	1.90
7	14	Open	Natural	Turf	2.87
7	14	Open	Natural	Turf	2.62
7	14	Open	Natural	Turf	6.38
7	14	Open	Natural	Turf	2.39
7	14	Open	Natural	Turf	2.39
7	14	Open	Natural	Turf	3.68
7	14	Open	Natural	Turf	1.87

7	14	Open	Natural	Turf	3.92
7	14	Open	Natural	Turf	4.65
7	14	Open	Natural	Turf	1.84
7	14	Open	Natural	Turf	4.68
7	14	Open	Natural	Turf	3.78
7	14	Open	Natural	Turf	2.59
7	14	Open	Natural	Turf	1.84
7	14	Open	Natural	Turf	3.65
7	14	Open	Natural	Turf	2.41
7	14	Open	Natural	Turf	2.33
7	14	Open	Natural	Turf	1.56
7	14	Open	Natural	Sed	4.46
7	14	Open	Natural	Sed	1.75
7	14	Open	Natural	Sed	2.08
7	14	Open	Natural	Sed	2.20
7	14	Open	Natural	Sed	1.32
7	14	Open	Natural	Sed	1.72
7	14	Open	Natural	Sed	1.30
7	14	Open	Natural	Sed	1.40
7	14	Open	Natural	Sed	1.12
7	14	Open	Natural	Sed	1.73
7	14	Open	Natural	Sed	1.66
7	14	Open	Natural	Sed	3.04
7	14	Open	Natural	Sed	2.30
7	14	Open	Natural	Sed	1.65
7	14	Open	Natural	Sed	1.49
7	14	Open	Natural	Sed	3.21
7	14	Open	Natural	Sed	3.38
7	14	Open	Natural	Sed	1.91
7	14	Open	Natural	Sed	2.45
7	14	Open	Natural	Sed	2.48
7	12	Half	Addition	Turf	4.08
7	12	Half	Addition	Turf	4.43
7	12	Half	Addition	Turf	4.16
7	12	Half	Addition	Turf	4.38
7	12	Half	Addition	Turf	5.19
7	12	Half	Addition	Turf	1.88
7	12	Half	Addition	Turf	4.91
7	12	Half	Addition	Turf	8.26
7	12	Half	Addition	Turf	2.18
7	12	Half	Addition	Turf	3.00
7	12	Half	Addition	Turf	3.01
7	12	Half	Addition	Turf	2.96
7	12	Half	Addition	Turf	4.20
7	12	Half	Addition	Turf	3.74
7	12	Half	Addition	Turf	5.52
7	12	Half	Addition	Turf	2.40
7	12	Half	Addition	Turf	4.27
7	12	Half	Addition	Turf	4.76
7	12	Half	Addition	Turf	5.55
7	12	Half	Addition	Turf	10.36
7	12	Half	Addition	Sed	5.59
7	12	Half	Addition	Sed	1.10

7	12	Half	Addition	Sed	3.07
7	12	Half	Addition	Sed	3.09
7	12	Half	Addition	Sed	1.34
7	12	Half	Addition	Sed	1.27
7	12	Half	Addition	Sed	1.12
7	12	Half	Addition	Sed	9.68
7	12	Half	Addition	Sed	1.81
7	12	Half	Addition	Sed	2.19
7	12	Half	Addition	Sed	1.67
7	12	Half	Addition	Sed	3.10
7	12	Half	Addition	Sed	3.29
7	12	Half	Addition	Sed	2.54
7	12	Half	Addition	Sed	2.93
7	12	Half	Addition	Sed	3.61
7	12	Half	Addition	Sed	4.17
7	12	Half	Addition	Sed	1.69
7	12	Half	Addition	Sed	2.93
7	12	Half	Addition	Sed	4.78
7	5	Cage	Addition	Turf	1.64
7	5	Cage	Addition	Turf	5.58
7	5	Cage	Addition	Turf	2.10
7	5	Cage	Addition	Turf	3.52
7	5	Cage	Addition	Turf	2.38
7	5	Cage	Addition	Turf	2.43
7	5	Cage	Addition	Turf	1.64
7	5	Cage	Addition	Turf	2.78
7	5	Cage	Addition	Turf	1.74
7	5	Cage	Addition	Turf	2.22
7	5	Cage	Addition	Turf	2.34
7	5	Cage	Addition	Turf	4.80
7	5	Cage	Addition	Turf	2.36
7	5	Cage	Addition	Turf	2.26
7	5	Cage	Addition	Turf	1.90
7	5	Cage	Addition	Turf	3.86
7	5	Cage	Addition	Turf	2.50
7	5	Cage	Addition	Turf	1.74
7	5	Cage	Addition	Turf	2.10
7	5	Cage	Addition	Turf	3.46
7	5	Cage	Addition	Sed	1.17
7	5	Cage	Addition	Sed	1.00
7	5	Cage	Addition	Sed	1.07
7	5	Cage	Addition	Sed	1.49
7	5	Cage	Addition	Sed	1.91
7	5	Cage	Addition	Sed	1.88
7	5	Cage	Addition	Sed	1.14
7	5	Cage	Addition	Sed	2.40
7	5	Cage	Addition	Sed	1.89
7	5	Cage	Addition	Sed	1.25
7	5	Cage	Addition	Sed	1.92
7	5	Cage	Addition	Sed	1.14
7	5	Cage	Addition	Sed	2.67
7	5	Cage	Addition	Sed	1.23
7	5	Cage	Addition	Sed	1.05

7	5	Cage	Addition	Sed	1.44
7	5	Cage	Addition	Sed	2.19
7	5	Cage	Addition	Sed	1.79
7	5	Cage	Addition	Sed	1.82
7	5	Cage	Addition	Sed	0.95
7	8	Half	Natural	Turf	2.45
7	8	Half	Natural	Turf	2.65
7	8	Half	Natural	Turf	4.24
7	8	Half	Natural	Turf	3.70
7	8	Half	Natural	Turf	1.42
7	8	Half	Natural	Turf	2.37
7	8	Half	Natural	Turf	2.68
7	8	Half	Natural	Turf	1.62
7	8	Half	Natural	Turf	6.18
7	8	Half	Natural	Turf	2.62
7	8	Half	Natural	Turf	1.40
7	8	Half	Natural	Turf	3.70
7	8	Half	Natural	Turf	1.91
7	8	Half	Natural	Turf	5.02
7	8	Half	Natural	Turf	2.52
7	8	Half	Natural	Turf	1.36
7	8	Half	Natural	Turf	3.00
7	8	Half	Natural	Turf	2.19
7	8	Half	Natural	Turf	2.86
7	8	Half	Natural	Turf	2.06
7	8	Half	Natural	Sed	0.86
7	8	Half	Natural	Sed	1.21
7	8	Half	Natural	Sed	1.58
7	8	Half	Natural	Sed	0.93
7	8	Half	Natural	Sed	1.15
7	8	Half	Natural	Sed	1.25
7	8	Half	Natural	Sed	0.54
7	8	Half	Natural	Sed	2.66
7	8	Half	Natural	Sed	1.86
7	8	Half	Natural	Sed	1.71
7	8	Half	Natural	Sed	1.47
7	8	Half	Natural	Sed	2.37
7	8	Half	Natural	Sed	0.74
7	8	Half	Natural	Sed	2.84
7	8	Half	Natural	Sed	1.13
7	8	Half	Natural	Sed	0.76
7	8	Half	Natural	Sed	1.64
7	8	Half	Natural	Sed	2.65
7	8	Half	Natural	Sed	0.62
7	8	Half	Natural	Sed	1.46
7	15	Open	Natural	Turf	1.89
7	15	Open	Natural	Turf	1.81
7	15	Open	Natural	Turf	0.80
7	15	Open	Natural	Turf	2.26
7	15	Open	Natural	Turf	2.82
7	15	Open	Natural	Turf	2.56
7	15	Open	Natural	Turf	3.63
7	15	Open	Natural	Turf	3.70

7	15	Open	Natural	Turf	1.58
7	15	Open	Natural	Turf	1.21
7	15	Open	Natural	Turf	1.24
7	15	Open	Natural	Turf	2.44
7	15	Open	Natural	Turf	1.93
7	15	Open	Natural	Turf	6.87
7	15	Open	Natural	Turf	2.42
7	15	Open	Natural	Turf	2.27
7	15	Open	Natural	Turf	3.43
7	15	Open	Natural	Turf	3.21
7	15	Open	Natural	Turf	2.29
7	15	Open	Natural	Turf	3.06
7	15	Open	Natural	Sed	1.14
7	15	Open	Natural	Sed	2.04
7	15	Open	Natural	Sed	3.77
7	15	Open	Natural	Sed	0.65
7	15	Open	Natural	Sed	3.51
7	15	Open	Natural	Sed	0.92
7	15	Open	Natural	Sed	2.25
7	15	Open	Natural	Sed	1.40
7	15	Open	Natural	Sed	2.34
7	15	Open	Natural	Sed	1.68
7	15	Open	Natural	Sed	1.83
7	15	Open	Natural	Sed	1.10
7	15	Open	Natural	Sed	0.99
7	15	Open	Natural	Sed	1.75
7	15	Open	Natural	Sed	0.99
7	15	Open	Natural	Sed	0.95
7	15	Open	Natural	Sed	2.17
7	15	Open	Natural	Sed	1.48
7	15	Open	Natural	Sed	3.65
7	15	Open	Natural	Sed	0.90
7	32	Open	Natural	Turf	3.69
7	32	Open	Natural	Turf	1.80
7	32	Open	Natural	Turf	3.42
7	32	Open	Natural	Turf	3.38
7	32	Open	Natural	Turf	9.89
7	32	Open	Natural	Turf	5.66
7	32	Open	Natural	Turf	3.68
7	32	Open	Natural	Turf	3.08
7	32	Open	Natural	Turf	2.50
7	32	Open	Natural	Turf	10.22
7	32	Open	Natural	Turf	3.28
7	32	Open	Natural	Turf	6.53
7	32	Open	Natural	Turf	5.25
7	32	Open	Natural	Turf	1.23
7	32	Open	Natural	Turf	1.58
7	32	Open	Natural	Turf	3.13
7	32	Open	Natural	Turf	1.83
7	32	Open	Natural	Turf	2.23
7	32	Open	Natural	Turf	2.82
7	32	Open	Natural	Turf	2.34
7	32	Open	Natural	Sed	1.81

7	32	Open	Natural	Sed	2.08
7	32	Open	Natural	Sed	0.69
7	32	Open	Natural	Sed	1.21
7	32	Open	Natural	Sed	1.12
7	32	Open	Natural	Sed	1.12
7	32	Open	Natural	Sed	0.00
7	32	Open	Natural	Sed	1.21
7	32	Open	Natural	Sed	1.47
7	32	Open	Natural	Sed	0.78
7	32	Open	Natural	Sed	1.11
7	32	Open	Natural	Sed	1.01
7	32	Open	Natural	Sed	1.16
7	32	Open	Natural	Sed	1.83
7	32	Open	Natural	Sed	1.91
7	32	Open	Natural	Sed	2.13
7	32	Open	Natural	Sed	1.63
7	32	Open	Natural	Sed	2.93
7	32	Open	Natural	Sed	1.76
7	32	Open	Natural	Sed	1.57
7	20	Cage	Natural	Turf	5.60
7	20	Cage	Natural	Turf	4.97
7	20	Cage	Natural	Turf	2.06
7	20	Cage	Natural	Turf	7.52
7	20	Cage	Natural	Turf	8.22
7	20	Cage	Natural	Turf	3.65
7	20	Cage	Natural	Turf	4.69
7	20	Cage	Natural	Turf	6.90
7	20	Cage	Natural	Turf	4.98
7	20	Cage	Natural	Turf	7.46
7	20	Cage	Natural	Turf	9.74
7	20	Cage	Natural	Turf	7.65
7	20	Cage	Natural	Turf	3.33
7	20	Cage	Natural	Turf	3.54
7	20	Cage	Natural	Turf	5.99
7	20	Cage	Natural	Turf	2.62
7	20	Cage	Natural	Turf	4.30
7	20	Cage	Natural	Turf	3.21
7	20	Cage	Natural	Turf	5.70
7	20	Cage	Natural	Turf	5.05
7	20	Cage	Natural	Sed	1.87
7	20	Cage	Natural	Sed	2.09
7	20	Cage	Natural	Sed	3.76
7	20	Cage	Natural	Sed	1.93
7	20	Cage	Natural	Sed	1.04
7	20	Cage	Natural	Sed	2.76
7	20	Cage	Natural	Sed	2.45
7	20	Cage	Natural	Sed	1.04
7	20	Cage	Natural	Sed	1.72
7	20	Cage	Natural	Sed	2.20
7	20	Cage	Natural	Sed	1.49
7	20	Cage	Natural	Sed	1.80
7	20	Cage	Natural	Sed	1.33
7	20	Cage	Natural	Sed	1.28

7	20 Cage	Natural	Sed	2.91
7	20 Cage	Natural	Sed	2.52
7	20 Cage	Natural	Sed	1.09
7	20 Cage	Natural	Sed	3.34
7	20 Cage	Natural	Sed	1.18
7	20 Cage	Natural	Sed	2.27
7	35 Open	Addition	Turf	2.98
7	35 Open	Addition	Turf	2.06
7	35 Open	Addition	Turf	2.28
7	35 Open	Addition	Turf	3.34
7	35 Open	Addition	Turf	5.68
7	35 Open	Addition	Turf	3.73
7	35 Open	Addition	Turf	2.08
7	35 Open	Addition	Turf	2.15
7	35 Open	Addition	Turf	3.65
7	35 Open	Addition	Turf	5.61
7	35 Open	Addition	Turf	4.98
7	35 Open	Addition	Turf	2.80
7	35 Open	Addition	Turf	3.05
7	35 Open	Addition	Turf	2.56
7	35 Open	Addition	Turf	7.25
7	35 Open	Addition	Turf	8.73
7	35 Open	Addition	Turf	7.01
7	35 Open	Addition	Turf	2.22
7	35 Open	Addition	Turf	2.48
7	35 Open	Addition	Turf	4.69
7	35 Open	Addition	Sed	3.90
7	35 Open	Addition	Sed	1.13
7	35 Open	Addition	Sed	2.25
7	35 Open	Addition	Sed	2.23
7	35 Open	Addition	Sed	3.85
7	35 Open	Addition	Sed	2.48
7	35 Open	Addition	Sed	2.04
7	35 Open	Addition	Sed	3.94
7	35 Open	Addition	Sed	3.26
7	35 Open	Addition	Sed	3.46
7	35 Open	Addition	Sed	1.29
7	35 Open	Addition	Sed	1.38
7	35 Open	Addition	Sed	3.47
7	35 Open	Addition	Sed	7.08
7	35 Open	Addition	Sed	2.30
7	35 Open	Addition	Sed	1.89
7	35 Open	Addition	Sed	8.11
7	35 Open	Addition	Sed	3.12
7	35 Open	Addition	Sed	3.94
7	35 Open	Addition	Sed	2.12
7	31 Open	Natural	Turf	4.59
7	31 Open	Natural	Turf	3.72
7	31 Open	Natural	Turf	4.86
7	31 Open	Natural	Turf	2.07
7	31 Open	Natural	Turf	9.50
7	31 Open	Natural	Turf	9.31
7	31 Open	Natural	Turf	5.15

7	31	Open	Natural	Turf	3.33
7	31	Open	Natural	Turf	3.46
7	31	Open	Natural	Turf	2.72
7	31	Open	Natural	Turf	1.76
7	31	Open	Natural	Turf	3.32
7	31	Open	Natural	Turf	2.57
7	31	Open	Natural	Turf	1.17
7	31	Open	Natural	Turf	2.99
7	31	Open	Natural	Turf	4.16
7	31	Open	Natural	Turf	2.54
7	31	Open	Natural	Turf	2.66
7	31	Open	Natural	Turf	4.98
7	31	Open	Natural	Turf	5.45
7	31	Open	Natural	Sed	0.66
7	31	Open	Natural	Sed	1.57
7	31	Open	Natural	Sed	1.16
7	31	Open	Natural	Sed	0.85
7	31	Open	Natural	Sed	0.99
7	31	Open	Natural	Sed	1.14
7	31	Open	Natural	Sed	1.14
7	31	Open	Natural	Sed	1.67
7	31	Open	Natural	Sed	1.67
7	31	Open	Natural	Sed	1.61
7	31	Open	Natural	Sed	0.87
7	31	Open	Natural	Sed	1.20
7	31	Open	Natural	Sed	2.37
7	31	Open	Natural	Sed	1.79
7	31	Open	Natural	Sed	2.71
7	31	Open	Natural	Sed	0.80
7	31	Open	Natural	Sed	1.19
7	31	Open	Natural	Sed	1.31
7	31	Open	Natural	Sed	1.28
7	31	Open	Natural	Sed	1.48
7	30	Half	Addition	Turf	2.91
7	30	Half	Addition	Turf	2.18
7	30	Half	Addition	Turf	2.95
7	30	Half	Addition	Turf	1.40
7	30	Half	Addition	Turf	1.61
7	30	Half	Addition	Turf	2.73
7	30	Half	Addition	Turf	2.78
7	30	Half	Addition	Turf	3.01
7	30	Half	Addition	Turf	9.04
7	30	Half	Addition	Turf	2.65
7	30	Half	Addition	Turf	3.51
7	30	Half	Addition	Turf	1.82
7	30	Half	Addition	Turf	2.52
7	30	Half	Addition	Turf	2.22
7	30	Half	Addition	Turf	2.56
7	30	Half	Addition	Turf	4.70
7	30	Half	Addition	Turf	2.17
7	30	Half	Addition	Turf	2.11
7	30	Half	Addition	Turf	2.98
7	30	Half	Addition	Turf	4.36

7	30	Half	Addition	Sed	4.74
7	30	Half	Addition	Sed	6.18
7	30	Half	Addition	Sed	1.76
7	30	Half	Addition	Sed	1.15
7	30	Half	Addition	Sed	0.84
7	30	Half	Addition	Sed	1.88
7	30	Half	Addition	Sed	0.80
7	30	Half	Addition	Sed	0.55
7	30	Half	Addition	Sed	3.73
7	30	Half	Addition	Sed	3.12
7	30	Half	Addition	Sed	2.47
7	30	Half	Addition	Sed	2.87
7	30	Half	Addition	Sed	1.66
7	30	Half	Addition	Sed	2.56
7	30	Half	Addition	Sed	0.00
7	30	Half	Addition	Sed	1.97
7	30	Half	Addition	Sed	1.38
7	30	Half	Addition	Sed	1.84
7	30	Half	Addition	Sed	1.68
7	30	Half	Addition	Sed	1.17
