# Supplementary material for: Ecological Consequences of Sediment on High-Energy Coral Reefs
Source: PLoS One. 2013 Oct 4;8(10):e77737. doi: 10.1371/journal.pone.0077737 (PMC3790735; doi:10.1371/journal.pone.0077737)
Supplement: Table S2 — 2-way ANOVA of T7 turf length data (square root x + 21.0633 transformed data, sites pooled). (PDF) [file pone.0077737.s002.pdf]

Table S2: 2-way ANOVA of T_7 turf length data (square root x + 21.0633 transformed data, sites pooled)

	SS	df	MS	F	P
Cage	82.595	2	41.298	6.309	0.005
Sediment	16.180	1	16.180	2.472	0.126
Cage × Sediment	12.300	2	6.150	0.940	0.402
Error	196.368	30	6.546		
