# Supplementary material for: Ecological Consequences of Sediment on High-Energy Coral Reefs
Source: PLoS One. 2013 Oct 4;8(10):e77737. doi: 10.1371/journal.pone.0077737 (PMC3790735; doi:10.1371/journal.pone.0077737)
Supplement: Table S3 — RM MANOVA results of EAM depth over time, across caging and sediment treatments, pooled between sites (Figure 3). Assumptions of sphericity were violated by univariate tests, as such multivariate tests (Pillai’s Trace) were used. (PDF) [file pone.0077737.s003.pdf]

Table S3: RM MANOVA results of EAM depth over time, across caging and sediment treatments, pooled between sites (Fig. 3). Assumptions of sphericity were violated by univariate tests, as such multivariate tests (Pillai's Trace) were used.

	Sphericity			
	W	χ^2	df	*P*
Time	0.31	32.54	20	0.038

	Multivariate RM ANOVA				
	Pillai's trace	*F*	Effect	Error	*P*
Time	0.65	7.64	6	25	<0.0001
Time × Cage	0.64	2.02	12	52	0.041
Time × Sed	0.08	0.36	6	25	0.900
Time × Cage × Sed	0.46	1.31	12	52	0.243
