# Supplementary material for: Ecological Consequences of Sediment on High-Energy Coral Reefs
Source: PLoS One. 2013 Oct 4;8(10):e77737. doi: 10.1371/journal.pone.0077737 (PMC3790735; doi:10.1371/journal.pone.0077737)
Supplement: Table S4 — RM MANOVA results of sediment depth over time, across caging and sediment treatments, pooled between sites (Figure 4). Assumptions of sphericity were violated by univariate tests, as such multivariate tests (Pillai’s Trace) were used. (PDF) [file pone.0077737.s004.pdf]

Table S4: RM MANOVA results of sediment depth over time, across caging and sediment treatments, pooled between sites (Fig. 4). Assumptions of sphericity were violated by univariate tests, as such multivariate tests (Pillai's Trace) were used.

Sphericity				
	W	χ^2	df	*P*
Time	0.01	92.45	27	0.000

Multivariate RM ANOVA					
	Pillai's trace	*F*	Effect	Error	*P*
Time	0.97	82.01	7	18	<0.0001
Time × Site	0.90	24.16	7	18	<0.0001
Time × Cage	0.92	2.33	14	38	0.020
Time × Sed	0.95	51.59	7	18	0.000
Time × Site × Cage	1.02	2.84	14	38	0.005
Time × Site × Sed	0.84	13.90	7	18	<0.0001
Time × Cage × Sed	0.87	2.09	14	38	0.036
Time × Site × Cage × Sed	0.87	2.11	14	38	0.035
